# The Water Microbiome Through a Pilot Scale Advanced Treatment Facility for Direct Potable Reuse

**DOI:** 10.3389/fmicb.2019.00993

**Published:** 2019-05-08

**Authors:** Rose S. Kantor, Scott E. Miller, Kara L. Nelson

**Affiliations:** ^1^Department of Civil and Environmental Engineering, University of California, Berkeley, Berkeley, CA, United States; ^2^Engineering Research Center for Re-inventing the Nation’s Urban Water Infrastructure, Berkeley, CA, United States

**Keywords:** direct potable reuse, metagenomics, 16S rRNA gene sequencing, drinking water microbiome, advanced water treatment, antibiotic resistance

## Abstract

Advanced treatment facilities for potable water reuse of wastewater are designed to achieve high removal levels of specific pathogens, as well as many other constituents. However, changes to the microbial community throughout treatment, storage, and distribution of this water have not been well characterized. We applied high-throughput amplicon sequencing, read-based, assembly-based, and genome-resolved metagenomics, and flow cytometry to investigate the microbial communities present in a pilot-scale advanced water treatment facility. Advanced treatment of secondary-treated wastewater consisted of ozonation, chloramination, microfiltration, reverse osmosis (RO), advanced oxidation (UV/H_2_O_2_), granular activated carbon (GAC) filtration, and chlorination. Treated water was fed into bench-scale simulated distribution systems (SDS). Cell counts and microbial diversity in bulk water decreased until GAC filtration, and the bacterial communities were significantly different following each treatment step. Bacteria grew within GAC media and contributed to a consistent microbial community in the filtrate, which included members of the *Rhizobiales* and *Mycobacteriaceae*. After chlorination, some of the GAC filtrate community was maintained within the SDS, and community shifts were associated with stagnation. Putative antibiotic resistance genes and potential opportunistic pathogens were identified before RO and after advanced oxidation, although few if any members of the wastewater microbial community passed through these treatment steps. These findings can contribute to improved design of advanced treatment trains and management of microbial communities in post-treatment steps.

## Introduction

Regions facing water scarcity are turning to new sources of drinking water, including wastewater that has been purified through advanced treatment to meet potable water quality standards (Raucher and Tchobanoglous, 2014). Advanced treatment follows secondary or tertiary wastewater treatment and typically includes MF and RO, followed by advanced oxidation (UV/H_2_O_2_) to degrade remaining chemical contaminants and inactivate pathogens (Gerrity et al., 2013).

In direct potable reuse systems, purified water is blended into the drinking water distribution system after minimal storage time, and therefore microbial risks must be considered carefully. Current regulations focus on virus and protozoan cyst removal, and ensuring that the risk level for these pathogens in advanced treated water is as low as for conventional drinking water. For example, California has implemented the so-called 12/10/10 rule for indirect potable reuse, requiring treatment trains to meet specific log_10_ removals of viruses, *Cryptosporidium* oocysts, and *Giardia* cysts, respectively (California Code of Regulations, 2014), and regulations for direct potable reuse are under development (State Water Resources Control Board, 2016; Pecson et al., 2017). However, bacteria are also of concern, and bacterial communities established during treatment have been shown to influence communities found in distributed water (Pinto et al., 2012). Critically, unlike human viruses and enteric protozoa, bacteria can replicate during and after treatment, and their growth is dependent on a variety of factors including disinfectant residual and nutrient concentrations during distribution (Nescerecka et al., 2014; Prest et al., 2016a,b). To understand how advanced treatment affects microbial water quality, it is necessary to examine removal and growth of bacteria across treatment trains and in distribution.

In addition to culture-based methods (e.g., heterotrophic plate counts) and direct biomass quantification methods (e.g., adenosine triphosphate and flow cytometry), water engineers are increasingly making use of high-throughput DNA sequencing technologies and microbial ecology analyses to study the effects of drinking water treatment and distribution on microbial communities. Amplicon sequencing is used to inventory the microbial species present in water or biofilm in terms of taxonomic identity and relative abundance through use of a common marker sequence, typically one or several regions of the 16S ribosomal RNA (rRNA) gene (Vignola et al., 2017; Liu et al., 2018). Metagenomics, the reconstruction of genes and genomes from uncultured environmental microorganisms, has also been applied to drinking water treatment and distribution (Pinto et al., 2016; Zhang et al., 2017; Oh et al., 2018). However, there are few studies of microbial communities in the water of potable reuse treatment trains and in distribution systems fed with advanced purified water (Salveson et al., 2018; Stamps et al., 2018). Given that variations in treatment design and post-treatment processes may impact microbial communities, multiple studies of different treatment trains will be needed to advance the field.

We studied a pilot-scale advanced water treatment facility in El Paso, Texas. Here, we report on DNA sequencing-based analyses of microbial communities sampled across the advanced treatment train and chlorinated SDS fed with the advanced treated water. In a separate manuscript, we will report in more detail on changes in total and intact cells via flow cytometry, as well as metrics of microbial growth capacity.

With our analyses, we demonstrate the utility and pitfalls of high-throughput sequencing to study potable reuse treatment trains and simulated distribution, in which: (1) low-biomass samples are highly sensitive to contamination; (2) high resolution of sequences is critical; and (3) the engineering goals require information about absolute abundance. To meet these challenges, we report observations about our sequencing controls, make use of recent advances allowing resolution of Amplicon Sequencing Variants (ASVs) that in some cases correspond to near-complete MAGs, and combine sequencing-based relative abundance with absolute cell counts. We use this information to examine changes in microbial community composition through advanced treatment and water distribution to identify populations that may persist through treatment, and to search for potential pathogens. We use metagenomic data to investigate antibiotic resistance potential before and after treatment and to explore possible reasons for the growth of specific organisms in highly purified water.

## Materials and Methods

### Experimental Facilities

We sampled a pilot-scale advanced purification facility in El Paso, Texas that operated from June 8, 2015 to January 29, 2016 and treated 0.14 million gallons per day. The feed to the plant was secondary-treated wastewater, which was ozonated to a target concentration of 3.5 mg/L (∼5 min storage time, after which there was no detectable residual) and chloraminated to a target residual of 2–4 mg/L as Cl_2_ to reduce fouling of the MF membranes. The flow was split and treated by parallel MF units (Pall module type UNA-620A and Evoqua Memcor CPII L40N), and effluents were recombined in a storage tank. Water leaving the storage tank was then split equally and fed into parallel NF (Dow, model NF90-400/34i) and RO (Hydranautics, model ESPA2-LD) processes. The recombined flow was treated by an ultraviolet/advanced oxidation process (UV/AOP). UV was supplied via Trojan UV Swift SC B08^TM^ at a dose of 840 mJ/cm^2^, and oxidation was achieved by addition of hydrogen peroxide to a target concentration of 4 mg/L. UV/AOP treated water was then split for parallel filtration by three types of GAC (see Supplementary Methods).

On alternating days, effluents from each of the GAC filters were collected in sterile bottles, transported on ice to the laboratory, and chlorinated within 6 h in a 5 L glass reservoir using sodium hypochlorite (ACS grade, Spectrum Chemical MFG Corp.) to achieve a CT of 30 mg-min/L and a free chlorine residual of approximately 0.8–1.0 mg/L as Cl_2_ at the end of the 30-min batch chlorination (unless otherwise noted). Chlorinated water was then transferred to three separate storage containers and continuously pumped to three annular reactors to simulate distribution (referred to throughout as SDS). Prior to operation, the SDS were sterilized and prepared as previous described (Wang et al., 2013). SDS were operated in the dark at a hydraulic residence time of 18 h (flow rate ∼0.92 mL ⋅ min^−1^) and ambient temperature of ∼22°C. The inner cylinder rotation speed was set to 50 rpm, resulting in a shear stress of ∼0.15 N ⋅ m^−2^ on the inner cylinder surface. All components of the simulated system (including the chlorination and storage reservoirs) were enclosed in aluminum foil to block light. Reactor operation is further described in Supplementary Methods. Several days before the end of SDS operation, SDS reactors were intentionally left stagnant for 24 h, after which feeding and mixing were resumed to allow stagnated reactor effluent to be sampled. Stagnation was induced only this once in the entire SDS operation.

### Sample Collection

**FIGURE 1:**
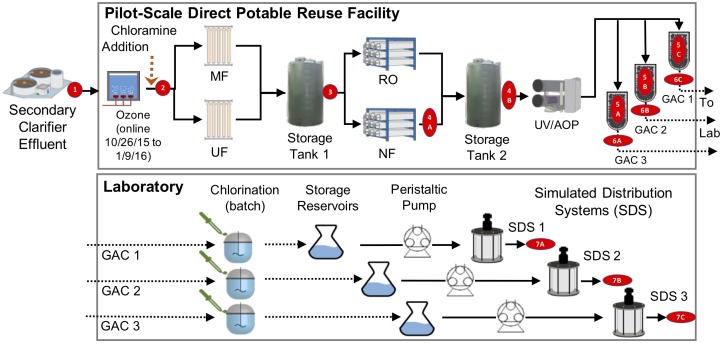
Treatment train and experimental design. Secondary treated wastewater was fed to a pilot-scale advanced water treatment train which included parallel microfiltration (MF) and ultrafiltration (UF), parallel nanofiltration (NF), and reverse osmosis (RO), UV/advanced oxidation process (AOP), and three parallel granular activated carbon columns (see Supplementary Methods). Distribution was modeled by three parallel flows through batch chlorination, storage, and annular reactors. Red numbers indicate sample locations corresponding to identifiers in Table 1.

Bulk water was sampled weekly from November 2015 through January 2016, during months 6–8 of pilot facility operation. Samples were collected in autoclave-sterilized 1-L Nalgene bottles from secondary wastewater feed to the pilot facility and from taps located after each treatment step: after chloramination, post-MF storage tank, NF permeate, combined storage after parallel NF/RO, after each of three parallel GAC column filters, and after each of three parallel SDS (Figure 1 and Table 1). Samples with residual chloramine disinfectant were quenched with excess sodium thiosulfate. Sampling of just the RO permeate did not yield sufficient DNA for sequencing, and therefore sampling was changed to the NF/RO combined tap in an attempt to concentrate more biomass onto a single filter. Sample taps were flushed for following periods: secondary wastewater and chloramine (>5 min), MF/UF filtrate (>15 min), NF and NF/RO combined permeate (>30 min), and GAC filtrate (>15 min). Water samples were filtered onto 0.22 μm mixed cellulose ester filters (Millipore) (see Supplementary Table S1 for filtration volumes). All filtration equipment (glass vacuum flask, glass funnel, glass filter supports) was autoclave-sterilized prior to use. Filters were handled using flame-sterilized tweezers. Filtered samples were stored in sterile 5-mL screw cap tubes (Axygen^TM^, Thermo Fisher Scientific).

**Table 1:** Counts and locations of samples.

Location	Identifier (Figure 1)	Total amplicon	Post-QC amplicon	Metagenomics
Secondary wastewater	1	6	6	3
Chloramine	2	4	3	–
MF	3	5	4	–
NF/RO	4A, 4B	3	2	–
GAC filtrate (3)	5A, 5B, 5C	15	14	3
GAC media (3)	6A, 6B, 6C	12	–	3
SDS (3)	7A, 7B, 7C	11	8	3
Amplification blank		3	1	–
Extraction blank		3	1	–
Field blank		3	–	–
Zymobiomics mock		2	2	1

Total and intact cell counts in bulk water grab samples were determined by flow cytometry as described in Supplementary Methods. Biofilm samples were collected during plant shutdown by swabbing the internal surfaces of pipes downstream from each treatment process using sterile cotton swabs, but samples did not yield enough DNA for further analysis (data not shown). GAC media samples were collected by disassembling GAC columns immediately following the end of plant operation on January 28, 2016 (day 234 of the study). Media was poured into 50 mL tubes and stored at −80°C until DNA extraction. Media from columns 1 and 2 was from the top of the column, while column 3 was sampled from the middle. Sampling information is summarized in Table 1 and presented in detail in Supplementary Table S1.

### DNA Extraction, Library Preparation, and Sequencing

An optimized nucleic acid extraction protocol was used on the concentrated bulk water samples because very low biomass was expected in some pilot facility and annular reactor bulk water samples. Briefly, filter membranes were torn and placed into Lysing Matrix A tubes (MP Biomedicals) with flame-sterilized tweezers. A modified phenol-chloroform-isoamyl alcohol extraction was used as previously described, with the exception of the 70°C water bath pre-heat of the filter (Pinto et al., 2012; Bautista-de Los Santos et al., 2016). Field blanks were created by filtering 1 L of autoclaved de-ionized water onto 0.22 μm mixed cellulose ester filters (Millipore) and then processed identically to field samples.

Genomic DNA (gDNA) was extracted from the GAC media samples using the MoBio DNeasy PowerSoil Kit (Qiagen) according to manufacturer’s instructions, with the following modifications: samples were incubated in lysis buffer at 65°C for 15 min prior to proceeding with the extraction protocol, bead-beating was performed at maximum speed for 10 min using a vortex adapter, the two inhibitor removal steps were combined into a single step to limit loss of gDNA, and the spin filter was incubated at 55°C for 10 min with nuclease-free water prior to elution to increase gDNA eluted. Extraction blanks were created by processing PCR-grade water alongside with and identically to field samples. Media samples yielded DNA for both amplicon and metagenomic sequencing. The amplicon libraries suffered from contamination and were excluded from analysis.

Extraction yields were assessed via Qubit dsDNA HS assay kit (Thermo Fisher Scientific). When necessary, gDNA was concentrated using a SpeedVac, and inhibitors were reduced with OneStep PCR Inhibitor Removal Kit (Zymo Research, Irvine, CA, United States).

Library preparation for amplicon sequencing followed the Schloss Lab MiSeq wet-lab protocol for amplification of the V4 region of the 16S rRNA gene^1^ (Kozich et al., 2013) with slight modification (Supplementary Table S2). Briefly, the V4 region was amplified using uniquely barcoded 515F and 806R primers with Phusion Hot Start II polymerase, HF buffer, dimethyl sulfoxide (3.75% final concentration) and 1–2 μl of genomic DNA. Triplicate 25 μl reactions were combined and concentrated by SpeedVac. For difficult reactions, up to five replicates were pooled. The dual-barcoded libraries (including sampling, extraction, and PCR negative control libraries) were normalized using the SequalPrep Normalization Plate kit (Invitrogen), pooled, and sequenced on a MiSeq with V3 chemistry for 600 cycles, yielding paired-end 250 bp reads. Mock-community control libraries were constructed using the ZymoBIOMICS Microbial Community DNA Standard (Zymo Research, Irvine, CA, United States) and included in amplicon and metagenomic sequencing (Supplementary Figure S1).

Library preparation for metagenomic sequencing was performed at the Functional Genomics Laboratory at UC Berkeley. Briefly, 500 bp insert libraries were constructed using the KAPA Hyper prep kit with a PCR amplification step. Sequencing was performed on an Illumina HiSeq 4000 at the Vincent J. Coates Genomics Sequencing Laboratory at UC Berkeley, yielding 150 bp paired-end reads.

### Amplicon Sequence Data Processing

Reads were demultiplexed and mapped to PhiX (21.7%). Forward reads were processed using the DADA2 pipeline v1.4.0 (Callahan et al., 2016) according to http://benjjneb.github.io/dada2/bigdata.html to produce ASVs. Briefly, based on evaluation of read quality using FastQC^2^, reads were truncated to 230 nucleotides (nt) and left-trimmed to remove 15 nt from the 5′ end, resulting in 215 nt sequences. Reads were also truncated where quality dipped below *Q* = 11, and any reads <200 nt were removed. On average 70% of reads remained after trimming. Error modeling used the first 2 million filtered reads, and samples were denoised individually using this error model. The denoised sequences were screened for chimeras with removeBimeraDenovo, which removed 3153 sequences accounting for 4.3% of all reads. Taxonomy was assigned using the Ribosomal Database Project’s naive Bayesian classifier (Wang et al., 2007) within DADA2. The classifier was trained on Silva v128 (Quast et al., 2013) and set to use a minimum bootstrap confidence of 50 to assign a given rank (default minBoot = 50). Error-corrected, taxonomically classified data were analyzed with the Phyloseq (v1.20.0) package in R (McMurdie and Holmes, 2013) (see Supplementary Data Sheet S2 and https://github.com/rosekantor/16S_metagenomics_notebooks).

Data from bulk water samples and corresponding field, extraction, and amplification negative controls were prefiltered for successful sequencing (>10000 reads), and low-biomass samples were removed prior to analyses due to obvious contamination with secondary wastewater (two samples) and MF filtrate (one sample). This prefiltering yielded 37 samples and 3 negative controls. DESeq2 analysis was performed with all ASVs found in any prefiltered negative control or sample. Of 108 ASVs that were shared between any sample and any negative control, 7 were significantly enriched in samples over negative controls (DESeq2 p-adjust <0.01). These may represent cross-contamination of negative controls with samples or different but closely related organisms present in samples and negative controls. We removed from samples all ASVs found in negative controls except for these 7. After filtering contaminant reads, one sample and one negative control with fewer than 300 reads were removed, and the two remaining amplification and extraction controls contained 2 and 3 ASVs, respectively.

Rarefaction curves were plotted for bulk water samples using *rarecurve* in the vegan R package (v2.4.4). The DADA2 pipeline removes singletons because they cannot be reliably distinguished from sequencing errors, precluding the use of alpha diversity metrics that rely on singletons. Given that the rarefaction curves plateau for all samples (Supplementary Figure S2), total observed ASVs were used as a metric of alpha diversity. Beta diversity was analyzed via principle coordinates analysis (PCoA) using Bray-Curtis dissimilarity and variables contributing to differences between communities were identified with PERMANOVA using *adonis* in the vegan package for R. Additional PERMANOVA testing using Aitchison distance found the same level of significance as was observed when using Bray-Curtis dissimilarity of percent relative abundance data. Calculating and testing for homogeneity of group dispersions used the *betadisper* and *permutest* functions within the vegan package in R (groups were sample locations).

To estimate abundances of genera containing opportunistic pathogens, the relative abundances of all ASVs (>0.05% relative abundance in any sample) belonging to the genera *Legionella* and *Mycobacterium* were summed for each sample at each location in the treatment train. The minimum, median, and maximum relative abundances were then determined for each location. To generate the range of absolute abundances, we multiplied minimum, median, and maximum relative abundances by the minimum, median, and maximum total cell counts ever observed at a given location across the duration of the study.

### Metagenomic Sequence Processing

Metagenomic read processing used FastQC (Babraham Bioinformatics, see text footnote 2) to inspect quality, bbmap (Bushnell^3^) to remove PhiX and adapter sequences, and sickle (Joshi and Fass, 2011) for quality trimming. Read-based phylogenetic characterization was performed with MetaPhlAn2 using default parameters (Truong et al., 2015). Assembly was performed independently for each sample using idba_ud (Peng et al., 2012) with the “–pre_correction” option. Annotation of scaffolds ( 1 kbp was performed with Prokka v1.12 (Seemann, 2014) and RNAmmer (Lagesen et al., 2007).

Metagenomic analysis was conducted in Anvi’o (Eren et al., 2015) according to the metagenomic workflow for v3 and v4. For each assembly (scaffolds ≥2.5 kbp), an Anvi’o contig database was generated and a profile database was created that included read-mapping information from each sample. Taxonomy was added using Centrifuge (v1.0.2-beta). Binning was performed manually based on hierarchical clustering by sequence composition and differential coverage in Anvi’o. Genome bins >70% complete with <10% contamination according to CheckM (Parks et al., 2015) were de-replicated to generate a non-redundant set of MAGs using dRep (Olm et al., 2017) with primary clustering at 95% ANI and secondary clustering at 99% ANI, requiring 60% coverage of the larger genome in each pairwise comparison. MAGs were taxonomically identified based on their position in a concatenated gene tree (see Supplementary Methods), Centrifuge results, and CheckM taxonomy placement (Supplementary Tables S3, S4). Non-redundant MAGs were curated to remove misassemblies using ra2.py (Brown et al., 2015)^4^ and open reading frames were predicted using Prodigal in single genome mode (Hyatt et al., 2010). Annotation of predicted proteins was conducted using USEARCH (Edgar, 2010) against the KEGG database (Kanehisa et al., 2016). C1-carbon utilization was determined by manual searches of KEGG-based annotations against select proteins representative of the pathways of interest (Supplementary Table S5).

To investigate shared microbial community members across samples, reads from each sample were stringently mapped to (1) assemblies of each sample (scaffolds ≥1 kbp), (2) scaffolds ≥1 kbp containing unique RpsC sequences, and (3) the non-redundant MAGs (see Supplementary Methods). Stringent mappings were also used to calculate indices of replication (iRep values) (Brown et al., 2016) for each MAG in each sample using iRep^5^ with default settings.

Antibiotic resistance genes were detected via HMMsearch of the predicted proteins against the ResFams core database of hidden Markov models (HMMs) (Gibson et al., 2015). Within each resistance gene family, ResFam hits were sorted by length and clustered at 99% identity using USEARCH -cluster_fast (Edgar, 2010). Tabulated results were searched for any clusters containing proteins from both secondary wastewater and post-NF/RO samples. Ammonium monooxygenase genes were identified using TIGRFAM HMMs, and custom HMMs were used to search for Adenovirus and JC Polyomavirus sequences (see Supplementary Methods).

A phylogenomic tree for the Mycobacteriaceae was constructed with the Anvi’o phylogenomics pipeline, using thirty reference genomes downloaded from NCBI representing each of the major clades described in Gupta et al. (2018). A subset of 108 of the single copy genes/domains from Campbell et al. (2013) were identified via HMMsearch (Eddy, 2009), and individual alignments were constructed with MUSCLE (Edgar, 2004). A concatenated alignment was manually curated to remove C- and N-termini overhangs and regions of poor alignment. The resulting alignment was used to construct a phylogenetic tree with RAxML (Stamatakis, 2014) using 100 bootstraps.

## Results

Bulk water was sampled over a 3-month span at a pilot-scale advanced treatment facility that processed secondary wastewater to potable quality via ozonation, chloramination, MF, RO or NF, UV-hydrogen peroxide AOP, and GAC filtration (Figure 1). Samples were collected after each treatment process, and sample totals for 16S rRNA gene (V4 region) amplicon and metagenomic sequencing are shown in Table 1. GAC media were also sampled and passed QC for metagenomics but not amplicon sequencing (Table 1). Amplicon sequencing for 37 bulk water samples yielded a total of 2900 unique ASVs. After data decontamination based on negative controls (see methods), sample read-counts ranged from 3499 to 78462, and negative controls contained fewer than 1000 reads. The majority of samples were taken between days 184–228 of pilot plant operation.

Metagenomic sequencing was performed for 12 samples including three secondary wastewater feed samples from different days, GAC filtrates sampled from three parallel columns on the same day, GAC media samples from three parallel columns on the same day, and bulk water samples from three SDS on the same day (Table 1 and Supplementary Table S3). Illumina paired-end sequencing of total genomic DNA yielded between 3.45 and 7.05 Gbp of raw reads per sample, and between 52 and 94% of trimmed reads could be mapped to their respective assemblies (scaffolds ≥1 kbp). Hierarchical clustering based on differential coverage and sequence composition aided in reconstruction of 317 bins across all samples, and de-replication of 72 high-quality bins resulted in a set of 38 non-redundant MAGs (Supplementary Tables S3, S4). These bins collectively represented as little as 8% of the wastewater community (these assemblies had many incomplete bins), but as much as 73% of the GAC filtrate and SDS communities, based on percentages of reads mapped.

### Microbial Diversity and Absolute Abundance

**FIGURE 2:**
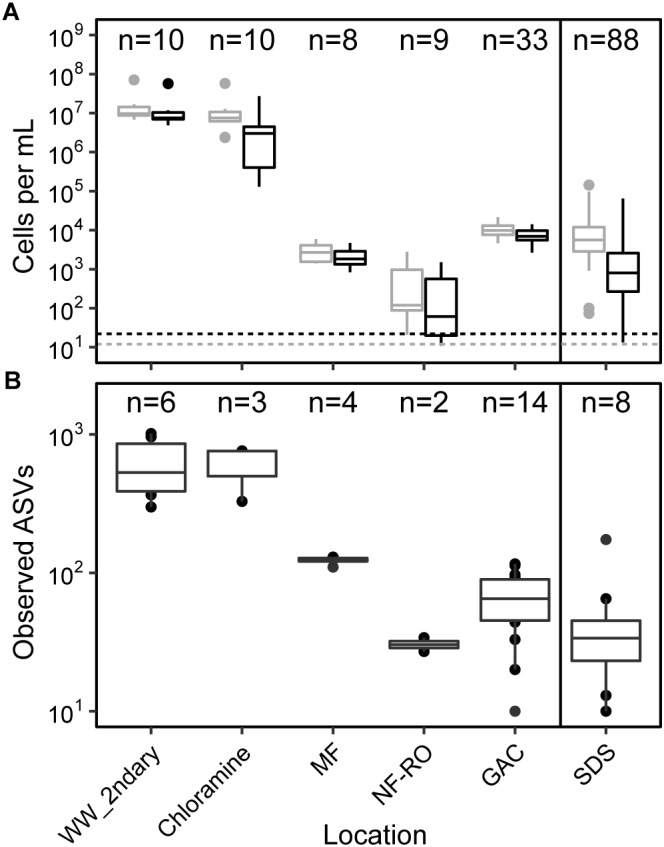
Cell counts and diversity in bulk water across the treatment train. **(A)** Counts of total (gray) and intact (black) cells based on flow cytometry, and **(B)** counts of observed amplicon sequence variants (ASVs). Solid line in both panels indicates chlorination and storage prior to feeding into simulated distribution systems (SDS). Dashed lines indicate minimum detection limits for total and intact cell counts (see Supplementary Table S7 for supporting data). Sample numbers for flow cytometry and amplicon sequencing (after data decontamination) are noted for each location. In panel **(A)**, MF and NF/RO cell counts represent interprocess storage tanks after the respective process.

Absolute abundances and alpha diversity of the bacterial community followed similar trajectories throughout the treatment train. Flow-cytometric cell counts decreased from secondary wastewater through NF/RO and then increased after GAC filtration, indicating growth on filter media (Figure 2A). Cell counts in GAC filtrate and SDS were 3 to 4 log_10_ lower than the secondary wastewater feed. Despite chlorination following GAC filtration, the median intact cell count in the three SDS was 2.7 × 10^3^ cells ⋅ mL^−1^. Intact cell counts were often lower than total cell counts, indicating that some sequence information was derived from non-viable cells. Median richness decreased across the treatment train with a slight increase after GAC (Figure 2B). Observed ASVs were used as a proxy for richness within each sample because rarefaction curves plateaued for nearly all bulk water samples (Supplementary Figure S2) and singletons had been removed during data processing. Between secondary wastewater and SDS, median observed ASVs decreased from 536 to 34 (a 1.09 log_10_ reduction) (Figure 2B).

### Microbial Communities Across Treatment and Simulated Distribution

**FIGURE 3:**
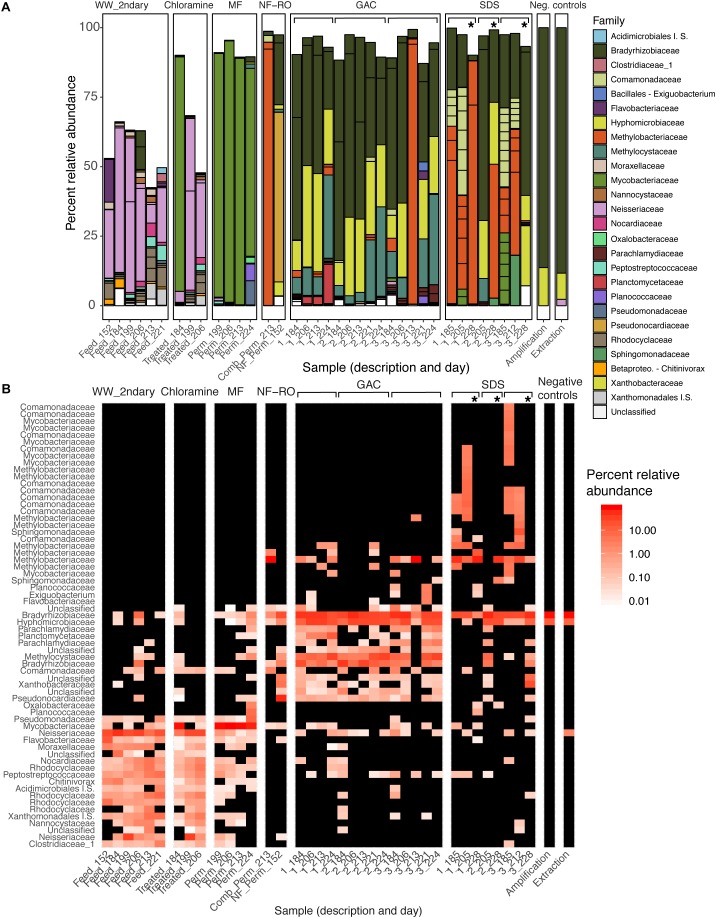
Changes in the bacterial composition of bulk water through advanced treatment and simulated distribution. **(A)** Barplot and **(B)** heatmap of amplicon data across the treatment train. **(A)** Samples are bars grouped by location in the treatment train. Bar height indicates relative abundances and Family-level taxonomy of ASVs, and each segment within a bar represents a unique ASV and is colored by Family (I.S., “Incertae Sedis”). **(B)** Samples are columns and ASVs are rows. Color indicates percent relative abundance on a log-scale (black indicates non-detection). Both panels report the same 64 ASVs present at a minimum relative abundance of 2% in at least one sample (see Supplementary Table S6 for additional data). Sample descriptions (*x*-axis) indicate secondary wastewater entering advanced treatment (“Feed”), chloramine treated (“Treated”), MF permeate in storage tank (“Perm”), NF permeate in storage tank (“Comb_Perm”), NF permeate (“NF_Perm”), filtrate from one of three parallel GAC filters (1–3, grouped by brackets), or bulk water from one of three SDS (1–3, grouped by brackets). Sample day (days since pilot plant start-up) is indicated following the underscore for each sample. Asterisks indicate samples taken from the three SDS after 24-h stagnation.

**FIGURE 4:**
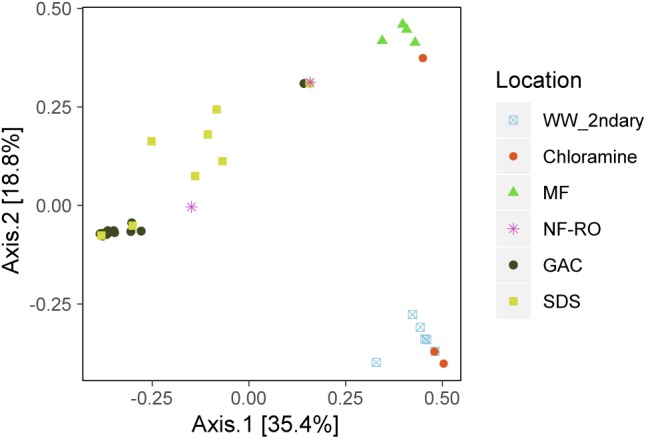
Bacterial community dissimilarity by location. Principle coordinate analysis (PCoA) of amplicon data based on Bray-Curtis dissimilarity. Locations within the advanced treatment train are indicated by color and shape and include secondary wastewater (“WW_2ndary”), chloramine treated (“Chloramine”), MF permeate in storage tank (“MF”), NF permeate and NF permeate in storage tank (“NF-RO”), filtrates from three parallel GAC filters (“GAC”) and bulk water from three SDS (“SDS”).

There were significant differences in the taxonomic composition of bulk water bacterial communities from different sample locations (Figure 3A,B). By principal coordinate analysis (PCoA), bacterial communities clustered by location within the treatment train (Figure 4). This clustering was supported by PERMANOVA, which suggested differences between communities were partially explained by sample location (PERMANOVA *R*^2^ = 0.57; *p* < 0.001; same level of significance using proportion-normalized or rarefied data), but not sample date (*R*^2^ = 0.15; *p* < 0.08). Some of the significance of sample location in this test may be due to heterogeneous group dispersions of samples within different locations (permutest *p* = 0.053 with 10000 permutations) (Supplementary Figure S3). Notably, MF and GAC had the lowest distance to the centroid of their groups, suggesting the most consistent communities. NF/RO permeate and negative controls did not include enough successfully amplified and decontaminated samples to provide information about within-location variability (*n* = 2 for each).

After each treatment step, the taxonomy of the most prevalent ASVs changed, and overall membership also changed (Figure 3B). One exception was that secondary wastewater communities appeared similar to some samples taken after chloramine treatment. This effect might be expected as ozonation and chloramination inactivated cells (Figure 2A) but may not have significantly damaged their DNA. Relative abundances and phylogenetic classifications based on metagenomic data yielded a somewhat similar picture of the microbial community, except for among low-biomass samples. We used a read-based method (MetaPhlAn2) (Truong et al., 2015; Supplementary Figure S4), unique RpsC sequences from assembled data (Supplementary Figure S5), and non-redundant MAGs from across all samples (Figure 6A) to assess the community composition and structure within metagenomes compared with amplicon data.

In the secondary wastewater before and after chloramine treatment, the dominant ASVs (as high as 57% relative abundance), most abundant RpsC sequence, and most abundant MAGs corresponded to the family Neisseriaceae. Meanwhile, MetaPhlAn2 identified Rhodocyclaceae as the most abundant Family in secondary wastewater (Supplementary Figure S4). One post-chloramine sample and all MF filtrate samples were overwhelmingly dominated by a single ASV classified as Mycobacteriaceae. In the NF permeate sample, the most prevalent ASV was classified as Pseudonocardiaceae (61.0% relative abundance), while in the NF/RO permeate storage tank, an ASV classified as Methylobacteriaceae was highly abundant (92.1% relative abundance) (Figure 3A). Abundant ASVs, metagenomic reads, RpsC sequences, and MAGs in the GAC filtrates were classified as Rhizobiales (including Bradyrhizobiaceae, Hyphomicrobiaceae, Methylocystaceae, and Methylobacteriaceae) (Figure 3A, 6 and Supplementary Figures S4, S5). The SDS communities were more variable over time than GAC communities, likely due to variation of the chlorine residual and a 24-h stagnation event just prior to the final sampling (day 227). In the two sample days prior to stagnation, SDS 1 and SDS 3 communities contained abundant ASVs, RpsCs, and MAGs classified as Burkholderiales/Comamonadaceae, Methylobacteriaceae, and Bradyrhizobiaceae. By amplicon sequencing, the community in SDS 2 prior to stagnation (SDS 2, day 205) appeared similar to the GAC filtrate (Figure 3), but by all metagenomic analyses, the same sample appeared more similar to the other SDS samples prior to stagnation (Supplementary Figures S4, S5 and Figure 6A). Based on our amplicon data with limited sampling, all three SDS communities were different before and after stagnation (Figure 3A,B).

Seven ASVs found in negative controls and in samples from all locations were not removed during data decontamination because they were significantly enriched (adjusted *p* < 0.005) in samples relative to controls. Two of these ASVs, classified as *Bradyrhizobium* and Hyphomicrobiaceae, were abundant in low biomass samples, especially GAC filtrates (33 and 22% median relative abundances) and some SDS samples. In metagenomic data, these two taxa were also highly abundant in GAC media and filtrate but not SDS (Supplementary Figure S5 and Figure 6A).

In addition to bacterial genomes, several eukaryotic nuclear and plastid sequences were detected with metagenomics. Most notably, all three GAC media metagenomes contained identical sequences from a single Chlorophyta bin. Based on best BLAST hits of chloroplast 16S, 23S, and co-binned nuclear 28S rRNA genes to NCBI-nr, the genome was likely related to the Sphaeropleales. The chloroplast RpsC sequence from this bin was detected by read-mapping in all three GAC media metagenomes (Supplementary Figure S5), and the presence of algae is consistent with observations of green growth on walls of the media columns. An additional Chlorophyta species with 100% identity to *Parachlorella kessleri* across both the chloroplast 16S rRNA gene and RpsC sequence (NCBI accessions FJ968741.1 and YP_003058311.1) was present in the metagenome of only GAC media from column 3 (Supplementary Figure S5), and this column was designed to filter water 69% more slowly than the others (see Supplementary Methods).

### Persistence of Bacteria Through Advanced Treatment

**FIGURE 5:**
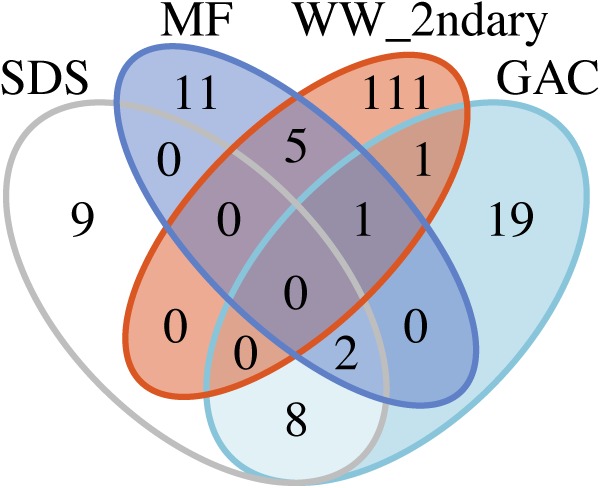
Recurring ASVs shared between different locations in the treatment train. Counts include only ASVs that appear in at least two samples from the same location with at least 50 reads. All parallel GAC samples and all SDS samples were included together as single “locations” for this analysis. Read counts and abundances of the four ASVs shared between secondary wastewater (“WW_2ndary”) and MF can be found in Supplementary Figure S7.

To examine potential persistence of bacteria from secondary wastewater and early treatment steps into post-NF/RO samples, we investigated the reoccurrence of specific ASVs, and used metagenomic read-mapping to assemblies, to unique RpsC-containing scaffolds, and to non-redundant MAGs. ASVs were defined as recurring if they were detected with >50 reads in at least two samples from a given location. The 50-read cut-off was used as minimal protection against barcode bleed (also known as sample cross-talk) from Illumina sequencing (Mitra et al., 2015; Edgar, 2016). The criterion for recurrence in two samples was used to protect against one-off cross-contamination of samples or barcodes. There was no intersection between the recurring ASVs in the secondary wastewater or MF samples and ASVs found in the SDS (all three SDS treated as one location) (Figure 5). A total of four ASVs were shared between pre-NF/RO samples (secondary wastewater and MF) and post-NF/RO samples (GAC, all three treated as one location) (Figure 5 and Supplementary Figure S7). One of these four ASVs was the Neisseriaceae sequence highly abundant in secondary wastewater. This ASV was detected but not prevalent in samples after NF/RO, having a maximum of 160 reads or 1.3% relative abundance in any post-NF/RO sample (GAC column 3, day 184). Two other recurring ASVs, classified as Methylocystaceae and Bradyrhizobiaceae were most prevalent in GAC and SDS samples and were widespread in these samples. These ASVs were also detected before NF/RO with as many as 309 reads (1.5%) and 2168 reads (8.1%), respectively, in pre-NF/RO samples. The Bradyrhizobiaceae and Neisseriaceae ASVs were also present in negative controls, but were not removed by data decontamination (see section “Materials and Methods”). Lastly, the fourth ASV shared between pre- and post-NF/RO samples was classified as Comamonadaceae, and was relatively rare in all samples. This ASV constituted less than 0.44% relative abundance in any pre-NF/RO and less than 2.4% in any post-NF/RO sample (Supplementary Figure S7).

**FIGURE 6:**
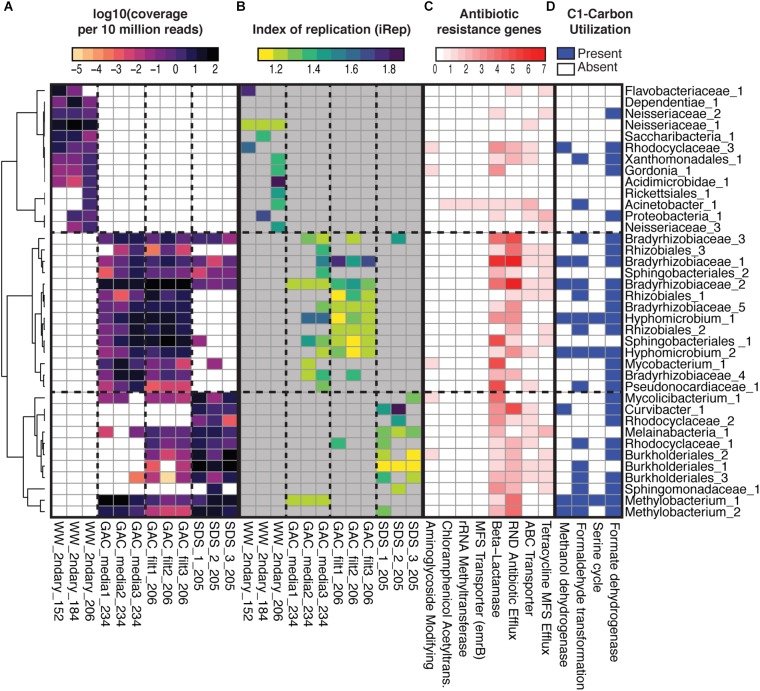
Metagenome assembled genomes (MAGs) relative abundance, indices of replication, ARG contents, and selected C1-compound utilization. **(A)** Relative abundance of 38 MAGs (row) across the 12 samples (columns) based on stringent read mapping. Cell color indicates log-10 normalized coverage per 10 million reads, and white indicates non-detection (detection threshold required at least 1× coverage across >5% of genome length). MAGs are named based on their placement in a concatenated ribosomal protein tree (Supplementary Figure S6), with numerical identifiers added for uniqueness when necessary. Hierarchical clustering of MAGs (*y*-axis) is based on a distance matrix calculated from Spearman’s rho. Sample names are based on location in treatment train, secondary wastewater (“WW_2ndary”), media from three parallel GAC columns (“GAC_media”), filtrate from three parallel GAC columns (GAC_filt), and bulk water from three parallel SDS (“SDS”). **(B)** iRep values for each MAG in each sample. Gray indicates no data where requirements for calculating iRep were not met. **(C)** Counts of ARGs by category based on ResFams. **(D)** Presence of selected pathways for C1-carbon utilization in each MAG, based on annotations of a subset of predicted proteins involved in those pathways. See Supplementary Table S5 for data.

Metagenomic analyses with stringent read-mapping also revealed little to no overlap between secondary wastewater and post-NF/RO samples (GAC media, GAC filtrate, and SDS bulk water) (Supplementary Figure S8). At most, 0.011% of metagenomic reads from any given secondary wastewater sample were stringently mapped to the assemblies of any post-NF/RO samples (=1 mismatch per read across scaffolds ≥1 kbp). Likewise, fewer than 0.10% of reads from any post-NF/RO sample mapped to a secondary wastewater assembly (Supplementary Figure S8). Based on read-mapping to scaffolds containing the marker gene RpsC, no overlap was detected between the secondary wastewater and the post-NF/RO (Supplementary Figure S5). Mapping reads to 38 non-redundant MAGs gave the same result (Figure 6A).

As a group, GAC media samples shared the majority of unique RpsC sequences and MAGs with GAC filtrates. Individually, between 42 and 59% of GAC filtrate reads could be mapped to their respective GAC media assemblies (Supplementary Figure S8). Between GAC filtrates and SDS samples, overall community structure differed; while some MAGs were shared, others were detected in only GAC filtrate or SDS (Figure 5). Between these sample points, water underwent batch chlorination, storage, and simulated distribution, and there was a 10-fold drop in median intact cell counts (Figure 2A). Thus, some of the ASVs shared between GAC filtrate and SDS may represent chlorine-inactivated cells whose DNA remained intact (Figure 3B, 6A and Supplementary Figure S5).

### Potential Pathogens and Antibiotic Resistance Genes

To investigate whether the advanced treatment facility could be a source of opportunistic drinking water pathogens, we calculated the minimum, median, and maximum possible relative and absolute abundances of *Legionella* spp. and *Mycobacterium* spp. at each location in the treatment train (Table 2). Calculations were based on amplicon sequencing (including ASVs >0.05% relative abundance in at least one sample) combined with total cell counts quantified via flow cytometry. The estimated maximum *Legionella* spp. in secondary wastewater and SDS was 4.1 × 10^5^ cells ⋅ mL^−1^ and 1.6 × 10^3^ cells ⋅ mL^−1^, respectively (Table 2 and Supplementary Figure S9). Of the seven *Legionella* ASVs included in the calculations, four were from pre-NF/RO, two from post-NF/RO, and one shared between pre- and post-NF/RO samples (Supplementary Figure S9). In the genus *Mycobacterium*, maximum possible concentrations in secondary wastewater and SDS were 1.7 × 10^6^ cells ⋅ mL^−1^ and 3.7 × 10^4^ cells ⋅ mL^−1^, respectively (Table 2). Only one *Mycobacterium* sp. ASV was detected in both pre- and post-NF/RO samples. This was the same ASV that was highly abundant in MF filtrate samples and detected at very low abundances in every other location. The *Legionella* and *Mycobacterium* ASVs found in pre-and post-NF/RO were not identified in the analysis of persistent ASVs across the treatment train (see section “Persistence of Bacteria Through Advanced Treatment”) because they didn’t meet the criteria for consideration. Thus, while they were detected, their presence in the data may be due to Illumina barcode bleed or sample cross-contamination. The reported maximum opportunistic pathogen concentrations were likely overestimated because they were calculated using maximum relative abundances and maximum cell counts ever observed at a given location across the duration of the study, regardless of whether these occurred at the same time. Furthermore, abundance calculations used total cell counts and thus also included non-viable cells (Figure 2A) and assumed a single copy of the 16S rRNA gene per cell. *Legionella* spp. and *Mycobacterium* spp. have median 16S rRNA gene copy numbers of 3 and 1, respectively^6^.

**Table 2:** Relative and absolute abundance (total cells per milliliter) estimates for genera containing opportunistic pathogens.

		WW_2ndary	Chloramine	MF	NF-RO	GAC	SDS
**Total cell counts**	Min. cells/mL	6.8E+06	2.4E+06	1.4E+03	2.1E+01	4.6E+03	7.3E+01
	Med. cells/mL	9.7E+06	7.6E+06	2.7E+03	1.2E+02	9.8E+03	5.6E+03
	Max. cells/mL	7.1E+07	5.8E+07	5.9E+03	2.8E+03	2.1E+04	1.4E+05

**Legionella^∗^**	Min. % abund	0	0	0	0	0	0
	Med. % abund	0.081	0.035	0	0.16	0.3	0.036
	Max. % abund	0.57	0.11	0	0.32	1.3	1.1
	
	Min. cells/mL	0.0E+00	0.0E+00	0.0E+00	0.0E+00	0.0E+00	0.0E+00
	Med. cells/mL	7.9E+03	2.6E+03	0.0E+00	1.9E-01	3.0E+01	2.0E+00
	Max. cells/mL	4.1E+05	6.5E+04	0.0E+00	8.9E+00	2.7E+02	1.6E+03

**Mycobacterium^∗^**	Min. % abund	0.27	0.38	68	0.029	0	0
	Med. % abund	0.88	1.9	93	0.044	0	0.074
	Max. % abund	2.3	87	97	0.06	2.4	26
	
	Min. cells/mL	1.8E+04	8.9E+03	9.5E+02	6.2E-03	0.0E+00	0.0E+00
	Med. cells/mL	8.5E+04	1.4E+05	2.5E+03	5.3E-02	0.0E+00	4.1E+00
	Max. cells/mL	1.7E+06	5.0E+07	5.7E+03	1.7E+00	5.2E+02	3.7E+04

*^∗^Includes only ASVs present at ≥0.05% relative abundance in at least one sample. Absolute abundance calculations assume 16S rRNA gene copy number of 1.*

Mycobacteriaceae were also identified in metagenomic analyses, where they could be more accurately classified. A recent phylogenomic study split the genus *Mycobacterium* into five new genera, of which one, the *Mycobacterium* spp., contains the majority of slow-growing pathogens (Gupta et al., 2018) (not reflected in SILVA v128 used for amplicon analysis). We reconstructed two unique, near-complete Mycobacteriaceae MAGs, each detected by read-mapping in at least three samples. Using Gupta et al. (2018) as a starting point, we conducted a phylogenomic analysis, placing the two MAGs within the Mycobacteriaceae. One MAG was nearest neighbor to *M. gordonae* (within the slow-growing, pathogen-containing *Tuberculosis-Simiae* clade, genus *Mycobacterium*), and the other closest to *M. smegmatis* (within the rapid-growing *Fortuitum-Vaccae* clade, genus *Mycolicibacterium*) (Supplementary Figure S10). Notably, the *M. gordonae*-related MAG was not detected by read-mapping in two of the SDS, and in the third SDS it was at low relative abundance (coverage of 0.24× per 10 million reads) (Figure 6A).

In addition to opportunistic pathogens, we searched metagenomic assemblies for protein sequences from human adenoviruses and polyomaviruses (both dsDNA enteric viruses) that could indicate passage through the treatment train. Using custom built HMMs (see Supplementary Methods), no hits were identified for any of these proteins within any metagenome, including secondary wastewater samples. This result was not surprising, given that pore size of membranes used for sample collection (0.22 μm) was much larger than these viruses and the sample concentration method was not optimized for retention of viruses.

We examined whether ARGs from secondary wastewater had passed through advanced treatment or were sourced from bacteria within the treatment train. Putative ARGs were identified in assembled metagenomic data by searching against the ResFams database (Gibson et al., 2015). Of the 123 ARG families in ResFams, only 34 were found, and these were mechanistically classified as efflux pumps, enzymes catalyzing structural modifications to antibiotics, beta-lactamases, and regulatory proteins (Figure 6C and Supplementary Figure S11). Of the 1609 protein hits across 12 metagenomes, no putative ARG sequences from secondary wastewater samples clustered with sequences in post-NF/RO samples at ≥99% amino acid identity. Putative ARGs were found at higher normalized coverages in GAC media, GAC filtrates, and SDS samples relative to wastewater, an effect primarily driven by ARG families associated with transport, and beta-lactamases of classes A and B (Supplementary Figure S11). Many ARGs co-occurred in the same MAGs (Figure 6C).

### Microbial Growth in Advanced Treatment and Distribution

Given the increases in microbial cell counts after GAC filtration (Figure 2A), we were interested in microbial growth in oligotrophic conditions after NF/RO. We estimated TOC that could have passed through a single GAC column over 205 days prior to sampling. With a maximum flow rate of 4,400 L per day through a GAC column, and the typical 50–300 μg ⋅ L^−1^ of TOC after RO in advanced treatment trains (Zeng et al., 2016), we estimated the maximum possible amount of TOC adsorbed in one column was 45–135 g. Chemical analyses have shown that aldehydes and other small carbon molecules are formed after ozonation of wastewater (Wert et al., 2007), and other studies have shown that small uncharged compounds may pass through RO membranes (Bellona et al., 2004). Other carbon sources for bacterial growth may have included carbon fixed during algal growth in the columns, and the GAC media itself.

Given these possible carbon sources, annotations of predicted proteins from the non-redundant MAGs were used to assess the capacity for C1-carbon compound utilization, including methanol and formaldehyde (Figure 6D). Seven MAGs recovered from post-NF/RO samples encoded putative methanol or methanol/ethanol dehydrogenases. Additional MAGs encoded the capacity to convert formaldehyde to formate, which could then be a substrate for energy generation via formate dehydrogenase. Lastly, incorporation of C1-carbon compounds into biomass may occur via the serine cycle, which was encoded in some MAGs (Figure 6D). It should be noted that the absence of specific pathways in MAGs does not necessarily indicate the inability of the corresponding organisms to grow on C1 compounds. These analyses were not exhaustive of all possible pathways, nor were all genomes complete.

Lastly, a proxy for bacterial growth rate, the index of replication (iRep), was calculated for each MAG in each sample. After filtering to remove MAG-by-sample combinations with low coverage, high genome fragmentation, and poorly fitted regressions (see Brown et al., 2016), iRep values were retained for 80 MAG-by-sample combinations (Figure 6B and Supplementary Table S5). Many genomes had low iReps (1.1–1.2) suggesting they were not actively replicating. The fastest replication rates (>1.5) were observed for four genomes in the secondary wastewater samples (Rhodocyclaceae_3, Flavobacteriaceae_1, Proteobacterium_1, and Acidimicrobidae_1), two genomes in GAC media (Hyphomicrobium_1 and Bradyrhizobiaceae_1), one genome in GAC filtrate (Bradyrhizobiaceae_1), and one genome in an SDS (Curvibacter_1).

## Discussion

Based on amplicon sequencing and metagenomic analyses of microbial communities in a pilot advanced treatment facility for potable reuse, we found that: (1) bulk water bacterial diversity paralleled cell counts through treatment; (2) bacterial communities were significantly different by location in treatment; (3) few or no community members were shared between secondary wastewater and SDSs; (4) absolute abundances of potential opportunistic pathogens dropped substantially during treatment and neither opportunistic pathogens nor ARGs present in treated water were likely due to passage of bacteria through advanced treatment; and (5) microbial growth occurred after NF/RO and could have been due to metabolism of small carbon compounds.

Based on cell counts by flow cytometry and amplicon sequencing, advanced treated water at the pilot facility was not completely sterile, but its microbiota was significantly different from that of secondary wastewater. Changes in microbial communities across another advanced treatment train were also recently reported via a read-based metagenomic approach (Stamps et al., 2018). The present study and work by Stamps et al. (2018) complement a large body of work documenting the ability of advanced treatment to transform and removal chemical constituents as well as specific pathogen targets (enteric viruses and protozoan cysts) (Trussell et al., 2011). Together, these studies illustrate that the chemical and microbial identity of advanced treated water intended for potable reuse is fundamentally distinct from its wastewater source.

We looked for evidence of microbial persistence through treatment and found four ASVs detected before and after NF/RO. For all four, the ASV was differentially abundant either before or after NF/RO, meaning it was primarily removed through treatment or that it survived or grew in later treatment processes while other ASVs became less abundant. Alternative explanations besides persistence through treatment include Illumina barcode bleed and sample cross-contamination. In the future, taxa identified in this manner could be quantified from the original samples using qPCR, which would provide a means to rule out barcode bleed or contamination during library preparation. In a larger-scale study, separately sequenced technical replicates could be used to address cross-contamination. Some ASVs were detected only in post-NF/RO sampling locations (Figure 5). Assuming they were not simply below the detection limit in pre-NF/RO samples or controls, these post-NF/RO sequences could represent organisms that grew in the oligotrophic, post-NF/RO environment, especially in GAC filter media. Sequences classified within the order Rhizobiales were well-represented in the GAC media and filtrate samples. Rhizobiales were previously identified in ultrapure water (ironically, in studies of sequencing contaminants) (Kulakov et al., 2002; Barton et al., 2006), and are suggested to be capable of oligotrophic metabolism (McAlister et al., 2002). The prevalence of Rhizobiales is also consistent with studies of GAC filter microbiota in laboratory-scale surface water treatment (Vignola et al., 2017) and in conventional drinking water treatment facilities (Lautenschlager et al., 2014; Oh et al., 2018).

The media samples harvested from the three different GAC columns shared community members (MAGs, RpsC sequences, and reads), likely due installation under the same environmental conditions (introduction of same GAC-colonizing organisms) and feeding with the same water. However, the media communities also exhibited differences from one another (Figure 6 and Supplementary Figures S5, S8), likely attributable to the different types of GAC and differing empty bed contact times. Based on read-mapping to assemblies, column 3, which had a greater empty bed contact time, shared less of its microbial community with columns 1 and 2 than those columns shared with each other (Supplementary Figure S8). Column 3 was also the only column with a second unique algal species.

GAC media, GAC filtrates, and SDS shared some ASVs and MAGs (Figure 5, 6A), consistent with findings from conventional drinking water systems in which “leaky” treatment filter colonizers seed filtrates (Vignola et al., 2017) and enter drinking water distribution systems (Pinto et al., 2012). The laboratory-scale batch chlorination applied to GAC filtrate in our study was likely much more aggressive than that of full-scale, continuous-flow treatment plants. This chlorination step is one likely reason for differences between GAC filtrate and SDS microbial communities, a difference that would have been more pronounced had we sequenced DNA from intact cells only (Chiao et al., 2014). SDS communities appeared to be affected by stagnation, which has been shown to change microbial communities in premise plumbing (Ling et al., 2018).

Our estimated concentration ranges of opportunistic pathogens (i.e., *Legionella* spp. and *Mycobacterium* spp.; Table 2) in GAC filtrate and SDS effluent are similar to previously reported values for simulated or full-scale drinking water systems (Wang et al., 2012, 2013; Waak et al., 2018). Wang et al. (2012) observed average concentrations of 1.4 × 10^4^ and 187 gene copies ⋅ mL^−1^ of *Mycobacterium* spp. and *Legionella* spp., respectively, in two chloraminated drinking water distribution systems. In a follow-up drinking water simulation using annular reactors, Wang et al. (2013) found *Mycobacterium* spp. and *Legionella* spp. concentrations to range from 10^3.5^ to 10^5^ and 10^2^ to 10^4.5^ gene copies ⋅ mL^−1^. In the current study, the observed increase in *Legionella* spp. through GAC filtration is unsurprising, given previous reports on drinking water filters as potential harbors of opportunistic pathogens (Wullings and van der Kooij, 2006; Vignola et al., 2017). Finally, we note that not all organisms in the genera *Legionella* and *Mycobacterium* are pathogenic, pathogenicity cannot be inferred from amplicon data, and amplification of low-biomass samples may lead to biased data (Kennedy et al., 2014). In metagenomic data, extensive phylogenetic characterization was performed for Mycobacteriaceae genomes, but we did not convert this data to a metric of absolute abundance due to the fact that all 38 MAGs accounted for only a fraction of total reads (52–55% for GAC media metagenomes).

In our study, no ARG sequences from secondary wastewater were found in post-NF/RO advanced treated water. Other studies have sought to use metagenomic sequencing and qPCR to trace ARGs from wastewater treatment into the environment or from raw water through drinking water treatment to potable water. Xi et al. (2009) observed 0.5–3 log_10_ removal of six ARGs across a conventional drinking water treatment plant, followed by a 0–1 log_10_ increase in ARG concentrations after distribution. Using metagenomics and qPCR, Garner et al. (2018) found no significant difference in ARG abundances before and after conventional drinking water treatment, but it is unclear if the exact same ARG sequences were present before and after and thus uncertain whether these ARGs passed through treatment. In another study, multiple ARGs and mobile genetic elements increased in relative abundance through chlorination, but subsequently decreased in relative abundance after pipeline distribution (Shi et al., 2013). Especially in studies of advanced treatment trains, deeper sequencing of the highly diverse secondary wastewater could capture more of the upstream microbial community and increase the likelihood of detecting any ARGs that reappear downstream. It should be noted that even when putative ARGs are predicted in sequence-based analyses, experimental validation is required to determine whether these genes confer antibiotic resistance. Here, the majority of ARGs identified in post-NF/RO metagenomes encode beta-lactamases and transporters, which could have biological functions besides or in addition to antibiotic resistance (e.g., catabolism of beta-lactamases) (Crofts et al., 2018).

A challenge of this study and of Stamps et al. (2018) was the low-biomass in samples taken after NF/RO. Prior to data decontamination, many of the low-biomass amplicon samples appeared similar to the negative controls (not shown), and several ASVs were shared between samples and controls. Although flow cytometry results for NF/RO permeate indicated cell counts above the limit of detection (Figure 2A), only two samples from this location were successfully amplified and decontaminated. As the presence of reagent contaminants in amplicon data is directly dependent on input DNA concentration (Salter et al., 2014; de Goffau et al., 2018), filtration of larger sample volumes, as well as sequencing of more negative controls, may allow future studies to more comprehensively and conclusively profile microorganisms present in advanced treated water. Additionally, recovery of longer 16S rRNA gene sequences could provide greater resolution to distinguish between contaminants from reagents made with molecular-grade water and true sequences present in advanced treated water, which may be very similar but not identical. We did not perform metagenomic sequencing of negative controls due to low DNA concentrations in field and extraction blanks, but possible contaminants of metagenomes were several MAGs classified as *Methylobacterium*, *Bradyrhizobium*, and *Melainabacteria*. These MAGs were present at high coverages across all low-biomass samples (GAC media, GAC filtrate, and SDS) (Figure 6A), and similar patterns for the same taxa were observed in amplicon sequencing data prior to decontamination.

We used multiple high-throughput sequencing and bioinformatic approaches for studying advanced treatment, which converged on similar results, and provided different types of information. Amplicon sequencing yielded greater depth than metagenomics, allowing detection of rarer bacterial populations. To complement this approach, assembly based metagenomics provided more accurate phylogenetic identification of abundant populations (Supplementary Figures S6, S10), additional confidence about the presence/absence of specific populations represented by MAGs in a given sample, as well as predicted metabolisms and replication rates (Figure 6). Amplicon and metagenomic sequencing also had different biases as observed in multiple analyses of the same mock community (Supplementary Figure S1). Read-based metagenomics often disagreed with both amplicon and assembly based metagenomic data in terms of taxonomy (Supplementary Figure S4), which was not surprising, as it relied upon a fixed database. A strength of read-based analysis is that it includes all data, even reads from genomes that may not assemble well. However, read-mapping against a database using short reads cannot directly determine whether the exact same gene or genome sequence is present in multiple samples (such as across advanced treatment), and read mapping without *de novo* assembly provides limited information about novel genomes and genes from the environment. The utility of MAGs to address questions of microbial transfer across environments and into patients has been previously demonstrated (Brooks et al., 2017; Olm et al., 2019), and we predict that this high-resolution approach will be most useful in future studies of advanced water treatment, provided that higher-biomass sampling and deep sequencing can be achieved.

There are practical implications to the finding that new microbial growth occurs after advanced treatment (Park and Hu, 2010) and is not completely mitigated by disinfection or maintaining a disinfectant residual in the distribution network (Nescerecka et al., 2014). Such growth, including opportunistic pathogens and ARG-containing organisms, might be expected to occur where biofilms attach or nutrients accumulate (such as in GAC filters, chemical conditioning processes, decarbonation towers, or storage tanks). Thus, advanced treatment facility design should also consider the types of processes (and associated microorganisms) intentionally placed after NF/RO and AOP in treatment trains. Ultimately, a more comprehensive understanding of chemical and microbial water quality in advanced treatment will also aid in effective management of direct potable reuse distribution systems, considering corrosion, disinfectant by-products, and opportunistic pathogens.

## Data Availability Statement

The datasets generated for this study can be found in the National Center for Biotechnology Information (https://www.ncbi.nlm.nih.gov/) under Bioproject PRJNA490743 with Sample IDs SAMN10056964–SAMN10057130.

## Author Contributions

SM and KN designed the study. SM performed the fieldwork. RK performed all bioinformatic analyses and wrote the manuscript with contributions from KN and SM. SM and RK jointly completed DNA extractions and amplicon library preparation.

## Conflict of Interest Statement

The authors declare that the research was conducted in the absence of any commercial or financial relationships that could be construed as a potential conflict of interest.

## Footnotes

1 https://github.com/SchlossLab/MiSeq_WetLab_SOP/blob/master/MiSeq_WetLab_SOP.md

2 https://www.bioinformatics.babraham.ac.uk/projects/fastqc/

3 https://sourceforge.net/projects/bbmap/

4 https://github.com/christophertbrown/fix_assembly_errors

5 https://github.com/christophertbrown/iRep

6 https://rrndb.umms.med.umich.edu/ (accessed August 14, 2018)

## Abbreviations

AOP: advanced oxidation process
ARG: antibiotic resistance gene
ASV: amplicon sequence variant
GAC: granular activated carbon
MAG: metagenome assembled genome
MF: microfiltration
NF: nanofiltration
RO: reverse osmosis
RpsC: ribosomal protein S3
SDS: simulated distribution system(s)
TOC: total organic carbon.
