# Erratum: The Water Microbiome Through a Pilot Scale Advanced Treatment Facility for Direct Potable Reuse

**DOI:** 10.3389/fmicb.2019.02636

**Published:** 2019-11-11

**Authors:** Frontiers Production Office

**Affiliations:** Frontiers Media SA, Lausanne, Switzerland

**Keywords:** direct potable reuse, metagenomics, 16S rRNA gene sequencing, drinking water microbiome, advanced water treatment, antibiotic resistance

Due to an editorial error, the Data Availability Statement containing the accession numbers were erroneously omitted from the article. The Data availability Statement appears below. The publisher apologizes for the mistake. The original article has been updated.

## Data Availability Statement

The datasets generated for this study can be found in the National Center for Biotechnology Information (https://www.ncbi.nlm.nih.gov/) under Bioproject PRJNA490743 with Sample IDs SAMN10056964–SAMN10057130.

