# Supplementary material for: The Water Microbiome Through a Pilot Scale Advanced Treatment Facility for Direct Potable Reuse
Source: Front Microbiol. 2019 May 8;10:993. doi: 10.3389/fmicb.2019.00993 (PMC6517601; doi:10.3389/fmicb.2019.00993)
Supplement: Supplementary file 1 [file Data_Sheet_1.PDF]

The water microbiome through a pilot scale advanced treatment facility for direct potable reuse

Rose S. Kantor[1,2], Scott E. Miller[1,2], Kara L. Nelson[1,2]*

[1] University of California, Berkeley, Department of Civil and Environmental Engineering, Berkeley, CA, USA
[2] Engineering Research Center for Re-inventing the Nation's Urban Water Infrastructure (ReNUWIt), Berkeley, CA 94720 United States
*** Correspondence:**
Kara Nelson
karanelson@berkeley.edu

Keywords: Direct potable reuse (DPR), metagenomics, 16S rRNA gene sequencing, drinking water microbiome, advanced water treatment, antibiotic resistance

Supplementary Methods

GAC column operation

Three granular activated carbon (GAC) filters were established one week before full operation of the advanced treatment facility. The filter media was catalytic re-agglomerated bituminous GAC (column 1; Calgon Centaur(R) HSL 8x30), catalytic coconut-shell derived GAC (column 2; Evoqua AquaCarb(R) 830), and re-agglomerated bituminous GAC (column 3; Calgon Filtrasorb(R) 300), respectively. Columns 1 and 2 were manufactured with proprietary "catalytic" properties that purportedly increase rates of reaction with oxidants like hydrogen peroxide by increasing the number of sites available for catalysis. GAC filters were constructed identically (except for media type) at the pilot site in unsterile conditions. GAC media was installed as six-foot deep media medis loaded into 4-inch diameter clear PVC pipes that were exposed to ambient light.

Columns 1 and 2 were operated in catalytic mode at 0.8 gpm, corresponding to a surface loading rate of 9.2 gpm per square foot and an empty bed contact time of 5 minutes. Column 3 was operated in adsorption mode at a flow rate of 0.25 gpm, corresponding to a surface loading rate of 2.9 gpm per square foot and an empty bed contact time of 15 minutes. Column 3 was operated in adsorption mode to assess potential benefits of additional total organic carbon removal. The filter columns were backwashed with stored GAC filtrate approximately every 2-4 weeks after appreciable increases in head loss.

Simulated distribution system operation

GAC filtrate was collected every two days in 2 L Pyrex® bottles. Bottles were carbon-cleaned prior to first use as described previously (Hammes and Egli, 2007), and were autoclaved immediately after each use. GAC filtrate was chlorinated and stored in the refrigerator for a maximum of four days before replacing old GAC filtrate in the SDS reservoirs. To meet the intended chlorine residual (~1 mg/L) the applied chlorine dose varied from 1.5 to 7 mg/L due to large fluctuations in chlorine demand. Magnetic stir plates ensured full and rapid mixing of

chlorine in the chlorination reservoir. Chlorine measurements were taken using a DR/850 Portable Colorimeter (Hach USA, Loveland, CO), and DPD 10 mL Total Chlorine (#97009-464; VWR International, Radnor, PA) and DPD 10 mL Free Chlorine (#97009-454; VWR International) Reagent Powder Pillows. Chlorinated water was then transferred to a storage reservoir and served as feed for an annular reactor (AR; Biosurface Technologies Corporation, Bozeman, MT).

The three ARs were operated at the Robertson-Umbenhauer Water Treatment Plant in El Paso, TX. Each annular reactor was fed with a different chlorinated GAC filtrate from the pilot facility. The GAC filters were operated continuously for approximately five months prior to AR setup.

Flow cytometry

Total and intact bacterial cell concentrations (TCC and ICC, respectively) were measured by flow cytometry in triplicate using slight modifications from a previously optimized protocol (Prest et al. 2013). Briefly, water samples (1,000 µL or 1,500 µL) were preheated to 35 ^{o}C (10 minutes), stained with a working solution of fluorescent dye(s), and then incubated in the dark at 35 ^{o}C (10 minutes) before measurement on a flow cytometer. To assess TCC and ICC, samples were stained at 10 µL mL^{-1} with working solutions of SYBR Green I (10,000x in DMSO, S9430; Sigma-Aldrich, St. Louis, MO) that was 100x diluted in buffer (10 mM TRIS in 0.1 µm filtered nanopure water); dye solutions for ICC additionally included propidium iodide (30 mM. P1304MP; Life Technologies, Carlsbad, CA) at a working PI concentration of 0.6 mM. Where necessary, samples were diluted before preheating in 0.1 µm filtered (Millex-VV Syringe Filter Unit; Millipore, Billerica, MA) bottled mineral water (Evian, France) to achieve final bacterial cell concentrations less than 2 x 10^5 cells mL^{-1}.

Measurements were performed on an Accuri C6 flow cytometer (BD Biosciences, San Jose, CA) equipped with a 50 mW laser emitting a fixed wavelength of 488 nm. To limit background noise, the machine was cleaned prior to use each day by running diluted bleach through the sample port followed by 0.1 µm filtered ultrapure water. The flow cytometer was equipped with volumetric counting software, calibrated to measure the number of fluorescent particles in a user-defined fluid volume. Run volumes for samples were 50 uL, except for RO and NF permeates (1,000 µL) and GAC filtrate (100 µL). Measurements were performed at the "fast" flow rate of 66 µL $minute^{-1}$. Bacterial signals were distinguished and enumerated from background and instrument noise on density plots of green (FL1; 533 ± 30 nm) and red (FL3; >670 nm) fluorescence using an electronic gating system provided by the Accuri C6 software. Gate positions were modified slightly from a template publically available for the BD Accuri C6 (Gatza et al., 2013).

Limits of quantification (LOQ) for TCC and ICC were determined for NF and RO membrane permeates by staining stepwise dilutions of bottled Evian mineral water in sample blank water (0.1 µm-filtered Evian water; data not shown) as well as repeated (11x) measurements of stained blanks run with 1,000 uL of sample. LOQ was calculated as the blank average + 3x the standard deviation of the blanks, yielding LOQs for TCC (12 cells mL^{-1}) and ICC (22 cells mL^{-1}).

Amplicon sequencing: Library pooling, mock community and contaminant analysis

For the majority of samples, library preparation consisted of triplicate 25 µl reactions that were combined and concentrated by SpeedVac prior to normalization by SequalPrep. However,

samples with average PCR amplicon concentration <10 ng μl^{-1} (Qubit HS; ThermoFisher) were assumed to have failed, because negative control reactions yielded ~5-10 ng μl^{-1}. To achieve >10 ng μl^{-1} of amplicon product, any failed samples underwent a second round of PCR amplification with triplicate 25 µl reactions, and then a third and final round of PCR amplification with quintuplicate 25 µl reactions. A sample was considered successfully amplified in the second and third amplification rounds if any one well yielded >10 ng μl^{-1} of PCR product. All wells with >10 ng μl^{-1} were pooled. Several samples ultimately failed to have any well yield >10 ng μl^{-1}.

Duplicate samples of a mock community (Zymobiomics DNA mock community with 8 bacterial and 2 eukaryotic members) yielded 18 and 15 ASVs, respectively, of which 8 and 9 were identical matches to the expected reference sequences. The matching sequence that differed between the two replicates was the *Cryptococcus neoformans* 18S rRNA gene, which was present in the mock DNA pool, but amplified poorly with bacterial primers, resulting in zero reads in Mock1 and three reads in Mock2. The other eukaryotic member of the mock community, *Saccharomyces cerevisiae*, was not detected in either mock control. An additional *Salmonella enterica* sequence accounted for ~3% of all reads in each mock control sample. This sequence had a single nucleotide mismatch from the reference sequence, which was present at 16-18% of reads per sample. There were two contaminant sequences present in both Mock controls with $\geq$ 15 reads corresponding to each sequence. These sequences, classified as *Methylobacterium* spp. and *Mycobacterium* spp., were widespread throughout the experimental samples and negative controls, and were removed from all data during DESeq2-based data decontamination. Relative abundances of the mock community members did not exactly match the expected relative abundances (**Figure S1A**), likely due to primer bias, although this was not as extreme as a recent observation of the same community with different primers (Nearing et al., 2018).

Metagenomic library preparation

Twelve samples (three secondary wastewater, three GAC filtrate, three GAC media, and three SDS bulk water) and one positive control were submitted for metagenomic sequencing at the Functional Genomics Laboratory at UC Berkeley. Samples of secondary wastewater and GAC media were submitted with >300 ng and >800 ng of total extracted DNA, respectively. Total DNA extract for the GAC filtrate and SDS bulk water samples ranged from 10 – 121 ng. DNA extract for GAC media was obtained via pooling multiple DNA extractions of the filter media (five extractions for GAC 1 and 2, and two for GAC 3).

Metagenomic read processing, assembly, and read-based analyses

Metagenomic read processing used FastQC (Babraham Bioinformatics, https://www.bioinformatics.babraham.ac.uk/projects/fastqc/) to inspect quality, bbmap (Bushnell, https://sourceforge.net/projects/bbmap/) to remove PhiX and adapter sequences, and sickle (Joshi et al., https://github.com/najoshi/sickle) for quality trimming. Subsequent inspection with FastQC found that samples 1, 9, and 66 required further trimming of either forward or reverse reads to remove regions with overrepresented kmers. For samples 1 and 9, this extensive trimming (an additional bbmap step with parameters forcetrimleft=50, qtrim=20) resulted in better assemblies, but greater non-specific mapping due to some shorter reads (see below).

Read-based phylogenetic characterization was performed for each sample with MetaPhlAn2 using default parameters (Truong et al., 2015). Assembly was performed

independently for each sample using idba_ud (Peng et al., 2012) with the "--pre_correction" option. To estimate the amount of data shared between samples, reads from each sample were mapped to each assembly (scaffolds ≥ 1 kbp) using Bowtie2 (Langmead and Salzberg, 2012) with the "--reorder" option. The resulting sam files were filtered stringently to include only reads with ≤ 1 mismatch to the consensus using mapped.py (https://github.com/christophertbrown/bioscripts/blob/master/ctbBio/mapped.py), and converted to bam using samtools (Li et al., 2009) with the parameter "-F 4", to remove unmapped paired reads. For this analysis, reads from samples 1 and 9 were trimmed with bbmap flag forcetrimleft=50, and as with all other samples, sickle was used to restrict read length to ≥ 75 nt.

Metagenome annotation and gene-based analyses

All assemblies (scaffolds ≥ 1 kbp) were annotated with Prokka v1.12 (Seemann, 2014) and RNAmmer (Lagesen et al., 2007). Ribosomal protein S3 (RPS3) sequences were identified by annotation and RPS3 proteins ≥ 147 amino acids (to exclude short sequences) were clustered at 99% identity using USEARCH -cluster_fast (Edgar, 2010) (query and target coverages = 0.5). Scaffolds containing the centroids of these clusters were used as representatives of unique community members. Bowtie2 was used to map all reads to these scaffolds, and mappings were filtered stringently to include only reads with ≤ 1 mismatch to the consensus using mapped.py and converted to bam using samtools (Li et al., 2009) with the parameter "-F 4". Filtered mappings were imported into Anvi'o to calculate detection and coverage. Smoothed Q2Q3 coverage was reported for each RPS3-containing scaffold in each sample. Detection (≥ 1x coverage) across 75 % of each scaffold was used as a threshold above which coverage was reported. RPS3 protein sequences were identified by best BLAST hit against the NCBI-nr database (accessed May 17, 2018).

Antibiotic resistance genes were detected via HMMsearch of the predicted proteins against the ResFams core database of hidden Markov models (HMMs) {Gibson:2014dd} using gathering thresholds (parameter "--cut_ga"). Within each resistance gene family, ResFam hits were sorted by decreasing length and clustered at 99% identity using USEARCH -cluster_fast (Edgar, 2010). Tabulated results were searched for any clusters containing proteins from both secondary wastewater and post-NF/RO samples. We built additional custom HMMs based on MUSCLE alignments (Edgar, 2004) of proteins downloaded from the NCBI Identical Protein Groups database (accessed August 22, 2018). Proteins included full-length Mip (genus Legionella only), Adenovirus hexon protein, and JC Polyomavirus proteins (vp1, vp2, vp3, agnoprotein, large T antigen, small T antigen). Trusted cut-offs were defined as the lowest score from any protein used in the seed alignment.

Metagenome binning and non-redundant genomes analyses

In preparation for binning in Anvi'o (Eren et al., 2015), reads from each sample were again mapped pairwise to each assembly (scaffolds ≥ 2.5 kbp) using Bowtie2 (Langmead and Salzberg, 2012) with default parameters, and resulting sam files were converted to sorted, indexed bam files using samtools (Li et al., 2009). For each sample, an Anvi'o contig database was generated and a profile database was created that included mappings of every sample to the assembly of that sample. Anvi'o databases were originally created with Anvi'o v3, but later upgraded to v4. Taxonomy was added using Centrifuge (v1.0.2-beta). Binning was performed manually based on hierarchical clustering by sequence composition and differential coverage in Anvi'o. Some genome bins contained mobile elements that clearly associated with the genome

and the coverages for elements were exact multiples of genome coverage across all samples. In these cases, the mobile elements were included in the genome bin. Bins were reviewed for completeness based on single copy marker genes and bins ≥ 45 % complete (according to single copy domains from Campbell *et al.* (Campbell et al., 2013)) were exported for further completeness estimation with CheckM (Parks et al., 2015). Bins > 70% complete with < 10% contamination according to CheckM were de-replicated to generate a non-redundant set of 38 metagenome assembled genomes (MAGs) using dRep (Olm et al., 2017) with primary clustering at 95% ANI and secondary clustering at 99% ANI, requiring 60% coverage of the larger genome in each pairwise comparison.

To determine coverages of each non-redundant MAG in each sample, reads were mapped using Bowtie2 with default settings, and read-mappings were filtered stringently to include only reads with ≤ 1 mismatch to the consensus using mapped.py (https://github.com/christophertbrown/bioscripts/blob/master/ctbBio/mapped.py). These mappings were also used to calculate indices of replication (iRep values) (Brown et al., 2016) for each MAG in each sample using iRep (https://github.com/christophertbrown/iRep) with default settings. De-replicated MAGs and filtered read-mapping files were imported to Anvi'o. The Anvi'o Q2Q3 smoothed genome coverages were used to calculate relative abundances of MAGs in samples, and the Anvi'o detection parameter (percent of genome with 1x coverage) was used as a threshold to ensure coverages were only reported where MAGs had at least 1x coverage across 5 % of their length. Heatmaps of metagenomic data were created using the pheatmap and ggplot2 packages in R.

Phylogenetic placement of MAGs

To identify genomes in metagenomes, a concatenated gene tree was constructed using 16 conserved single-copy marker genes from metagenomes and reference genomes (ribosomal proteins L2, L3, L4, L5, L6, L14, L15, L16, L18, L22, L24, S10, S17, S19, S3, and S8) (Hug et al., 2016). To avoid forming chimeras through concatenation, metagenomic sequences were included in the tree only where proteins co-occurred on the same scaffold. Alignments of each protein set were conducted with MUSCLE (Edgar, 2004) and edited in Geneious. The N- and C-termini were trimmed and columns with ≥ 99% gaps were stripped. The 16 alignments were concatenated to produce a final alignment containing 2986 columns, within which the shortest and longest sequences were 1212 and 2501 un-gapped amino acids, respectively. FastTree (v2) with default parameters was used to create an approximately-maximum likelihood phylogenetic tree, and FigTree was used to visualize the tree.

Supplementary Figures

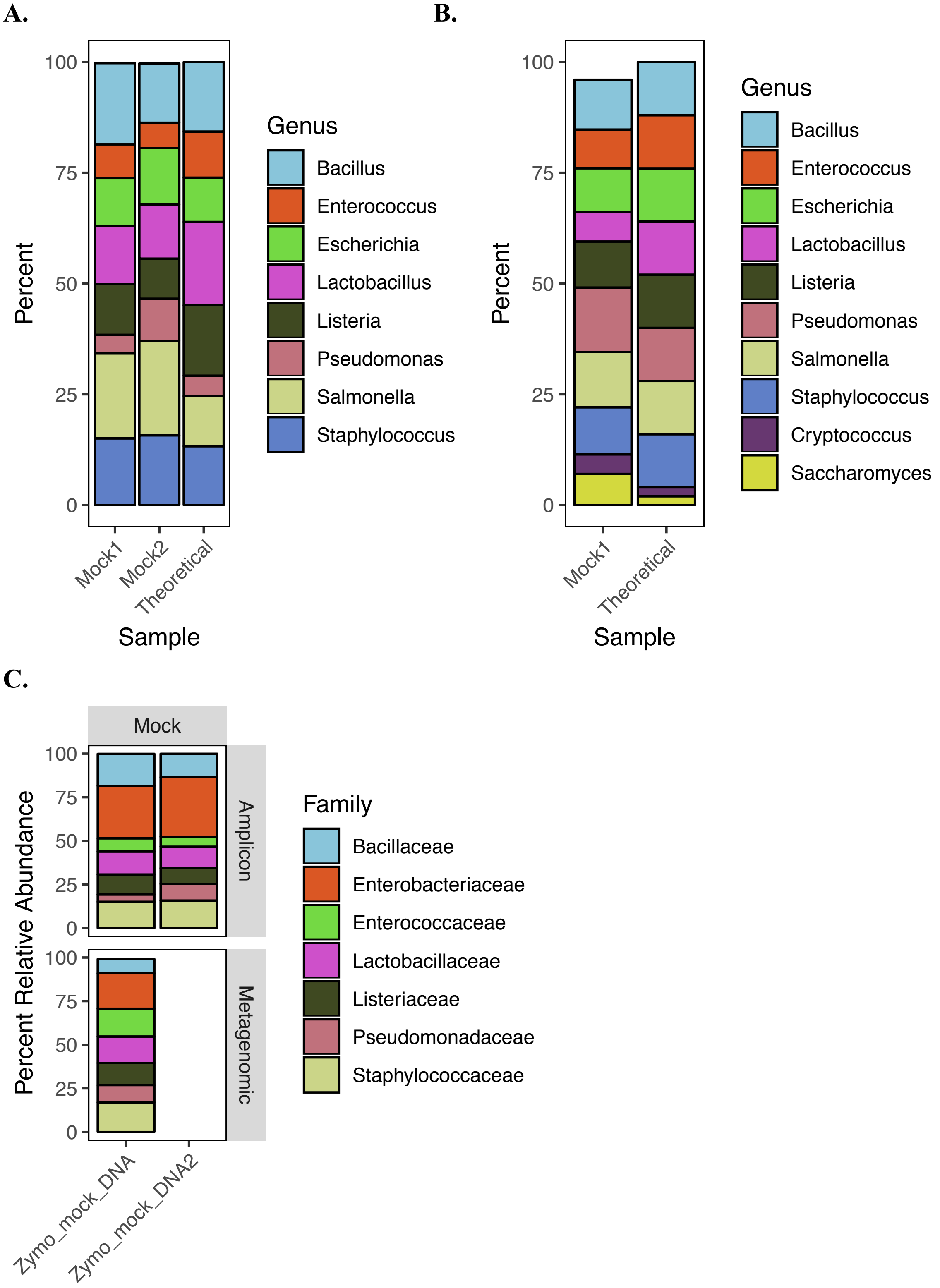

Figure S1. Comparison of actual and theoretical percent relative abundances for the Zymobiomics DNA standard mock community (Zymo Research). **(A)** Amplicon sequencing for

duplicate samples ("Mock1" and "Mock2") showed moderate amplification bias with bacteria-specific primers, as compared to the theoretical relative abundances in which 16S rRNA gene copy number has already been accounted for. **(B)** Relative abundances of metagenome assembled genomes (MAGs) ("Mock1" determined by read-mapping) differed somewhat from theoretical abundances, in part due to incomplete reconstruction of genomes. Genomes were reconstructed from scaffolds > 2.5 kbp, and accounted for 96% of total reads (**Table S3**). **(C)** Comparison between amplicon sequencing and unassembled metagenomic read-based classification with MetaPhlAn2 at the family level.

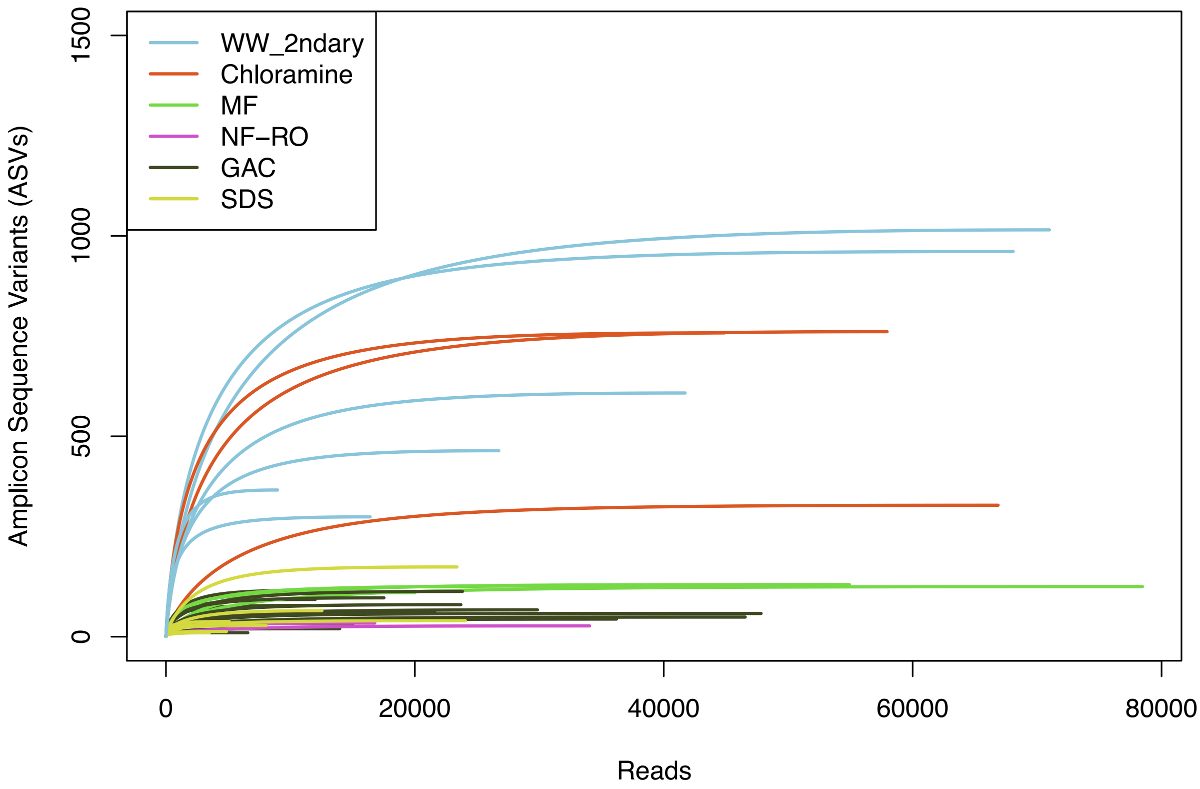

Figure S2. Rarefaction curves for all bulk water amplicon sequencing samples. Curves were made using DADA2-corrected reads (no singletons) after data decontamination. Colors indicate sample locations.

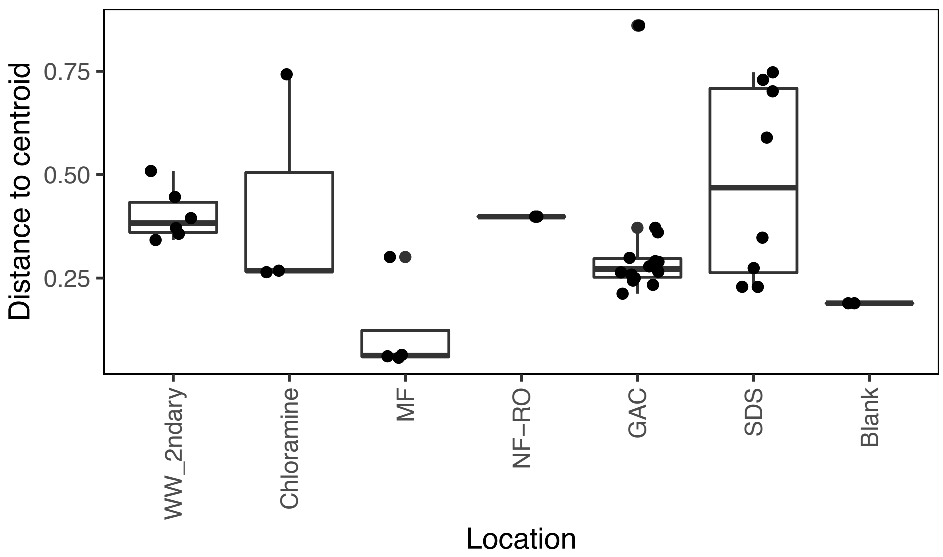

Figure S3. Group dispersions are not homogeneous across locations. Chloramine and SDS have the largest within-group dispersions, while NF/RO and negative controls ("Blank") do not have enough samples to determine dispersions. A permutation test for homogeneity of multivariate dispersions (permutest, vegan package in R) revealed somewhat significant differences by location ($p = 0.053$).

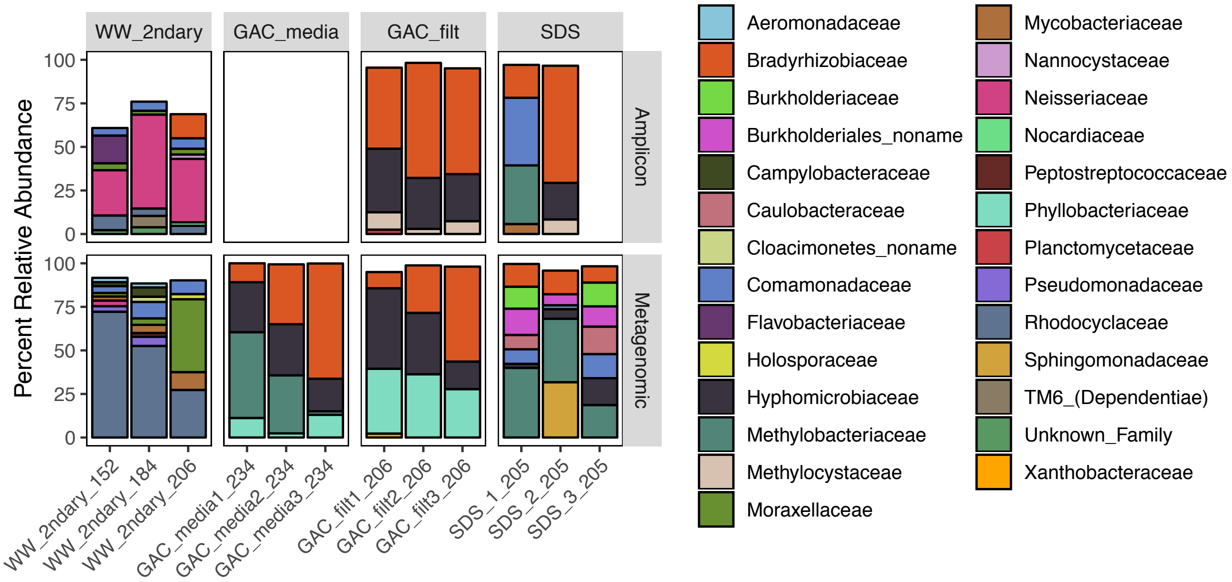

Figure S4. Comparison of microbial communities at the Family level, using amplicon and metagenomic sequencing. Amplicon data were classified with DADA2 using RDP classifier trained on SILVA v128, while metagenomic data were classified by MetaPhlAn2. Amplicon and metagenomic data are shown for Families with relative abundances ≥ 2%. For the SDS, one of the three samples with both metagenomics and amplicon sequencing did not pass amplicon QC. For amplicon data, if Family classification was “NA”, Order or Phylum is shown.

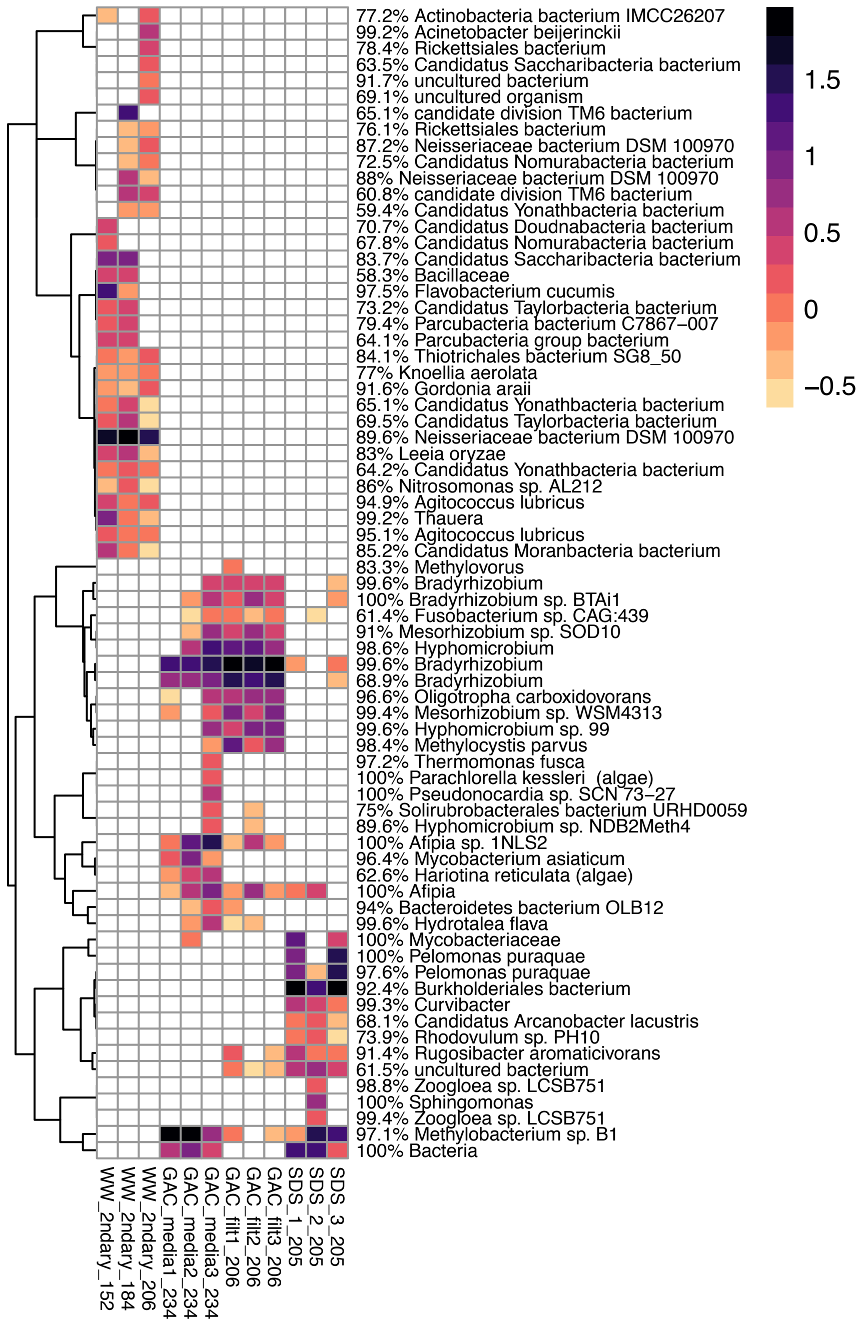

Figure S5. Heatmap of normalized relative abundances for 71 scaffolds containing unique ribosomal protein S3 (RPS3; rows) across the 12 samples (columns) based on stringent read mapping. Cell color indicates log-10 normalized coverage per 10 million reads. White indicates non-detection, and at a detection threshold requiring coverage > 1x across > 75% of scaffold length. Unique RPS3 sequences are identified by the identity to and taxonomy of the best BLAST hit to the RPS3 in NCBI-nr (accessed 5/17/18). All RPS3-containing scaffolds ≥ 1 kbp are included. Clustering of scaffolds is based on a distance matrix calculated from Spearman's rho.

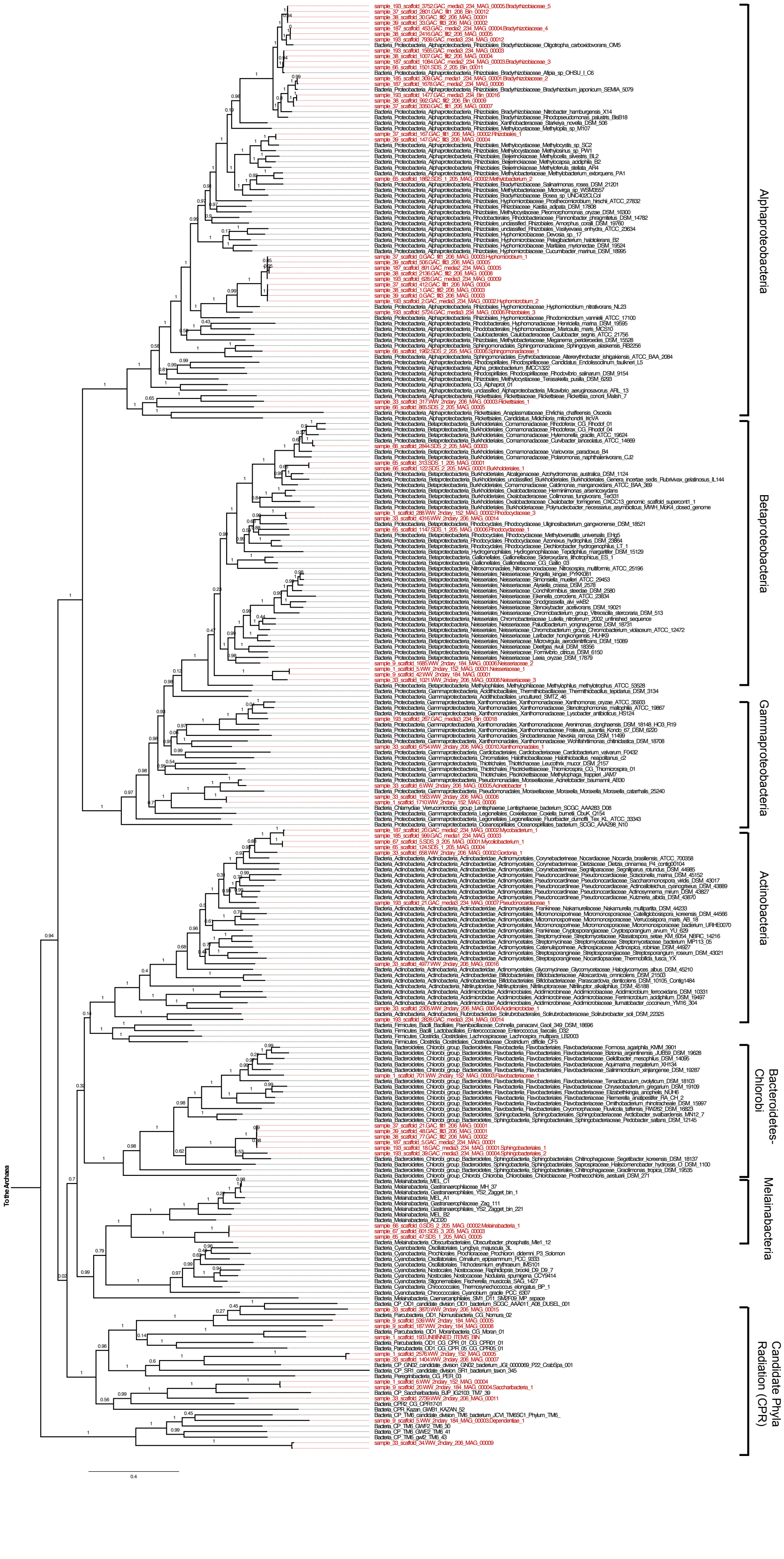
Alphaproteobacteria
Betaproteobacteria
Gammaproteobacteria
Actinobacteria
Bacteroidetes-Chlorobi
Melainabacteria
Candidate Phyla Radiation (CPR)
To the Archaea
0.4

Figure S6. Phylogenetic tree showing genomes from metagenomes placed within the domain Bacteria. The tree was constructed with FastTree (v2) based on the concatenated alignments of 16 ribosomal proteins. Sequences from all 12 metagenomic assemblies (red) and from reference genomes (black) were included, and the tree was rooted with 8 Archaeal references. Metagenomic scaffold and bin name are included in the tip names and more information about each bin can be found in **Table S4**. The scale bar represents 0.4 amino acid substitutions per site. Zoom for greater detail.

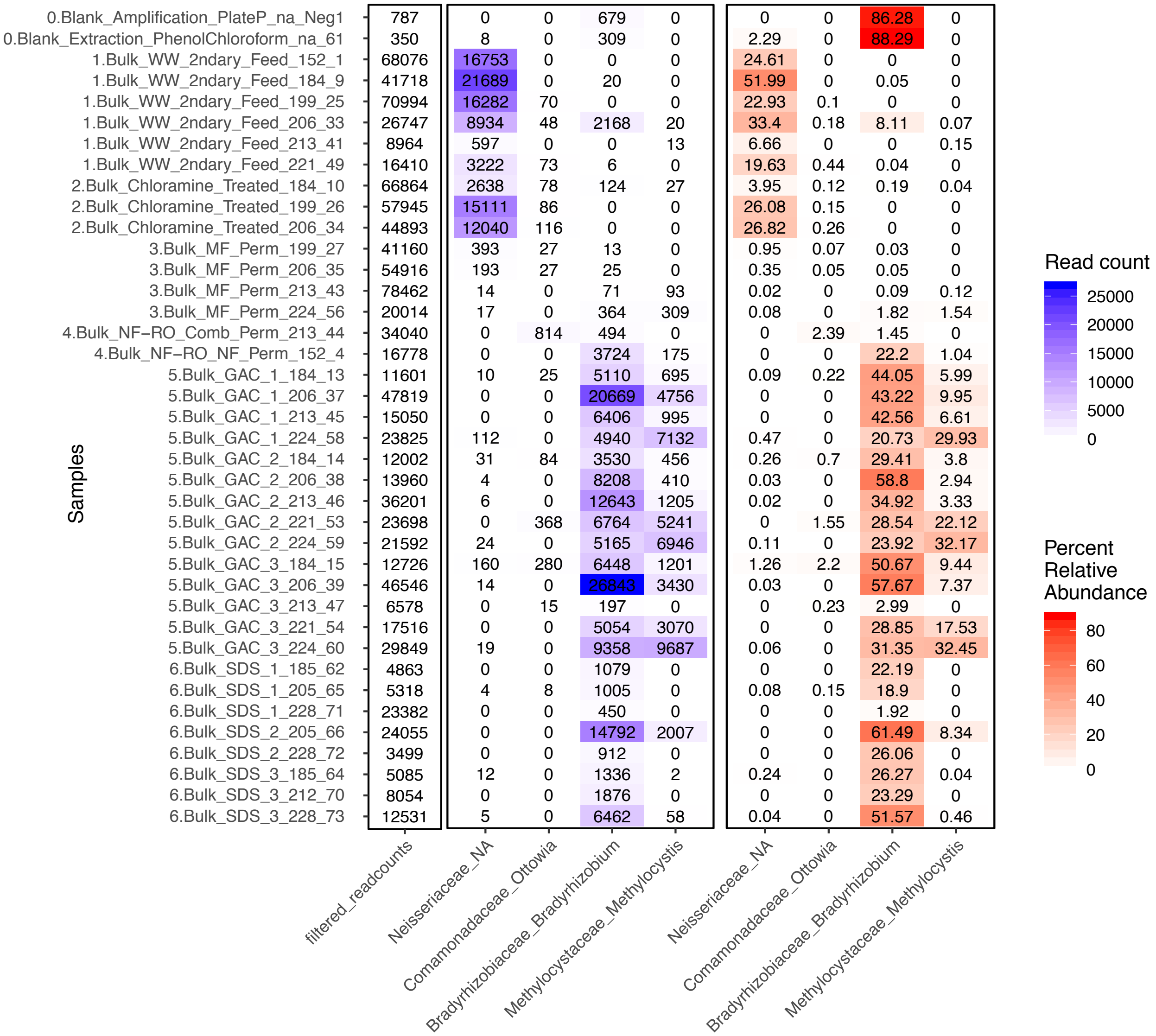

Figure S7. Heatmaps of read counts and relative abundances for four ASVs shared between pre-NF/RO samples (secondary wastewater or MF permeate) and post-NF/RO samples (GAC only). Left: total read counts for each sample after data decontamination (“filtered_readcounts”); middle: read counts for each of the four ASVs in each sample (blue); right: percent relative abundance of each ASV (red).

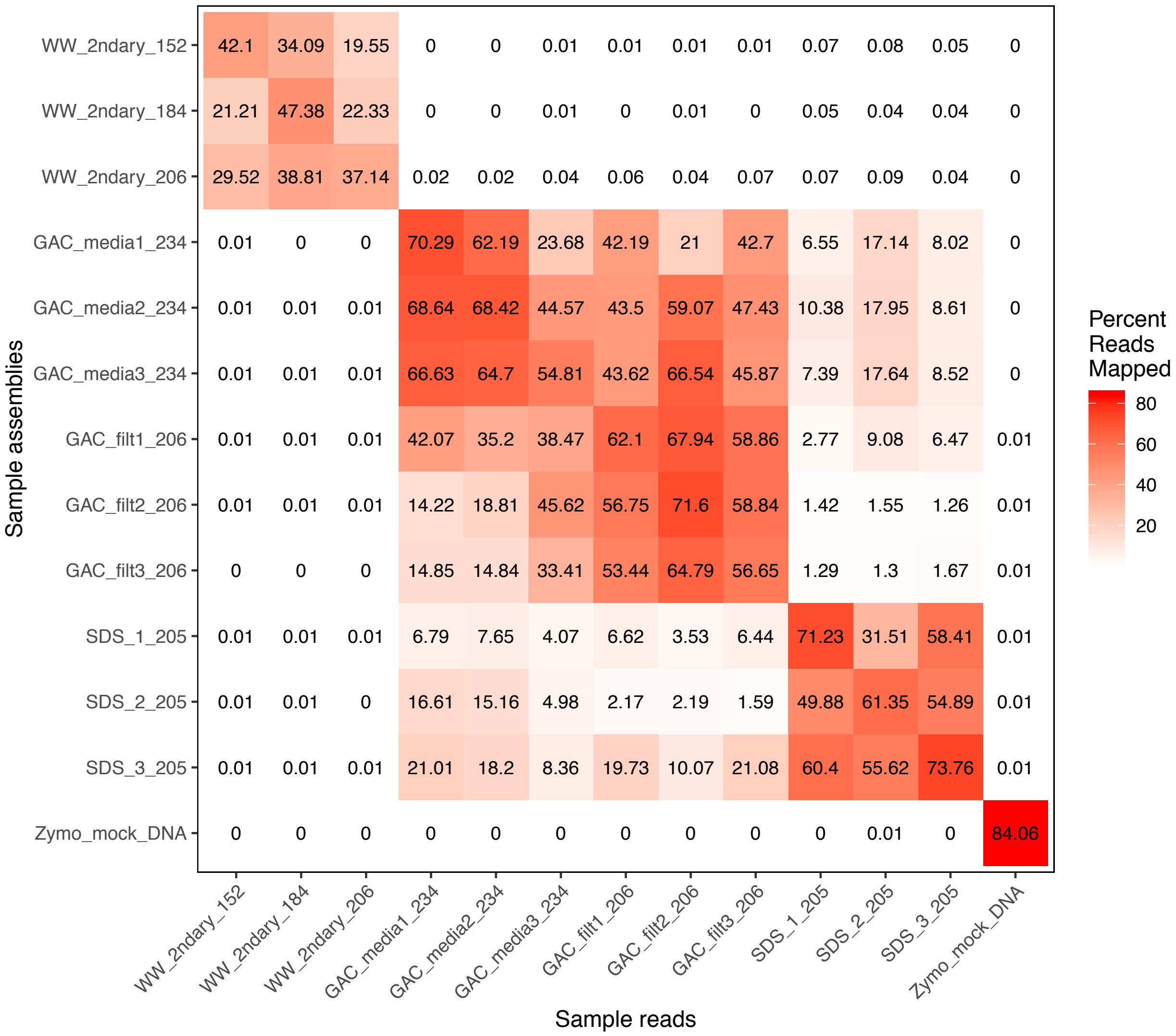

Figure S8. Heatmap showing the results of stringent read-mapping of all reads to all metagenomic assemblies (scaffolds > 1 kbp), allowing only 1 nucleotide mismatch per read. Color indicates the percent of total trimmed reads mapped from low (white) to high (red) on a linear scale.

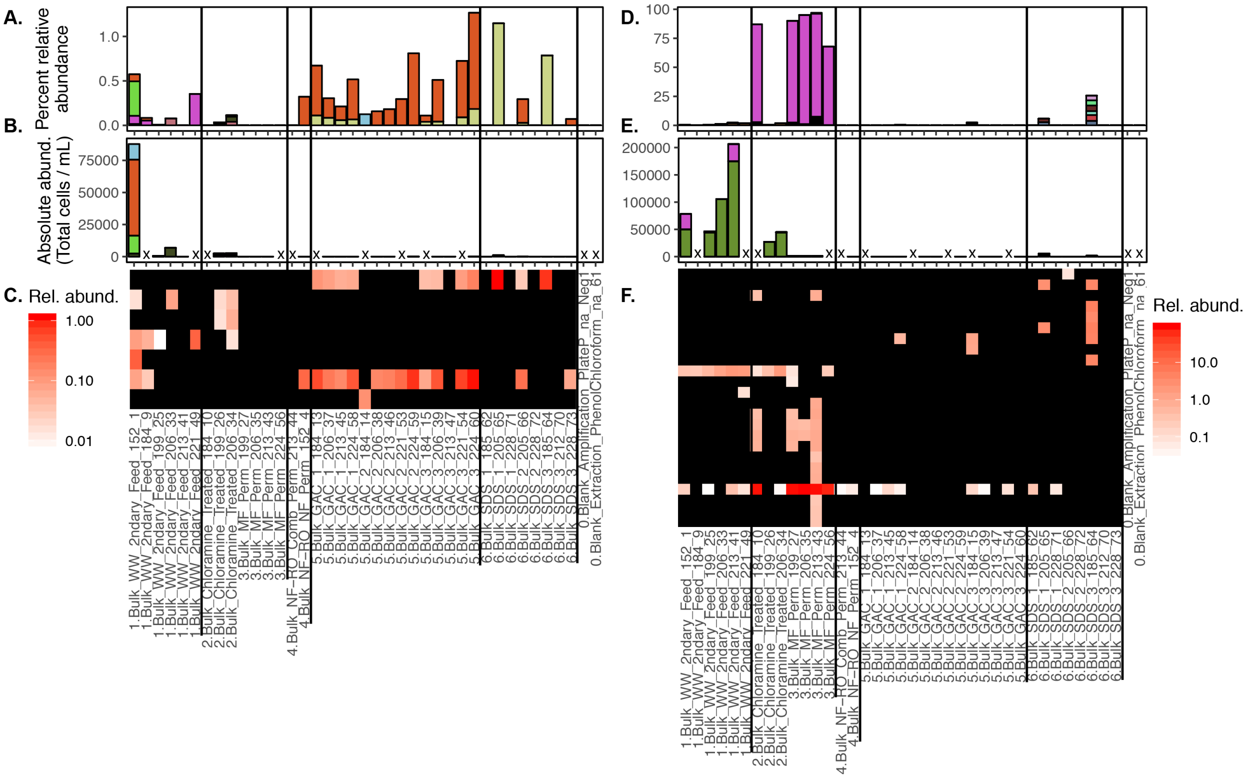

Figure S9. Relative and absolute abundances of genera containing opportunistic pathogens in amplicon data. **(A-C)** Abundances of Legionella spp. ASVs and **(D-F)** abundances of Mycobacterium spp. ASVs (note different y-axes). Within each plot, bar segments are colored by unique ASV sequence. Percent relative abundances **(A and C)** were multiplied by total cell counts from paired samples taken within 3 days of DNA samples to compute absolute abundances **(B and E)**. An “x” indicates no cell count data corresponding to the sampling date. Heatmaps **(C and F)** show that some ASVs occurred in samples from across the treatment train, but at very low percent relative abundances either before or after NF/RO (note log scale). *Legionella* and *Mycobacterium* sequences were absent from negative controls (far left). Analysis considered only ASVs within the genera of interest and present at relative abundance ≥ 0.05% in at least one sample.

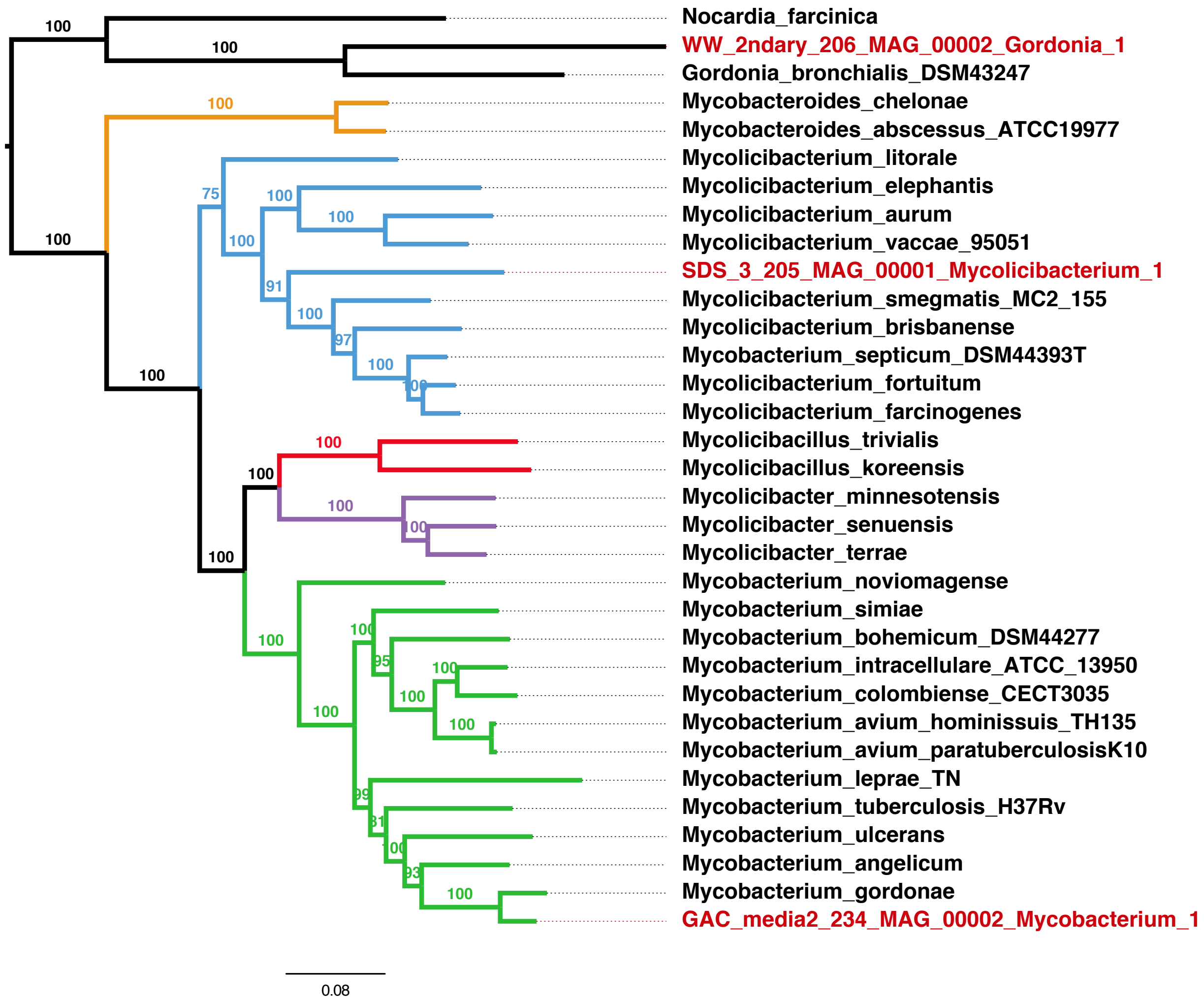

Figure S10. Phylogenetic placement of two Mycobacteriaceae metagenome assembled genomes (MAGs). The tree was constructed using 30 reference genomes chosen to represent the clades described in Gupta *et al.* (2018) (colored on tree). Protein alignments of 108 single-copy genes were concatenated resulting in an alignment with 32150 columns, and a maximum likelihood phylogenetic tree was constructed using RAxML with rapid bootstrapping (100 bootstraps). Scale bar represents 0.06 amino acid substitutions per site.

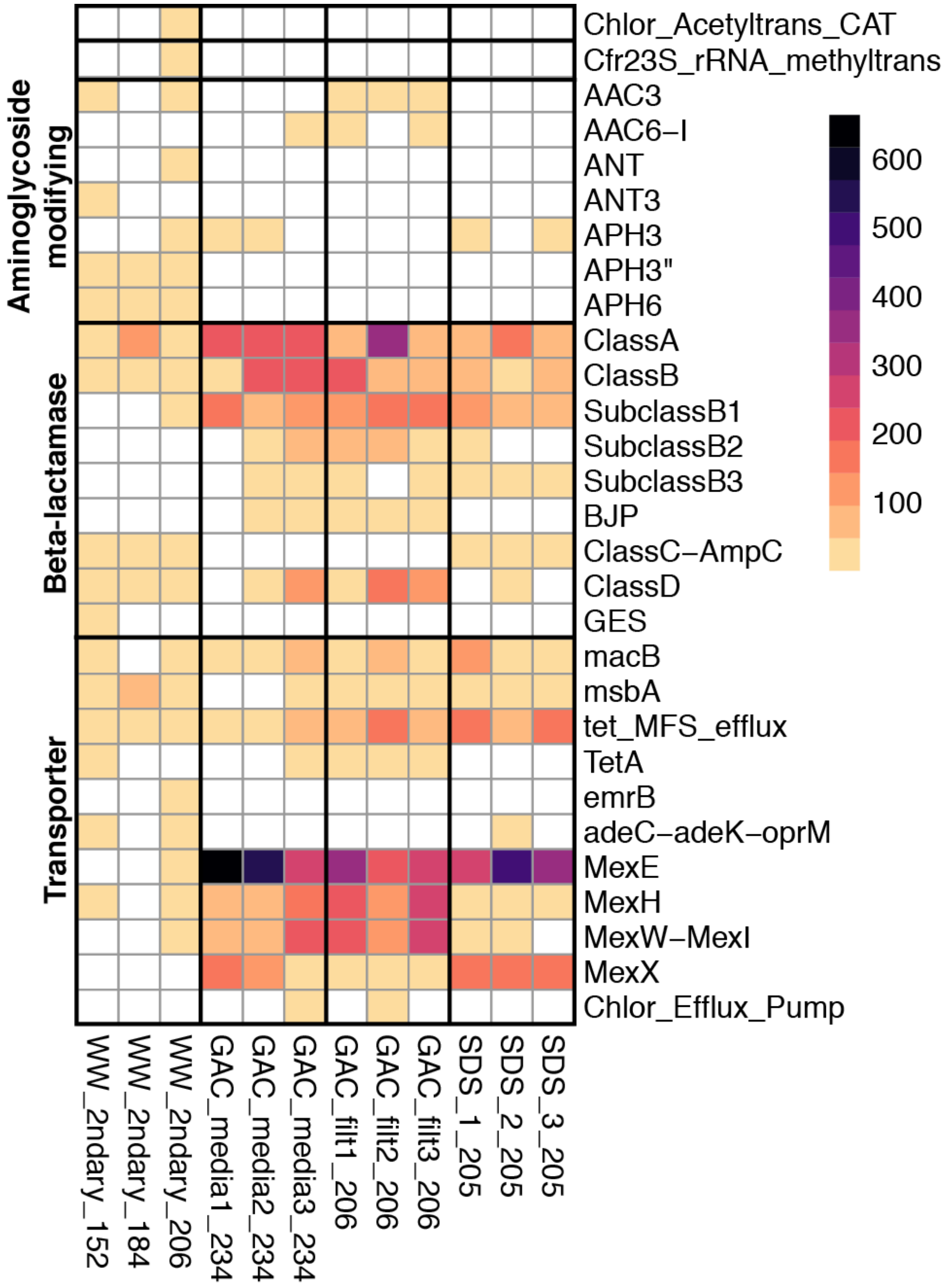

Figure S11. Heatmap of relative abundance of antibiotic resistance genes by class in each sample. Within each sample, coverage was summed over all members of each individual ResFam gene family and normalized to coverage per 10 million reads. Of 34 ARGs detected in any sample via HMMsearch against ResFams, we excluded transcriptional regulatory proteins (vanS, soxR, baeR) and major transporters (RND efflux and ABC efflux) from this figure due to their higher potential for false-positives. When summed ARG coverages within each sample were normalized to summed coverages of scaffolds containing the single-copy marker gene ribosomal protein S3 to account for differences in community diversity, the results were similar (not shown).

References

Brown, C. T., Olm, M. R., Thomas, B. C., and Banfield, J. F. (2016). Measurement of bacterial replication rates in microbial communities. *Nat. biotechnol.* 34, 1256–1263. doi:10.1038/nbt.3704.

Campbell, J. H., O'Donoghue, P., Campbell, A. G., Schwientek, P., Sczyrba, A., Woyke, T., et al. (2013). UGA is an additional glycine codon in uncultured SR1 bacteria from the human microbiota. *Proc. Natl. Acad. Sci. U.S.A.* 110, 5540–5545. doi:10.1073/pnas.1303090110.

Edgar, R. C. (2004). MUSCLE: multiple sequence alignment with high accuracy and high throughput. *Nucleic Acids Res.* 32, 1792–1797. doi:10.1093/nar/gkh340.

Edgar, R. C. (2010). Search and clustering orders of magnitude faster than BLAST. *Bioinformatics* 26, 2460–2461. doi:10.1093/bioinformatics/btq461.

Eren, A. M., Esen, Ö. C., Quince, C., Vineis, J. H., Morrison, H. G., Sogin, M. L., et al. (2015). Anvi"o: an advanced analysis and visualization platform for "omics data. *PeerJ* 3, e1319. doi:10.7717/peerj.1319.

Gupta, R. S., Lo, B., and Son, J. (2018). Phylogenomics and Comparative Genomic Studies Robustly Support Division of the Genus Mycobacterium into an Emended Genus Mycobacterium and Four Novel Genera. *Front. Microbio.* 9, 67. doi:10.3389/fmicb.2018.00067.

Hammes, F., and Egli, T. (2007). A flow cytometric method for AOC determination. *Techneau*, 1–20.

Hug, L. A., Baker, B. J., Anantharaman, K., Brown, C. T., Probst, A. J., Castelle, C. J., et al. (2016). A new view of the tree of life. *Nature Microbiology* 1, 16048. doi:10.1038/nmicrobiol.2016.48.

Lagesen, K., Hallin, P., Rodland, E. A., Staerfeldt, H. H., Rognes, T., and Ussery, D. W. (2007). RNAmmer: consistent and rapid annotation of ribosomal RNA genes. *Nucleic Acids Res.* 35, 3100–3108. doi:10.1093/nar/gkm160.

Langmead, B., and Salzberg, S. L. (2012). Fast gapped-read alignment with Bowtie 2. *Nat. Methods* 9, 357–359. doi:10.1038/nmeth.1923.

Li, H., Handsaker, B., Wysoker, A., Fennell, T., Ruan, J., Homer, N., et al. (2009). The Sequence Alignment/Map format and SAMtools. *Bioinformatics* 25, 2078–2079. doi:10.1093/bioinformatics/btp352.

Nearing, J. T., Douglas, G. M., Comeau, A. M., and Langille, M. G. I. (2018). Denoising the Denoisers: An independent evaluation of microbiome sequence error-correction methods. e26566v1. doi:10.7287/peerj.preprints.26566v1.

Olm, M. R., Brown, C. T., Brooks, B., and Banfield, J. F. (2017). dRep: a tool for fast and accurate genomic comparisons that enables improved genome recovery from metagenomes through de-replication. *ISME J* 11, 2864–2868. doi:10.1038/ismej.2017.126.

Parks, D. H., Imelfort, M., Skennerton, C. T., Hugenholtz, P., and Tyson, G. W. (2015). CheckM: assessing the quality of microbial genomes recovered from isolates, single cells, and metagenomes. *Genome Res.* 25, 1043–1055. doi:10.1101/gr.186072.114.

Peng, Y., Leung, H. C. M., Yiu, S. M., and Chin, F. Y. L. (2012). IDBA-UD: a *de novo* assembler for single-cell and metagenomic sequencing data with highly uneven depth. *Bioinformatics* 28, 1420–1428. doi:10.1093/bioinformatics/bts174.

Seemann, T. (2014). Prokka: rapid prokaryotic genome annotation. *Bioinformatics* 30, 2068–2069. doi:10.1093/bioinformatics/btu153.

Truong, D. T., Franzosa, E. A., Tickle, T. L., Scholz, M., Weingart, G., Pasolli, E., et al. (2015). MetaPhlAn2 for enhanced metagenomic taxonomic profiling. *Nat. Methods* 12, 902–903. doi:10.1038/nmeth.3589.
