# Supplementary material for: The Water Microbiome Through a Pilot Scale Advanced Treatment Facility for Direct Potable Reuse
Source: Front Microbiol. 2019 May 8;10:993. doi: 10.3389/fmicb.2019.00993 (PMC6517601; doi:10.3389/fmicb.2019.00993)
Supplement: Supplementary file 2 [file Data_Sheet_2.PDF]

1. Load libraries and data

Load libraries and set colors

In [1]:
```
library(phyloseq); packageVersion("phyloseq")
library(dada2)
library(ggplot2); packageVersion("ggplot2")
library(tidyr)
library(dplyr)
library(vegan); packageVersion("vegan")
library(scales)
library(repr)
library(reshape2)
library(genefilter)
library(VennDiagram)
library(svglite)
library(gridExtra)
library(DESeq2); packageVersion("DESeq2")

options(jupyter.plot_mimetypes = c("text/plain", "image/png" ))

#colorblind color vector for taxonomy plots
colors <- c("#89C5DA", "#DA5724", "#74D944", "#CE50CA", "#3F4921", "#C0717C", "#CBD588", "#5F7FC7",
            "#673770", "#D3D93E", "#38333E", "#508578", "#D7C1B1", "#689030", "#AD6F3B", "#CD9BCD",
            "#D14285", "#6DDE88", "#652926", "#7FDCC0", "#C84248", "#8569D5", "#5E738F", "#D1A33D",
            "#8A7C64", "#599861", "orange", "666666", "gray80", "#FFCC00")

set.seed(100)
```

```
[1] ‘1.24.2’

Loading required package: Rcpp

[1] ‘3.0.0’

Warning message:
“package ‘dplyr’ was built under R version 3.5.1”
Attaching package: ‘dplyr’

The following objects are masked from ‘package:stats’:

    filter, lag

The following objects are masked from ‘package:base’:

    intersect, setdiff, setequal, union

Loading required package: permute
Loading required package: lattice
This is vegan 2.5-2

[1] ‘2.5.2’
```

```
Attaching package: 'reshape2'

The following object is masked from 'package:tidyr':

    smiths

Loading required package: grid
Loading required package: futile.logger

Attaching package: 'gridExtra'

The following object is masked from 'package:dplyr':

    combine

Loading required package: S4Vectors
Loading required package: stats4
Loading required package: BiocGenerics
Loading required package: parallel

Attaching package: 'BiocGenerics'

The following objects are masked from 'package:parallel':

    clusterApply, clusterApplyLB, clusterCall, clusterEvalQ,
    clusterExport, clusterMap, parApply, parCapply, parLapply,
    parLapplyLB, parRapply, parSapply, parSapplyLB

The following object is masked from 'package:gridExtra':

    combine

The following objects are masked from 'package:dplyr':

    combine, intersect, setdiff, union

The following objects are masked from 'package:stats':

    IQR, mad, sd, var, xtabs

The following objects are masked from 'package:base':

    anyDuplicated, append, as.data.frame, basename, cbind, colMeans,
    colnames, colSums, dirname, do.call, duplicated, eval, evalq,
    Filter, Find, get, grep, grepl, intersect, is.unsorted, lapply,
    lengths, Map, mapply, match, mget, order, paste, pmax, pmax.int,
    pmin, pmin.int, Position, rank, rbind, Reduce, rowMeans, rownames,
    rowSums, sapply, setdiff, sort, table, tapply, union, unique,
    unsplit, which, which.max, which.min


Attaching package: 'S4Vectors'

The following objects are masked from 'package:dplyr':

    first, rename

The following object is masked from 'package:tidyr':

    expand

The following object is masked from 'package:base':

    expand.grid

Loading required package: IRanges

Attaching package: 'IRanges'

The following objects are masked from 'package:dplyr':

    collapse, desc, slice

The following object is masked from 'package:phyloseq':

    distance

Loading required package: GenomicRanges
Loading required package: GenomeInfoDb
Loading required package: SummarizedExperiment
Loading required package: Biobase
Welcome to Bioconductor

    Vignettes contain introductory material; view with
    'browseVignettes()'. To cite Bioconductor, see
    'citation("Biobase")', and for packages 'citation("pkgname")'.


Attaching package: 'Biobase'

The following object is masked from 'package:phyloseq':

    sampleNames

Loading required package: DelayedArray
Loading required package: matrixStats

Attaching package: 'matrixStats'

The following objects are masked from 'package:Biobase':

    anyMissing, rowMedians

The following objects are masked from 'package:genefilter':

    rowSds, rowVars

The following object is masked from 'package:dplyr':

    count

Loading required package: BiocParallel

Attaching package: 'DelayedArray'
```

```
The following objects are masked from ‘package:matrixStats’:

    colMaxs, colMins, colRanges, rowMaxs, rowMins, rowRanges

The following objects are masked from ‘package:base’:

    aperm, apply


[1] ‘1.20.0’
```

Load data and make phyloseq object

In [2]:
```
taxa <- readRDS("/SCIENCE/Nelson_lab/data_files_nelson/el_paso_16S/dada2_full/tax_final.rds")
seqtab.nochim <- readRDS("/SCIENCE/Nelson_lab/data_files_nelson/el_paso_16S/dada2_full/seqtab_final.rds")

# Make a data.frame holding the sample data where rownames are sampleIDs and headers are metadata parameters
metadata <- read.table("/SCIENCE/Nelson_lab/data_files_nelson/el_paso_16S/sample_data.txt",
                       header=TRUE, row.names = 1, sep="\t")
metadata$SampleID <- row.names(metadata)
metadata$combined_name <- paste(metadata$Description, metadata$Day, sep="_")
metadata$longer_name <- paste(metadata$Location, metadata$combined_name, sep="_")

#unique(metadata$Location) #to show all locations, then set factor levels so they are ordered along treatment train
metadata$Location <- factor(metadata$Location,
                            levels=c("WW_2ndary", "Chloramine", "MF", "NF-RO",
                                     "AOP", "Unknown", "GAC", "ESB", "SDS",
                                     "Amplification", "Extraction", "Field", "Mock"))

# Construct phyloseq object from dada2 outputs and metadata table
ps <- phyloseq(otu_table(seqtab.nochim, taxa_are_rows=FALSE),
               sample_data(metadata),
               tax_table(taxa))

#exported the sequences to fasta for other analyses
#uniquesToFasta(seqtab.nochim, "/SCIENCE/Nelson_lab/data_files_nelson/el_paso_16S/dada2_full/all_seqs.fasta")

ps
```

```
phyloseq-class experiment-level object
otu_table()   OTU Table:         [ 5336 taxa and 168 samples ]
sample_data() Sample Data:       [ 168 samples by 28 sample variables ]
tax_table()   Taxonomy Table:    [ 5336 taxa by 6 taxonomic ranks ]
```

In [3]:
```
##Sample counts before filtering
sample_count_prefiltering <- metadata %>% group_by(Type, Location) %>% summarize(sample_count=length(SampleID))
sample_count_prefiltering <- dcast(sample_count_prefiltering, Location~Type, value.var="sample_count")
sample_count_prefiltering
```

Location	Biofilm	Blank	Bulk	Control	Media
WW_2ndary	3	NA	6	NA	NA
Chloramine	3	NA	4	NA	NA
MF	8	NA	5	NA	NA
NF-RO	6	NA	3	NA	NA
AOP	1	NA	NA	NA	NA
Unknown	1	NA	NA	NA	NA
GAC	9	NA	15	NA	12
ESB	4	NA	NA	NA	NA
SDS	52	NA	11	NA	NA
Amplification	NA	3	NA	NA	NA
Extraction	NA	17	NA	NA	NA
Field	NA	3	NA	NA	NA
Mock	NA	NA	NA	2	NA

2. Contaminant and sample removal

In [4]:
```
## Remove mock controls and samples that look very weird based on previous data exploration

#remove low-biomass samples that look strongly contaminated with influent
ps_pruned <- prune_samples(sample_names(ps) != "11", ps)
ps_pruned <- prune_samples(sample_names(ps_pruned) != "68", ps_pruned) #SDS
ps_pruned <- prune_samples(sample_names(ps_pruned) != "12", ps_pruned) #NF-RO

#remove samples that look strongly contaminated with MF filtrate
#(Two sequences took over the whole library and one is the Mycobacterium from all MF samples)
ps_pruned <- prune_samples(sample_names(ps_pruned) != "69", ps_pruned) #SDS

#remove sample whose barcodes were unknown
ps_pruned <- prune_samples(sample_names(ps_pruned) != "200", ps_pruned)

#remove mock community positive controls
ps_nomock <- prune_samples(sample_names(ps_pruned) != "Mock1", ps_pruned)
ps_nomock <- prune_samples(sample_names(ps_nomock) != "Mock2", ps_nomock)
```

In [5]:
```
#save mock data for comparison to metagenomics
ps_mock <- subset_samples(ps_pruned, subset = Location %in% "Mock")
ps_mock <- filter_taxa(ps_mock, filterfun(kOverA(1, 0)), TRUE) #where number of samples=1, min_reads>0
ps_mock_perc <- transform_sample_counts(ps_mock, function(OTU) 100*OTU/sum(OTU))
#saveRDS(ps_mock_perc, "/SCIENCE/Nelson_lab/write-ups/EPseq_paper/revised/amplicon_mock_controls.rds")
```

In [6]:
```
sample_data(ps_pruned)
```

	Plate	step_number	Type	Location	Description	Day	DNA_conc	is_blank	field_blank	extraction_blank	⋯	Forward_Barcode	Reverse_Barcode	reads_unfiltered	reads_filtered_D
**103**	X	6	Biofilm	AOP	Treated	234	NA	n	na	104	⋯	TCGACGAG	ACCTACTG	97284	69057
**104**	X	0	Blank	Extraction	PowerBiofilm	234	NA	y	na	104	⋯	TACGAGAC	GAGCTCAT	32455	25056
**105**	X	7	Biofilm	GAC	1	234	0.186	n	na	na	⋯	ACGTCTCG	GTATGACG	143531	99912
**106**	X	7	Biofilm	GAC	1	234	0.131	n	na	na	⋯	GTCAGATA	TAGACTGA	131682	97884
**107**	Y	7	Biofilm	GAC	1	234	0.0868	n	na	na	⋯	GACACTGA	AGCGCTAT	54684	40911
**109**	Y	9	Biofilm	SDS	1	184	NA	n	na	117	⋯	ATATACAC	CTAGCTCG	76189	57130
**10**	P	2	Bulk	Chloramine	Treated	184	0.388	n	16	na	⋯	ATATACAC	TCACGATG	98914	73091
**110**	Y	9	Biofilm	SDS	1	184	NA	n	na	117	⋯	ACGACGTG	TAGACTGA	94841	73282
**111**	Y	9	Biofilm	SDS	2	184	NA	n	na	117	⋯	TGCGTACG	AGCGCTAT	151098	109287
**112**	Y	9	Biofilm	SDS	2	184	NA	n	na	117	⋯	CGTCGCTA	AGTCTAGA	87325	64883
**113**	Y	9	Biofilm	SDS	2	184	NA	n	na	117	⋯	CTAGAGCT	CTCTAGAG	39055	29447
**114**	X	9	Biofilm	SDS	3	184	NA	n	na	117	⋯	CGTTACTA	CTAGCTCG	11098	7975
**115**	Y	9	Biofilm	SDS	3	184	0.0216	n	na	117	⋯	CGTCGCTA	CTCTAGAG	84602	65739
**116**	X	9	Biofilm	SDS	3	184	NA	n	na	117	⋯	AGAGTCAC	GTATGACG	64376	49092
**117**	X	0	Blank	Extraction	PowerBiofilm	na	NA	y	na	117	⋯	ACGTCTCG	ACCTACTG	323	166
**118**	X	7	Biofilm	GAC	2	234	0.0776	n	na	124	⋯	GATCGTGT	TCACGATG	52903	38588
**119**	X	7	Biofilm	GAC	2	234	0.07	n	na	124	⋯	CGTTACTA	AGTCTAGA	39835	29324
**120**	Y	7	Biofilm	GAC	2	234	0.203	n	na	124	⋯	ACGACGTG	AGTCTAGA	130753	95809
**121**	Y	7	Biofilm	GAC	3	234	0.137	n	na	124	⋯	GCTCTAGT	CTCTAGAG	88695	69199
**122**	Y	7	Biofilm	GAC	3	234	0.074	n	na	124	⋯	TAGTGTAG	CATGAGGA	18136	13843
**123**	Y	7	Biofilm	GAC	3	234	0.108	n	na	124	⋯	GCTCTAGT	CTAGCTCG	125662	96980
**124**	Y	0	Blank	Extraction	PowerBiofilm	na	NA	y	na	124	⋯	TGCGTACG	CATGAGGA	459	259
**125**	X	9	Biofilm	SDS	1	193	0.237	n	na	134	⋯	AGAGTCAC	CTCTAGAG	7919	5924
**126**	X	9	Biofilm	SDS	1	193	0.112	n	na	134	⋯	ACGTCTCG	AGCGCTAT	254	113
**127**	Y	9	Biofilm	SDS	1	193	0.0904	n	na	134	⋯	CTAGAGCT	GGTATGCT	96837	73871
**128**	P	9	Biofilm	SDS	2	193	0.0264	n	na	134	⋯	GACACTGA	GGTATGCT	54811	40766
**129**	Y	9	Biofilm	SDS	2	193	NA	n	na	134	⋯	TGCGTACG	GTATGACG	123335	94891
**130**	Z	9	Biofilm	SDS	2	193	0.02	n	na	134	⋯	GATCGTGT	GTATGACG	147905	113862
**131**	Y	9	Biofilm	SDS	3	193	0.0332	n	na	134	⋯	GACACTGA	CTCTAGAG	577	339
**132**	Y	9	Biofilm	SDS	3	193	NA	n	na	134	⋯	CTAGAGCT	ACCTACTG	105051	81707
⋮	⋮	⋮	⋮	⋮	⋮	⋮	⋮	⋮	⋮	⋮	⋱	⋮	⋮	⋮	⋮
**73**	Y	6	Bulk	SDS	3	228	0.203	n	na	74	⋯	GCTCTAGT	CATGAGGA	163492	122297
**74**	X	0	Blank	Extraction	PhenolChloroform	na	NA	y	na	74	⋯	CTACTATA	TAGACTGA	2069	1514
**75**	X	1	Biofilm	WW_2ndary	Feed	234	1.2	n	na	84	⋯	GTCAGATA	AGCGCTAT	192	97
**76**	Y	1	Biofilm	WW_2ndary	Feed	234	17.5	n	na	84	⋯	TAGTGTAG	GAGCTCAT	109536	86032
**77**	X	1	Biofilm	WW_2ndary	Feed	234	5.32	n	na	84	⋯	TCGACGAG	GGTATGCT	122	52
**78**	X	2	Biofilm	Chloramine	Treated	234	7.36	n	na	84	⋯	TACGAGAC	AGTCTAGA	42532	33175
**79**	X	2	Biofilm	Chloramine	Treated	234	12.7	n	na	84	⋯	GTCAGATA	TCACGATG	73162	51816
**80**	Y	2	Biofilm	Chloramine	Treated	234	5.12	n	na	84	⋯	TAGTGTAG	TAGACTGA	94105	67956
**81**	X	3	Biofilm	MF	B	234	16.8	n	na	84	⋯	ACGTCTCG	CTCTAGAG	369	223
**82**	Y	3	Biofilm	MF	B	234	15.7	n	na	84	⋯	TGCGTACG	TAGACTGA	88321	66682
**83a**	X	3	Biofilm	MF	B	234	21.6	n	na	84	⋯	AGAGTCAC	TCACGATG	54524	41482
**83b**	X	3	Biofilm	MF	B	234	21.6	n	na	84	⋯	CGTTACTA	GTATGACG	1242	862
**83c**	Y	3	Biofilm	MF	B	234	21.6	n	na	84	⋯	TGCGTACG	ACCTACTG	134245	104385
**84**	X	0	Blank	Extraction	PowerBiofilm	234	NA	y	na	84	⋯	TACGAGAC	TCACGATG	14712	10810
**85**	Y	3	Biofilm	MF	A	234	17.5	n	na	94	⋯	CGTCGCTA	ACCTACTG	134720	103286
**86**	Y	3	Biofilm	MF	A	234	19.6	n	na	94	⋯	ACGACGTG	GAGCTCAT	101095	78038
**87**	Z	3	Biofilm	MF	A	234	17.5	n	na	94	⋯	GATCGTGT	TCGAGCTC	113595	83882
**88**	X	5	Biofilm	NF-RO	RO_Feed	234	1.53	n	na	94	⋯	CGTTACTA	CTCTAGAG	10393	7367
**89**	X	5	Biofilm	NF-RO	RO_Feed	234	1.32	n	na	94	⋯	GATCGTGT	TAGACTGA	133772	97902
**90**	X	5	Biofilm	NF-RO	RO_Feed	234	2.21	n	na	94	⋯	CGTTACTA	TAGACTGA	51445	38272
**92**	X	5	Biofilm	NF-RO	RO_Perm	234	0	n	na	94	⋯	GTCAGATA	GAGCTCAT	73374	51111
**93**	Y	5	Biofilm	NF-RO	RO_Perm	234	0	n	na	94	⋯	ATATACAC	AGCGCTAT	60079	45948
**94**	X	0	Blank	Extraction	PowerBiofilm	234	NA	y	na	94	⋯	TACGAGAC	TCGAGCTC	19291	14435
**95**	P	4	Biofilm	NF-RO	NF_Feed	234	0.05	n	na	104	⋯	ACGACGTG	GGTATGCT	54437	39666
**9**	Z	1	Bulk	WW_2ndary	Feed	184	18.4	n	16	na	⋯	GACACTGA	ACCTACTG	72523	49701
**Mock1**	Y	M	Control	Mock	1	na	NA	n	na	na	⋯	ACGACGTG	TCGAGCTC	81655	58796
**Mock2**	Y	M	Control	Mock	2	na	NA	n	na	na	⋯	ATATACAC	GTATGACG	87275	61875
**Neg1**	P	0	Blank	Amplification	PlateP	na	NA	y	na	na	⋯	AGAGTCAC	CATGAGGA	114110	85546
**Neg2**	X	0	Blank	Amplification	PlateX	na	NA	y	na	na	⋯	GATCGTGT	GAGCTCAT	11559	7835
**Neg3**	Y	0	Blank	Amplification	PlateY	na	NA	y	na	na	⋯	CGTCGCTA	GTATGACG	3138	1035

In [7]:

```
#run DESeq2 on all bulk and blank ASVs together
#identify ASVs significantly more prevalent in bulk
#keep ASVs present only in bulk and ASVs significantly more prevalent in bulk than blank

## Preparing for DESeq

#select only samples of interest
ps_bulk <- subset_samples(ps_nomock, subset = Type %in% c("Bulk"))
ps_blank <- subset_samples(ps_nomock, subset = Type %in% c("Blank"))

blanks_list <- c("Amplification_PlateX_na", "Amplification_PlateY_na", "Amplification_PlateP_na",
  "Extraction_PhenolChloroform_na", "Field_NA_184", "Field_NA_206", "Field_NA_213") #blanks that apply to bulk data

ps_blank <- subset_samples(ps_blank, subset = longer_name %in% c(blanks_list))

#merge bulk and blank samples back together and filter for only taxa that occur in at least one sample
ps_bulk_blank <- merge_phyloseq(ps_bulk, ps_blank)
ps_bulk_blank <- filter_taxa(ps_bulk_blank, filterfun(kOverA(1, 0)), TRUE) #where number of samples=1, min_reads>0

#remove samples with too few reads
ps_bulk_blank <- prune_samples(sample_sums(ps_bulk_blank)>=10000, ps_bulk_blank) #remove failed samples

## Filter taxa occurring in bulk samples and taxa occuring in blank.
#for later: Get bulk-only taxa and intersecting taxa.

ps_blank_occur <- filter_taxa(ps_blank, filterfun(kOverA(1, 0)), TRUE) #where number of samples=1, min_reads>0
ps_bulk_occur <- filter_taxa(ps_bulk, filterfun(kOverA(1, 0)), TRUE) #where number of samples=1, min_reads>0

blank_taxa <- row.names(tax_table(ps_blank_occur))
bulk_taxa <- row.names(tax_table(ps_bulk_occur))
intersecting_taxa <- intersect(bulk_taxa, blank_taxa)
bulk_only_taxa <- setdiff(bulk_taxa, intersecting_taxa)
blank_only_taxa <- setdiff(blank_taxa, intersecting_taxa)

ps_bulk_only <- prune_taxa(bulk_only_taxa, ps_bulk_blank)


ps_bulk_blank
```

```
phyloseq-class experiment-level object
otu_table()   OTU Table:         [ 3033 taxa and 40 samples ]
sample_data() Sample Data:       [ 40 samples by 28 sample variables ]
tax_table()   Taxonomy Table:    [ 3033 taxa by 6 taxonomic ranks ]
```

In [8]:
```
#run DESeq on the bulk and blank taxa

dds = phyloseq_to_deseq2(ps_bulk_blank, ~ is_blank)
dds = DESeq(dds, test="Wald", fitType="local")

#analyze results of DESeq filtering for significance
res = results(dds, cooksCutoff = FALSE)
alpha = 0.01
sigtab = res[which(res$padj < alpha), ]
sigtab = cbind(as(sigtab, "data.frame"), as(tax_table(ps_bulk_blank)[rownames(sigtab), ], "matrix"))
sigtab
significant_taxa <- row.names(sigtab[sigtab$log2FoldChange < 0,]) #remove anything significantly enriched in blanks
```

```
converting counts to integer mode
estimating size factors
estimating dispersions
gene-wise dispersion estimates
mean-dispersion relationship
final dispersion estimates
fitting model and testing
-- replacing outliers and refitting for 1705 genes
-- DESeq argument 'minReplicatesForReplace' = 7
-- original counts are preserved in counts(dds)
estimating dispersions
fitting model and testing
```

CGTTGTCCGGAATTACTGGGCGTAAAGAGCTCGTAGGTGGTTTGTCACGTTGTCCGTGAAAACTCACAGCTTAACTGTGGGCGTGCGGGCGATACGGGCAGACTGGAGTACTGTAGGGGAGACTGGAATTCCTGGTGT
CGTTGCTCGGAATCACTGGGCGTAAAGGGCGCGTAGGCGGCGTTTTAAGTCGGGGGTGAAAGCCTGTGGCTCAACCACAGAATGGCCTTCGATACTGGGACGCTTGAGTATGGTAGAGGTTGGTGGAACTGCGAGTG
CGTTGCTCGGAATCACTGGGCGTAAAGGGTGCGTAGGCGGGTCTTTAAGTCAGGGGTGAAATCCTGGAGCTCAACTCCAGAACTGCCTTTGATACTGAAGATCTTGAGTTCGGGAGAGGTGAGTGGAACTGCGAGTG
CGTTAATCGGAATTACTGGGCGTAAAGCGTGCGCAGGCGGATATGTAAGTTAGAAGTGAAATCCCCGGGCTCAACCTGGGAATGGCTTTTAAGACTGCGTATCTAGAGTTTGTCAGAGGGGGGTGGAATTCCAAGTGTA
CGTTGTTCGGAATCACTGGGCGTAAAGCGCACGTAGGCGGATTTGTAAGTCAGGGGTGAAATCCCGGGGCTCAACCTCGGAACTGCCTTTGATACTGCGAATCTTGAGTCCGATAGAGGTGGGTGGAATTCCTAGTGT
CGTTATCCGGAATCATTGGGTTTAAAGGGTCCGTAGGCGGTCTTATAAGTCAGTGGTGAAAGCCCATCGCTCAACGATGGAACTGCCATTGATACTGTAAGACTTGAATGCTTAGGAAGTAACTAGAATATGTAGTGTAGC
CGTTGTTCGGAATCACTGGGCGTAAAGCGCACGTAGGCGGATCTTTAAGTCAGGGGTGAAATCCCGAGGCTCAACCTCGGAACTGCCTTTGATACTGGAGGTCTCGAGTCCGGGAGAGGTGAGTGGAACTGCGAGTG
CGTTGCTCGGAATCACTGGGCGTAAAGGGTGCGTAGGCGGGTCTTTAAGTCAGAGGTGAAAGCCTGGAGCTCAACTCCAGAACTGCCTTTGATACTGAGGATCTCGAGTTCGGGAGAGGTGAGTGGAACTGCGAGTG
CGTTAATCGGAATTACTGGGCGTAAAGCGTGCGCAGGCGGATATGTAAGTTAGGAGTGAAATCCCCGGGCTCAACCTGGGAATGGCTTTTAAGACTGCGTATCTAGAGTTTGTCAGAGGGGGGTGGAATTCCAAGTGTA
CGTTATCCGGAATCATTGGGTTTAAAGGGTCCGTAGGCGGTTGAATAAGTCAGTGGTGAAATCCGGCAGCTTAACTGTCGAACTGCCATTGATACTGTTTAACTTGAATTACTGTGAAGTAACTAGAATATGTAGTGTAGC
CGTTAATCGGAATTACTGGGCGTAAAGCGTGCGCAGGCGGTTATATAAGACAGATGTGAAATCCCCGGGCTCAACCTGGGAACTGCATTTGTGACTGTATAGCTAGAGTACGGCAGAGGGGGATGGAATTCCGCGTGTA
CGTTGTCCGGATTTATTGGGCGTAAAGAGCTCGTAGGCGGTTTGTCGCGTCGGATGTGAAAACCCAATGGCTTAACCTTGGGCCTGCATTCGATACGGGCAAACTAGAGTGTGGTAGGGGAGACTGGAACTCCTGGTG
CGTTAATCGGAATTACTGGGCGTAAAGCGTGCGCAGGCGGACTTTTAAGCCAGATGTGAAAGCCCCGAGCTTAACTTGGGAATTGCGTTTGGAACTGGGAGTCTAGAGTCTGTCAGAGGGGGATGGAATTCCACGTGT
CGTTATCCGGAATTACTGGGCGTAAAGGGTGCGTAGGTGGTTTCTTAAGTCAGAGGTGAAAGGCTACGGCTCAACCGTAGTAAGCCTTTGAAACTGGGAAACTTGAGTGCAGGAGAGGAGAGTGGAATTCCTAGTGTAG
CGTTAATCGGAATTACTGGGCGTAAAGCGTGCGCAGGCGGTTTGCTAAGACAGGTGTGAAATCCCCGGGCTTAACCTGGGAACTGCGCTTGTGACTGGCAGGCTAGAGTACGGCAGAGGGGGGTGGAATTCCTGGTG
CGTTAATCGGAATTACTGGGCGTAAAGCGTGCGCAGGCGGTTTTGTAAGACAGATGTGAAATCCCCGGGCTTAACCTGGGAACTGCGTTTGTGACTGCAAGGCTAGAGTACGGCAGAGGGGGGTGGAATTCCTGGTGT
CGTTAATCGGAATTACTGGGCGTAAAGCGCGTGTAGGCGGTTGGCTAAGTCAGATGTGAAAGCCCCGGGCTCAACCTGGGAATTGCATTTGATACTGGCTGGCTAGAATGTGGTAGAGGGAGGCGGAATTCCCGGTGT
CGTTAATCGGAATTACTGGGCGTAAAGCGTGCGCAGGCGGTTATGTAAGACAGAGGTGAAATCCCCGGGCTCAACCTGGGAACTGCCTTTGTGACTGCATAGCTGGAGTGCGGCAGAGGGGGATGGAATTCCGCGTG
CGTTGTCCGGAATTACTGGGCGTAAAGAGCTCGTAGGCGGTTTGTCGCGTCGTCTGTGAAATTCTGCAGCTTAACTGTAGGCGTGCAGGCGATACGGGCAGACTTGAGTACTACAGGGGAGACTGGAATTCCTGGTGT
CGTTAATCGGAATTACTGGGCGTAAAGCGCGCGTAGGCGGTTTGTTAAGTCGGATGTGAAATCCCTGGGCTCAACCTAGGCACTGCATCCGATACTGGCTAACTAGAGTGTGGGAGAGGAAGGTAGAATTCCAGGTGTA
CGTTAATCGGAATTACTGGGCGTAAAGCGTGCGCAGGCGGTGATGTAAGACAGATGTGAAATCCCCGGGCTCAACCTGGGAACTGCGTTTGTGACTGCATCACTCGAGTACGGCAGAGGGAGGTGGAATTCCGCGTG
CGTTGTCCGGAATTACTGGGCGTAAAGAGCTCGTAGGTGGTTTGTCGCGTTGTTCGTGAAAACTCACAGCTCAACTGTGGGCGTGCGGGCGATACGGGCAGACTGGAGTACTGCAGGGGAGACTGGAATTCCTGGTG
CGTTAATCGGAATTACTGGGCGTAAAGCGTGCGCAGGCGGTTGTGCAAGACAGGTGTGAAATCCCCGGGCTTAACCTGGGAACTGCACTTGTGACTGCACAGCTGGAGTACGGCAGAGGGGGATGGAATTCCGCGTG
CGTTATCCGGATTCACTGGGTTTAAAGGGTGCGTAGGTGGGTTGATAAGTCAGTGGTGAAAGCTCCGAGCTTAACTTGGAAACTGCCATTGATACTGTCAGTCTTGAATGTCGTAGAGGTAAGCGGAATGTTGCATGTAG
CGTTGTCCGGAGTTACTGGGCGTAAAGCGCGCGCAGGCGGCTCGTCAAGCCTGACGTGAAAGCCCCCGGCTCAACCGGGGAGGGTCGTCGGGGACTGGCGGGCTTGAGGTCGGCAGGGGTTGGTGGAATTCCCG
CATTAATCGGATTTATTGGGCGTAAAGGGCGCGTAGGCGGGAAAGTCAGTCAGATGTGAAAGCCCAGGGCTCAACCCTGGAACAGCATTTGAAACTACTTTTCTAGAGGGTAGGCGGAGAAAACAGAATTCCACGTGTA
CATTAATCGGATTTATTGGGCGTAAAGGGCGCGTAGGCGGGAAAGTCAGTCAGATGTGAAAGCCCAGGGCTCAACCCTGGAACAGCATTTGAAACTACTTTTCTAGAGGGTAGGCGGAGAAAACAGAATTCCACGTGTA
CGTTACTCGGAATTACTGGGCGTAAAGCGTGCGTAGGTGGTTCGTTAAGTCTGATGTGAAAGCCCTGGGCTCAACCTGGGAATTGCATTGGATACTGGCGAGCTAGAGTGCGGTAGAGGATGGCGGAATTCCCGGTGTA
CGTTAATCGGAATTACTGGGCGTAAAGCGCGCGTAGGCGGTTTGTTAAGTCGGATGTGAAATCCCTGGGCTCAACCTAGGCACTGCATCCGATACTGGCTGACTAGAGTGTGGGAGAGGAAGGTAGAATTCCAGGTGTA
CGTTAATCGGAATTACTGGGCGTAAAGGGTGCGCAGGCGGATGTTTAAGTTAGAAGTGAAATCCCCGGGCTTAACCTGGGAATTGCTTTTAAGACTGGGCATCTAGAGTTTGTCAGAGGGGGGTGGAATTCCAAGTGTA
CGTTAATCGGAATTACTGGGCGTAAAGCGTGCGCAGGCGGCTTTGCAAGACAGATGTGAAATCCCCGGGCTCAACCTGGGAACTGCATTTGTGACTGCAAGGCTAGAGTACGGTAGAGGGGGATGGAATTCCGCGTGT
CGTTAATCGGAATTACTGGGCGTAAAGCGTGCGCAGGCGGCTTTGCAAGACAGAGGTGAAATCCCCGGGCTCAACCTGGGAACTGCCTTTGTGACTGCAAGGCTAGAGTACGGCAGAGGGGGATGGAATTCCGCGTG
CGTTAATCGGAATTACTGGGCGTAAAGCGTGCGCAGGCGGTTGATTAAGTTTGATGTGAAATCCCCGGGCTTAACCTGGGAACTGCATTGAATACTGGTCAGCTAGAGTGTGTCAGAGGGGGGTAGAATTCCACGTGTA
CGTTAATCGGAATTACTGGGCGTAAAGCGTGCGCAGGCGGTGATGTAAGACAGAGGTGAAATCCCCGGGCTCAACCTGGGAACTGCCTTTGTGACTGCATCGCTGGAGTACGGCAGAGGGGGATGGAATTCCGCGTG
CGTTGTTCGGAATCACTGGGCGTAAAGGGTGCGTAGGTGGCGTGGTAAGTCAGATGTGAAAGCCCGGGGCTCAACCTCGGAATTGCATCCGATACTGCCATGCTCGAGTACTGAAGAGGTGACTAGAATTCTAGGTGT
CGTTGTCCGGATTTACTGGGCGTAAAGGGAGCGTAGGCGGACTTTTAAGTGAGATGTGAAATACCCGGGCTCAACTTGGGTGCTGCATTTCAAACTGGAAGTCTAGAGTGCAGGAGAGGAGAATGGAATTCCTAGTGTA
CGTTGTTCGGAATTACTGGGCTTAAAGCGCACGTAGGCTGTGTCGTAAGTGTCTGGTGAAATCCCACGGCTCACCCGTGGAACTGCCAGGCATACTGCGGTACTCGAGTGTGGTAGGGGAGAGCGGAACTCCTGGTG
CGTTGTCCGGATTTATTGGGTTTAAAGGGTGCGTAGGTTGTGTTGTAAGTCAGTGGTGAAATCCGGCAGCTTAACTGTCGATGTGCCATTGATACTGCAGCACTTGAGTACAGACGAGGTAGGCGGAATTGACGGTGTAG
CGTTAATCGGAATTACTGGGCGTAAAGCGTGCGCAGGCGGACTTTTAAGCCAGATGTGAAAGCCCCGAGCTTAACTTGGGAACCGCGTTTGGAACTGAAAGTCTAGAGTCTGTCAGAGGGGGATGGAATTCCACGTGT

CGTTAATCGGAATTACTGGGCGTAAAGCGCGCGTAGGCGGTTAATTAAGCGAGATGTGAAATCCCTGGGCTCAACCTAGGAATTGCGTTTCGAACTGATTAGCTAGAGTGCAGTAGAGGGTGGTGGAATTTCCGGTGTAG
CGTTGTTCGGAATTACTGGGCGTAAAGCGCACGTAGGCGGATACTTAAGTCAGGGGTGAAATCCCGGGGCTCAACCACGGAACTGCCTTTGATACTGGGTATCTCGAGTCCGGAAGAGGTGAGTGGAATTCCGAGTGTA
CGTTAATCGGAATTACTGGGCGTAAAGCGCGCGTAGGCGGCTTGTTAAGCGGAATGTGAAATCCCCGGGCTCAACCTGGGCACTGCATTCCGAACTGGCTGGCTAGAGTGTGGTAGAGGAAGGTGGAATTCCAGGTGT
CGTTAATCGGAATTACTGGGCGTAAAGCGTGCGCAGGCGGTTTCGTAAGACAGATGTGAAATCCCCGGGCTTAACCTGGGAACTGCGTTTGTGACTGCGAGACTTGAGTGCGGCAGAGGGGGGTGGAATTCCACGTGT
CGTTAATCGGAATTACTGGGCGTAAAGCGCGCGTAGGCGGCTTGCTAAGTCGGATGTGAAATCCCCGAGCTCAACTTGGGAACTGCATTCGATACTGGCTCGCTAGAGTGTGGTAGAGGGAAGTGGAATTCCAGGTGTA
CGTTACTCGGAATCACTGGGCGTAAAGAGCGTGTAGGCGGATTGATAAGTTTGAAGTGAAATCCTATAGCTTAACTATAGAACTGCTTTGAAAACTGTTAATCTAGAATGTGGGAGAGGTAGATGGAATTTCTGGTGTAGGG
CGTTAATCGGAATTACTGGGCGTAAAGGGTGCGTAGGTGGTTTATTAAGTTATCTGTGAAATTCCTGGGCTTAACCTGGGCAGGTCAGATAAGACTGGTGGACTAGAGTATGGGAGAGGGTAGTGGAATTTCCGGTGTAGC
CGTTATCCGGAATCACTGGGCGTAAAGGGAGCGTAGGCGGTTTGACAAGCCAGGGGTGAAAGGCAATAGCTTAACTATTGTAAGCCTTTGGAACTGTCGAACTTGAGTGCAGGAGAGGAAAGTGGAATTCCTAGTGTAG
CGTTGTTCGGAATCATTGGGCGTAAAGCGGGTGTAGGCGGATTTATAAGTCAGGTGTGAAATCCCAGGGCTCAACCCTGGAAGTGCACTTGATACTGTAAATCTTGAGTGTGGTAGAGGTTACTGGAATTGTTGGTGTAGT
CGTTGTTCGGAATTACTGGGCGTAAAGCGCGCGTAGGCGGACTTTCAAGTCAGAGGTGAAATGCCGGGGCTTAACCCCGGAACTGCCTTTGAAACTGTGAGTCTGGAGTTCGAGAGGGGATGGTGGAATTCCTAATGTA
CGTTATCCGGAATCATTGGGCGTAAAGGGTGAGTAGGTGGTATTATTAGTCGTGTGTTAAAGTCCAAGGGCTCAACCTTTGGAATGCATACGAAACGGTAAAACTAGAATATGTAAGAGGTGAGTGGAACTCTATGTGTAGG

In [9]:
```
#plot deseq results
options(repr.plot.width = 5, repr.plot.height = 5)
plotMA(res, ylim=c(-2,2))
#plot shows that the only significantly enriched ASVs were those with negative log fold-change
#(enriched in samples over blanks)
#none were significantly enriched in blanks over samples
```

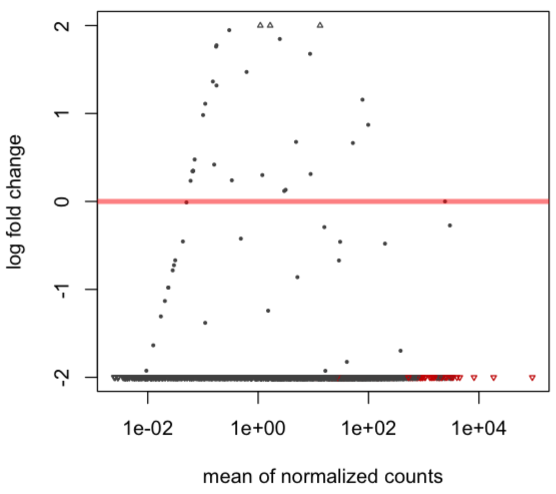

In [10]:
```
## Identify ASVs that were in both blank and bulk and were significantly enriched in bulk
samples_over_blanks <- intersect(intersecting_taxa, significant_taxa)

sigtab[row.names(sigtab) %in% samples_over_blanks,] #what are these taxa?

## Combine to get taxa from bulk-only and taxa significant by DESeq, convert to relative abundance of remaining reads
ps_bulk_blank_sig <- prune_taxa(taxa=union(significant_taxa, bulk_only_taxa), ps_bulk_blank)
sample_data(ps_bulk_blank_sig)$filtered_readcounts <- sample_sums(ps_bulk_blank_sig)

ps_bulk_blank_sig <- prune_samples(sample_sums(ps_bulk_blank_sig)>=300, ps_bulk_blank_sig) #remove samples now with too few reads
ps_bulk_blank_sig_perc <- transform_sample_counts(ps_bulk_blank_sig, function(OTU) 100*OTU/sum(OTU))

ps_bulk_blank_sig

ps_bulk_clean <- subset_samples(ps_bulk_blank_sig, subset= Type %in% "Bulk")
ps_bulk_clean_perc <- transform_sample_counts(ps_bulk_clean, function(OTU) 100*OTU/sum(OTU))

#saveRDS(ps_bulk_clean_perc, "/SCIENCE/Nelson_lab/write-ups/EPseq_paper/revised/amplicon_data_deseq.rds")
```

**CGTTGTCCGGAATTACTGGGCGTAAAGAGCTCGTAGGTGGTTTGTCACGTTGTCCGTGAAAACTCACAGCTTAACTGTGGGCGTGCGGGCGATACGGGCAGACTGGAGTACTGTAGGGGAGACTGGAATTCCTGGTGT**
**CGTTGCTCGGAATCACTGGGCGTAAAGGGTGCGTAGGCGGGTCTTTAAGTCAGGGGTGAAATCCTGGAGCTCAACTCCAGAACTGCCTTTGATACTGAAGATCTTGAGTTCGGGAGAGGTGAGTGGAACTGCGAGTGT**
**CGTTAATCGGAATTACTGGGCGTAAAGCGTGCGCAGGCGGATATGTAAGTTAGAAGTGAAATCCCCGGGCTCAACCTGGGAATGGCTTTTAAGACTGCGTATCTAGAGTTTGTCAGAGGGGGGTGGAATTCCAAGTGTAG**
**CGTTGTTCGGAATCACTGGGCGTAAAGCGCACGTAGGCGGATTTGTAAGTCAGGGGTGAAATCCCGGGGCTCAACCTCGGAACTGCCTTTGATACTGCGAATCTTGAGTCCGATAGAGGTGGGTGGAATTCCTAGTGTA**
**CGTTATCCGGAATCATTGGGTTTAAAGGGTCCGTAGGCGGTCTTATAAGTCAGTGGTGAAAGCCCATCGCTCAACGATGGAACTGCCATTGATACTGTAAGACTTGAATGCTTAGGAAGTAACTAGAATATGTAGTGTAGC**
**CGTTAATCGGAATTACTGGGCGTAAAGCGTGCGCAGGCGGATATGTAAGTTAGGAGTGAAATCCCCGGGCTCAACCTGGGAATGGCTTTTAAGACTGCGTATCTAGAGTTTGTCAGAGGGGGGTGGAATTCCAAGTGTA**
**CGTTATCCGGAATTACTGGGCGTAAAGGGTGCGTAGGTGGTTTCTTAAGTCAGAGGTGAAAGGCTACGGCTCAACCGTAGTAAGCCTTTGAAACTGGGAAACTTGAGTGCAGGAGAGGAGAGTGGAATTCCTAGTGTAG**

```
phyloseq-class experiment-level object
otu_table()   OTU Table:         [ 2900 taxa and 39 samples ]
sample_data() Sample Data:       [ 39 samples by 29 sample variables ]
tax_table()   Taxonomy Table:    [ 2900 taxa by 6 taxonomic ranks ]
```

In [11]:
```
#save sequences for metagenomics
ps_bulk_clean_perc_metagenomics <- subset_samples(ps_bulk_clean_perc, !is.na(Metagenomes))
ps_bulk_clean_perc_metagenomics
#saveRDS(ps_bulk_clean_perc_metagenomics, "/SCIENCE/Nelson_lab/write-ups/EPseq_paper/revised/amplicon_data_for_metagenomes_deseq.rds")
```

```
phyloseq-class experiment-level object
otu_table()   OTU Table:         [ 2900 taxa and 8 samples ]
sample_data() Sample Data:       [ 8 samples by 29 sample variables ]
tax_table()   Taxonomy Table:    [ 2900 taxa by 6 taxonomic ranks ]
```

Look at how many samples we have after filtering

In [12]:
```
sample_count_filtered <- as.data.frame(sample_data(ps_bulk_blank_sig)) %>% group_by(Type, Location) %>% summarize(sample_count=length(SampleID))
sample_count_filtered <- dcast(sample_count_filtered, Location~Type, value.var="sample_count")
sample_count_filtered
```

Location	Bulk	Blank
WW_2ndary	6	NA
Chloramine	3	NA
MF	4	NA
NF-RO	2	NA
GAC	14	NA
SDS	8	NA
Amplification	NA	1
Extraction	NA	1

In [13]:
```
#collect samples that have metagenomics
ps_metagenome <- subset_samples(ps_nomock, subset = SampleID %in%
                                c("Mock1", "Mock2", "Neg1", "Neg2", "Neg3", "191a", "191b", "192", "32", "40", "61", "74", "16", "1","9","33","37","38","39","65"
,"66","67","185a","185b","187a","187b","193", "mock1", "mock2"))
ps_metagenome <- prune_samples(sample_sums(ps_metagenome)>=750, ps_metagenome)
ps_metagenome_perc <- transform_sample_counts(ps_metagenome, function(OTU) 100*OTU/sum(OTU))

#saveRDS(ps_metagenome_perc, file="/SCIENCE/Nelson_lab/data_files_nelson/el_paso_16S/dada2_full/phyloseq_metagenomic_samples.rds")
```

3. Rarefaction curves and alpha diversity

Rarefaction curves appear to plateau (note that singletons have been removed by DADA2). Singletons arising from sequencing errors can artificially inflate counts of observed taxa but removing them could underestimate richness. Given that curves mostly plateau, counts of observed ASVs may approximate richness despite different sequencing depths.

In [14]:
```
otu_bulk_clean <- as.data.frame(otu_table(ps_bulk_clean))

bulk_locations_colors <- c(colors[1],colors[2],colors[3],colors[4],colors[5],colors[10])
palette(c(colors[1],colors[2],colors[3],colors[4],colors[5],colors[10]))

options(repr.plot.width = 8, repr.plot.height = 5)

#pdf("/SCIENCE/Nelson_lab/write-ups/EPseq_paper/revised/figures/rarecurve_bulk_nolabels.pdf", height=6, width=8)
rarecurve(otu_bulk_clean, step=100, col=sample_data(ps_bulk_blank_sig)$Location,
          xlab="Reads", ylab="Amplicon Sequence Variants (ASVs)", lwd=2, ylim=c(0,1500), label=FALSE)

legend("topleft", legend=c("WW_2ndary", "Chloramine", "MF", "NF-RO", "GAC", "SDS"),
       col=bulk_locations_colors,
       lty=1, lwd=2)
#dev.off() #uncomment to save figure
```

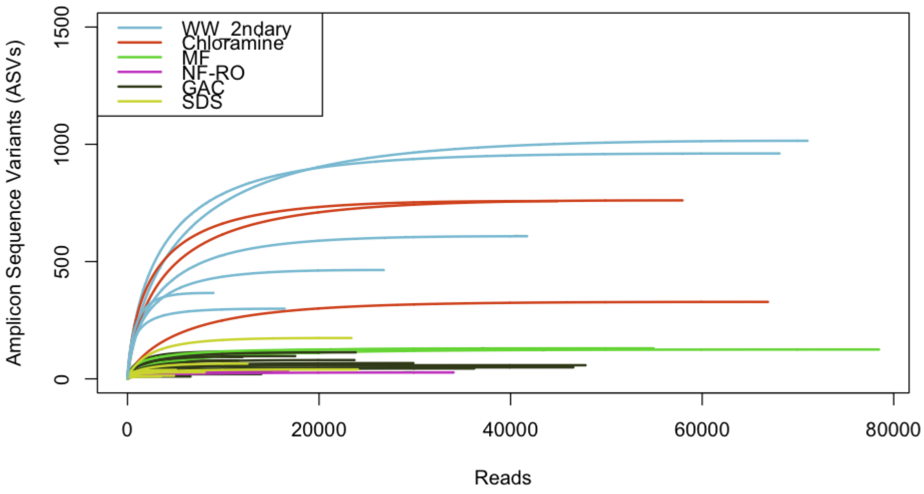

In [15]:
```
#count observed ASVs in bulk water samples and associated blanks

min(rowSums(otu_table(ps_bulk_clean))) #3499 (Amplification = 787 reads, Extraction = 350)
max(rowSums(otu_table(ps_bulk_clean))) #78462 reads

metadata_bulk <- as.data.frame(sample_data(ps_bulk_blank_sig))
metadata_bulk <- cbind(metadata_bulk, estimate_richness(ps_bulk_blank_sig, measures="Observed"))
metadata_bulk %>%
    select(Location, Observed) %>%
    group_by(Location) %>%
    summarize(min(Observed), max(Observed), mean(Observed), median(Observed))

#calculate log-fold change in observed ASVs between WW_2ndary and NF/RO perm
log10(49.75000/618.83333)#based on mean, ww2ndary vs SDS
```

3499

78462

```
Warning message in estimate_richness(ps_bulk_blank_sig, measures = "Observed"):
“The data you have provided does not have
any singletons. This is highly suspicious. Results of richness
estimates (for example) are probably unreliable, or wrong, if you have already
trimmed low-abundance taxa from the data.

We recommended that you find the un-trimmed data and retry.”
```

Location	min(Observed)	max(Observed)	mean(Observed)	median(Observed)
WW_2ndary	299	1015	618.83333	536.0
Chloramine	328	761	615.66667	758.0
MF	110	130	123.00000	126.0
NF-RO	27	34	30.50000	30.5
GAC	10	116	65.78571	65.0
SDS	10	174	49.75000	34.0
Amplification	2	2	2.00000	2.0
Extraction	3	3	3.00000	3.0

-1.09478061142145

Note: alpha diversity functions produce a warning about singletons having already been removed. This is because many alpha diversity calculations rely on singletons despite the fact that they may be due to sequencing errors. Singletons were removed as part of the DADA2 workflow. We proceed anyway, after reviewing rarefaction curves.

In [16]:
```
options(repr.plot.width = 6, repr.plot.height = 5) #for plotting size in jupyter

plot_richness(ps_bulk_clean, x="Name", measures="Observed", color="Location") +
theme_bw() +
scale_y_log10(breaks = trans_breaks("log10", function(x) 10^x),
                labels = trans_format("log10", math_format(10^.x))) +
theme(panel.background=element_blank(), panel.border=element_rect(color = "black", fill = NA),
      axis.text.x = element_text(angle = 90, hjust = 1))
#ggsave("/SCIENCE/Nelson_lab/data_files_nelson/el_paso_16S/figures_dada2/alpha_diversity_observed_seqs.pdf", device="pdf", width=16, height=10)
```

```
Warning message in estimate_richness(physeq, split = TRUE, measures = measures):
“The data you have provided does not have
any singletons. This is highly suspicious. Results of richness
estimates (for example) are probably unreliable, or wrong, if you have already
trimmed low-abundance taxa from the data.

We recommended that you find the un-trimmed data and retry.”
```

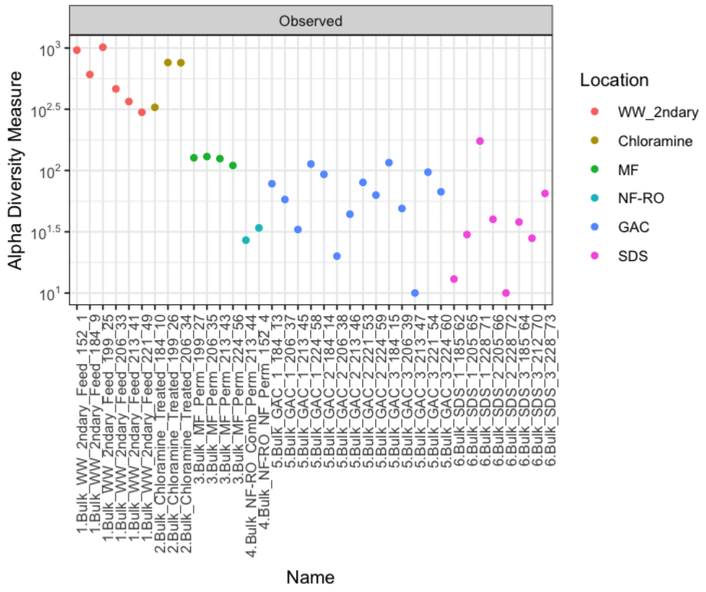

Plot alpha diversity as observed ASVs (Figure 2B)

In [17]:
```
#define function to put sample count above data
n_fun <- function(x){return(data.frame(y = max(x), label = paste0("n=",length(x))))}
#ps_bulk_sig <- subset_samples(ps_bulk_blank_sig, subset = Type %in% "Bulk")
options(repr.plot.width = 3.5, repr.plot.height = 2.75)
plot_richness(ps_bulk_clean, x="Location", measures=c("Observed"))+
    geom_boxplot()+
    theme(panel.background=element_blank(), panel.border=element_rect(color = "black", fill = NA),
     strip.background = element_blank(),
     strip.text.x = element_blank(),
     #axis.title.x = element_blank(),
     axis.text.x = element_text(angle = 45, hjust = 1, vjust = 1))+
    ylab("Observed ASVs") +
    scale_y_continuous(trans="log10", limits=c(10,1800), labels=trans_format("log10", math_format(10^.x)))+
    stat_summary(fun.data = n_fun, geom = "text", vjust=-1)+
    geom_vline(xintercept=5.5, linetype='solid')

#ggsave("/SCIENCE/Nelson_lab/write-ups/EPseq_paper/revised/figures/asv_observed_boxplot.svg", device="svg", width=3.5, height=2.75)
```

```
Warning message in estimate_richness(physeq, split = TRUE, measures = measures):
“The data you have provided does not have
any singletons. This is highly suspicious. Results of richness
estimates (for example) are probably unreliable, or wrong, if you have already
trimmed low-abundance taxa from the data.

We recommended that you find the un-trimmed data and retry.”
```

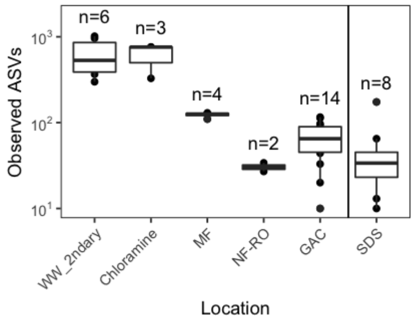

Plot flow cytometry cell counts for comparison to alpha diversity (Figure 2A)

In [18]:
```
#load FCM data
fcm_all <- read.table("/SCIENCE/Nelson_lab/data_files_nelson/el_paso_fcm/elpaso_fcm_all_071918.tsv", header=TRUE, sep="\t")
fcm_all <- fcm_all[, c("Location", "TCC_avg_cells_per_mL", "ICC_avg_cells_per_mL")]
fcm_all <- subset(fcm_all, fcm_all$Location %in% c('WW_2ndary', 'Chloramine', 'MF_combined', 'NF-RO_combined', 'GAC', 'SDS'))
fcm_all$Location <- factor(fcm_all$Location, levels=c('WW_2ndary', 'Ozone', 'Chloramine', 'MF_combined', 'NF-RO_combined', 'GAC', 'SDS'))
fcm_all_melt <- melt(fcm_all, id.vars = c("Location"), value.name = "Cells_per_mL", variable.name = "Assay")
n_fun <- function(x){return(data.frame(y = median(x), label = paste0("n=",length(x))))}

#make plot
options(repr.plot.width = 3.5, repr.plot.height = 2.75)
ggplot(fcm_all_melt, aes(x=Location, y=Cells_per_mL, color=Assay))+
    geom_boxplot()+
    scale_color_manual(values=c("darkgray", "black"))+
    #add sample size (just show for TCC bc it's the same for ICC)
    stat_summary(data = subset(fcm_all_melt, fcm_all_melt$Assay %in% c("TCC_avg_cells_per_mL")),
       fun.data = n_fun, geom = "text", vjust=-3, color='black')+
    scale_y_continuous(trans="log",
                       breaks=c(10, 100, 1000, 10000, 100000, 1000000, 10000000, 100000000, 1000000000),
                       labels=trans_format("log10", math_format(10^.x)),
                       limits=c(10,1000000000))+
    ylab("Cells per mL") +
    scale_x_discrete(labels= c('WW_2ndary', 'Chloramine', 'MF', 'NF-RO', 'GAC', 'SDS')) +
    theme(panel.background=element_blank(), panel.border=element_rect(color = "black", fill = NA),
         #axis.text.x = element_blank(),
        axis.text.x = element_text(angle = 45, hjust = 1, vjust = 1),
        #axis.title.x = element_blank(),
         legend.position="none")+
    geom_vline(xintercept=5.5, linetype='solid')+
    geom_hline(yintercept=12, linetype='dashed', color="darkgray")+
    geom_hline(yintercept=22, linetype='dashed', color="black")

#ggsave("/SCIENCE/Nelson_lab/write-ups/EPseq_paper/revised/figures/fcm_boxplot.svg", device="svg", width=3.5, height=2.75)
```

```
Warning message:
“Removed 7 rows containing non-finite values (stat_boxplot).”Warning message:
“Removed 2 rows containing non-finite values (stat_summary).”
```

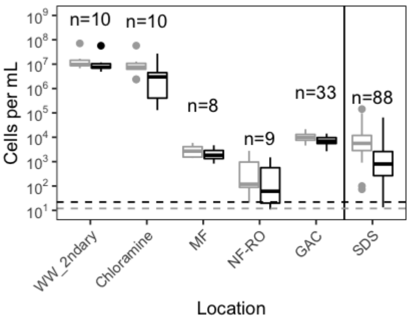

In [19]:
```
fcm_all %>% group_by(Location) %>% na.omit() %>% summarize(median(ICC_avg_cells_per_mL))
#metadata %>% group_by(Type, Location) %>% summarize(sample_count=length(SampleID))
log10(6933/760)
```

Location	median(ICC_avg_cells_per_mL)
WW_2ndary	7495000
Chloramine	3010666
MF_combined	1853
NF-RO_combined	22
GAC	6933
SDS	760

0.96010760791798

4. Taxonomic composition

In [20]:
```
## Plot bar and heatmap
ps_bulk_blank_sig_perc_filt2 <- filter_taxa(ps_bulk_blank_sig_perc, filterfun(kOverA(1, 2)), TRUE) #one sample, 2 perc.
options(repr.plot.width = 12, repr.plot.height = 5)
#playing with the colors a bit:
colors2 <- colors[-c(2,3,4)]
colors2[8] <- colors[2]

plot_bar(ps_bulk_blank_sig_perc_filt2, x="combined_name", fill="Family") + scale_fill_manual(values = colors2) +
  theme(panel.background=element_blank(), panel.border=element_rect(color = "black", fill = NA),
        axis.text.x = element_text(angle = 45, hjust = 1, vjust = 1, size=9),
        axis.text.y = element_text(size=9),
        legend.text=element_text(size=9),
        legend.title=element_text(size=9))+
    facet_grid(.~Location, scale="free_x", space="free_x")+
ylab("Percent relative abundance") +
xlab("Sample name")+
guides(fill=guide_legend(ncol=2))

#ggsave("/SCIENCE/Nelson_lab/write-ups/EPseq_paper/revised/figures/tax_barplot_min2perc.pdf", device="pdf", width=12, height=6)

options(repr.plot.width = 9, repr.plot.height = 6.5)
plot_heatmap(ps_bulk_blank_sig_perc_filt2, taxa.label = "Family", sample.label = "combined_name", sample.order = "Name", method="NMDS",
        low="white", high="red", na.value="black", trans = log_trans(10))+
     theme(axis.text.y = element_text(size=7),
           axis.title.y = element_blank(),
           axis.ticks = element_blank(),
           axis.text.x = element_text(angle = 45, hjust = 1, vjust = 1, size=9))+
     facet_grid(.~Location, scale="free_x", space="free_x")

#ggsave("/SCIENCE/Nelson_lab/write-ups/EPseq_paper/revised/figures/tax_heatmap_min2perc.pdf", device="pdf", width=8, height=6.5)
```

```
Warning message:
“Transformation introduced infinite values in discrete y-axis”
```

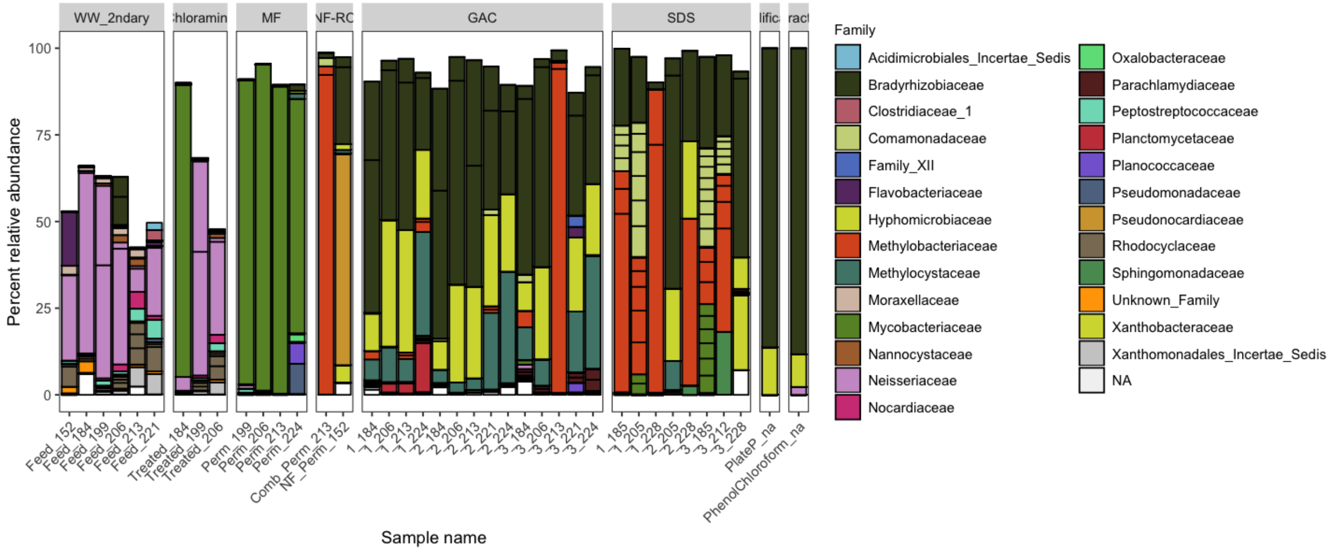

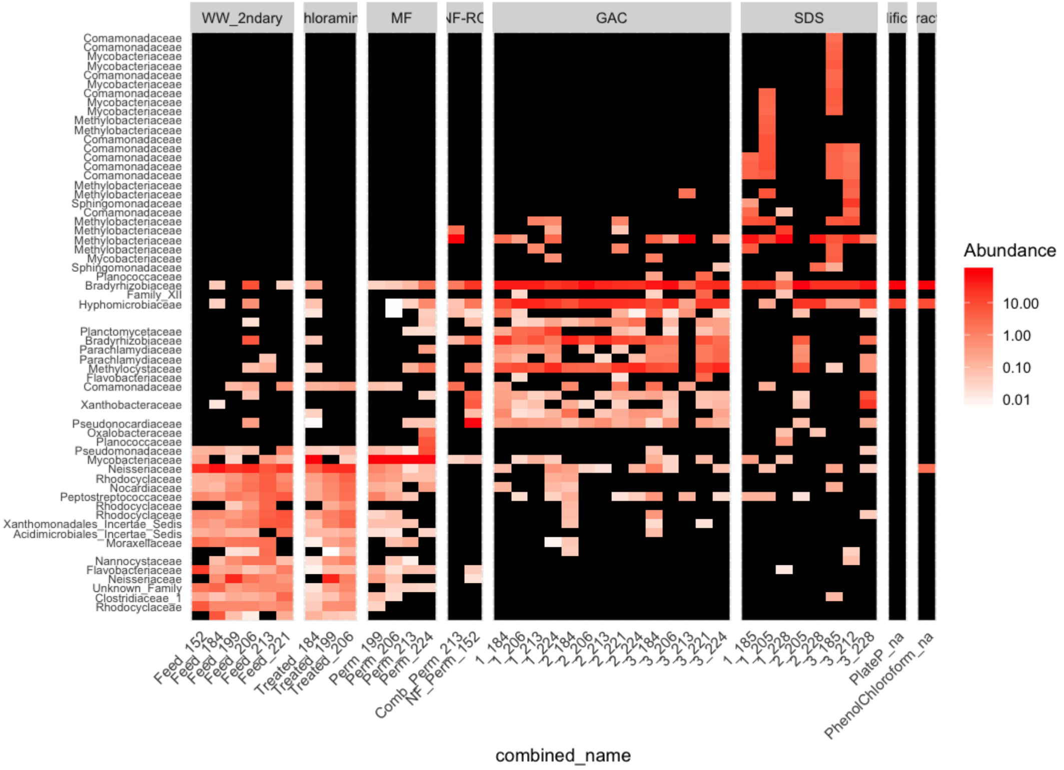

In [21]:
```
ps_bulk_blank_sig_perc_filt2
tax_table(ps_bulk_blank_sig_perc_filt2) #get names for Family_XII and Unknown_Family in above figure
```

```
phyloseq-class experiment-level object
otu_table()   OTU Table:         [ 64 taxa and 39 samples ]
sample_data() Sample Data:       [ 39 samples by 29 sample variables ]
tax_table()   Taxonomy Table:    [ 64 taxa by 6 taxonomic ranks ]
```

CGTTGTCCGGAATTACTGGGCGTAAAGAGCTCGTAGGTGGTTTGTCACGTTGTCCGTGAAAACTCACAGCTTAACTGTGGGCGTGCGGGCGATACGGGCAGACTGGAGTACTGTAGGGGAGACTGGAATTCCTGGTGT
CGTTGCTCGGAATCACTGGGCGTAAAGGGCGCGTAGGCGGCGTTTTAAGTCGGGGGTGAAAGCCTGTGGCTCAACCACAGAATGGCCTTCGATACTGGGACGCTTGAGTATGGTAGAGGTTGGTGGAACTGCGAGTG
CGTTGCTCGGAATCACTGGGCGTAAAGGGTGCGTAGGCGGGTCTTTAAGTCAGGGGTGAAATCCTGGAGCTCAACTCCAGAACTGCCTTTGATACTGAAGATCTTGAGTTCGGGAGAGGTGAGTGGAACTGCGAGTGT
CGTTAATCGGAATTACTGGGCGTAAAGCGTGCGCAGGCGGATATGTAAGTTAGAAGTGAAATCCCCGGGCTCAACCTGGGAATGGCTTTTAAGACTGCGTATCTAGAGTTTGTCAGAGGGGGGTGGAATTCCAAGTGTA
CGTTGTTCGGAATCACTGGGCGTAAAGCGCACGTAGGCGGATTTGTAAGTCAGGGGTGAAATCCCGGGGCTCAACCTCGGAACTGCCTTTGATACTGCGAATCTTGAGTCCGATAGAGGTGGGTGGAATTCCTAGTGTA
CGTTATCCGGAATCATTGGGTTTAAAGGGTCCGTAGGCGGTCTTATAAGTCAGTGGTGAAAGCCCATCGCTCAACGATGGAACTGCCATTGATACTGTAAGACTTGAATGCTTAGGAAGTAACTAGAATATGTAGTGTAGC
CGTTGTTCGGAATCACTGGGCGTAAAGCGCACGTAGGCGGATCTTTAAGTCAGGGGTGAAATCCCGAGGCTCAACCTCGGAACTGCCTTTGATACTGGAGGTCTCGAGTCCGGGAGAGGTGAGTGGAACTGCGAGTG
CGTTGCTCGGAATCACTGGGCGTAAAGGGTGCGTAGGCGGGTCTTTAAGTCAGAGGTGAAAGCCTGGAGCTCAACTCCAGAACTGCCTTTGATACTGAGGATCTCGAGTTCGGGAGAGGTGAGTGGAACTGCGAGTG
CGTTGTCCGGAATTATTGGGCGTAAAGAGCTCGTAGGCGGTCTGTCGCGTCGGTCGTGAAAACCTGCAGCTTAACTGTGGGCTTGCGGTCGATACGGGCATGACTGGAGTTCGGCAGGGGAGACTGGAATTCCTGGT
CGTTAATCGGAATTACTGGGCGTAAAGCGTGCGCAGGCGGATATGTAAGTTAGGAGTGAAATCCCCGGGCTCAACCTGGGAATGGCTTTTAAGACTGCGTATCTAGAGTTTGTCAGAGGGGGGTGGAATTCCAAGTGTA
CGTTAATCGGAATTACTGGGCGTAAAGCGTGCGCAGGCGGTTTTGTAAGACAGAGGTGAAATCCCCGGGCTCAACCTGGGAACTGCCTTTGTGACTGCAAGGCTGGAGTGCGGCAGAGGGGGATGGAATTCCGCGTG
CGTTGCTCGGAATCACTGGGCGTAAAGGGCGCGTAGGCGGCGTTTTAAGTCGGGGGTGAAAGCCTGTGGCTCAACCACAGAATGGCCTTCGATACTGGGACGCTTGAGTCTGGTAGAGGTTGGTGGAACTGCGAGTG
CGTTGCTCGGAATCACTGGGCGTAAAGCGCACGTAGGCGGGTCGTTAAGTCAGAGGTGAAAGCCTGGAGCTCAACTCCAGAACTGCCTTTGATACTGGCGACCTCGAGTTCGAGAGAGGTTGGTGGAACTCCGAGTG
CGTTGCTCGGAATCACTTGGCGTAAAGGGCGCGTAGGCGGCGTTTTAAGTCGGGGGTGAAAGCCTGTGGCTCAACCACAGAATGGCCTTCGATACTGGGACGCTTGAGTATGGTAGAGGTTGGTGGAACTGCGAGTG
CGTTGTTCGGAATCACTGGGCTTAAAGGGCGCGTAGGCGGGCCGCCAAGTCCGGGGTGAAAGCCTCCGGCTCAACCGGAGAATTGCCTGGGATACTGGTGGTCTTGAGGGAGGTAGGGGCACGCGGAACTTCCGG
TGTTACTCATCAATACTGGGCGTAAAGCGCATGTAGGCGGTTTATGATGTCAGAATAAAAATCTAGAGATCACCTTTATATTAATATTTTTAAACTAATAAACTAAGAGTATAAGAGAAGTGTGTTGCACTTTCAGTGGAGAGG
CGTTAATCGGAATTACTGGGCGTAAAGCGTGCGCAGGCGGACTTTTAAGCCAGATGTGAAAGCCCCGAGCTTAACTTGGGAATTGCGTTTGGAACTGGGAGTCTAGAGTCTGTCAGAGGGGGATGGAATTCCACGTGT
CGTTATCCGGAATTACTGGGCGTAAAGGGTGCGTAGGTGGTTTCTTAAGTCAGAGGTGAAAGGCTACGGCTCAACCGTAGTAAGCCTTTGAAACTGGGAAACTTGAGTGCAGGAGAGGAGAGTGGAATTCCTAGTGTAG
CGTTAATCGGAATTACTGGGCGTAAAGCGTGCGCAGGCGGTTTGCTAAGACAGGTGTGAAATCCCCGGGCTTAACCTGGGAACTGCGCTTGTGACTGGCAGGCTAGAGTACGGCAGAGGGGGGTGGAATTCCTGGTG
CGTTAATCGGAATTACTGGGCGTAAAGCGTGCGCAGGCGGTTTTGTAAGACAGATGTGAAATCCCCGGGCTTAACCTGGGAACTGCGTTTGTGACTGCAAGGCTAGAGTACGGCAGAGGGGGGTGGAATTCCTGGTGT
CGTTATTCGGAATTATTGGGCGTAAAGAGTGCGTAGGCGGTATGATAAGTCTGTTGTTAAATCTCCTGGCTCAACCAGGAAAATGCAATGGAAACTATTGTTCTAGAGGGTGAGAGAGAGAAGTGGAATTCCAGGAGTTAG
CGTTAATCGGAATTACTGGGCGTAAAGCGCGTGTAGGCGGTTGGCTAAGTCAGATGTGAAAGCCCCGGGCTCAACCTGGGAATTGCATTTGATACTGGCTGGCTAGAATGTGGTAGAGGGAGGCGGAATTCCCGGTGTA
CGTTGTCCGGAATTACTGGGCGTAAAGAGCTCGTAGGCGGTTTGTCGCGTCGTCTGTGAAATTCTGCAGCTTAACTGTAGGCGTGCAGGCGATACGGGCAGACTTGAGTACTACAGGGGAGACTGGAATTCCTGGTGT
CGTTAATCGGAATTACTGGGCGTAAAGCGCGCGTAGGCGGTTTGTTAAGTCGGATGTGAAATCCCTGGGCTCAACCTAGGCACTGCATCCGATACTGGCTAACTAGAGTGTGGGAGAGGAAGGTAGAATTCCAGGTGTA
CGTTAATCGGAATTACTGGGCGTAAAGCGTGCGCAGGCGGTGATGTAAGACAGATGTGAAATCCCCGGGCTCAACCTGGGAACTGCGTTTGTGACTGCATCACTCGAGTACGGCAGAGGGAGGTGGAATTCCGCGTG
CGTTAATCGGAATTACTGGGCGTAAAGCGCGCGTAGGTGGTTCAGCAAGTTGGAGGTGAAATCCCCGGGCTCAACCTGGGAACTGCCTCCAAAACTACTGAGCTAGAGTACGGTAGAGGGTAGTGGAATTTCCTGTGT
CGTTGTCCGGAATTACTGGGCGTAAAGAGCTCGTAGGTGGTTTGTCGCGTTGTTCGTGAAAACTCACAGCTTAACTGTGGGCGTGCGGGCGATACGGGCAGACTAGAGTACTGCATGGGAGACTGGAATTCCTGGTGT
CGTTGCTCGGAATCACTGGGCGTAAAGGGCGCGTAGGCGGCGTTTTAAGTCGGGGGTGAAAGCCTGTGGCTCAACCACAGAATGGCCTTCGATACTTGGACGCTTGAGTATGGTAGAGGTTGGTGGAACTGCGAGTG
TGTTATTCGTCTTAATGGGTGTAAAACGCGTGTAGATGGGATATCGAGTTTAACTTTAAATTTTTATAATATATTATAAAAAAAGTTTTAATACAGTTATTCTTGAGTGCTTATAAAACAACAGTATTTTAAGTGTAAGATGACATTT
CGTTATCCGGAATGATTGGGCGTAAAGCGTCTGTAGGTGGTTTGACAAGTCTCCTGTCAAAGATCAGGGCTTAACCCTGGGCCGGCAGGAGAAACTATCAGGCTAGAGTTCGGTAGGGGCAGAGGGAATTCCCGGTGG
⋮
CGTTGTCCGGAATTACTGGGCGTAAAGAGCTCGTAGGTGGTTTGTCGCGTTGTTCGTGAAAACTCACAGCTTAACTGTGGGCGTGCGGGCGATACTGGCAGACTAGAGTACTGCAGGGGAGACTGGAATTCCTGGTGT
CGTTGTCCGGATTTATTGGGCGTAAAGAGCTCGTAGGCGGTTCCGTAAGTCGAGTGTGAAAAATCTGGGCTCAACCCAGTGAAGCACTCGATACTGCGGTGACTAGAGTACGGTAGAGGAGTGTGGAATTTGTGGTGTA
CGTTGTCCGGAATTATTGGGCGTAAAGCGCGCGCAGGCGGCCTCTTAAGTCTGATGTGAAAGCCCCCGGCTCAACCGGGGAGGGCCATTGGAAACTGGGAGGCTTGAGTATAGGAGAGAAGAGTGGAATTCCACGTG
CGTTATTCGGAATTATTGGGCGTAAAGGGTGTGCAGACGGTTTGTTAAGTCACTTGTTAAATTCTCCGGCTTAACCGGAGGCCAGCATGTGAAACTAGCAGACTAGAGGGTGAGAGAGAGAAGTGGAATTCTCGGAGTA
CGTTGCTCTGAATCACTGGGCGTAAAGGGCGCGTAGGCGGCGTTTTAAGTCGGGGGTGAAAGCCTGTGGCTCAACCACAGAATGGCCTTCGATACTGGGACGCTTGAGTATGGTAGAGGTTGGTGGAACTGCGAGTG
CGTTGTCCGGAATTATTGGGCGTAAAGCGCGCGCAGGCGGTCCCTTAAGTCTGATGTGAAAGCCCACGGCTCAACCGTGGAGGGTCATTGGAAACTGGGGGACTTGAGTGCAGAAGAGGAAAGTGGAATTCCATGTG
CGTTGTCCGGATTTACTGGGCGTAAAGGGAGCGTAGGCGGACTTTTAAGTGAGATGTGAAATACCCGGGCTCAACTTGGGTGCTGCATTTCAAACTGGAAGTCTAGAGTGCAGGAGAGGAGAATGGAATTCCTAGTGTA
CGTTGCTAGGAATCACTGGGCGTAAAGGGCGCGTAGGCGGCGTTTTAAGTCGGGGGTGAAAGCCTGTGGCTCAACCACAGAATGGCCTTCGATACTGGGACGCTTGAGTATGGTAGAGGTTGGTGGAACTGCGAGTG
CGTTAATCGGAATTACTGGGCGTAAAGCGTGCGCAGGCGGCTTTGCAAGACAGAGGTGAAATCCCCGGGCTCAACCTGGGAACTGCCTTTGTGACTGCAAGGCTAGAGTACGGCAGAGGGGGATGGAATTCCGCGTG
CGTTAATCGGAATTACTGGGCGTAAAGCGTGCGCAGGCGGCTTTGCAAGACAGAGGTGAAATCCCAGGGCTCAACCTGGGAACTGCCTTTGTGACTGCAAGGCTAGAGTACGGCAGAGGGGGATGGAATTCCGCGTG
CGTTAATCGGAATTACTGGGCGTAAAGCGTGCGCAGGCGGTTATGTAAGACAGATGTGAAATCCCCGGGCTCAACCTGGGAACTGCGTTTGTGACTGCATAACTAGAGTACGGCAGAGGGAGGTGGAATTCCGCGTGT
CGTTAATCGGAATTACTGGGCGTAAAGCGTGCGCAGGCGGCTTTGCAAGACAGAGGTGAAATCCCCGGGCTCAACCTGGGAACTGCCTTTGTGACTGCAAGGCTAGAGTACGGCAGATGGGGATGGAATTCCGCGTG
CGTTAATCGGAATTACTGGGCGTAAAGCGTGCGCAGGCGGTTTTGTAAGTCTGTCGTGAAAGCCCCGGGCTCAACCTGGGAATTGCGATGGAGACTGCAAGGCTTGAATCTGGCAGAGGGGGGTAGAATTCCACGTGT
CGTTAATCGGAATTACTGGGCGTAAAGCGTGCGCAGGCGGCTTTGCAAGACAGATGTGAAATCCCCGGGCTCAACCTGGGAACTGCATTTGTGACTGCAAGGCTAGAGTACGGCAGAGGGGGATGGAATTCCGCGTG
CGTTAATCGGAATTACTGGGCGTAAAGCGTGCGCAGGCGGTGATGTAAGACAGATGTGAAATCCCCGGGCTCAACCTGGGAACTGCATTTGTGACTGCATCGCTGGAGTGCGGCAGAGGGGGATGGAATTCCGCGTG
CGTTAATCGGAATTACTGGGCGTAAAGCGTGCGCAGGCGGCTTTGCAAGACAGAGGTGAAATCCACGGGCTCAACCTGGGAACTGCCTTTGTGACTGCAAGGCTAGAGTACGGCAGAGGGGGATGGAATTCCGCGTG
CGTTGTCCGGAATTATTGGGCGTAAAGCGCGCGCAGGCGGTCCTTTAAGTCTGATGTGAAAGCCCACGGCTCAACCGTGGAGGGTCATTGGAAACTGGGGGACTTGAGTGCAGAAGAGGAAAGTGGAATTCCACGTG
CGTTGTCCGGAATTACTGGGCGTAAAGAGCTCGTAGGTGGTTTGTCGCGTTGTTCGTGAAAACTCACAGCTTAACTGTGGGCGTGCTGGCGATACGGGCAGACTAGAGTACTGCAGGGGAGACTGGAATTCCTGGTGT
CGTTAATCGGAATTATTGGGCGTAAAGGGTGAGTAGGTGGTTTGATAAGATAGCTGTGAAAGCCCCAGGCTTAACCTGGGAAAGTCAGCTGTGACTGTGAGACTAGAGTCTAGGAGAGGGTAGTGGAATTTCCGGTGTAG
CGTTATCCGGATTTATTGGGTTTAAAGGGTCCGTAGGCGGATCTGTAAGTCAGTGGTGAAATCTCATAGCTTAACTATGAAACTGCCATTGATACTGCAGGTCTTGAGTAAAGTAGAAGTGGCTGGAATAAGTAGTGTAGCG
CGTTGTCCGGAATTACTTGGCGTAAAGAGCTCGTAGGTGGTTTGTCGCGTTGTTCGTGAAAACTCACAGCTTAACTGTGGGCGTGCGGGCGATACGGGCAGACTAGAGTACTGCAGGGGAGACTGGAATTCCTGGTGT
CGTTAATCGGAATTACTGGGCGTAAAGCGTGCGCAGGCGGCTTTGCAAGACAGAGGTGAAATCCCCGGGCTCAACCTGGGAACTGCCTTTGTGACTGCAAGGCTAGAGTACGGCAGAGGGGGATGGAATTCCGCGTG
CGTTAATCGGAATTACTGGGCGTAAAGCGTGCGCAGGCGGTTATGTAAGACAGATGTGAAATCCCCGGGCTCAACCTGGGAACTGCATTAGTGACTGCATAGCTGGAGTACGGCAGAGGTGGATGGAATTCCGCGTGTA
CGTTGTCCGGAATTACTGGGCGTAAAGAGCTCGTAGGTGGTTTGTCGCGTTGTTCGTGAAAACTCACAGCTTAACTGTTGGCGTGCGGGCGATACGGGCAGACTAGAGTACTGCAGGGGAGACTGGAATTCCTGGTGT
CGTTGCTCGGAATCACTGGGCGTAAAGGGCGCGTAGGCGGCGTTTTAAGTCGGGGGTGAAAGCCTGTGGCTCAACCACAGAATGGCATTCGATACTGGGACGCTTGAGTATGGTAGAGGTTGGTGGAACTGCGAGTG
CGTTATTCGGAATTACTGGGCGTAAAGCGCACGTAGGCGGCTTTGTAAGTTAGAGGTGAAAGCCTGGAGCTCAACTCCAGAACTGCCTTTAAGACTGCATCGCTTGAATCCAGGAGAGGTGAGTGGAATTCCGAGTGTA
CGTTGCTCGGAATCACTGGGCGTAAAGGGCGCGTAGGCGGCGTTTTAAGTCGGGGGTGAAAGCCTGTGGCTAAACCACAGAATGGCCTTCGATACTGGGACGCTTGAGTATGGTAGAGGTTGGTGGAACTGCGAGTG
CGTTGTCCGGAATTACTGGGCGTAAAGAGCTCGTAGGTGGTTTGTCGCGTTGTTCGTGAAAACTCACAGCTTAACTGTGGGCGTGCGGGCGATACGGGCAGACTAGAGTACTGCAGGGGAGACTGGAATTCCTGGTGT
CGTTAATCGGAATTACTGGGCGTAAAGCGTGCGCAGGCGGTTATGTAAGACAGATGTGAAATCCCAGGGCTCAACCTGGGAACTGCATTAGTGACTGCATAGCTGGAGTACGGCAGAGGGGGATGGAATTCCGCGTGTA
CGTTAATCGGAATTACTGGGCGTAAAGCGTGCGCAGGCGGTTATGTAAGACAGATGTGAAATCCACGGGCTCAACCTGGGAACTGCATTAGTGACTGCATAGCTGGAGTACGGCAGAGGGGGATGGAATTCCGCGTGTA

In [22]:
```
#relative abundance of Neisseriaceae in WW_2ndary (plant feed)
bulk_neisseriaceae <- subset_taxa(ps_bulk_blank_sig_perc, Family=="Neisseriaceae")
bulk_neisseriaceae <- subset_samples(bulk_neisseriaceae, subset=Location %in% "WW_2ndary")
rowSums(otu_table(bulk_neisseriaceae))
```

**1**	26.0076972795111
**25**	57.1090514691382
**33**	36.4227763861368
**41**	8.87996430165105
**49**	21.4381474710542
**9**	54.0222445946594

In [23]:
```
##making supplementary table with same data as heatmap plus more
#get otu table and taxonomy table
ps_bulk_blank_sig_perc_filt1 <- filter_taxa(ps_bulk_blank_sig_perc, filterfun(kOverA(1, 1)), TRUE) #one sample, 2 perc.

otu.tab <- as.data.frame(otu_table(ps_bulk_blank_sig_perc_filt1))
tax.tab <- as.data.frame(tax_table(ps_bulk_blank_sig_perc_filt1))

#add sample names to otu table
sample_names <- subset(sample_data(ps_bulk_blank_sig_perc_filt1), select = c("longer_name"))
otu.tab <- merge(sample_names, otu.tab, all.y = TRUE, by=0)
row.names(otu.tab) <- otu.tab$longer_name
otu.tab <- t(subset(otu.tab, select = c(-Row.names, -longer_name)))
otu.tab <- as.data.frame(otu.tab) #convert back from matrix to df

#add taxonomy to otu table
otu.tab <- merge(tax.tab, otu.tab, by=0)
row.names(otu.tab) <- otu.tab$Row.names
otu.tab <- subset(otu.tab, select = -Row.names)

head(otu.tab)
#write.table(otu.tab, '/SCIENCE/Nelson_lab/write-ups/EPseq_paper/revised/otu_tab_min1perc.tsv', sep='\t', quote=FALSE)
```

**CATTAATCGGATTTATTGGGCGTAAAGGGCGCGTAGGCGGGAAAGTCAGTCAGATGTGAAAGCCCAGGGCTCAACCCTGGAACAGCATTTGAAACTACTTTTCTAGAGGGTAGGCGGAGAAAACAGAATTCCACGTGTA**
**CATTAATCGGATTTATTGGGCGTAAAGGGCGCGTAGGCGGGAAAGTCAGTCAGATGTGAAAGCCCAGGGCTCAACCCTGGAACAGCATTTGAAACTACTTTTCTAGAGGGTAGGCGGAGAAAACAGAATTCCACGTGTA**
**CGTTAATCGGAATTACTGGGCGTAAAGCGCGCGTAGGCGGTTTGTTAAGTCGGATGTGAAATCCCTGGGCTCAACCTAGGCACTGCATCCGATACTGGCTAACTAGAGTGTGGGAGAGGAAGGTAGAATTCCAGGTGTA**
**CGTTAATCGGAATTACTGGGCGTAAAGCGCGCGTAGGCGGTTTGTTAAGTCGGATGTGAAATCCCTGGGCTCAACCTAGGCACTGCATCCGATACTGGCTGACTAGAGTGTGGGAGAGGAAGGTAGAATTCCAGGTGTA**
**CGTTAATCGGAATTACTGGGCGTAAAGCGCGCGTAGGTGGTTCAGCAAGTTGGAGGTGAAATCCCCGGGCTCAACCTGGGAACTGCCTCCAAAACTACTGAGCTAGAGTACGGTAGAGGGTAGTGGAATTTCCTGTGTA**
**CGTTAATCGGAATTACTGGGCGTAAAGCGCGTGTAGGCGGTTGGCTAAGTCAGATGTGAAAGCCCCGGGCTCAACCTGGGAATTGCATTTGATACTGGCTGGCTAGAATGTGGTAGAGGGAGGCGGAATTCCCGGTGTA**

NF-RO vs. blank:

In [24]:
```
#subset blank and bulk, then NF-RO and amplification blank (only one remains after filtering)
ps_NFRO_blank_perc <- subset_samples(ps_bulk_blank_sig_perc, subset = Location %in% c("NF-RO", "Amplification", "Extraction")) #subset just that location

ps_NFRO_blank_perc <- filter_taxa(ps_NFRO_blank_perc, filterfun(kOverA(1, 0)), TRUE) #keep only ASVs that had reads in bulk/blanks

ps_NF_RO_filt <- filter_taxa(ps_NFRO_blank_perc, filterfun(kOverA(1, 0.1)), TRUE) #filter for ASVs > 5% relative abundance

sample_data(ps_NF_RO_filt)$Description <- factor(sample_data(ps_NF_RO_filt)$Description,
                                                 levels=c('PlateP', 'PhenolChloroform','NF_Perm', 'Comb_Perm'))

options(repr.plot.width = 3.5, repr.plot.height = 4)
plot_heatmap(ps_NF_RO_filt, taxa.label = "Family", sample.label = "Description",
             sample.order = c('Neg1', '61','4', '44'),
             low="white", high="red", na.value="black", trans = log_trans(10))

ggsave("/SCIENCE/Nelson_lab/write-ups/EPseq_paper/revised/figures/NF-RO_heatmap_min0.1perc.png", device="png", width=3.5, height=4)

options(repr.plot.width = 4, repr.plot.height = 4)
plot_bar(ps_NF_RO_filt, x="Description", fill="Family") + scale_fill_manual(values = colors) +
    ylab("Percent relative abundance") +
    xlab("Sample name")+
    theme(panel.background=element_blank(), panel.border=element_rect(color = "black", fill = NA),
          axis.text.x = element_text(angle = 90, hjust = 1, vjust = .5),
          axis.text.y = element_text(size=12),
          axis.title=element_text(size=12),
          legend.text=element_text(size=9),
          legend.title=element_text(size=9))
#ggsave("/SCIENCE/Nelson_lab/write-ups/EPseq_paper/revised/figures/NF-RO_barplot_min0.1perc.png", device="png", width=4, height=4)
```

```
Warning message in metaMDS(veganifyOTU(physeq), distance, ...):
“stress is (nearly) zero: you may have insufficient data”Warning message in postMDS(out$points, dis, plot = max(0, plot - 1), ...):
“skipping half-change scaling: too few points below threshold”Warning message:
“Transformation introduced infinite values in discrete y-axis”

Warning message:
“Transformation introduced infinite values in discrete y-axis”
```

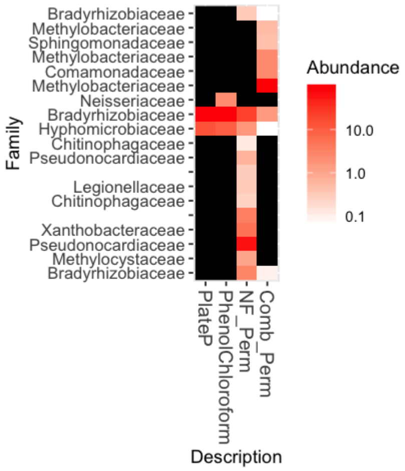

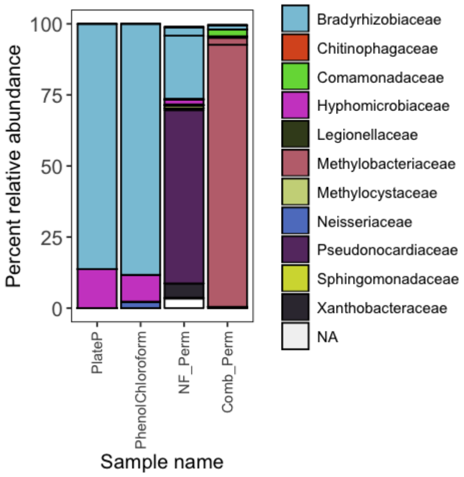

In [25]:
```
#make readable OTU table to get relative abundance values directly
NF_RO_otus <- as.data.frame(otu_table(ps_NF_RO_filt))
NF_RO_otus <- as.data.frame(t(NF_RO_otus))
NF_RO_taxa <- as.data.frame(tax_table(ps_NF_RO_filt) [, "Family"])
NF_RO_seq_info <- merge(NF_RO_otus, NF_RO_taxa, by=0)

colnames(NF_RO_seq_info) <- c("sequence", "NF-RO combined", "NF perm", "PhenolChloroform", "Amplification", "Family")
NF_RO_seq_info
```

sequence
CGTTAATCGGAATTACTGGGCGTAAAGCGTGCGCAGGCGGATATGTAAGTTAGAAGTGAAATCCCCGGGCTCAACCTGGGAATGGCTTTTAAGACTGCGTATCTAGAGTTTGTCAGAGGGGGGTGGAATTCCAAGTGTAGCAGT
CGTTAATCGGAATTACTGGGCGTAAAGCGTGCGCAGGCGGTTTTGTAAGACAGAGGTGAAATCCCCGGGCTCAACCTGGGAACTGCCTTTGTGACTGCAAGGCTGGAGTGCGGCAGAGGGGGATGGAATTCCGCGTGTAGC
CGTTAATCGGAATTACTGGGCGTAAAGCGTGCGTAGGTGGTTGATTAAGTTATCTGTGAAATCCCTGGGCTTAACCTGGGCAGGTCAGATGATACTGGTTGACTCGAGTATGGGAGAGGGTAGTGGAATTTCCGGTGTAGCGGT
CGTTATCCGGATTCACTGGGTTTAAAGGGTGCGTAGGCGGGCATGTAAGTCAGTGGTGAAATCTTTGGGCTTAACCCGAAAACTGCCATTGATACTATATGTCTTGAATATTGTGGAGGTTAGCGGAATATGTCATGTAGCGGTGAA
CGTTATCCGGATTCACTGGGTTTAAAGGGTGCGTAGGCGGGCTGTTAAGTCAGTGGTGAAATCTTTGGGCTTAACCCGAAAACTGCCATTGATACTATCAGTCTTGAATATTGTGGAGGTCTGCGGAATATGTCATGTAGCGGTGA
CGTTGCTCGGAATCACTGGGCGTAAAGCGCACGTAGGCGGGTCGTTAAGTCAGAGGTGAAAGCCTGGAGCTCAACTCCAGAACTGCCTTTGATACTGGCGACCTCGAGTTCGAGAGAGGTTGGTGGAACTCCGAGTGTAGA
CGTTGCTCGGAATCACTGGGCGTAAAGGGCGCGTAGGCGGCGTTTTAAGTCGGGGGTGAAAGCCTGTGGCTCAACCACAGAATGGCCTTCGATACTGGGACGCTTGAGTATGGTAGAGGTTGGTGGAACTGCGAGTGTAGA
CGTTGCTCGGAATCACTGGGCGTAAAGGGCGCGTAGGCGGCGTTTTAAGTCGGGGGTGAAAGCCTGTGGCTCAACCACAGAATGGCCTTCGATACTGGGACGCTTGAGTCTGGTAGAGGTTGGTGGAACTGCGAGTGTAGA
CGTTGCTCGGAATCACTGGGCGTAAAGGGCGCGTAGGCGGCGTTTTAAGTCGTGGGTGAAAGCCTGTGGCTCAACCACAGAATGGCCTTCGATACTGGGACGCTTGAGTATGGTAGAGGTTGGTGGAACTGCGAGTGTAGA
CGTTGCTCGGAATCACTGGGCGTAAAGGGTGCGTAGGCGGGTCTTTAAGTCAGAGGTGAAAGCCTGGAGCTCAACTCCAGAACTGCCTTTGATACTGAGGATCTCGAGTTCGGGAGAGGTGAGTGGAACTGCGAGTGTAGA
CGTTGCTCGGAATCACTGGGCGTAAAGGGTGCGTAGGCGGGTCTTTAAGTCAGGGGTGAAATCCTGGAGCTCAACTCCAGAACTGCCTTTGATACTGAAGATCTTGAGTTCGGGAGAGGTGAGTGGAACTGCGAGTGTAGAG
CGTTGCTCGGAATCACTGGGCGTAAAGGGTGCGTAGGCGGGTCTTTAAGTCAGGGGTGAAATCCTGGAGCTCAACTCCAGAACTGCCTTTGATACTGAGGATCTTGAGTCCGGAAGAGGTGAGTGGAACTGCGAGTGTAGAG
CGTTGTCCGGAATTATTGGGCGTAAAGAGCTCGTAGGCGGTCTGTCGCGTCGGTCGTGAAAACCTGCAGCTTAACTGTGGGCTTGCGGTCGATACGGGCATGACTGGAGTTCGGCAGGGGAGACTGGAATTCCTGGTGTAGC
CGTTGTCCGGAATTATTGGGCGTAAAGAGCTCGTAGGCGGTCTGTCGCGTCGGTCGTGAAAACTTGGGGCTTAACTCTGAGCTTGCGGTCGATACGGGCATGACTGGAGTTCGGCAGGGGAGACTGGAATTCCTGGTGTAGC
CGTTGTTCGGAATCACTGGGCGTAAAGCGCACGTAGGCGGATCTTTAAGTCAGGGGTGAAATCCCGAGGCTCAACCTCGGAACTGCCTTTGATACTGGAGGTCTCGAGTCCGGGAGAGGTGAGTGGAACTGCGAGTGTAGA
CGTTGTTCGGAATCACTGGGCGTAAAGCGCACGTAGGCGGATTTGTAAGTCAGGGGTGAAATCCCGGGGCTCAACCTCGGAACTGCCTTTGATACTGCGAATCTTGAGTCCGATAGAGGTGGGTGGAATTCCTAGTGTAGAGG
CGTTGTTCGGAATTACTGGGCGTAAAGCGCACGTAGGCGGCTATTCAAGTCAGAGGTGAAAGCCCGGGGCTCAACCCCGGAACTGCCTTTGAAACTAGATAGCTTGAATCCAGGAGAGGTGAGTGGAATTCCGAGTGTAGAG
TGTTACTCAACTTGACTGGGCGTAAAGGGCGTGAAGGTGGTACAGTGCGTTTTTTTGTAAAAACTCAAACATAATTTGAGGGACTAAAAAATACGGCTGTGCTTGAGTTTACAGGAAGAAAGTTGTACTTCTAGTGTAGAGGTGAA
TGTTACTCATCAATACTGGGCGTAAAGCGCATGTAGGCGGTTTATGATGTCAGAATAAAAATCTAGAGATCACCTTTATATTAATATTTTTAAACTAATAAACTAAGAGTATAAGAGAAGTGTGTTGCACTTTCAGTGGAGAGGTAAAATA

5. Beta diversity

In [26]:
```
#Bray-Curtis distance in permanova
#based on DIY public restrooms tutorial: http://joey711.github.io/phyloseq-demo/Restroom-Biogeography.html

#Normalized by percent
bulk_sample_info = as((sample_data(ps_bulk_clean_perc)), "data.frame")
bulk_bray_perc = phyloseq::distance(ps_bulk_clean_perc, "bray")
bulk_adonis = adonis(bulk_bray_perc ~ Location + Day , bulk_sample_info)
bulk_adonis

#rarefied
bulk_sample_info = as((sample_data(ps_bulk_clean_perc)), "data.frame")
ps_bulk_clean_rare <- rarefy_even_depth(ps_bulk_clean)
bulk_bray_rare = phyloseq::distance(ps_bulk_clean_rare, "bray")
bulk_adonis_rare = adonis(bulk_bray_rare ~ Location + Day , bulk_sample_info)
bulk_adonis_rare

##CLR-transformed Euclidean distance (Aitchison distance)
##Suggested in Gloor et al 2017: https://www.ncbi.nlm.nih.gov/pmc/articles/PMC5695134/
library(microbiome)
ps_bulk_clr <- microbiome::transform(ps_bulk_clean, transform='clr')
bulk_sample_info = as(sample_data(ps_bulk_clr), "data.frame")
bulk_eucl = phyloseq::distance(ps_bulk_clr, "euclidean")
bulk_adonis_clr = adonis(bulk_eucl ~ Location + Day, bulk_sample_info)
bulk_adonis_clr
```

```
Call:
adonis(formula = bulk_bray_perc ~ Location + Day, data = bulk_sample_info)

Permutation: free
Number of permutations: 999

Terms added sequentially (first to last)

          Df SumsOfSqs MeanSqs F.Model      R2 Pr(>F)
Location   5    7.3151 1.46301  9.2221 0.57493  0.001 ***
Day        9    1.9182 0.21313  1.3435 0.15076  0.090 .
Residuals 22    3.4901 0.15864         0.27431
Total     36   12.7234                 1.00000
---
Signif. codes:  0 ‘***’ 0.001 ‘**’ 0.01 ‘*’ 0.05 ‘.’ 0.1 ‘ ’ 1

You set `rngseed` to FALSE. Make sure you've set & recorded
 the random seed of your session for reproducibility.
See `?set.seed`

...
962OTUs were removed because they are no longer
present in any sample after random subsampling

...
Call:
adonis(formula = bulk_bray_rare ~ Location + Day, data = bulk_sample_info)

Permutation: free
Number of permutations: 999

Terms added sequentially (first to last)

          Df SumsOfSqs MeanSqs F.Model      R2 Pr(>F)
Location   5    7.2847 1.45694  9.0377 0.57038  0.001 ***
Day        9    1.9404 0.21560  1.3374 0.15193  0.098 .
Residuals 22    3.5466 0.16121         0.27769
Total     36   12.7717                 1.00000
---
Signif. codes:  0 ‘***’ 0.001 ‘**’ 0.01 ‘*’ 0.05 ‘.’ 0.1 ‘ ’ 1

microbiome R package (microbiome.github.com)



 Copyright (C) 2011-2018 Leo Lahti, Sudarshan Shetty et al. <microbiome.github.io>


Attaching package: ‘microbiome’

The following object is masked from ‘package:SummarizedExperiment’:

    coverage

The following object is masked from ‘package:GenomicRanges’:

    coverage

The following objects are masked from ‘package:IRanges’:

    coverage, transform

The following object is masked from ‘package:S4Vectors’:

    transform

The following object is masked from ‘package:base’:

    transform


Call:
adonis(formula = bulk_eucl ~ Location + Day, data = bulk_sample_info)

Permutation: free
Number of permutations: 999

Terms added sequentially (first to last)

          Df SumsOfSqs MeanSqs F.Model      R2 Pr(>F)
Location   5     21626  4325.2  5.2643 0.43627  0.001 ***
Day        9      9869  1096.6  1.3347 0.19910  0.090 .
Residuals 22     18075   821.6         0.36464
Total     36     49570                 1.00000
---
Signif. codes:  0 ‘***’ 0.001 ‘**’ 0.01 ‘*’ 0.05 ‘.’ 0.1 ‘ ’ 1
```

In [27]:
```
beta <- betadisper(bulk_bray_perc, bulk_sample_info$Location)
permutest(beta, permutations = 10000)
```

```
Permutation test for homogeneity of multivariate dispersions
Permutation: free
Number of permutations: 10000

Response: Distances
          Df  Sum Sq  Mean Sq      F N.Perm  Pr(>F)
Groups     5 0.37350 0.074700 2.6871  10000 0.05519 .
Residuals 31 0.86177 0.027799
---
Signif. codes:  0 '***' 0.001 '**' 0.01 '*' 0.05 '.' 0.1 ' ' 1
```

In [28]:
```
betadisper_distances <- as.data.frame(beta$distances)
colnames(betadisper_distances) <- c('distance_to_centroid')
betadisper_distances <- merge(betadisper_distances, bulk_sample_info, by = 0)

options(repr.plot.width = 5, repr.plot.height = 3) #for plotting size in jupyter
ggplot(betadisper_distances, aes(x=Location, y=distance_to_centroid))+
    geom_boxplot()+
    geom_jitter(position=position_jitter(0.2))+
    theme(panel.background=element_blank(), panel.border=element_rect(color = "black", fill = NA), axis.text.x = element_text(angle = 90, hjust = 1, vjust = .5))
+
    ylab('Distance to centroid')

ggsave("/SCIENCE/Nelson_lab/write-ups/EPseq_paper/revised/figures/beta_dispersion.pdf", device="pdf", width=5, height=3)
```

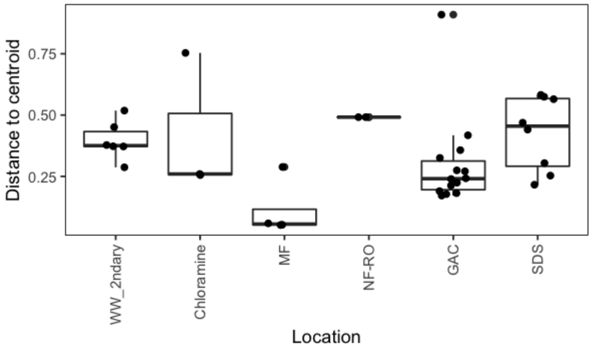

In [29]:
```
bulk.nmds.bray <- ordinate(ps_bulk_clean_perc, method="NMDS", distance="bray")
```

```
Square root transformation
Wisconsin double standardization
Run 0 stress 0.1430745
Run 1 stress 0.1497957
Run 2 stress 0.1571088
Run 3 stress 0.1446276
Run 4 stress 0.147733
Run 5 stress 0.1430027
... New best solution
... Procrustes: rmse 0.00267851  max resid 0.01171683
Run 6 stress 0.1609016
Run 7 stress 0.1372294
... New best solution
... Procrustes: rmse 0.08270109  max resid 0.2428147
Run 8 stress 0.1363555
... New best solution
... Procrustes: rmse 0.07672686  max resid 0.4364245
Run 9 stress 0.1363553
... New best solution
... Procrustes: rmse 0.000212737  max resid 0.00106038
... Similar to previous best
Run 10 stress 0.1372294
Run 11 stress 0.1372294
Run 12 stress 0.1364798
... Procrustes: rmse 0.01880287  max resid 0.09116619
Run 13 stress 0.1431415
Run 14 stress 0.1461884
Run 15 stress 0.14424
Run 16 stress 0.1337662
... New best solution
... Procrustes: rmse 0.0428591  max resid 0.2413135
Run 17 stress 0.1430026
Run 18 stress 0.1500599
Run 19 stress 0.1556339
Run 20 stress 0.151004
*** No convergence -- monoMDS stopping criteria:
    20: stress ratio > sratmax
```

In [30]:
```
#FIGURE 4
options(repr.plot.width = 4.5, repr.plot.height = 3) #for plotting size in jupyter
plot_ordination(ps_bulk_clean_perc, bulk.nmds.bray, color="Location", shape="Location") +
    geom_point(size=2)+
    scale_shape_manual(values=c(7,16,17,19,19,15, 0))+ #values=c(7,16,17,19,19,15, 0, 1)
    scale_color_manual(values = c(colors[1],colors[2],colors[3],colors[4],colors[5],colors[10], colors[11]))+ #, colors[12]
    #geom_text(aes(label=SampleID),hjust=1, vjust=1)+
    theme(panel.background=element_blank(), panel.border=element_rect(color = "black", fill = NA))
```

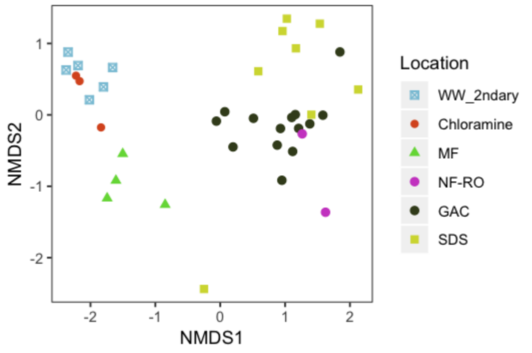

In [31]:
```
bulk.nmds.bray
```

```
Call:
metaMDS(comm = veganifyOTU(physeq), distance = distance)

global Multidimensional Scaling using monoMDS

Data:     wisconsin(sqrt(veganifyOTU(physeq)))
Distance: bray

Dimensions: 2
Stress:     0.1337662
Stress type 1, weak ties
No convergent solutions - best solution after 20 tries
Scaling: centring, PC rotation, halfchange scaling
Species: expanded scores based on 'wisconsin(sqrt(veganifyOTU(physeq)))'
```

Checking the stress-plot will tell us how well the NMDS coordinates ("ordination distances") relate to the actual dissimilarities between samples ("observed dissimilarity") as determined by Bray-Curtis dissimilarity.

See: https://mb3is.megx.net/gustame/dissimilarity-based-methods/nmds (https://mb3is.megx.net/gustame/dissimilarity-based-methods/nmds)

In [32]:
```
options(repr.plot.width = 5, repr.plot.height = 5) #for plotting size in jupyter

stressplot(bulk.nmds.bray)
```

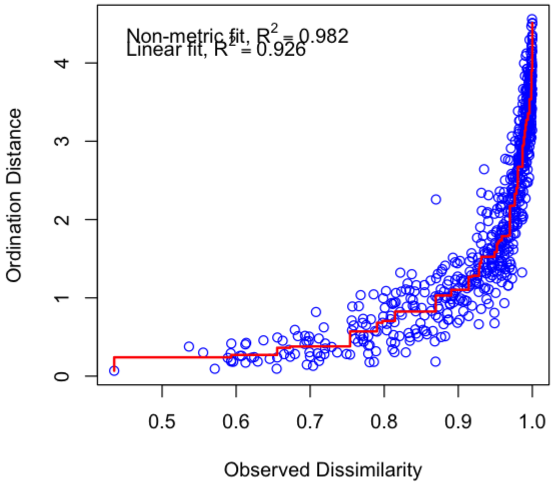

In [33]:
```
bulk.pcoa.bray <- ordinate(ps_bulk_clean_perc, method="PCoA", distance="bray")
options(repr.plot.width = 4.5, repr.plot.height = 3) #for plotting size in jupyter
plot_ordination(ps_bulk_clean_perc, bulk.pcoa.bray, color="Location", shape="Location") +
    scale_shape_manual(values=c(7,16,17,8,19,15))+
    scale_color_manual(values = c(colors[1],colors[2],colors[3],colors[4],colors[5],colors[10], colors[11], colors[12]))+
    #geom_text(aes(label=SampleID),hjust=1, vjust=1)+
    theme(panel.background=element_blank(), panel.border=element_rect(color = "black", fill = NA))
#ggsave("/SCIENCE/Nelson_lab/write-ups/EPseq_paper/revised/figures/beta_diversity_BC_PCoA_bulk.pdf", device="pdf", width=4.5, height=3)
#ggsave("/SCIENCE/Nelson_lab/write-ups/EPseq_paper/revised/figures/beta_diversity_BC_PCoA_bulk.svg", device="svg", width=4.5, height=3)
```

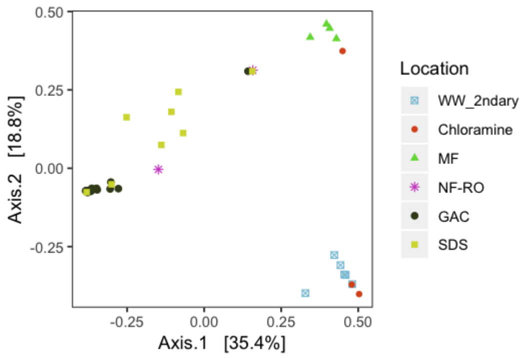

ANOSIM of bulk locations

In [34]:
```
location_group = get_variable(ps_bulk_clean_perc, "Location")
location_ano = anosim(phyloseq::distance(ps_bulk_clean_perc, "bray"), location_group)
#had to specify phyloseq here because there is another "distance" method in another package that's running
location_ano$signif
location_ano$statistic
```

0.001

0.785068114091103

SIMPER Analysis

In [35]:
```
#bulk_perc_otu <- as.data.frame(otu_table(ps_bulk_clean))
#loc <- sample_data(ps_bulk_clean)$Location
#bulk_simper <- simper(bulk_perc_otu, loc, permutations = 100)

#summary(bulk_simper) #this produces lists of OTUs responsible fore pairwise differences
```

GAC comparisons

In [36]:
```
ps_GAC_perc <- subset_samples(ps_bulk_clean_perc, subset= Location %in% "GAC")
ps_blank_sig_perc <- subset_samples(ps_bulk_blank_sig_perc, subset = Type %in% "Blank")
ps_GAC_blank_perc <- merge_phyloseq(ps_GAC_perc, ps_blank_sig_perc)
ps_GAC_blank_perc <- filter_taxa(ps_GAC_blank_perc, filterfun(kOverA(1, 0.5)), TRUE)
ps_GAC_blank_perc

ps_GAC <- subset_samples(ps_bulk_blank_sig, subset= Location %in% "GAC")
ps_blank_sig <- subset_samples(ps_bulk_blank_sig, subset = Type %in% "Blank")
ps_GAC_blank_reads <- merge_phyloseq(ps_GAC, ps_blank_sig)
ps_GAC_blank_reads <- filter_taxa(ps_GAC_blank_reads, filterfun(kOverA(1, 100)), TRUE)
```

```
phyloseq-class experiment-level object
otu_table()   OTU Table:         [ 48 taxa and 16 samples ]
sample_data() Sample Data:       [ 16 samples by 29 sample variables ]
tax_table()   Taxonomy Table:    [ 48 taxa by 6 taxonomic ranks ]
```

In [37]:
```
GAC_sample_info = as((sample_data(ps_GAC_perc)), "data.frame")
GAC_bray_perc = phyloseq::distance(ps_GAC_perc, "bray")
GAC_adonis = adonis(GAC_bray_perc ~ Description + Day , GAC_sample_info)
GAC_adonis

ps_GAC_rare <- rarefy_even_depth(ps_GAC_perc)
GAC_bray_rare = phyloseq::distance(ps_GAC_rare, "bray")
GAC_adonis_rare = adonis(GAC_bray_rare ~ Description + Day , GAC_sample_info)
GAC_adonis_rare
```

```
Call:
adonis(formula = GAC_bray_perc ~ Description + Day, data = GAC_sample_info)

Permutation: free
Number of permutations: 999

Terms added sequentially (first to last)

            Df SumsOfSqs  MeanSqs F.Model      R2 Pr(>F)
Description  2   0.28432 0.142159  1.6203 0.16962  0.046 *
Day          4   0.77772 0.194430  2.2160 0.46398  0.004 **
Residuals    7   0.61417 0.087739         0.36640
Total       13   1.67621                  1.00000
---
Signif. codes:  0 '***' 0.001 '**' 0.01 '*' 0.05 '.' 0.1 ' ' 1

You set `rngseed` to FALSE. Make sure you've set & recorded
 the random seed of your session for reproducibility.
See `?set.seed`

...
2824OTUs were removed because they are no longer
present in any sample after random subsampling

...

Call:
adonis(formula = GAC_bray_rare ~ Description + Day, data = GAC_sample_info)

Permutation: free
Number of permutations: 999

Terms added sequentially (first to last)

            Df SumsOfSqs MeanSqs F.Model      R2 Pr(>F)
Description  2   0.29496 0.14748  1.5513 0.17816  0.059 .
Day          4   0.69516 0.17379  1.8280 0.41988  0.006 **
Residuals    7   0.66549 0.09507         0.40196
Total       13   1.65560                 1.00000
---
Signif. codes:  0 '***' 0.001 '**' 0.01 '*' 0.05 '.' 0.1 ' ' 1
```

In [38]:
```
options(repr.plot.width = 9, repr.plot.height = 5)
plot_bar(ps_GAC_blank_reads, x="combined_name", fill="Family") + scale_fill_manual(values = colors) +
  theme(panel.background=element_blank(), panel.border=element_rect(color = "black", fill = NA),
        axis.text.x = element_text(angle = 45, hjust = 1, vjust = 1, size=9),
        axis.text.y = element_text(size=9),
        legend.text=element_text(size=9),
        legend.title=element_text(size=9))+
    facet_grid(.~Description, scale="free_x", space="free_x")+
ylab("Percent relative abundance") +
xlab("Sample name")+
guides(fill=guide_legend(ncol=2))

options(repr.plot.width = 9, repr.plot.height = 6.5)
plot_heatmap(ps_GAC_blank_perc, taxa.label = "Family", sample.label = "combined_name", sample.order = "Name", method="NMDS",
        low="white", high="red", na.value="black", trans = log_trans(10))+
     theme(axis.text.y = element_text(size=7),
           axis.title.y = element_blank(),
           axis.ticks = element_blank(),
           axis.text.x = element_text(angle = 45, hjust = 1, vjust = 1, size=9))+
     facet_grid(.~Description, scale="free_x", space="free_x")
```

```
Warning message in metaMDS(veganifyOTU(physeq), distance, ...):
“stress is (nearly) zero: you may have insufficient data”Warning message:
“Transformation introduced infinite values in discrete y-axis”
```

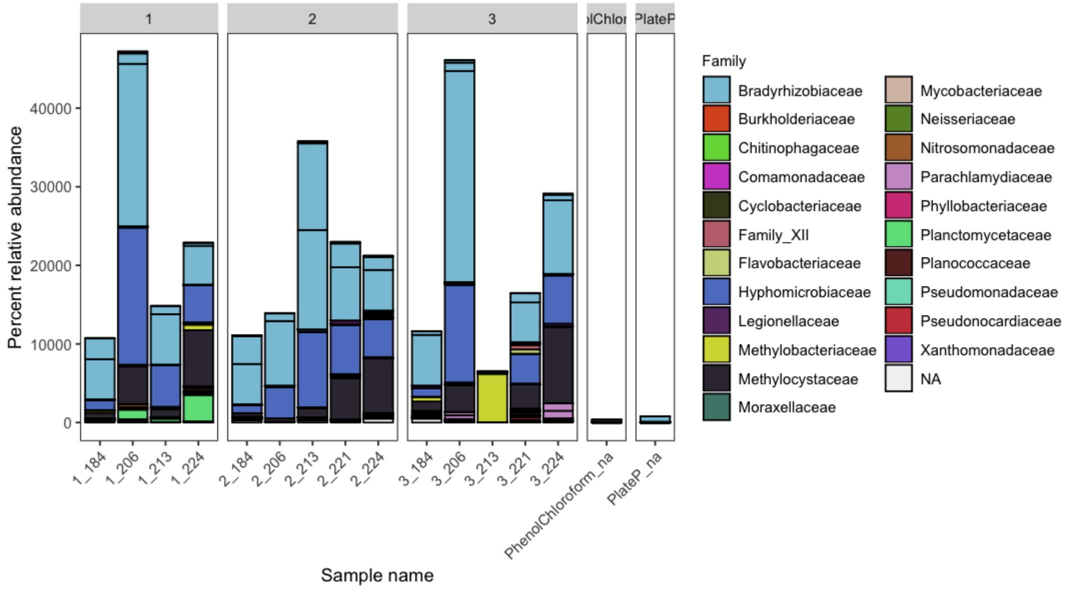

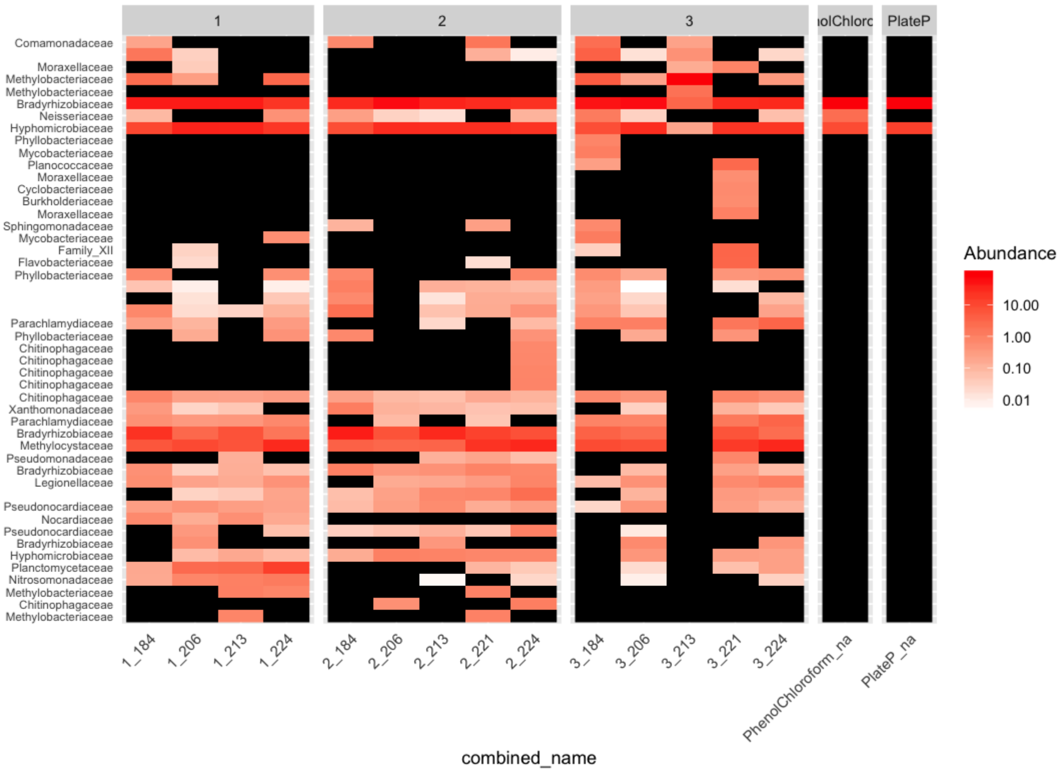

In [39]:
```
ps_GAC_blank_perc_trim <- subset_samples(ps_GAC_blank_perc, combined_name != "3_213")
GAC.pcoa.bray <- ordinate(ps_GAC_blank_perc_trim, method="PCoA", distance="bray")
options(repr.plot.width = 4.5, repr.plot.height = 3) #for plotting size in jupyter
plot_ordination(ps_GAC_blank_perc, GAC.pcoa.bray, color="Day", shape="Description") +
    scale_shape_manual(values=c(7,17,19,15, 0, 1))+
    scale_color_manual(values = c(colors[1],colors[2],colors[3],colors[4],colors[5],colors[10], colors[11], colors[12]))+
    #geom_text(aes(label=SampleID),hjust=1, vjust=1)+
    theme(panel.background=element_blank(), panel.border=element_rect(color = "black", fill = NA))
```

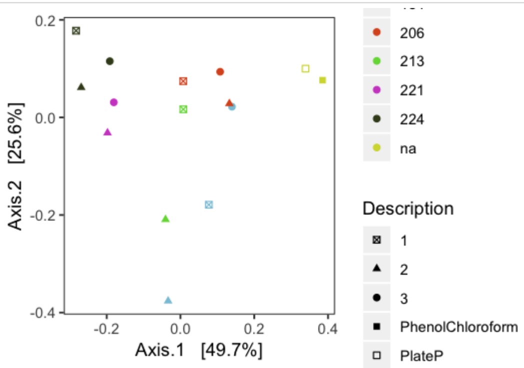

SDS Comparisons

In [40]:
```
ps_SDS_perc <- subset_samples(ps_bulk_clean_perc, subset= Location %in% "SDS")
ps_blank_sig_perc <- subset_samples(ps_bulk_blank_sig_perc, subset = Type %in% "Blank")
ps_SDS_blank_perc <- merge_phyloseq(ps_SDS_perc, ps_blank_sig_perc)
ps_SDS_blank_perc <- filter_taxa(ps_SDS_blank_perc, filterfun(kOverA(1, 0.5)), TRUE)
ps_SDS_blank_perc

ps_SDS <- subset_samples(ps_bulk_blank_sig, subset= Location %in% "SDS")
ps_blank_sig <- subset_samples(ps_bulk_blank_sig, subset = Type %in% "Blank")
ps_SDS_blank_reads <- merge_phyloseq(ps_SDS, ps_blank_sig)
ps_SDS_blank_reads <- filter_taxa(ps_SDS_blank_reads, filterfun(kOverA(1, 100)), TRUE)
ps_SDS_blank_reads
```

```
phyloseq-class experiment-level object
otu_table()   OTU Table:         [ 44 taxa and 10 samples ]
sample_data() Sample Data:       [ 10 samples by 29 sample variables ]
tax_table()   Taxonomy Table:    [ 44 taxa by 6 taxonomic ranks ]

phyloseq-class experiment-level object
otu_table()   OTU Table:         [ 41 taxa and 10 samples ]
sample_data() Sample Data:       [ 10 samples by 29 sample variables ]
tax_table()   Taxonomy Table:    [ 41 taxa by 6 taxonomic ranks ]
```

In [41]:
```
options(repr.plot.width = 9, repr.plot.height = 5)
plot_bar(ps_SDS_blank_perc, x="combined_name", fill="Family") + scale_fill_manual(values = colors) +
  theme(panel.background=element_blank(), panel.border=element_rect(color = "black", fill = NA),
        axis.text.x = element_text(angle = 45, hjust = 1, vjust = 1, size=9),
        axis.text.y = element_text(size=9),
        legend.text=element_text(size=9),
        legend.title=element_text(size=9))+
    facet_grid(.~Description, scale="free_x", space="free_x")+
ylab("Percent relative abundance") +
xlab("Sample name")+
guides(fill=guide_legend(ncol=2))

options(repr.plot.width = 9, repr.plot.height = 6.5)
plot_heatmap(ps_SDS_blank_perc, taxa.label = "Family", sample.label = "combined_name", sample.order = "Name", method="NMDS",
        low="white", high="red", na.value="black", trans = log_trans(10))+
     theme(axis.text.y = element_text(size=7),
           axis.title.y = element_blank(),
           axis.ticks = element_blank(),
           axis.text.x = element_text(angle = 45, hjust = 1, vjust = 1, size=9))+
     facet_grid(.~Description, scale="free_x", space="free_x")
```

```
Warning message:
“Transformation introduced infinite values in discrete y-axis”
```

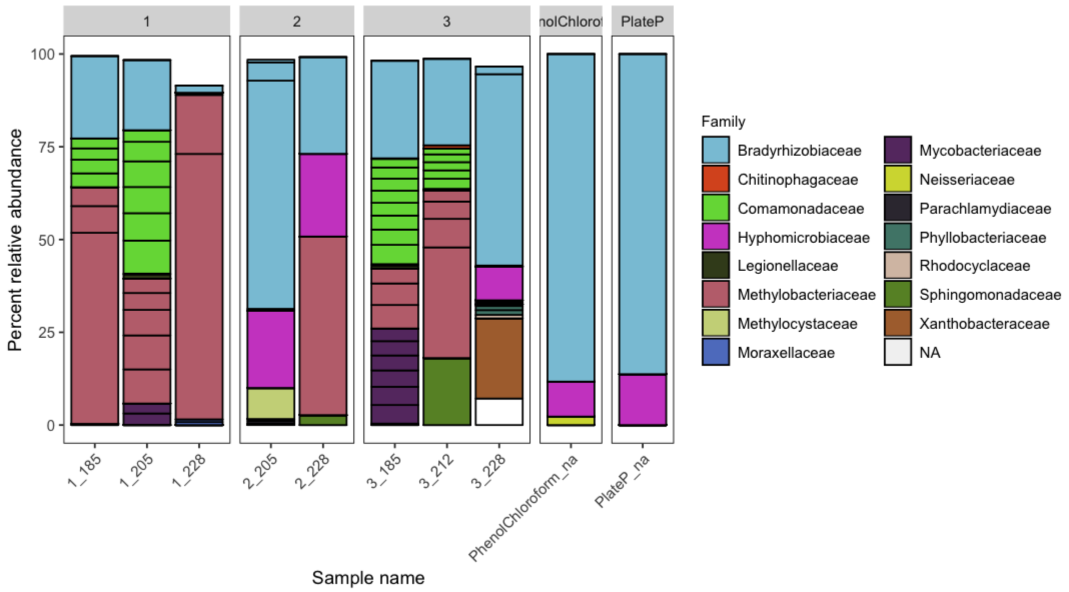

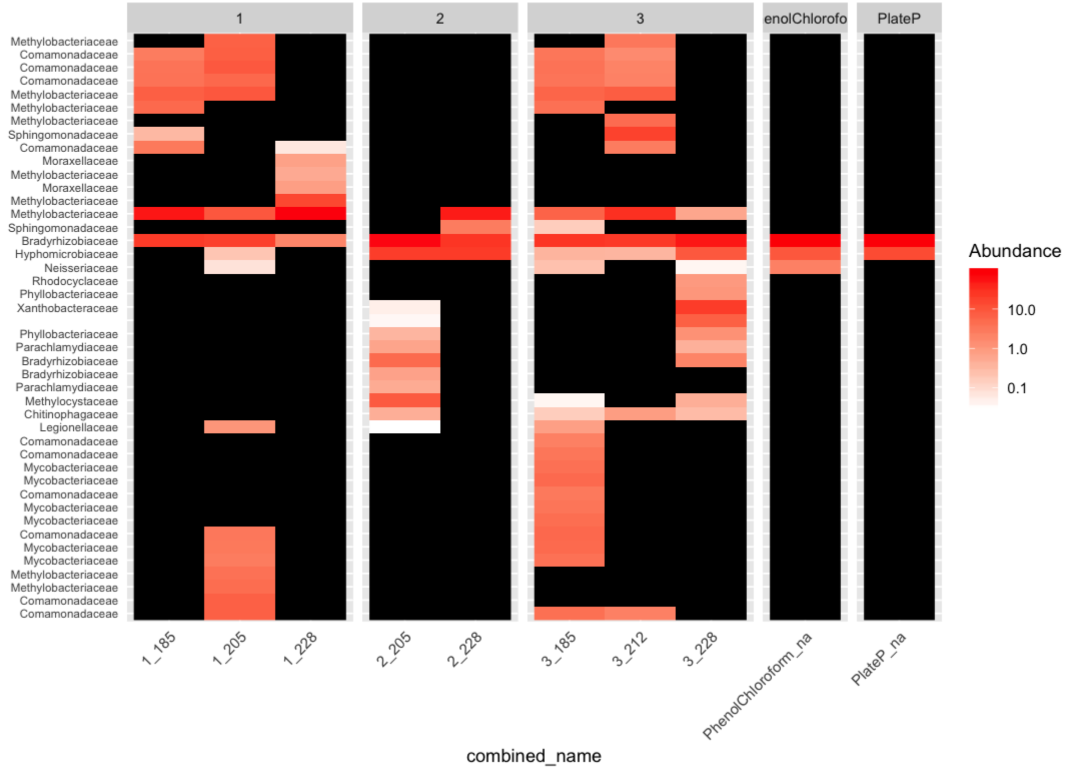

In [42]:
```
SDS.pcoa.bray <- ordinate(ps_SDS_blank_perc, method="PCoA", distance="bray")
options(repr.plot.width = 4.5, repr.plot.height = 3) #for plotting size in jupyter
plot_ordination(ps_SDS_blank_perc, SDS.pcoa.bray, color="Day", shape="Description") +
    scale_shape_manual(values=c(7,17,19,15, 0, 1))+
    scale_color_manual(values = c(colors[1],colors[2],colors[3],colors[4],colors[5],colors[10], colors[11], colors[12]))+
    #geom_text(aes(label=SampleID),hjust=1, vjust=1)+
    theme(panel.background=element_blank(), panel.border=element_rect(color = "black", fill = NA))
```

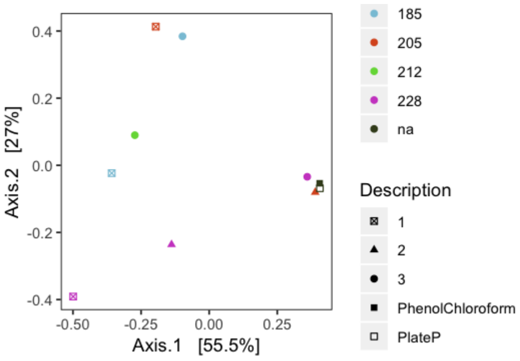

In [43]:
```
#SDS and GAC together, labeled PCoA

ps_GAC_SDS_perc <- subset_samples(ps_bulk_clean_perc, subset= Location %in% c("GAC", "SDS"))
ps_blank_sig_perc <- subset_samples(ps_bulk_blank_sig_perc, subset = Type %in% "Blank")
ps_GAC_SDS_blank_perc <- merge_phyloseq(ps_GAC_SDS_perc, ps_blank_sig_perc)
ps_GAC_SDS_blank_perc <- filter_taxa(ps_GAC_SDS_blank_perc, filterfun(kOverA(1, 0.5)), TRUE)

ps_GAC_SDS_blank_perc

GAC.SDS.pcoa.bray <- ordinate(ps_GAC_SDS_blank_perc, method="PCoA", distance="bray")
options(repr.plot.width = 6, repr.plot.height = 4)
plot_ordination(ps_GAC_SDS_blank_perc, GAC.SDS.pcoa.bray, color="Location", shape="Description") +
    geom_point(size=3)+
    geom_text(aes(label=combined_name),hjust=0.5, vjust=-1, size=3, show.legend = FALSE)+
    #scale_shape_manual(values=c(7,17,19,15, 0, 1))+
    scale_color_manual(values = c(colors[4],colors[5],colors[10], colors[12]))+
    #geom_text(aes(label=SampleID),hjust=1, vjust=1)+
    theme(panel.background=element_blank(), panel.border=element_rect(color = "black", fill = NA))
#ggsave('/SCIENCE/Nelson_lab/write-ups/EPseq_paper/revised/figures/GAC_SDS_blank_PCoA.svg', device='svg', height=4, width=6)
```

```
phyloseq-class experiment-level object
otu_table()   OTU Table:         [ 77 taxa and 24 samples ]
sample_data() Sample Data:       [ 24 samples by 29 sample variables ]
tax_table()   Taxonomy Table:    [ 77 taxa by 6 taxonomic ranks ]
```

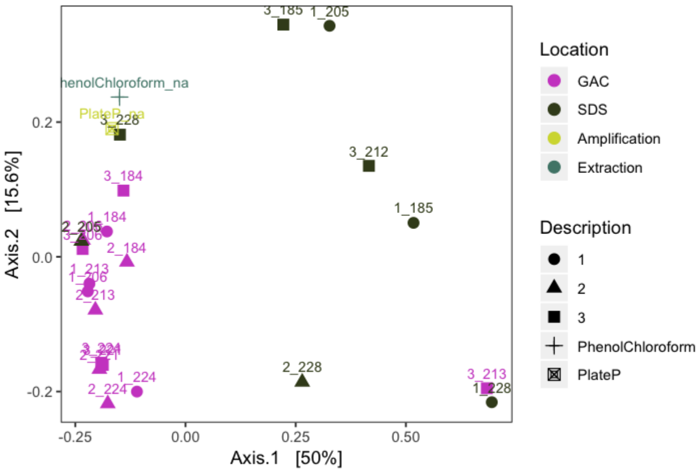

6. Potential Opportunistic Pathogens through treatment

In [44]:
```
#get ranges of flow cytometry (TCC) for each location
fcm_all$Location <- factor(fcm_all$Location, levels=c('WW_2ndary', 'Ozone', 'Chloramine', 'MF_combined', 'MF', 'NF-RO_combined', 'NF-RO', 'GAC', 'SDS'))
#change factor levels to allow NF-RO
tcc_range <- fcm_all %>% group_by(Location) %>% na.omit() %>% summarize(tcc_min = min(TCC_avg_cells_per_mL), tcc_median = median(TCC_avg_cells_per_mL), tcc_max = max(TCC_avg_cells_per_mL))
tcc_range$Location[tcc_range$Location=='NF-RO_combined'] <- 'NF-RO'
tcc_range$Location[tcc_range$Location=='MF_combined'] <- 'MF'
tcc_range
```

Location	tcc_min	tcc_median	tcc_max
WW_2ndary	6.788000e+06	9735000	70993333.0
Chloramine	2.353333e+06	7558000	57548000.0
MF	1.400000e+03	2740	5893.0
NF-RO	2.100000e+01	119	2780.0
GAC	4.593000e+03	9827	21447.0
SDS	7.333333e+01	5620	143293.3

In [45]:
```
##Legionella

#get ranges of relative abundances for each location
bulk_pathogen <- subset_taxa(ps_bulk_clean_perc, Genus=="Legionella")
bulk_pathogen <- filter_taxa(bulk_pathogen, filterfun(kOverA(1, 0.05)), TRUE) #remove any really low OTUs potentially from barcode bleed
bulk_pathogen_sum_abund <- data.frame(perc_pathogen = rowSums(otu_table(bulk_pathogen))) #sum all OTUs by sample

id2location <- subset(sample_data(bulk_pathogen), select=c(Location)) #add sample location
bulk_pathogen_sum_abund <- merge(bulk_pathogen_sum_abund, id2location, by=0)
pathogen_abund_range <- bulk_pathogen_sum_abund %>% group_by(Location) %>% summarize(Perc_min = min(perc_pathogen), Perc_median = median(perc_pathogen), Perc_max
= max(perc_pathogen))


pathogen_possible <- merge(tcc_range, pathogen_abund_range, by='Location')

#sort by location, make into rownames, then remove
pathogen_possible$Location <- factor(pathogen_possible$Location, levels=c('WW_2ndary', 'Chloramine', 'MF', 'NF-RO', 'GAC', 'SDS'))
pathogen_possible <- pathogen_possible[order(pathogen_possible$Location),]
row.names(pathogen_possible) <- pathogen_possible$Location
pathogen_possible <- subset(pathogen_possible, select=-Location)

#get ranges of absolute abundance
pathogen_possible$min <- pathogen_possible$tcc_min * pathogen_possible$Perc_min / 100
pathogen_possible$median <- pathogen_possible$tcc_median * pathogen_possible$Perc_median / 100
pathogen_possible$max <- pathogen_possible$tcc_max * pathogen_possible$Perc_max / 100

#pathogen_possible <- apply(pathogen_possible, c(1,2), function(x) signif(x, digits = 2))
pathogen_possible <- apply(pathogen_possible, c(1,2), function(x) scientific(x, digits = 2))

#pathogen_possible$TCC_range <- with(pathogen_possible, paste(tcc_min, tcc_median, tcc_max, sep=','))


#pathogen_possible$Location <- factor(pathogen_possible$Location, levels=c('WW_2ndary', 'Chloramine', 'MF', 'NF-RO', 'GAC', 'SDS'))
#pathogen_possible <- pathogen_possible[order(pathogen_possible$Location),]
#row.names(pathogen_possible) <- pathogen_possible$Location
#pathogen_possible <- subset(pathogen_possible, select=-Location)


pathogen_possible
#write.table(pathogen_possible, '/SCIENCE/Nelson_lab/write-ups/EPseq_paper/revised/figures/legionella_table.txt', sep='\t', quote=FALSE)
```

	tcc_min	tcc_median	tcc_max	Perc_min	Perc_median	Perc_max	min	median	max
**WW_2ndary**	6.8e+06	9.7e+06	7.1e+07	0e+00	8.1e-02	5.7e-01	0e+00	7.9e+03	4.1e+05
**Chloramine**	2.4e+06	7.6e+06	5.8e+07	0e+00	3.5e-02	1.1e-01	0e+00	2.6e+03	6.5e+04
**MF**	1.4e+03	2.7e+03	5.9e+03	0e+00	0e+00	0e+00	0e+00	0e+00	0e+00
**NF-RO**	2.1e+01	1.2e+02	2.8e+03	0e+00	1.6e-01	3.2e-01	0e+00	1.9e-01	8.9e+00
**GAC**	4.6e+03	9.8e+03	2.1e+04	0e+00	3e-01	1.3e+00	0e+00	3e+01	2.7e+02
**SDS**	7.3e+01	5.6e+03	1.4e+05	0e+00	3.6e-02	1.1e+00	0e+00	2e+00	1.6e+03

In [46]:
```
##Mycobacterium

#get ranges of relative abundances for each location
bulk_pathogen <- subset_taxa(ps_bulk_clean_perc, Genus=="Mycobacterium")
bulk_pathogen <- filter_taxa(bulk_pathogen, filterfun(kOverA(1, 0.05)), TRUE) #remove any really low OTUs potentially from barcode bleed
bulk_pathogen_sum_abund <- data.frame(perc_pathogen = rowSums(otu_table(bulk_pathogen))) #sum all OTUs by sample

id2location <- subset(sample_data(bulk_pathogen), select=c(Location)) #add sample location
bulk_pathogen_sum_abund <- merge(bulk_pathogen_sum_abund, id2location, by=0)
pathogen_abund_range <- bulk_pathogen_sum_abund %>% group_by(Location) %>% summarize(Perc_min = min(perc_pathogen), Perc_median = median(perc_pathogen), Perc_max
= max(perc_pathogen))

#get ranges of absolute abundance
pathogen_possible <- merge(tcc_range, pathogen_abund_range, by='Location')
pathogen_possible$min <- pathogen_possible$tcc_min * pathogen_possible$Perc_min / 100
pathogen_possible$median <- pathogen_possible$tcc_median * pathogen_possible$Perc_median / 100
pathogen_possible$max <- pathogen_possible$tcc_max * pathogen_possible$Perc_max / 100

pathogen_possible$Location <- factor(pathogen_possible$Location, levels=c('WW_2ndary', 'Chloramine', 'MF', 'NF-RO', 'GAC', 'SDS'))

pathogen_possible <- pathogen_possible[order(pathogen_possible$Location),]
row.names(pathogen_possible) <- pathogen_possible$Location
pathogen_possible <- subset(pathogen_possible, select=-Location)

pathogen_possible <- apply(pathogen_possible, c(1,2), function(x) signif(x, digits = 2))
pathogen_possible
#write.table(pathogen_possible, '/SCIENCE/Nelson_lab/write-ups/EPseq_paper/revised/figures/mycobacterium_table.txt', sep='\t', quote = FALSE)
```

	tcc_min	tcc_median	tcc_max	Perc_min	Perc_median	Perc_max	min	median	max
**WW_2ndary**	6800000	9700000	7.1e+07	0.270	0.880	2.30	1.8e+04	8.5e+04	1.7e+06
**Chloramine**	2400000	7600000	5.8e+07	0.380	1.900	87.00	8.9e+03	1.4e+05	5.0e+07
**MF**	1400	2700	5.9e+03	68.000	93.000	97.00	9.5e+02	2.5e+03	5.7e+03
**NF-RO**	21	120	2.8e+03	0.029	0.044	0.06	6.2e-03	5.3e-02	1.7e+00
**GAC**	4600	9800	2.1e+04	0.000	0.000	2.40	0.0e+00	0.0e+00	5.2e+02
**SDS**	73	5600	1.4e+05	0.000	0.074	26.00	0.0e+00	4.1e+00	3.7e+04

In [47]:
```
## pathogen plots
bulk_legionella <- subset_taxa(ps_bulk_clean_perc, Genus=="Legionella")
#filter at 0.05% to account for sample bleed
bulk_legionella <- filter_taxa(bulk_legionella, filterfun(kOverA(1, 0.05)), TRUE)
bulk_legionella

#heatmap of all Legionellales ASVs
options(repr.plot.width = 5, repr.plot.height = 3.5)
plot_heatmap(bulk_legionella, taxa.label = "Genus", sample.label = "Name", sample.order = "Name", method=NULL,
             low="white", high="red", na.value="black", trans = log_trans(10))+
            theme(axis.text.y = element_blank(), axis.title.y = element_blank(), axis.ticks = element_blank())
#ggsave("/SCIENCE/Nelson_lab/write-ups/EPseq_paper/revised/figures/Legionella_heatmap.pdf", device="pdf", width=5, height=3.5)

#relative abundance barplot
options(repr.plot.width = 5, repr.plot.height = 4)
plot_bar(bulk_legionella, x="Name", fill="OTU") +
    scale_fill_manual(values = colors[1:7]) +
    theme(panel.background=element_blank(), panel.border=element_rect(color = "black", fill = NA),
        axis.text.x = element_text(angle = 90, hjust = 1, vjust = .5), legend.position="none")+
    ylab("Percent relative\nabundance")
#ggsave("/SCIENCE/Nelson_lab/write-ups/EPseq_paper/revised/figures/Legionella_bar_perc.pdf", device="pdf", width=5, height=4)

### Absolute abundance

bulk_legionella_abs <- bulk_legionella
otu_table(bulk_legionella_abs) <- sample_data(bulk_legionella)$TCC_avg * otu_table(bulk_legionella)/100 #percent -> proportion

#trim dataset to include only samples with cell counts and legionella
bulk_legionella_abs <- filter_taxa(bulk_legionella_abs, filterfun(kOverA(1, 0)), TRUE)

options(repr.plot.width = 5, repr.plot.height = 4)
plot_bar(bulk_legionella_abs, x="Name", fill="OTU") +
    scale_fill_manual(values = colors[1:7]) +
    theme(panel.background=element_blank(), panel.border=element_rect(color = "black", fill = NA),
        axis.text.x = element_text(angle = 90, hjust = 1, vjust = .5), legend.position="none")+
    ylab("Absolute abund.\n(Total cells / mL)")
#ggsave("/SCIENCE/Nelson_lab/write-ups/EPseq_paper/revised/figures/Legionella_bar_absolute.pdf", device="pdf", width=5, height=4)
```

```
phyloseq-class experiment-level object
otu_table()   OTU Table:         [ 7 taxa and 37 samples ]
sample_data() Sample Data:       [ 37 samples by 29 sample variables ]
tax_table()   Taxonomy Table:    [ 7 taxa by 6 taxonomic ranks ]
```

```
Warning message:
“Transformation introduced infinite values in discrete y-axis”
```

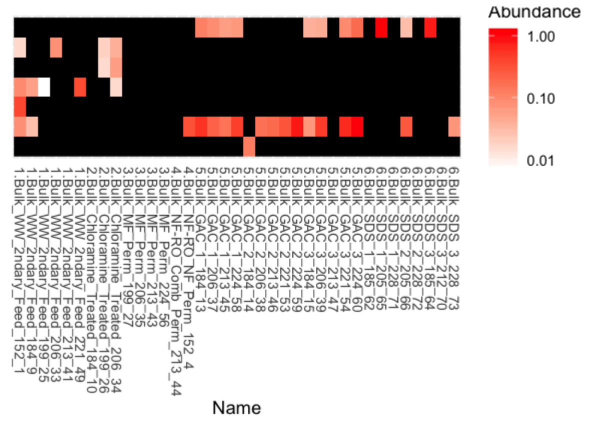


```
Warning message:
“Removed 60 rows containing missing values (position_stack).”
```

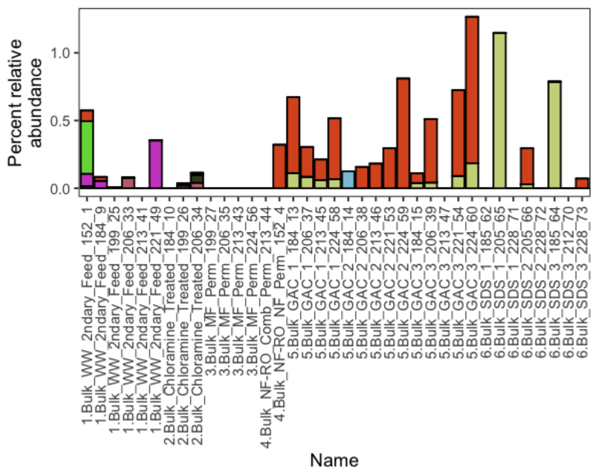

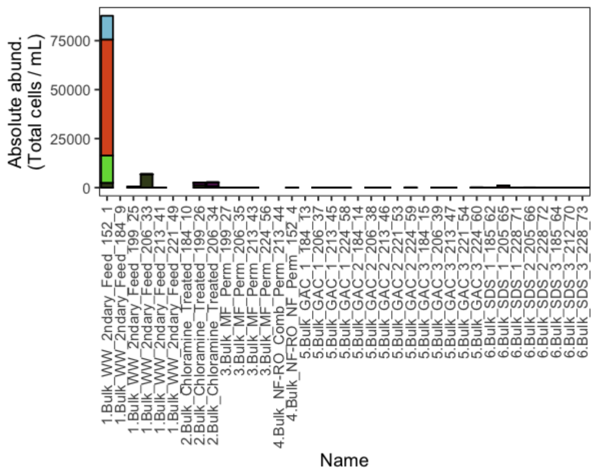

Mycobacterium species plots:

In [48]:
```
bulk_myco <- subset_taxa(ps_bulk_clean_perc, Genus=="Mycobacterium")
bulk_myco <- filter_taxa(bulk_myco, filterfun(kOverA(1, 0.05)), TRUE)
bulk_myco

#heatmap of all Mycobacterium ASVs
options(repr.plot.width = 5, repr.plot.height = 4.5)
plot_heatmap(bulk_myco, taxa.label = "Genus", sample.label = "Name", sample.order = "Name", method=NULL,
             low="white", high="red", na.value="black", trans = log_trans(10))+
            theme(axis.text.y = element_blank(), axis.title.y = element_blank(), axis.ticks = element_blank())
#ggsave("/SCIENCE/Nelson_lab/write-ups/EPseq_paper/revised/figures/Mycobacteria_heatmap.pdf", device="pdf", width=5, height=4.5)

#relative abundance barplot
options(repr.plot.width = 5, repr.plot.height = 4)
plot_bar(bulk_myco, x="Name", fill="OTU") +
    scale_fill_manual(values = colors) +
    theme(panel.background=element_blank(), panel.border=element_rect(color = "black", fill = NA),
        axis.text.x = element_text(angle = 90, hjust = 1, vjust = .5), legend.position="none")+
    ylab("Percent relative\nabundance")
#ggsave("/SCIENCE/Nelson_lab/write-ups/EPseq_paper/revised/figures/Mycobacteria_bar_perc.pdf", device="pdf", width=5, height=4)

### Absolute abundance

bulk_myco_abs <- bulk_myco
otu_table(bulk_myco_abs) <- sample_data(bulk_myco_abs)$TCC_avg * otu_table(bulk_myco_abs)/100 #percent -> proportion

#trim dataset to include only samples with cell counts and myco
bulk_myco_abs <- filter_taxa(bulk_myco_abs, filterfun(kOverA(1, 0)), TRUE)

options(repr.plot.width = 5, repr.plot.height = 4)
plot_bar(bulk_myco_abs, x="Name", fill="OTU") +
    scale_fill_manual(values = colors) +
    theme(panel.background=element_blank(), panel.border=element_rect(color = "black", fill = NA),
        axis.text.x = element_text(angle = 90, hjust = 1, vjust = .5), legend.position="none")+
    ylab("Absolute abund.\n(Total cells / mL)")
#ggsave("/SCIENCE/Nelson_lab/write-ups/EPseq_paper/revised/figures/Mycobacteria_bar_absolute.pdf", device="pdf", width=5, height=4)
```

```
phyloseq-class experiment-level object
otu_table()   OTU Table:         [ 24 taxa and 37 samples ]
sample_data() Sample Data:       [ 37 samples by 29 sample variables ]
tax_table()   Taxonomy Table:    [ 24 taxa by 6 taxonomic ranks ]

Warning message:
“Transformation introduced infinite values in discrete y-axis”
```

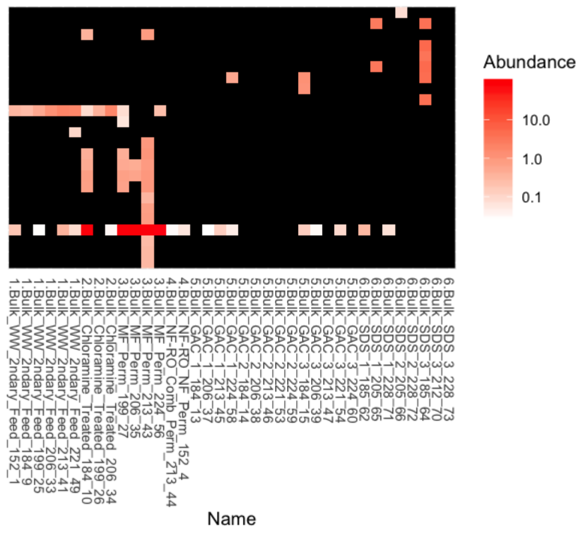


```
Warning message:
“Removed 220 rows containing missing values (position_stack).”
```

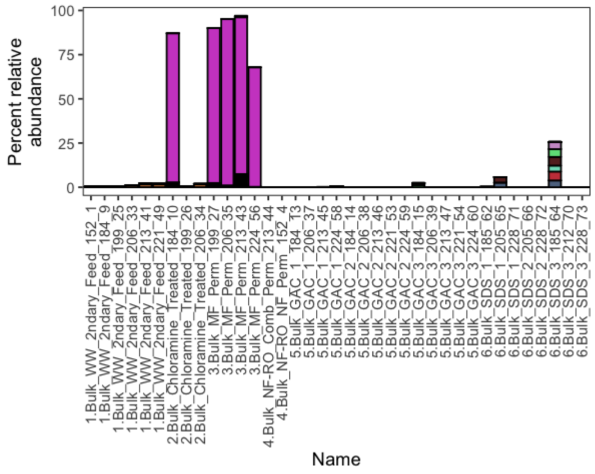

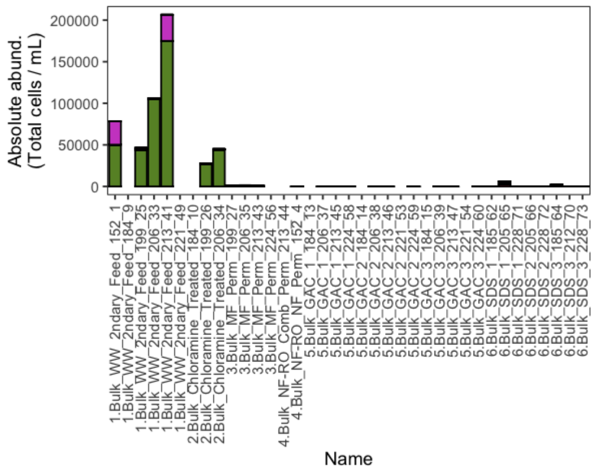

7. Shared ASVs across treatment processes

In [49]:
```
#function to identify core community within a location
get_core_seqs <- function(ps_name, min_abund, min_samples, location){
    location_ids = as.character(get_variable(ps_name, "Location")) == location
    ps_sub <- prune_samples(location_ids, ps_name) #subsample by location

    #where number of samples=min_samples, for counts input min_reads-1 (because KOverA uses > not >=)
    core <- filter_taxa(ps_sub, filterfun(kOverA(min_samples, min_abund)), prune=TRUE)
    return(row.names(tax_table(core))) #core sequences
}
```

In [50]:
```
###Figure 4A

min_abund <- 50 #reads #0.05 #percent
min_samples <- 2
#dataset <- ps_bulk_unfiltered
dataset <- ps_bulk_blank_sig

core.WW2 <- get_core_seqs(dataset, min_abund, min_samples, "WW_2ndary") #6 samples
core.MF <- get_core_seqs(dataset, min_abund, min_samples, "MF") #4 samples
core.GAC <- get_core_seqs(dataset, min_abund, min_samples, "GAC") #14 samples: 4 CatalyticReagg, 5 Reagg, 5 Coconut
core.SDS <- get_core_seqs(dataset, min_abund, min_samples, "SDS") #8 samples: 3 CatalyticReagg, 3 Reagg, 2 Coconut

options(repr.plot.width = 2, repr.plot.height = 2)
#pdf("/SCIENCE/Nelson_lab/write-ups/EPseq_paper/revised/figures/venndiagram_50reads_2samp_4way.pdf", width=2, height=2)
cores=list(
    "SDS"=core.SDS,
    "GAC"=core.GAC,
    "MF"=core.MF,
    "WW2"=core.WW2
    )
grid.draw(venn.diagram(cores, NULL, col=c("gray", "#89C5DA", "#5F7FC7", "#DA5724" ),
                       fill=c("white", "#89C5DA", "#5F7FC7", "#DA5724")))
#dev.off()

#plot intersect
partitions <- get.venn.partitions(cores)
allover_seqs <- partitions[partitions$GAC==TRUE & partitions$WW2==TRUE,]$..values..
allover_seqs <- append(allover_seqs, partitions[partitions$GAC==TRUE & partitions$MF==TRUE,]$..values..)
allover_seqs <- append(allover_seqs, partitions[partitions$SDS==TRUE & partitions$MF==TRUE,]$..values..)

allover_seqs <- unname(c(unlist(allover_seqs))) #convert to unnamed vector
ps_allover <- prune_taxa(allover_seqs, dataset)

options(repr.plot.width = 6, repr.plot.height = 4)
plot_bar(ps_allover, x="Name", fill="Family") + scale_fill_manual(values = colors) +
  theme(panel.background=element_blank(), panel.border=element_rect(color = "black", fill = NA),
        axis.text.x = element_text(angle = 90, hjust = 1, vjust = .5))

options(repr.plot.width = 6.5, repr.plot.height = 3.5)
plot_heatmap(ps_allover, taxa.label = "Family", sample.label = "Name", sample.order = "Name", method=NULL,
             low="white", high="red", na.value="black", trans = log_trans(10))
```

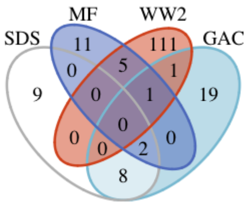


```
Warning message:
“Transformation introduced infinite values in discrete y-axis”
```

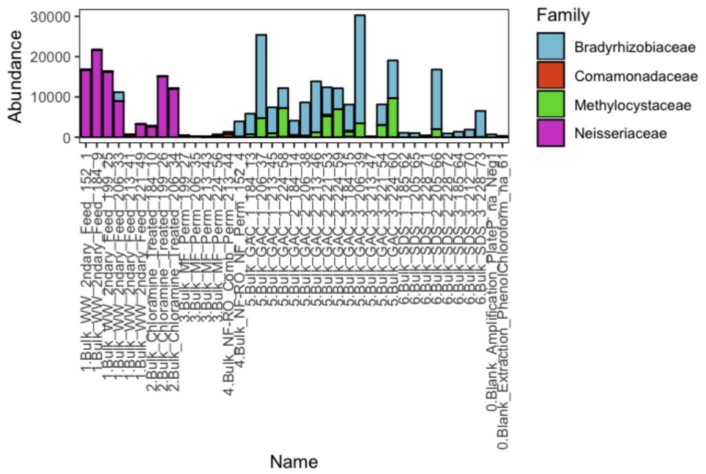

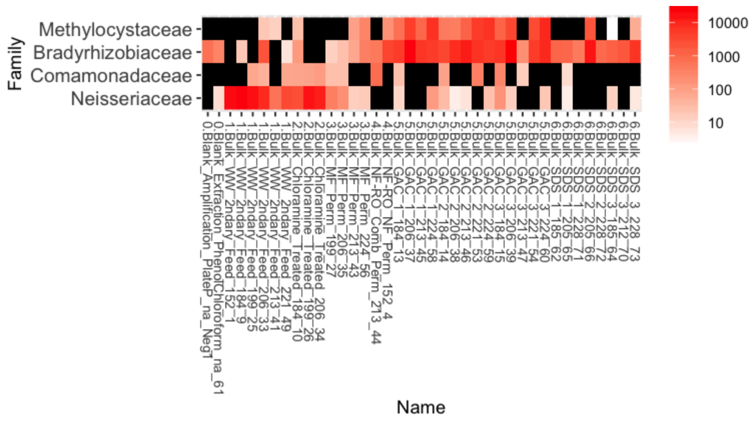

In [51]:
```
#what are these seqs and how abundant are they? Get otu table, tax table, merge, rename

shared_otus <- as.data.frame(otu_table(ps_allover)) #get otu table
shared_otus <- as.data.frame(t(shared_otus)) #otus as rows
shared_taxa <- as.data.frame(tax_table(ps_allover) [, c("Family","Genus")]) #get tax table
shared_seq_info <- merge(shared_otus, shared_taxa, by=0) #merge otus and tax tables

shared_seq_info$taxonomy <- paste(shared_seq_info$Family, shared_seq_info$Genus, sep="_") #convert tax to family_genus
row.names(shared_seq_info) <- shared_seq_info$taxonomy #make tax as rownames
shared_seq_info <- subset(shared_seq_info, select=c(-Row.names, -Family, -Genus, -taxonomy)) #drop extra columns

shared_seq_info <- t((shared_seq_info)) #transpose so taxa are columns, samples are rows
shared_seq_info <- as.data.frame(shared_seq_info)
names <- subset(sample_data(ps_allover), select=c(Name, filtered_readcounts)) #get sample names for rows
shared_seq_info <- merge(names, shared_seq_info, by=0)
row.names(shared_seq_info) <- shared_seq_info$Name
shared_seq_info <- subset(shared_seq_info, select=c(-Name, -Row.names))
shared_seq_info <- shared_seq_info[order(row.names(shared_seq_info)),] #sort by sample name

shared_seq_info
#write.table(shared_seq_info,'/SCIENCE/Nelson_lab/write-ups/EPseq_paper/revised/figures/shared_sequences_across_treatment.txt', sep='\t', quote=FALSE)
```

	filtered_readcounts	Neisseriaceae_NA	Comamonadaceae_Ottowia	Bradyrhizobiaceae_Bradyrhizobium	Methylocystaceae_Methylocystis
**0.Blank_Amplification_PlateP_na_Neg1**	787	0	0	679	0
**0.Blank_Extraction_PhenolChloroform_na_61**	350	8	0	309	0
**1.Bulk_WW_2ndary_Feed_152_1**	68076	16753	0	0	0
**1.Bulk_WW_2ndary_Feed_184_9**	41718	21689	0	20	0
**1.Bulk_WW_2ndary_Feed_199_25**	70994	16282	70	0	0
**1.Bulk_WW_2ndary_Feed_206_33**	26747	8934	48	2168	20
**1.Bulk_WW_2ndary_Feed_213_41**	8964	597	0	0	13
**1.Bulk_WW_2ndary_Feed_221_49**	16410	3222	73	6	0
**2.Bulk_Chloramine_Treated_184_10**	66864	2638	78	124	27
**2.Bulk_Chloramine_Treated_199_26**	57945	15111	86	0	0
**2.Bulk_Chloramine_Treated_206_34**	44893	12040	116	0	0
**3.Bulk_MF_Perm_199_27**	41160	393	27	13	0
**3.Bulk_MF_Perm_206_35**	54916	193	27	25	0
**3.Bulk_MF_Perm_213_43**	78462	14	0	71	93
**3.Bulk_MF_Perm_224_56**	20014	17	0	364	309
**4.Bulk_NF-RO_Comb_Perm_213_44**	34040	0	814	494	0
**4.Bulk_NF-RO_NF_Perm_152_4**	16778	0	0	3724	175
**5.Bulk_GAC_1_184_13**	11601	10	25	5110	695
**5.Bulk_GAC_1_206_37**	47819	0	0	20669	4756
**5.Bulk_GAC_1_213_45**	15050	0	0	6406	995
**5.Bulk_GAC_1_224_58**	23825	112	0	4940	7132
**5.Bulk_GAC_2_184_14**	12002	31	84	3530	456
**5.Bulk_GAC_2_206_38**	13960	4	0	8208	410
**5.Bulk_GAC_2_213_46**	36201	6	0	12643	1205
**5.Bulk_GAC_2_221_53**	23698	0	368	6764	5241
**5.Bulk_GAC_2_224_59**	21592	24	0	5165	6946
**5.Bulk_GAC_3_184_15**	12726	160	280	6448	1201
**5.Bulk_GAC_3_206_39**	46546	14	0	26843	3430
**5.Bulk_GAC_3_213_47**	6578	0	15	197	0
**5.Bulk_GAC_3_221_54**	17516	0	0	5054	3070
**5.Bulk_GAC_3_224_60**	29849	19	0	9358	9687
**6.Bulk_SDS_1_185_62**	4863	0	0	1079	0
**6.Bulk_SDS_1_205_65**	5318	4	8	1005	0
**6.Bulk_SDS_1_228_71**	23382	0	0	450	0
**6.Bulk_SDS_2_205_66**	24055	0	0	14792	2007
**6.Bulk_SDS_2_228_72**	3499	0	0	912	0
**6.Bulk_SDS_3_185_64**	5085	12	0	1336	2
**6.Bulk_SDS_3_212_70**	8054	0	0	1876	0
**6.Bulk_SDS_3_228_73**	12531	5	0	6462	58

In [52]:
```
#prepare data for ggplot-style heatmap
shared_seq_info2 <- shared_seq_info

shared_seq_info2$samples <- row.names(shared_seq_info2)
read_counts_only <- subset(shared_seq_info2, select=c(filtered_readcounts, samples))
shared_seq_info2 <- subset(shared_seq_info2, select=-filtered_readcounts)
shared_seq_info_melt <- melt(shared_seq_info2, id.vars='samples', value.name = 'reads', variable.name = 'taxon')
readcounts_melt <- melt(read_counts_only, id.vars='samples', value.name = 'reads', variable.name = 'filtered')

shared_seq_perc <- shared_seq_info

shared_seq_perc <- shared_seq_perc * 100 / shared_seq_perc$filtered_readcounts
shared_seq_perc$samples <- row.names(shared_seq_perc)
shared_seq_perc <- subset(shared_seq_perc, select=-filtered_readcounts)
shared_seq_perc_melt <- melt(shared_seq_perc, id.vars='samples', value.name = 'perc_rel_abund', variable.name = 'taxon')
```

In [53]:
```
options(repr.plot.width = 6, repr.plot.height = 8)
ggplot(shared_seq_info_melt, aes(taxon, samples)) +
    geom_tile(aes(fill = reads)) +
    geom_text(aes(label = reads), size=3) +
    scale_y_discrete(limits = rev((shared_seq_info2$samples)))+ #reverse order of y axis
    scale_fill_gradient(low = "white", high = "blue", name="Read count")+
    xlab("Shared taxa")+
    ylab("Samples")+
    theme(panel.background=element_blank(), panel.border=element_rect(color = "black", fill = NA),
        axis.text.x = element_text(angle = 45, hjust = 1, vjust = 1))
#ggsave("/SCIENCE/Nelson_lab/write-ups/EPseq_paper/revised/figures/shared_text_heatmap_reads.pdf", device="pdf", width=6, height=8)

#plot readcounts as their own column
options(repr.plot.width = 2, repr.plot.height = 8)
ggplot(readcounts_melt, aes(filtered, samples)) +
    geom_tile(aes(fill = reads)) +
    geom_text(aes(label = reads), size=3) +
    scale_y_discrete(limits = rev((read_counts_only$samples)))+ #reverse order of y axis
    scale_fill_gradient(low = "white", high = "white", name="Read count")+
    xlab("Total reads")+
    ylab("Samples")+
    theme(panel.background=element_blank(), panel.border=element_rect(color = "black", fill = NA),
        axis.text.x = element_text(angle = 45, hjust = 1, vjust = 1), axis.text.y = element_blank())
#ggsave("/SCIENCE/Nelson_lab/write-ups/EPseq_paper/revised/figures/shared_text_readcounts-only.pdf", device="pdf", width=2, height=7.5)


options(repr.plot.width = 6, repr.plot.height = 8)
ggplot(shared_seq_perc_melt, aes(taxon, samples)) +
    geom_tile(aes(fill = perc_rel_abund)) +
    geom_text(aes(label = round(perc_rel_abund,2)), size=3) +
    scale_y_discrete(limits = rev((shared_seq_perc$samples)))+ #reverse order of y axis
    scale_fill_gradient(low = "white", high = "red", name="Percent\nRelative\nAbundance")+
    xlab("Shared taxa")+
    ylab("Samples")+
    theme(panel.background=element_blank(), panel.border=element_rect(color = "black", fill = NA),
        axis.text.x = element_text(angle = 45, hjust = 1, vjust = 1))
#ggsave("/SCIENCE/Nelson_lab/write-ups/EPseq_paper/revised/figures/shared_text_heatmap_perc.pdf", device="pdf", width=6, height=8)
```

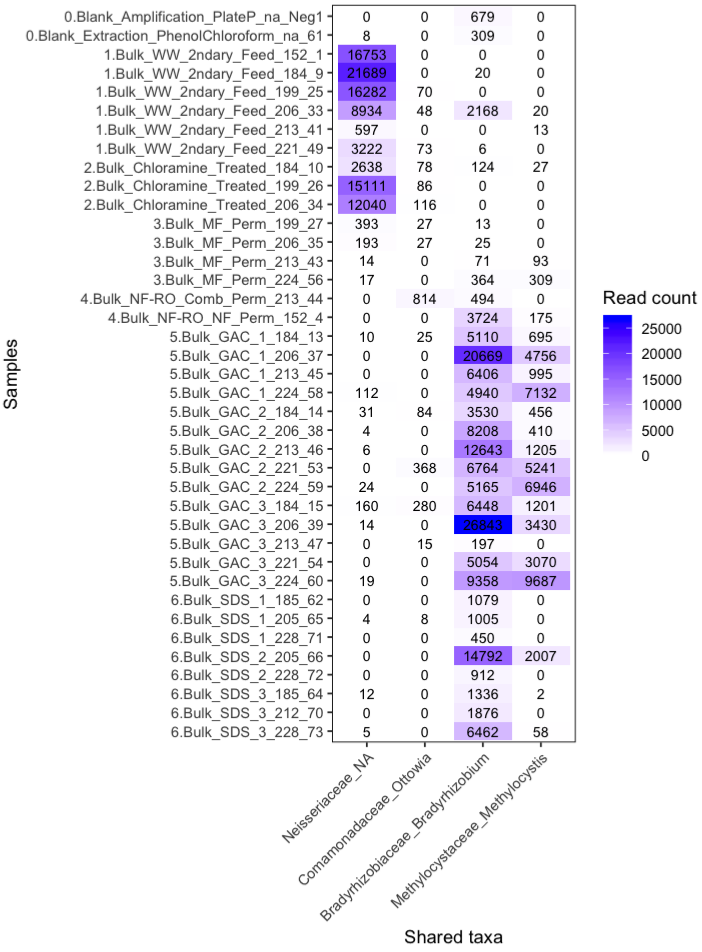
0.Blank_Amplification_PlateP_na_Neg1 0 0 679 0
0.Blank_Extraction_PhenolChloroform_na_61 8 0 309 0
1.Bulk_WW_2ndary_Feed_152_1 16753 0 0 0
1.Bulk_WW_2ndary_Feed_184_9 21689 0 20 0
1.Bulk_WW_2ndary_Feed_199_25 16282 70 0 0
1.Bulk_WW_2ndary_Feed_206_33 8934 48 2168 20
1.Bulk_WW_2ndary_Feed_213_41 597 0 0 13
1.Bulk_WW_2ndary_Feed_221_49 3222 73 6 0
2.Bulk_Chloramine_Treated_184_10 2638 78 124 27
2.Bulk_Chloramine_Treated_199_26 15111 86 0 0
2.Bulk_Chloramine_Treated_206_34 12040 116 0 0
3.Bulk_MF_Perm_199_27 393 27 13 0
3.Bulk_MF_Perm_206_35 193 27 25 0
3.Bulk_MF_Perm_213_43 14 0 71 93
3.Bulk_MF_Perm_224_56 17 0 364 309
4.Bulk_NF-RO_Comb_Perm_213_44 0 814 494 0
4.Bulk_NF-RO_NF_Perm_152_4 0 0 3724 175
5.Bulk_GAC_1_184_13 10 25 5110 695
5.Bulk_GAC_1_206_37 0 0 20669 4756
5.Bulk_GAC_1_213_45 0 0 6406 995
5.Bulk_GAC_1_224_58 112 0 4940 7132
5.Bulk_GAC_2_184_14 31 84 3530 456
5.Bulk_GAC_2_206_38 4 0 8208 410
5.Bulk_GAC_2_213_46 6 0 12643 1205
5.Bulk_GAC_2_221_53 0 368 6764 5241
5.Bulk_GAC_2_224_59 24 0 5165 6946
5.Bulk_GAC_3_184_15 160 280 6448 1201
5.Bulk_GAC_3_206_39 14 0 26843 3430
5.Bulk_GAC_3_213_47 0 15 197 0
5.Bulk_GAC_3_221_54 0 0 5054 3070
5.Bulk_GAC_3_224_60 19 0 9358 9687
6.Bulk_SDS_1_185_62 0 0 1079 0
6.Bulk_SDS_1_205_65 4 8 1005 0
6.Bulk_SDS_1_228_71 0 0 450 0
6.Bulk_SDS_2_205_66 0 0 14792 2007
6.Bulk_SDS_2_228_72 0 0 912 0
6.Bulk_SDS_3_185_64 12 0 1336 2
6.Bulk_SDS_3_212_70 0 0 1876 0
6.Bulk_SDS_3_228_73 5 0 6462 58
Samples
Neisseriaceae_NA
Comamonadaceae_Ottowia
Bradyrhizobiaceae_Bradyrhizobium
Methylocystaceae_Methylocystis
Shared taxa
Read count
25000
20000
15000
10000
5000
0

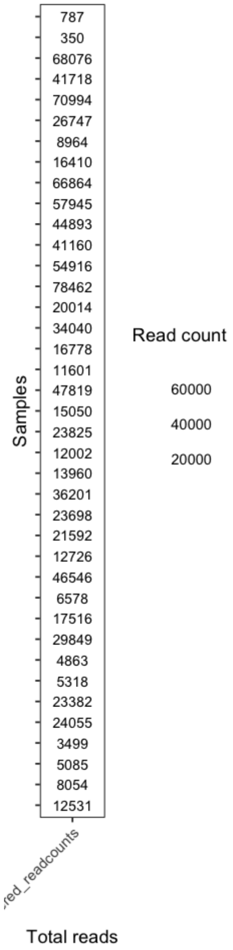
787
350
68076
41718
70994
26747
8964
16410
66864
57945
44893
41160
54916
78462
20014
34040
16778
11601
47819
15050
23825
12002
13960
36201
23698
21592
12726
46546
6578
17516
29849
4863
5318
23382
24055
3499
5085
8054
12531
Samples
red_readcounts
Total reads
Read count
60000
40000
20000

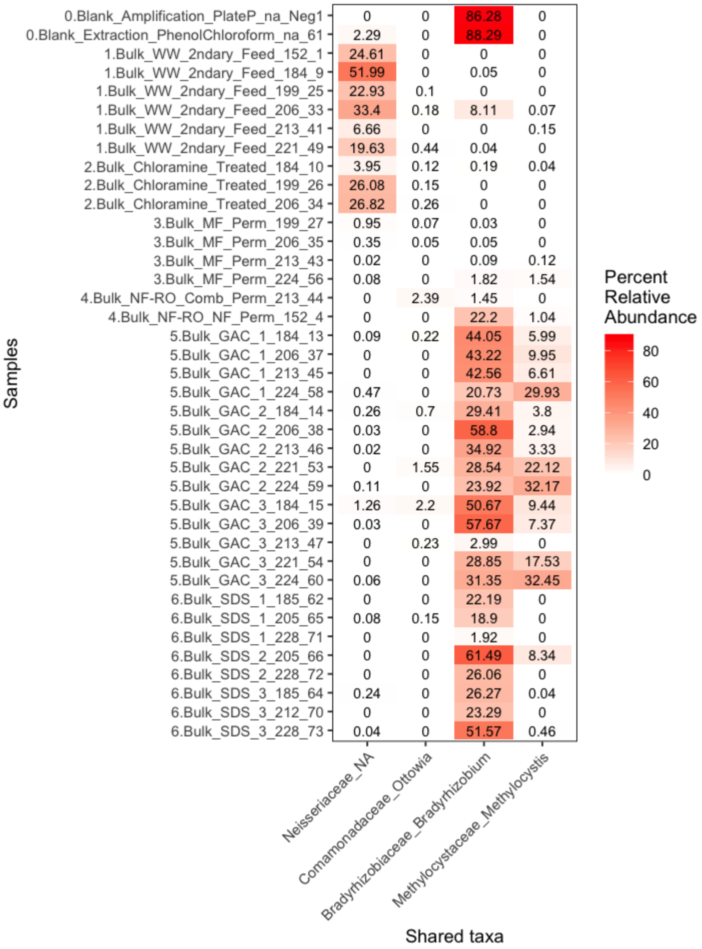

Samples
0.Blank_Amplification_PlateP_na_Neg1 0 0 86.28 0
0.Blank_Extraction_PhenolChloroform_na_61 2.29 0 88.29 0
1.Bulk_WW_2ndary_Feed_152_1 24.61 0 0 0
1.Bulk_WW_2ndary_Feed_184_9 51.99 0 0.05 0
1.Bulk_WW_2ndary_Feed_199_25 22.93 0.1 0 0
1.Bulk_WW_2ndary_Feed_206_33 33.4 0.18 8.11 0.07
1.Bulk_WW_2ndary_Feed_213_41 6.66 0 0 0.15
1.Bulk_WW_2ndary_Feed_221_49 19.63 0.44 0.04 0
2.Bulk_Chloramine_Treated_184_10 3.95 0.12 0.19 0.04
2.Bulk_Chloramine_Treated_199_26 26.08 0.15 0 0
2.Bulk_Chloramine_Treated_206_34 26.82 0.26 0 0
3.Bulk_MF_Perm_199_27 0.95 0.07 0.03 0
3.Bulk_MF_Perm_206_35 0.35 0.05 0.05 0
3.Bulk_MF_Perm_213_43 0.02 0 0.09 0.12
3.Bulk_MF_Perm_224_56 0.08 0 1.82 1.54
4.Bulk_NF-RO_Comb_Perm_213_44 0 2.39 1.45 0
4.Bulk_NF-RO_NF_Perm_152_4 0 0 22.2 1.04
5.Bulk_GAC_1_184_13 0.09 0.22 44.05 5.99
5.Bulk_GAC_1_206_37 0 0 43.22 9.95
5.Bulk_GAC_1_213_45 0 0 42.56 6.61
5.Bulk_GAC_1_224_58 0.47 0 20.73 29.93
5.Bulk_GAC_2_184_14 0.26 0.7 29.41 3.8
5.Bulk_GAC_2_206_38 0.03 0 58.8 2.94
5.Bulk_GAC_2_213_46 0.02 0 34.92 3.33
5.Bulk_GAC_2_221_53 0 1.55 28.54 22.12
5.Bulk_GAC_2_224_59 0.11 0 23.92 32.17
5.Bulk_GAC_3_184_15 1.26 2.2 50.67 9.44
5.Bulk_GAC_3_206_39 0.03 0 57.67 7.37
5.Bulk_GAC_3_213_47 0 0.23 2.99 0
5.Bulk_GAC_3_221_54 0 0 28.85 17.53
5.Bulk_GAC_3_224_60 0.06 0 31.35 32.45
6.Bulk_SDS_1_185_62 0 0 22.19 0
6.Bulk_SDS_1_205_65 0.08 0.15 18.9 0
6.Bulk_SDS_1_228_71 0 0 1.92 0
6.Bulk_SDS_2_205_66 0 0 61.49 8.34
6.Bulk_SDS_2_228_72 0 0 26.06 0
6.Bulk_SDS_3_185_64 0.24 0 26.27 0.04
6.Bulk_SDS_3_212_70 0 0 23.29 0
6.Bulk_SDS_3_228_73 0.04 0 51.57 0.46
Neisseriaceae_NA
Comamonadaceae_Ottowia
Bradyrhizobiaceae_Bradyrhizobium
Methylocystaceae_Methylocystis
Shared taxa
Percent Relative Abundance
80
60
40
20
0
