# Supplementary material for: The Water Microbiome Through a Pilot Scale Advanced Treatment Facility for Direct Potable Reuse
Source: Front Microbiol. 2019 May 8;10:993. doi: 10.3389/fmicb.2019.00993 (PMC6517601; doi:10.3389/fmicb.2019.00993)
Supplement: Supplementary file 3 [file Data_Sheet_3.PDF]

In [1]:
```
library(ggplot2)
library(tidyr)
library(plyr)
library(dplyr)
library(vegan)
library(scales)
library(repr)
library(reshape2)
library(pheatmap)
library(RColorBrewer)
library(viridis)
library(phyloseq)
library(genefilter)
options(jupyter.plot_mimetypes = c("text/plain", "image/png" ))

#colorblind color vector for taxonomy plots
colors <- c("#89C5DA", "#DA5724", "#74D944", "#CE50CA", "#3F4921", "#C0717C", "#CBD588", "#5F7FC7",
            "#673770", "#D3D93E", "#38333E", "#508578", "#D7C1B1", "#689030", "#AD6F3B", "#CD9BCD",
            "#D14285", "#6DDE88", "#652926", "#7FDCC0", "#C84248", "#8569D5", "#5E738F", "#D1A33D",
            "#8A7C64", "#599861", "orange", "666666", "gray80", "#FFCC00")
```

```
Warning message:
"package 'dplyr' was built under R version 3.5.1"
Attaching package: 'dplyr'

The following objects are masked from 'package:plyr':

    arrange, count, desc, failwith, id, mutate, rename, summarise,
    summarize

The following objects are masked from 'package:stats':

    filter, lag

The following objects are masked from 'package:base':

    intersect, setdiff, setequal, union

Loading required package: permute
Loading required package: lattice
This is vegan 2.5-2

Attaching package: 'reshape2'

The following object is masked from 'package:tidyr':

    smiths

Loading required package: viridisLite

Attaching package: 'viridis'

The following object is masked from 'package:scales':

    viridis_pal
```

Compare to metagenomic and 16S rRNA gene amplicon data

Metaphlan

In [2]:
```
metaphlan.fam <- read.table("/SCIENCE/Nelson_lab/data_files_nelson/el_paso_metagenomics/metaphlan.all.family.txt", sep="\t", col.names = c("Family", "Relative_abundance", "Sample_number"))
metaphlan.fam <- separate(data=metaphlan.fam, col=Family, sep = "f__", into=c("taxonomy_string", "Family"))

#add column indicating type of sequencing (for future combination with amplicon data)
Sequencing <- rep("Metagenomic", length(metaphlan.fam$Family))
metaphlan.fam <- cbind(metaphlan.fam, Sequencing)

#add sample name
metagenome_info <- read.table(
    "/SCIENCE/Nelson_lab/data_files_nelson/el_paso_metagenomics/metagenome_info.txt",
    header=TRUE, sep="\t")[1:2]

#get sample names and order for plotting
metaphlan.fam <- merge(metaphlan.fam, metagenome_info, by="Sample_number", all.x=TRUE)
metaphlan.fam$Sample_code <- factor(metaphlan.fam$Sample_code, levels=c(
    'WW_2ndary_152', 'WW_2ndary_184', 'WW_2ndary_206',
    'GAC_media1_234', 'GAC_media2_234', 'GAC_media3_234',
    'GAC_filt1_206', 'GAC_filt2_206', 'GAC_filt3_206',
    'SDS_1_205', 'SDS_2_205', 'SDS_3_205', 'Zymo_mock_DNA'))

metaphlan.fam <- subset(metaphlan.fam, select=c(Sample_code, Family, Relative_abundance, Sequencing))
metaphlan.fam <- metaphlan.fam[metaphlan.fam$Relative_abundance>=1,]

#remove the virus
metaphlan.fam <- metaphlan.fam[metaphlan.fam$Family!="Siphoviridae",]
```

In [3]:
```
metaphlan.fam
```

	Sample_code	Family	Relative_abundance	Sequencing
1	WW_2ndary_152	Rhodocyclaceae	72.20745	Metagenomic
2	WW_2ndary_152	Comamonadaceae	3.95739	Metagenomic
3	WW_2ndary_152	Pseudomonadaceae	3.21297	Metagenomic
4	WW_2ndary_152	Neisseriaceae	3.19016	Metagenomic
5	WW_2ndary_152	Aeromonadaceae	2.46961	Metagenomic
6	WW_2ndary_152	Mycobacteriaceae	2.33362	Metagenomic
7	WW_2ndary_152	Campylobacteraceae	2.23222	Metagenomic
8	WW_2ndary_152	Moraxellaceae	2.04776	Metagenomic
10	WW_2ndary_152	Bacteroidaceae	1.35564	Metagenomic
11	WW_2ndary_152	Burkholderiales_noname	1.07514	Metagenomic
32	GAC_media1_234	Methylobacteriaceae	49.21580	Metagenomic
33	GAC_media1_234	Hyphomicrobiaceae	28.74246	Metagenomic
34	GAC_media1_234	Phyllobacteriaceae	11.21916	Metagenomic
35	GAC_media1_234	Bradyrhizobiaceae	10.82258	Metagenomic
36	GAC_media2_234	Bradyrhizobiaceae	34.36884	Metagenomic
37	GAC_media2_234	Methylobacteriaceae	33.29071	Metagenomic
38	GAC_media2_234	Hyphomicrobiaceae	29.38093	Metagenomic
39	GAC_media2_234	Phyllobacteriaceae	2.37096	Metagenomic
41	GAC_media3_234	Bradyrhizobiaceae	66.14378	Metagenomic
42	GAC_media3_234	Hyphomicrobiaceae	18.71722	Metagenomic
43	GAC_media3_234	Phyllobacteriaceae	13.00175	Metagenomic
44	GAC_media3_234	Methylobacteriaceae	2.02819	Metagenomic
47	Zymo_mock_DNA	Enterobacteriaceae	20.31385	Metagenomic
48	Zymo_mock_DNA	Staphylococcaceae	16.99908	Metagenomic
49	Zymo_mock_DNA	Enterococcaceae	15.92603	Metagenomic
50	Zymo_mock_DNA	Lactobacillaceae	15.17750	Metagenomic
51	Zymo_mock_DNA	Listeriaceae	12.63227	Metagenomic
52	Zymo_mock_DNA	Pseudomonadaceae	9.91262	Metagenomic
53	Zymo_mock_DNA	Bacillaceae	8.16963	Metagenomic
57	WW_2ndary_206	Moraxellaceae	41.82474	Metagenomic
⋮	⋮	⋮	⋮	⋮
107	SDS_1_205	Burkholderiaceae	12.59560	Metagenomic
108	SDS_1_205	Comamonadaceae	8.35788	Metagenomic
109	SDS_1_205	Caulobacteraceae	8.23410	Metagenomic
110	SDS_1_205	Hyphomicrobiaceae	2.31857	Metagenomic
112	SDS_2_205	Methylobacteriaceae	36.40596	Metagenomic
113	SDS_2_205	Sphingomonadaceae	31.75282	Metagenomic
114	SDS_2_205	Bradyrhizobiaceae	13.54169	Metagenomic
115	SDS_2_205	Burkholderiales_noname	6.32820	Metagenomic
116	SDS_2_205	Hyphomicrobiaceae	5.51605	Metagenomic
117	SDS_2_205	Comamonadaceae	2.26178	Metagenomic
118	SDS_2_205	Burkholderiaceae	1.80417	Metagenomic
125	SDS_3_205	Methylobacteriaceae	18.65102	Metagenomic
126	SDS_3_205	Caulobacteraceae	15.77943	Metagenomic
127	SDS_3_205	Hyphomicrobiaceae	15.40430	Metagenomic
128	SDS_3_205	Comamonadaceae	13.83901	Metagenomic
129	SDS_3_205	Burkholderiaceae	13.68174	Metagenomic
130	SDS_3_205	Burkholderiales_noname	11.59973	Metagenomic
131	SDS_3_205	Bradyrhizobiaceae	9.33054	Metagenomic
132	SDS_3_205	Peptostreptococcaceae	1.29042	Metagenomic
134	WW_2ndary_184	Rhodocyclaceae	52.57059	Metagenomic
135	WW_2ndary_184	Comamonadaceae	9.45673	Metagenomic
136	WW_2ndary_184	Campylobacteraceae	5.33394	Metagenomic
137	WW_2ndary_184	Pseudomonadaceae	5.21830	Metagenomic
138	WW_2ndary_184	Mycobacteriaceae	4.67294	Metagenomic
139	WW_2ndary_184	Moraxellaceae	3.70962	Metagenomic
140	WW_2ndary_184	Cloacimonetes_noname	2.98043	Metagenomic
141	WW_2ndary_184	Aeromonadaceae	2.28704	Metagenomic
143	WW_2ndary_184	Peptostreptococcaceae	2.20501	Metagenomic
144	WW_2ndary_184	Dermatophilaceae	1.20485	Metagenomic
145	WW_2ndary_184	Neisseriaceae	1.17247	Metagenomic

Amplicon data

In [4]:
```
#new version
ps_amplicon_perc <- readRDS(file="/SCIENCE/Nelson_lab/write-ups/EPseq_paper/revised/amplicon_data_for_metagenomes_deseq.rds")

ps_amplicon_filt <- filter_taxa(ps_amplicon_perc, filterfun(kOverA(1, .05)), TRUE) #where number of samples=1, min_perc=1

#get taxa table
amplicon_tax <- as.data.frame(tax_table(ps_amplicon_filt))

#replace "NA" in taxonomy with higher taxonomy
amplicon_tax$Family <- ifelse(is.na(amplicon_tax$Family), as.character(amplicon_tax$Order), as.character(amplicon_tax$Family))
amplicon_tax$Family <- ifelse(is.na(amplicon_tax$Family), as.character(amplicon_tax$Phylum), as.character(amplicon_tax$Family))
amplicon_family <- subset(amplicon_tax, select=c("Family"))

#get otu_table
amplicon_abund <- t(as.data.frame(otu_table(ps_amplicon_filt)))

#merge otu and taxa tables
amplicon.fam <- merge(amplicon_abund, amplicon_family, by=0)

#merge otu and taxa tables
amplicon.fam <- merge(amplicon_abund, amplicon_family, by=0)
amplicon.fam
```

Row.names
CATTAATCGGAATGATTGGGCGTAAAGGGCGCGTAGGCGGGATATAAAGTTAGATGTGAAATTCTGAAGCTCAACTTCAGAGCTGCATTTAATACTCATATTCTTGAGGGATGGCGGAGAAAACGGAATTCCACGTGTAGCGGTGA
CATTAATCGGAATTATTGGGCGTAAAGGGCGCGTAGGCGGATAGGAAAGTCAGATGTGAAATTTCGGAGCTCAACTTCGAAGCTGCATTTGAAACTACTTGTCTAGAGGATAGGCGGAGAAAACGGAATTCCACGTGTAGCGGT
CATTAATCGGATTTATTGGGCGTAAAGGGCGCGTAGGCGGATGGGTAAGTCAGATGTGAAATTCCGAAGCTCAACTTCGGAGCTGCATTTGAAACTACTTATCTAGAGGAGTGGCGGAGAAAATGGAATTCCACGTGTAGCGGT
CATTAATCGGATTTATTGGGCGTAAAGGGCGCGTAGGCGGGAAAGTCAGTCAGATGTGAAAGCCCAGGGCTCAACCCTGGAACAGCATTTGAAACTACTTTTCTAGAGGGTAGGCGGAGAAAACAGAATTCCACGTGTAGCGG
CATTAATCGGATTTATTGGGCGTAAAGGGCGCGTAGGCGGGAAAGTCAGTCAGATGTGAAAGCCCAGGGCTCAACCCTGGAACAGCATTTGAAACTACTTTTCTAGAGGGTAGGCGGAGAAAACAGAATTCCACGTGTAGCGG
CATTACCCGGAATCATTGGGCGTAAAGCGTCCGCAGGCGGCGCAGTAAGTCAGGTATTAAATCTTAAGGCTCAACTTTAAGGCTGTACTTGAAACTACTGTGCTAGAGTTTTGGAGAGGTGAGCGGAATTCTACATGTAGGGGTA
CATTACCCGGATTTATTGGGCGTAAAGAGTTCGTAGGCGGCAAGATAAGTCAGATATTAAATCCTGAGGCTCAACTTCAGGCCTGTATCTGAAACTGTCTCGCTAGAGTCTTGGAGAGGTGAGTGGAATTCCTCATGTAGGGGTAA
CATTACCCGGATTTATTGGGCGTAAAGAGTTCGTAGGCGGCGAGGTAAGTCAGATATTAAATCCTGAGGCTCAACTTCAGGTCTGTATCTGAAACTGTCTTGCTAGAGTCTTGGAGAGGTGAGTGGAATTCCTCATGTAGGGGTAA
CATTACTCGGAATCACTGGGCGTAAAGAATGCGTAGGCGGCTTAGTAAGTTATTAATGAAATACCGGGGCTCAACTTCGGTGCTGTTAATAATACTGCTTCGCTAGAGTTTGGAAGAGGGAAGTGGAATTCCTAGTGTAGGGGTAA
CATTATTCGGATTTATTGGGCGTAAAGGGTCCGTAGGCGGTTTGATAAGTCAGGTGTGAAATCCCACAGCTTAACTGTGGACACGCATTTGATACTGTCAGACTAGAGTATGAGAGGGGTAAAAGGAATTTCCAGTGTAGGGGTGA
CGTTAATCGGAATAACTGGGCGTAAAGCGCACGCAGGCGGTTAGATAAGTCAGATGTGAAAGCCCCGGGCTCAACCTGGGAACTGCATTTGAAACTGTCTGACTAGAGTCTTGTAGAGGGGGGTAGAATTCCAGGTGTAGCGG
CGTTAATCGGAATAACTGGGCGTAAAGGGCGCGTAGGCGGGTATATAAGTTAGATGTGAAAGGCCTGGGCTTAACCTAGGTTGGTCATTTAATACTGTATATCTAGAGTGAAGTAGAGGGCAGTAGAATTCCCGGAGTAGCGGTGA
CGTTAATCGGAATGACTGGGCGTAAAGGGCGCGTAGGCGGTAGAGAGGGTGTGATGTGAAAGCCCTGGGCTTAACCTGGGAAGGGCATCGCAAACTGTTCTGCTAGAGTAGATGAGAGGGTGGCGGAATTTCCGGAGTAGC
CGTTAATCGGAATGACTGGGCGTAAAGGGCGCGTAGGCGGTAGAGAGGGTGTGATGTGAAAGCCCTGGGCTTAACCTGGGAAGGGCATCGCAAACTGTTCTGCTAGAGTAGATGAGAGGGTGGCGGAATTTCCGGAGTAGC
CGTTAATCGGAATGACTGGGCGTAAAGGGCGCGTAGGTGGTTGATTAAGTTGGATGTGAAATCCCTGGGCTTAACCTAGGAACTGCATTCAATACTGGTTAACTTGAGTATGGGAGAGGGTAGTGGAATTTCCGGTGTAGCGGTG
CGTTAATCGGAATGACTGGGCGTAAAGGGTGCGTAGGTGGCTGTTTAAGTTAGATGTGAAATCCCCGGGCTTAACCTGGGAACTGCATTTAATACTGGACGGCTAGAGTGTGGGAGAGGTAAGTGGAATTTCCGGTGTAGCGGT
CGTTAATCGGAATTACTGGGCGTAAAGCGAGCGCAGACGGTTACTTAAGCAGGATGTGAAATCCCCGGGCTTAACCTGGGAACGGCGTTCTGAACTGGGTAACTAGAGTATGTCAGAGGGGGGTAGAATTCCACGTGTAGCAG
CGTTAATCGGAATTACTGGGCGTAAAGCGCACGCAGGCGGTCTGTCAAGTCGGATGTGAAATCCCCGGGCTCAACCTGGGAACTGCATTCGAAACTGGCAGGCTAGAGTCTTGTAGAGGGGGGTAGAATTCCAGGTGTAGCG
CGTTAATCGGAATTACTGGGCGTAAAGCGCACGCAGGCGGTCTGTCAAGTCGGATGTGAAATCCCCGGGCTCAACCTGGGAACTGCATTCGAAACTGGCAGGCTGGAGTCTTGTAGAGGGGGGTAGAATTCCAGGTGTAGCG
CGTTAATCGGAATTACTGGGCGTAAAGCGCACGCAGGCGGTTTGTTAAGTCAGATGTGAAATCCCCGGGCTCAACCTGGGAACTGCATCTGATACTGGCAAGCTTGAGTCTCGTAGAGGGGGGTAGAATTCCAGGTGTAGCGG
CGTTAATCGGAATTACTGGGCGTAAAGCGCACGTAGGCGGTTACTTAAGTCGGATGTGAAAGCCCTGGGCTTAACCTGGGAATTGCATCCGATACTGGGTGACTAGAGTACGGTAGAGGGGGGTGGAATTCCAGGTGTAGCGG
CGTTAATCGGAATTACTGGGCGTAAAGCGCACGTAGGCGGTTCTGTAAGTGGGATGTGAAACCCCCGGGCTTAACCTGGGAATTGCATACCAGACTGCAAAGCTAGAGTATGAGAGAGGGTGGTGGAATTTCCGGTGTAGCGG
CGTTAATCGGAATTACTGGGCGTAAAGCGCACGTAGGCGGTTTGACAAGTGGGATGTGAAAGCCCTGGGCTCAACCTGGGAACTGCATCCCAAACTGTCAGGCTAGAGTATGGTAGAGGGGGGCGGAATTCCCGGTGTAGCG
CGTTAATCGGAATTACTGGGCGTAAAGCGCACGTAGGCGGTTTGGTAAGTTGGGTGTGAAATCCCTGGGCTTAACCCGGGAATGGCATTCAAAACTGCTAGACTTGAGTGTCGTAGAGGGTAGCGGAATTCCCGGTGTAGCGG
CGTTAATCGGAATTACTGGGCGTAAAGCGCACGTAGGCTGTAGTGTAAGTCAGGGGTGAAATCCCACGGCTCAACCGTGGAACTGCCTTTGATACTGCACAACTTGAATCCGGGAGAGGGTGGCGGAATTCCAGGTGTAGGA
CGTTAATCGGAATTACTGGGCGTAAAGCGCACGTAGGTGGCTTGGTAAGTCGGATGTGAAAGCCCCGGGCTCAACCTGGGAACGGCATTCGATACTGCTGAGCTAGAGTACGAAAGAGGGGGGTGGAATTCCAGGTGTAGCG
CGTTAATCGGAATTACTGGGCGTAAAGCGCACGTAGGTGGTTTGTTAAGCTAGCTGTGAAATCCCCGGGCTCAACCTGGGCACTGCAGTTAGAACTGGCAAGCTAGAGTAGGGTAGAGGGGTGTGGAATTCCAGGTGTAGCGG
CGTTAATCGGAATTACTGGGCGTAAAGCGCACGTAGGTGGTTTGTTAAGTTGGCTGTGAAAGCCCCGGGCTCAACCTGGGAAAGCCAGCCAATACTGATAGACTAGAGTACTGTAGAGGGTAGTGGAATTTCCGGTGTAGCGGT
CGTTAATCGGAATTACTGGGCGTAAAGCGCACGTAGGTGGTTTTCTAAGCCAGCTGTGAAATCCCCGGGCTCAACCTGGGCACTGCAGTTGGAACTGGAAAGCTAGAGTAGGGTAGAGGGGTGTGGAATTCCAGGTGTAGCG
CGTTAATCGGAATTACTGGGCGTAAAGCGCGCGTAGGCGGATACTTAAGTCGGATGTGAAATCCCCGAGCTTAACTTGGGAATTGCATTCGATACTGGGTATCTGGAGTATGGTAGAGGGAAGTGGAATTTCTGGTGTAGCGGTG
⋮
CGTTGTTCGGAATTACTGGGCGTAAAGGGCGCGTAGGCGGATTAGTAAGTTGGAAGTGAAAGCCCGGGGCTTAACCTCGGAACTGCTTTCAAAACTGCTAGTCTAGAGTGAAGTAGGGGATGGTGGAATTCCTAGTGTAGAGG
CGTTGTTCGGAATTACTGGGCGTAAAGGGCGCGTAGGCGGATTAGTAAGTTGGGAGTGAAAGCCCGGGGCTTAACCTCGGAACTGCTTTCAAAACTGCTAGTCTTGAGTGAAGTAGGGGATGATGGAATTCCTAGTGTAGAGG
CGTTGTTCGGAATTACTGGGCGTAAAGGGCGCGTAGGCGGCCAGATCAGTCGGGTGTGAAAGCCCGGGGCTCAACCCCGGAACGGCACCCGAGACGGTTTGGCTGGAGACCGGGAGAGGAGGGTGGAATAGCCAGTGTA
CGTTGTTCGGAATTACTGGGCGTAAAGGGCGCGTAGGCGGCCTTCTAAGTCGGACGTGAAAGCCCCAGGCTTAACCTGGGAACTGCGTCCGATACTGGGAGGCTTGGATTCGGGAGAGGGATGTGGAATTCCAGGTGTAGC
CGTTGTTCGGAATTACTGGGCGTAAAGGGCGCGTAGGTTGGTTTGTAAGTCTAATGTGAAATCCCCGGGCTTAACCCGGGACGTGCATTGGAAACTGCAGATCTTGAATGCTTAAGAGGGTGGTGGAATTCCAGGAGTAGAAGT
CGTTGTTCGGAATTACTGGGCTTAAAGCGCACGTAGGCGGCGCCGCAAGTGCGGGGTGAAATCCCACGGCTTACCCGTGGAACTGCCCTGCAGACTGTGGTGCTTGAGGATGGTAGGGGAGAGCGGAACTCCTGGTGGAG
CGTTGTTCGGAATTACTGGGCTTAAAGCGCACGTAGGCTGCACTGTAAGTGTCGAGTGAAATCCCACGGCTTACCCGTGGAACTGCTCGGCATACTGCGGTGCTGGAGTGTGGTAGGGGAGAGCGGAACTCCTGGTGGAGC
CGTTGTTCGGAATTACTGGGCTTAAAGCGCACGTAGGCTGTGTCGTAAGTGTCTGGTGAAATCCCACGGCTCACCCGTGGAACTGCCAGGCATACTGCGGTACTCGAGTGTGGTAGGGGAGAGCGGAACTCCTGGTGGAGC
CGTTGTTCGGAATTACTGGGCTTAAAGCGCGCGTAGGCTGCATCGTAAGTGTCTGGTGAAATCCCACGGCTCACCCGTGGAACTGCCAGGCATACTGCGATGCTAGAGTGTGGTAGGGGAGAGCGGAACTCCTGGTGGAGC
CGTTGTTCGGAATTATTGGGCGTAAAGCGCGCGCAGGCGGGTAGCAAAGTCCGATGTGAAATCCCTCGGCTCAACCGAGGACGTGCATTGGATACTGGCGACCTTGAGTCCTGGAGAGGGGAGCGGAATTTCCAGTGTAGA
CGTTGTTCGGAATTATTGGGCGTAAAGCGCGCGCAGGCGGGTGGCAAAGTCCGATGTGAAATCCCTCGGCTCAACCGAGGACGTGCATTGGATACTGGCGACCTTGAGTCCTGGAGAGGGGAGCGGAATTTCCAGTGTAGA
CGTTGTTCGGAATTATTGGGCGTAAAGCGCGCGCGGGCGGATAGGCAAGTCAGATGTGAAATCTCGAGGCTCAACTTCGAAACTGCGTCTGAAACTACCAATCTAGAATGTCGGAGGGGGAAGGGGAATATCGCATGTAGGGG
CGTTGTTCGGAATTATTGGGCGTAAAGGGCTCGCAGGTGGTTTGTTAAGTTGGTGGTTTAATCTCTGGGCTCAACCCAGAGTCAGCCATCAAAACTGGCGAACTTGAGTACGATAGGGGATAGCGGAATTCTCGGTGTAGCGGT
CGTTGTTCGGATTCACTGGGCGTAAAGGGTGCGTAGGCGGTGGGGTAAGTTTGAGGTGAAATCTCCGGGCTCAACCCGGAAATTGCCTTGAAGACTATCCTGCTAGAGGATCGGAGGGGAGACTGGAATTCTCGGTGTAGCA
CGTTGTTCGGATTCACTGGGCGTAAAGGGTGCGTAGGTGGCGGTCTAAGTCGGGTGTGAAATCTCCGGGCTCAACCCGGAGGGTGCGCTCGAAACTGGATCGCTCGAGGATGGGAGGGGAGACTGGAATTCTCGGTGTAG
CGTTGTTCGGATTCACTGGGCGTACAGGGTGTGTAGGCGGTTTGGTAAGCCTTCTGTTAAAGCTTCGGGCCCAACCCGGAAAGCGCAGAGGGTACTGCCAGGCTAGAGGGTGGGAGAGGAGCGCGGAATTCCCGGTGTAG
CGTTGTTCGGATTCACTGGGCGTACAGGGTGTGTAGGCGGTTTGGTAAGCCTTCTGTTAAAGCTTCGGGCCCAACCCGGAAAGCGCAGGGGGTACTGCCAGGCTAGAGGGTGGGAGAGGAGCGCGGAATTCCCGGTGTAG
CGTTGTTCGGATTCATTGGGCGTAAAGGGTGCGCAGGCGGCGTGGTAAGTTGAGTGTGAAATCCCGGGGCTCAACCTCGGAACTGCACTCAATACTGCCATGCTAGAGGAATGTAGAGGAGAGTGGAATTCACGGTGTAGCA
CGTTGTTCGGATTCATTGGGCGTAAAGGGTGCGCAGGCGGCGTGGTAAGTTGAGTGTGAAATCCTGAGGCTCAACCTCAGAACTGCACTCAATACTCCCACGCTAGAGGAATGTAGAGGAGAGTGGAATTCACGGTGTAGCA
CGTTGTTCGGATTCATTGGGCGTAAAGGGTGCGCAGGCGGTGTGGTAAGTCGGATGTGAAATTTAGAGGCTTAACCTCTAAACTGCATCTGATACTGCCATGCTAGAGGACTGTAGAGGAGATTGGAATTCACGGTGTAGCAGT
CGTTGTTCGGATTTACTGGGCGTAAAGAGTGCGTAGGCGGTTTGATAAGTCAGAAGTGAAAGCCCAGGGCTCAACCTTGGAATTGCTTTTGAAACTATCTTACTTGAATCCGGTAGAGGTTGGCGGAATTCTCAGTGTAGAGGTG
CGTTGTTCGGATTTACTGGGCGTAAAGCGCACGTAGGCGGCTTGTTAAGTCAGGGGTGAAATCCCGGAGCTCAACTCCGGAACTGCCTTTGATACTGGCAAGCTCGAGTGTGGAAGAGGTTGGTGGAACTGCGAGTGTAGAG
CGTTGTTCGGATTTACTGGGCGTAAAGGGTGCGTAGGCGGCGCGGAAAGTTCGGGGTGAAATCTCCAGGCTCAACCTGGAAACTGCCCTGAATACTTCCGTGCTCGAGGGCCGCAGAGGAGATTGGAATTCTCGGTGTAGC
CGTTGTTCGGATTTATTGGGCGTAAAGCGCGCGCAGGCGGATTTGTAAGTCAGGTGTGAAATCTCGAGGCTCAACCTCGAAACTGCGCCTGAAACTGTGAATCTAGAATATGGTAGGGGGAAGGGGAATTTCACGTGTAGGGG
CGTTGTTCGGATTTATTGGGCGTAAAGCGCGCGTAGGCGGACCTGTAAGTCAGATGTGAAATCTCGGTGCTCAACACCGAAACTGCGTCTGAAACTGCGGGTCTTGAATCTTAGAGGGGGAGTGGGAATTTCGCATGTAGGGG
TGGCTATCGCGATTATTGGGCCTAAAGCGTCCGTAGTCGGTTCTGTACATCCTCTGTGAAATCGCAGCGCTCAACGTTGCGGCATGCGGGGGACACGGCAGAGCTTGAGACCGGGAGGCGTGTAGGGTATTCCACGGGGAC
TGTTACTCAACTTGACTGGGCGTAAAGGGCGTGAAGGTGGTACAGTGCGTTTTTTTGTAAAAACTCAAACATAATTTGAGGGACTAAAAAATACGGCTGTGCTTGAGTTTACAGGAAGAAAGTTGTACTTCTAGTGTAGAGGTGAA
TGTTACTCATCAATACTGGGCGTAAAGCGCATGTAGGCGGTTTATGATGTCAGAATAAAAATCTAGAGATCACCTTTATATTAATATTTTTAAACTAATAAACTAAGAGTATAAGAGAAGTGTGTTGCACTTTCAGTGGAGAGGTAAAATA
TGTTATCCGGATTTATTGGGCGTAAAGCGTCCGCAGATTGTTTGACGGGTCAGGGGTTAAAATCCAGTGCTTAACACTGGAATCGCCTTTGAAACTATCAAGCTAGAGGGTGGAAGAGGTAAGCGGAATTGCAGGTGTAGTCGTA
TGTTATTCGTCTTAATGGGTGTAAAACGCGTGTAGATGGGATATCGAGTTTAACTTTAAATTTTTATAATATATTATAAAAAAAGTTTTAATACAGTTATTCTTGAGTGCTTATAAAACAACAGTATTTTAAGTGTAAGATGACATTTTAAAATAT

In [5]:
```
colnames(amplicon.fam) <- c("amplicon_sequence",
    "WW_2ndary_152", "WW_2ndary_206", "GAC_filt1_206", "GAC_filt2_206", "GAC_filt3_206",
    "SDS_1_205", "SDS_2_205", "WW_2ndary_184",
    "Family")
amplicon.fam <- subset(amplicon.fam, select=-c(amplicon_sequence)) #remove this column
amplicon.fam.melt <- melt(amplicon.fam, id.vars = c("Family"),
                          variable.name = "Sample_code", value.name = "Relative_abundance")

#collapse multiple amplicons from same family in each sample into one entry
amplicon.fam.melt <- amplicon.fam.melt %>%
    group_by(Sample_code, Family) %>%
    summarize(Relative_abundance=sum(Relative_abundance))

amplicon.fam.melt$Sample_code <- factor(amplicon.fam.melt$Sample_code, levels=c(
    "WW_2ndary_152", "WW_2ndary_184", "WW_2ndary_206",
    "GAC_filt1_206", "GAC_filt2_206", "GAC_filt3_206",
    "SDS_1_205", "SDS_2_205"))

#filter out low abundance families to make visualization easier

Sequencing <- rep("Amplicon", length(amplicon.fam.melt$Family))
amplicon.fam.melt$Sequencing <- Sequencing

amplicon.fam.melt
```

Sample_code	Family	Relative_abundance	Sequencing
WW_2ndary_152	0319-6G20	0.18508726	Amplicon
WW_2ndary_152	195up	0.03819261	Amplicon
WW_2ndary_152	34P16	0.17333568	Amplicon
WW_2ndary_152	Acidaminococcaceae	0.10282625	Amplicon
WW_2ndary_152	Acidimicrobiales_Incertae_Sedis	0.19977672	Amplicon
WW_2ndary_152	Acidobacteria	0.00000000	Amplicon
WW_2ndary_152	Actinobacteria	0.00000000	Amplicon
WW_2ndary_152	Actinomycetaceae	0.05288207	Amplicon
WW_2ndary_152	Aeromonadaceae	0.56554439	Amplicon
WW_2ndary_152	Alcaligenaceae	0.09841941	Amplicon
WW_2ndary_152	Alphaproteobacteria_Incertae_Sedis	0.07932311	Amplicon
WW_2ndary_152	Bacteriovoracaceae	0.07197838	Amplicon
WW_2ndary_152	Bacteroidaceae	0.40249133	Amplicon
WW_2ndary_152	Bacteroidetes	0.05288207	Amplicon
WW_2ndary_152	Bdellovibrionaceae	0.10429520	Amplicon
WW_2ndary_152	Bifidobacteriaceae	0.24531406	Amplicon
WW_2ndary_152	Bradymonadales	0.15130149	Amplicon
WW_2ndary_152	Bradyrhizobiaceae	0.00000000	Amplicon
WW_2ndary_152	Burkholderiaceae	0.14983254	Amplicon
WW_2ndary_152	Caenarcaniphilales	0.06904048	Amplicon
WW_2ndary_152	Caldilineaceae	0.17627358	Amplicon
WW_2ndary_152	Campylobacteraceae	0.21005935	Amplicon
WW_2ndary_152	Carnobacteriaceae	0.10870204	Amplicon
WW_2ndary_152	Caulobacteraceae	0.06316470	Amplicon
WW_2ndary_152	Cellvibrionaceae	0.34960926	Amplicon
WW_2ndary_152	Chitinophagaceae	0.09401257	Amplicon
WW_2ndary_152	Chlamydiales	0.10576415	Amplicon
WW_2ndary_152	Chloroflexi	0.07785416	Amplicon
WW_2ndary_152	Chthoniobacterales_Incertae_Sedis	0.05581997	Amplicon
WW_2ndary_152	Clostridiaceae_1	0.22768670	Amplicon
⋮	⋮	⋮	⋮
WW_2ndary_184	Rickettsiaceae	0.04794094	Amplicon
WW_2ndary_184	Rickettsiales_Incertae_Sedis	0.12224939	Amplicon
WW_2ndary_184	Rikenellaceae	0.04554389	Amplicon
WW_2ndary_184	Ruminococcaceae	0.19655784	Amplicon
WW_2ndary_184	Saccharibacteria	0.36674817	Amplicon
WW_2ndary_184	Saprospiraceae	0.24210173	Amplicon
WW_2ndary_184	SBR1093	0.01438228	Amplicon
WW_2ndary_184	SC-I-84	0.00000000	Amplicon
WW_2ndary_184	Simkaniaceae	0.12464644	Amplicon
WW_2ndary_184	SM2D12	0.05273503	Amplicon
WW_2ndary_184	Solirubrobacterales	0.08869073	Amplicon
WW_2ndary_184	Sphingobacteriales	0.17977851	Amplicon
WW_2ndary_184	Sphingomonadaceae	0.04314684	Amplicon
WW_2ndary_184	Sphingomonadales	0.03595570	Amplicon
WW_2ndary_184	Spirochaetaceae	0.05513208	Amplicon
WW_2ndary_184	Spongiibacteraceae	0.00000000	Amplicon
WW_2ndary_184	SR1_(Absconditabacteria)	0.07670550	Amplicon
WW_2ndary_184	Streptococcaceae	0.13902872	Amplicon
WW_2ndary_184	Thiotrichaceae	0.01198523	Amplicon
WW_2ndary_184	TM146	0.08869073	Amplicon
WW_2ndary_184	TM6_(Dependentiae)	6.50318807	Amplicon
WW_2ndary_184	Unknown_Family	3.88081883	Amplicon
WW_2ndary_184	Veillonellaceae	0.11745530	Amplicon
WW_2ndary_184	Verrucomicrobia	0.05033798	Amplicon
WW_2ndary_184	Verrucomicrobiaceae	0.29483676	Amplicon
WW_2ndary_184	Xanthobacteraceae	0.01438228	Amplicon
WW_2ndary_184	Xanthomonadaceae	0.28045448	Amplicon
WW_2ndary_184	Xanthomonadales_Incertae_Sedis	0.24929287	Amplicon
WW_2ndary_184	Z4MB62	0.00000000	Amplicon
WW_2ndary_184	NA	1.96078431	Amplicon

Combine amplicon and metaphlan data

In [6]:
```
#combine sequencing types into one dataframe to plot
amp.meta.combined <- bind_rows(amplicon.fam.melt, metaphlan.fam)
amp.meta.combined$Sample_code <- factor(amp.meta.combined$Sample_code, levels=c(
    "WW_2ndary_152", "WW_2ndary_184", "WW_2ndary_206",
    "GAC_media1_234", "GAC_media2_234", "GAC_media3_234",
    "GAC_filt1_206", "GAC_filt2_206", "GAC_filt3_206",
    "SDS_1_205", "SDS_2_205", "SDS_3_205", "Zymo_mock_DNA"))


#make additional column with "Type" so we can separate plots by type
#(Too many different families to use custom colors and plot all sample Types together)
sample2type <- data.frame(
    Sample_code=c(
    "WW_2ndary_152", "WW_2ndary_184", "WW_2ndary_206",
    "GAC_media1_234", "GAC_media2_234", "GAC_media3_234",
    "GAC_filt1_206", "GAC_filt2_206", "GAC_filt3_206",
    "SDS_1_205", "SDS_2_205", "SDS_3_205",
    "Zymo_mock_DNA"),
    Type=c(
    "WW_2ndary", "WW_2ndary", "WW_2ndary",
    "GAC_media", "GAC_media", "GAC_media",
    "GAC_filt", "GAC_filt", "GAC_filt",
    "SDS", "SDS", "SDS",
    "Mock"))
amp.meta.combined <- merge(amp.meta.combined, sample2type, by="Sample_code", all.x=TRUE)

#order for plotting
amp.meta.combined$Type <- factor(amp.meta.combined$Type, levels=c("WW_2ndary", "GAC_media", "GAC_filt", "SDS", "Mock"))
```

```
Warning message in bind_rows_(x, .id):
“Unequal factor levels: coercing to character”Warning message in bind_rows_(x, .id):
“binding character and factor vector, coercing into character vector”Warning message in bind_rows_(x, .id):
“binding character and factor vector, coercing into character vector”Warning message in bind_rows_(x, .id):
“binding character and factor vector, coercing into character vector”
```

In [7]:
```
amp.meta.combined.nomock <- amp.meta.combined[amp.meta.combined$Sample_code!='Zymo_mock_DNA',]
#amp.meta.combined.mock <- amp.meta.combined[amp.meta.combined$Sample_code=='Zymo_mock_DNA',]

#filter out low abundance families to make visualization easier
amp.meta.combined.nomock <- amp.meta.combined.nomock[amp.meta.combined.nomock$Relative_abundance>=2,]

options(repr.plot.width = 8, repr.plot.height = 4)
ggplot(amp.meta.combined.nomock, aes(x=Sample_code, y=Relative_abundance, fill=Family))+
    geom_bar(stat="identity", color="black")+
    scale_fill_manual(values = colors)+
    theme(panel.background=element_blank(), panel.border=element_rect(color = "black", fill = NA),
    axis.text.x = element_text(angle = 45, hjust = 1))+
    xlab("")+
    ylab("Percent Relative Abundance")+
    facet_grid(Sequencing~Type, scales="free_x")+
    guides(fill=guide_legend(ncol=2))
#ggsave("/SCIENCE/Nelson_lab/write-ups/EPseq_paper/revised/figures/metaphlan2.vs.amplicon.pdf", device="pdf", width=8, height=4)
```

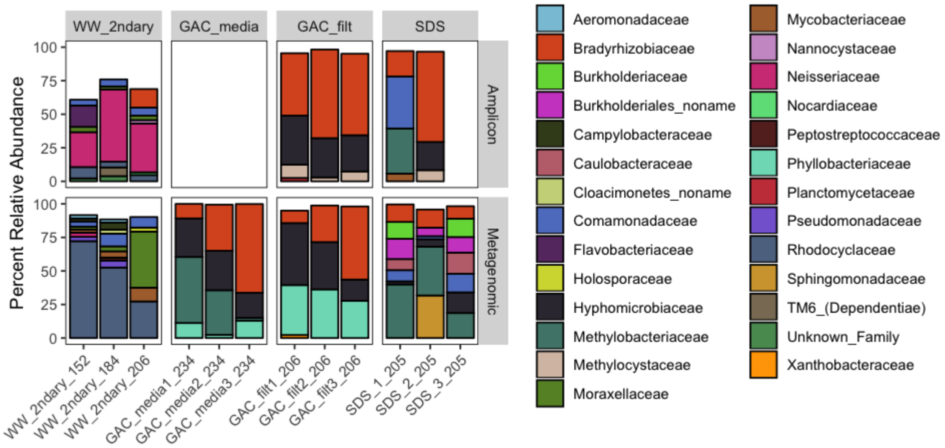

Mock community expected vs. theoretical relative abundance based on binning and percent relative abundance in Anvi'o

In [8]:
```
mock1_actual <- read.table("/SCIENCE/Nelson_lab/data_files_nelson/el_paso_metagenomics/anvio_work/sample_200/relative_abund_mock1.txt", sep="\t", header=TRUE)
zymo_DNA_theoretical <- read.table("/SCIENCE/Nelson_lab/data_files_nelson/el_paso_16S/zymobiomics_DNA_standard_theoretical_comp.txt", sep="\t", header=TRUE)
expect.v.actual <- merge(mock1_actual, zymo_DNA_theoretical, by="Genus")
expect.v.actual <- subset(expect.v.actual, select=-theoretical_16S)
melt.expect.v.actual <- melt(expect.v.actual, id.vars = "Genus", variable.name = "Expect_or_Actual", value.name = "Percent")

melt.expect.v.actual$Genus <- factor(melt.expect.v.actual$Genus,
                                     levels=c("Bacillus", "Enterococcus", "Escherichia",
                                              "Lactobacillus", "Listeria", "Pseudomonas",
                                               "Salmonella", "Staphylococcus",
                                              "Cryptococcus", "Saccharomyces"))

options(repr.plot.width = 3, repr.plot.height = 4) #for plotting size in jupyter
ggplot(melt.expect.v.actual, aes(x=Expect_or_Actual, y=Percent, fill=Genus))+
    geom_bar(stat="identity", color="black")+
    scale_fill_manual(values = colors)+
    theme(panel.background=element_blank(), panel.border=element_rect(color = "black", fill = NA),
        axis.text.x = element_text(angle = 45, hjust = 1, vjust = 1))+
    scale_x_discrete(labels=c("Mock1", "Theoretical"))+
    xlab("Sample")
#ggsave("/SCIENCE/Nelson_lab/data_files_nelson/el_paso_metagenomics/mock_community_theoretical_metagenomics.pdf", device = "pdf", height=4, width=3)
```

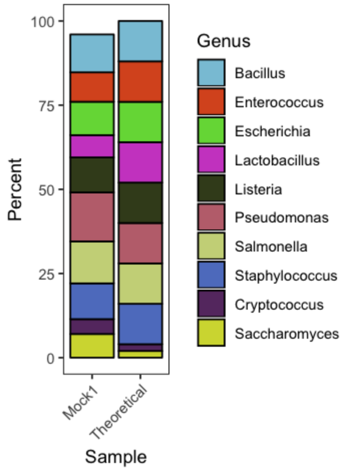

Sample cross-mapping to examine overlaps between communities

In [9]:
```
read_counts_m1_F4 <- read.table("/SCIENCE/Nelson_lab/data_files_nelson/el_paso_metagenomics/cross_map_min1000/all.read_counts_m1_F4.txt", header=TRUE)
metagenome_info <- read.table("/SCIENCE/Nelson_lab/data_files_nelson/el_paso_metagenomics/metagenome_info.txt", header=TRUE, sep="\t")

#get prefered ordering of samples
metagenome_info$Sample_code <- factor(metagenome_info$Sample_code, levels=c(
'WW_2ndary_152', 'WW_2ndary_184', 'WW_2ndary_206',
'GAC_media1_234', 'GAC_media2_234', 'GAC_media3_234',
'GAC_filt1_206', 'GAC_filt2_206', 'GAC_filt3_206',
'SDS_1_205', 'SDS_2_205', 'SDS_3_205', "Zymo_mock_DNA"))

metagenome_info <- subset(metagenome_info, select=c(Sample_code, Sample_number, Total_reads))
sample_names <- subset(metagenome_info, select=c(Sample_code, Sample_number))

#remove mapping counts from sample_1 and sample_9 and use the retrimmed sample_1 and sample_9 values instead
read_counts_m1_F4 <- read_counts_m1_F4[read_counts_m1_F4$reads!="sample_1",]
read_counts_m1_F4 <- read_counts_m1_F4[read_counts_m1_F4$reads!="sample_9",]
read_counts_m1_F4[read_counts_m1_F4$reads=="sample_1_retrimmed",3] <- "sample_1"
read_counts_m1_F4[read_counts_m1_F4$reads=="sample_9_retrimmed",3] <- "sample_9"

#get sample_codes for reads
read_counts_m1_F4 <- merge(read_counts_m1_F4, metagenome_info, by.x = "reads", by.y = "Sample_number")
names(read_counts_m1_F4)[names(read_counts_m1_F4)=="Sample_code"] <- "reads_sample_code"
read_counts_m1_F4 <- merge(read_counts_m1_F4, sample_names, by.x="assembly", by.y="Sample_number")
names(read_counts_m1_F4)[names(read_counts_m1_F4)=="Sample_code"] <- "assembly_sample_code"
read_counts_m1_F4$percent_mapped <- read_counts_m1_F4$count*100/read_counts_m1_F4$Total_reads
```

In [10]:
```
options(repr.plot.width = 8, repr.plot.height = 7)
ggplot(read_counts_m1_F4, aes(reads_sample_code, assembly_sample_code)) +
    geom_tile(aes(fill = percent_mapped)) +
    geom_text(aes(label = round(percent_mapped, 2)), size=3) +
    scale_y_discrete(limits = rev(levels(read_counts_m1_F4$assembly_sample_code)))+ #reverse order of y axis
    scale_fill_gradient(low = "white", high = "red", name="Percent\nReads\nMapped")+
    xlab("Sample reads")+
    ylab("Sample assemblies")+
    theme(panel.background=element_blank(), panel.border=element_rect(color = "black", fill = NA),
        axis.text.x = element_text(angle = 45, hjust = 1, vjust = 1))
#ggsave("/SCIENCE/Nelson_lab/data_files_nelson/el_paso_metagenomics/cross_map_min1000/crossmapping_figure_m1F4.pdf", device="pdf", width=8, height=7)
```

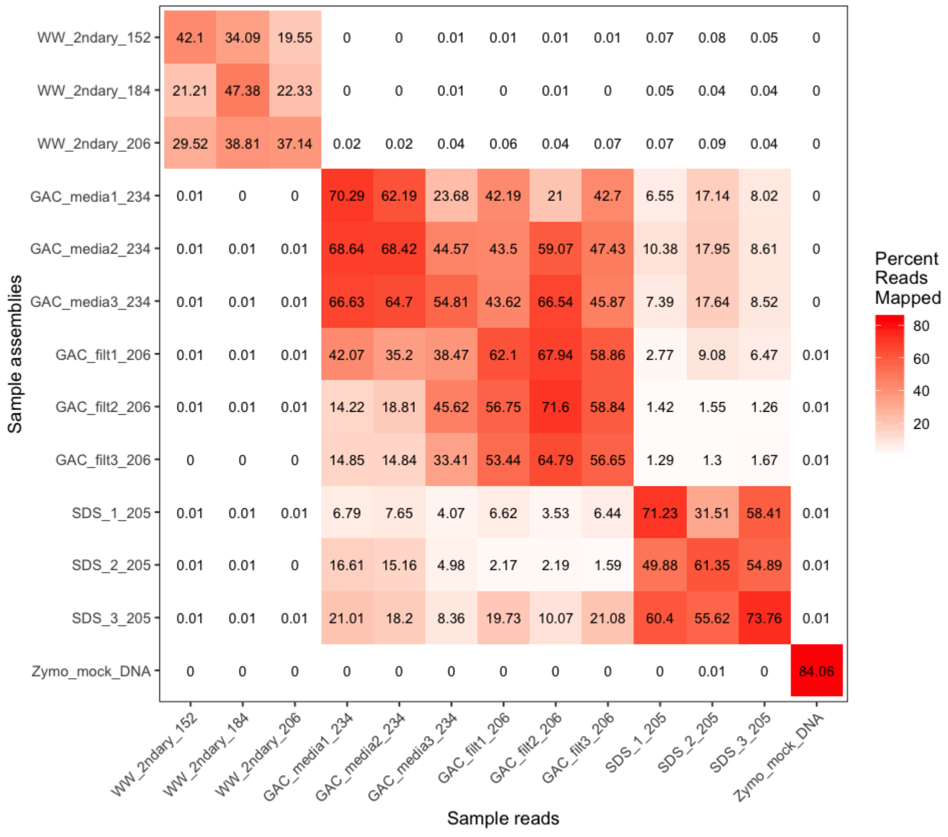

RPS3 abundance analyses

In [11]:
```
#import coverages and breadths

#using Q2Q3 coverage rather than mean because it is not as influenced by extreme high and low values
rps3_ab <- read.table(
    "/SCIENCE/Nelson_lab/data_files_nelson/el_paso_metagenomics/rps3_analyses/mean_coverage_Q2Q3_contigs.txt",
    sep="\t", header=TRUE)
row.names(rps3_ab) <- rps3_ab$X__parent__
rps3_ab <- subset(rps3_ab, select=-c(X__parent__, contig))

#filter on detection (aka breadth- how much of contig is at 1x coverage)
#note that read-mapping files were already filtered to allow only 1 mismatch between mapped reads and contigs
rps3_breadth <- read.table(
    "/SCIENCE/Nelson_lab/data_files_nelson/el_paso_metagenomics/rps3_analyses/detection_splits.txt",
    sep="\t", header=TRUE)
row.names(rps3_breadth) <- rps3_breadth$X__parent__
rps3_breadth <- subset(rps3_breadth, select=-c(X__parent__, contig))

#Create mask to use as filter- set breadth threshold)
rps3_mask <- rps3_breadth >= 0.65 #0.75
rps3_ab_masked <- replace(rps3_ab, !rps3_mask, 0)

#transpose to generate standard OTU table (samples are rows, RPS3-containing scaffolds are columns)
trps3_ab <- t(rps3_ab_masked)

#convert sample names
sample_name_lookup <- data.frame(old_name=c('awtp_infl_152', 'awtp_infl_184', 'awtp_infl_206',
                                            'effl_catreagg_206', 'effl_coconut_206', 'effl_reagg_206',
                                            'media_catreagg_234', 'media_coconut_234', 'media_reagg_234',
                                            'reactor_catreagg_205', 'reactor_coconut_205', 'reactor_reagg_205'),
                                new_name=c('WW_2ndary_152', 'WW_2ndary_184', 'WW_2ndary_206',
                                            'GAC_filt1_206', 'GAC_filt2_206', 'GAC_filt3_206',
                                            'GAC_media1_234', 'GAC_media2_234', 'GAC_media3_234',
                                            'SDS_1_205', 'SDS_2_205', 'SDS_3_205'))
trps3_ab <- merge(sample_name_lookup, trps3_ab, by.x="old_name", by.y=0, all.y=TRUE)
row.names(trps3_ab) <- trps3_ab$new_name
trps3_ab <- subset(trps3_ab, select=-c(old_name, new_name))
```

In [12]:
```
#merge with total reads info for normalization
metagenome_info <- read.table("/SCIENCE/Nelson_lab/data_files_nelson/el_paso_metagenomics/metagenome_info.txt", header=TRUE, sep="\t", row.names=1)
total_reads <- subset(metagenome_info, select=c(Total_reads))
trps3_ab <- merge(trps3_ab, total_reads, by = 0) #merge total reads for normalizing
row.names(trps3_ab) <- trps3_ab$Row.names

#get prefered ordering of samples
trps3_ab$Row.names <- factor(trps3_ab$Row.names, levels=c(
'WW_2ndary_152', 'WW_2ndary_184', 'WW_2ndary_206',
'GAC_media1_234', 'GAC_media2_234', 'GAC_media3_234',
'GAC_filt1_206', 'GAC_filt2_206', 'GAC_filt3_206',
'SDS_1_205', 'SDS_2_205', 'SDS_3_205'))
trps3_ab <- trps3_ab[order(trps3_ab$Row.names),]
trps3_ab <- subset(trps3_ab, select=-c(Row.names))

#normalize to total read depth
rps3_norm <- 10000000*subset(trps3_ab, select=-c(Total_reads))/trps3_ab$Total_reads
rps3_norm <- as.matrix(rps3_norm) #convert to matrix
rps3_norm
```

	sample_185_scaffold_309	sample_185_scaffold_999	sample_187_scaffold_1956	sample_187_scaffold_2018	sample_187_scaffold_8907	sample_187_scaffold_99	sample_193_scaffol
**WW_2ndary_152**	0.0000000	0.0000000	0.0000000	0.0000000	0.0000000	0.0000000	0.0000000
**WW_2ndary_184**	0.0000000	0.0000000	0.0000000	0.0000000	0.0000000	0.0000000	0.0000000
**WW_2ndary_206**	0.0000000	0.0000000	0.0000000	0.0000000	0.0000000	0.0000000	0.0000000
**GAC_media1_234**	23.1764956	1.4480663	0.0000000	91.6429288	0.0000000	0.5522718	0.0000000
**GAC_media2_234**	19.6322231	10.3434145	0.8981588	71.8963129	0.4936819	2.3544341	0.5067758
**GAC_media3_234**	28.1944104	0.7753804	0.0000000	6.7842386	4.9344971	4.4265683	1.3274809
**GAC_filt1_206**	78.5690411	0.0000000	0.0000000	1.0698373	2.8279888	0.0000000	0.7069758
**GAC_filt2_206**	43.1759791	0.0000000	0.0000000	0.0000000	4.7568158	0.0000000	0.0000000
**GAC_filt3_206**	80.4375560	0.0000000	0.0000000	0.3488985	2.4960147	0.0000000	0.0000000
**SDS_1_205**	0.6872464	0.0000000	14.3240751	0.7144817	0.0000000	0.0000000	0.0000000
**SDS_2_205**	0.0000000	0.0000000	0.0000000	30.0098755	0.0000000	0.0000000	0.0000000
**SDS_3_205**	0.9719412	0.0000000	2.6483517	19.9442767	0.0000000	0.0000000	0.0000000

In [13]:
```
#add taxonomic info, based on BLAST best-hit of RPS3 protein to NCBI-NR:
genera <- read.table("/SCIENCE/Nelson_lab/data_files_nelson/el_paso_metagenomics/rps3_analyses/besthit-nr_genus.txt",
                    header=TRUE, sep="\t", row.names = 1)
```

In [14]:
```
#clustering samples and scaffolds for clustered heatmap (make sure NAs are zeros)

#cluster samples by bray curtis (first turn NA to zero)
rps3_norm_for_clust <- ifelse(is.na(rps3_norm), 0, rps3_norm)

#cluster scaffolds by occurrence, using Spearman rank correlation converted to distance matrix
corrdist_dissimilarity <- as.dist(1 - cor(rps3_norm_for_clust, method="spearman"))
scaff_clust <- hclust(corrdist_dissimilarity)
```

In [15]:
```
#Log-scale and plot heatmaps
rps3_norm <- ifelse(rps3_norm==0, NA, rps3_norm) #make sure zeros are NAs for non-detects
rps3.logabundance <- log10(rps3_norm)

options(repr.plot.width = 4.5, repr.plot.height = 7)

pheatmap(t(rps3.logabundance),
         color=rev(magma(15)[1:14]),
         #cluster_cols=sample_clust,
         cluster_cols=FALSE,
         cluster_rows=scaff_clust,
         na_col="white",
         labels_row=as.character(genera$tax),
         #labels_col=as.character(metagenome_info$Sample_code), #to change col names
         fontsize_row=7, fontsize_col=7,
         treeheight_row=30)

#save as pdf (uncomment these lines)
        # filename="/SCIENCE/Nelson_lab/data_files_nelson/el_paso_metagenomics/rps3_analyses/rps3_heatmap.pdf", width=4.5, height=7)
```

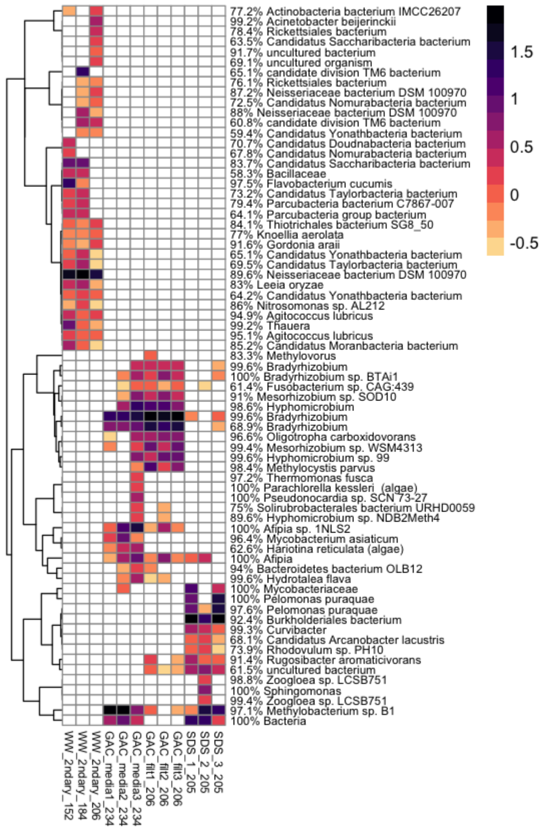

How abundant is the least abundant detected RPS3? On the order of 0.01 - 0.05 % of the total detected community

In [16]:
```
rps3_ab_perc_scaled <- scale(rps3_ab, center=FALSE, scale=colSums(rps3_ab))
#minimum non-zero value of each column (sample)- did this by first turning zeros to 100; there's probably a better way.
least_abund_rps3 <- 100*apply(ifelse(rps3_ab_perc_scaled>0, rps3_ab_perc_scaled, 100),2,min)
least_abund_rps3
```

**awtp_infl_152**	0.0387691216647561
**awtp_infl_184**	0.00208017241789528
**awtp_infl_206**	0.00136857642967486
**effl_catreagg_206**	0.0178059552319194
**effl_coconut_206**	0.02634443858378
**effl_reagg_206**	0.0294596964185749
**media_catreagg_234**	0.0184474726181787
**media_coconut_234**	0.0169709071264682
**media_reagg_234**	0.0754718697074531
**reactor_catreagg_205**	0.0160457476708192
**reactor_coconut_205**	0.017449326784259
**reactor_reagg_205**	0.0490337013983156

Metagenomic abundance data - using MAGs

In [17]:
```
#load data, from bam files parsed with Anvi'o
meta_ab <- read.table("/SCIENCE/Nelson_lab/data_files_nelson/el_paso_metagenomics/anvio_work/dereplication/anvio_dRep_MAGs_summary/mean_coverage_Q2Q3.txt", sep=
"\t", header=TRUE, row.names=1)

#filter on detection (aka breadth- how much of genome is at 1x coverage)
#read-mapping was filtered to include reads with 1 mismatch or less.
breadth <- read.table("/SCIENCE/Nelson_lab/data_files_nelson/el_paso_metagenomics/anvio_work/dereplication/anvio_dRep_MAGs_summary/detection.txt", sep="\t", head
er=TRUE, row.names=1)

#breadth <- read.table("/SCIENCE/Nelson_lab/data_files_nelson/el_paso_metagenomics/anvio_work/dereplication_OLD/mags_summary/bins_across_samples/detection.txt",
 sep="\t", header=TRUE, row.names=1)
mask <- breadth >= 0.05 #threshold is set to 5% of genome at 1x coverage
meta_ab_masked <- replace(meta_ab, !mask, 0)
```

In [18]:
```
#add taxonomy-based genome name (based on consensus of concatenated gene tree, checkM, and Centrifuge)
mag_info <- read.table("/SCIENCE/Nelson_lab/data_files_nelson/el_paso_metagenomics/anvio_work/genomes_info_091418.txt",
                       sep="\t", row.names = 1, header=TRUE)
mag_name <- mag_info["Genome_short_name"]

meta_ab_named <- merge(mag_name, meta_ab_masked, by = 0, all.y = TRUE, all.x=FALSE)
row.names(meta_ab_named) <- meta_ab_named$Genome_short_name
meta_ab_named <- subset(meta_ab_named, select=-c(Row.names, Genome_short_name))
head(meta_ab_named)
```

	GAC_filt1_206	GAC_filt2_206	GAC_filt3_206	GAC_media1_234	GAC_media2_234	GAC_media3_234	SDS_1_205	SDS_2_205	SDS_3_205	WW_2ndary_152	WW_2ndary_184	WW
**Rhizobiales_1**	42.896051	7.383023	15.97091957	0.3344239	0.004065798	2.477873	0.000000	0.00000000	0.000000	0	0	0
**Hyphomicrobium_1**	79.442379	46.975106	24.08627453	1.9522910	15.884926729	45.202817	0.000000	0.00000000	0.000000	0	0	0
**Rhizobiales_2**	22.206910	49.737862	17.57279031	0.4630752	1.924386346	40.583401	0.000000	0.00000000	0.000000	0	0	0
**Bradyrhizobiaceae_1**	6.342141	17.042300	10.03690327	0.2270441	3.578726295	12.399076	1.601318	0.02284708	2.692800	0	0	0
**Bradyrhizobiaceae_2**	232.977756	150.531394	260.94265113	59.9648610	71.426527111	89.934593	1.829865	0.93530079	3.846964	0	0	0
**Mycobacterium_1**	0.728629	2.071185	0.01869497	4.2003327	43.254839126	4.370308	0.000000	0.12885497	0.000000	0	0	0

In [19]:
```
#import iRep
irep <- read.table("/SCIENCE/Nelson_lab/data_files_nelson/el_paso_metagenomics/iRep/irep_for_heatmap.txt", sep="\t", header=TRUE, row.names=1)
row.names(irep) <- irep$Genome_short_name
irep <- subset(irep, select=-c(Genome_short_name))
```

In [20]:
```
#compile all MAG cov and iRep info for supplementary table
mag_all_info <- merge(meta_ab_named, irep, by=0, suffixes = c(".normcov", ".iRep"), all.x=TRUE, all.y=TRUE)
row.names(mag_all_info) <- mag_all_info$Row.names
mag_all_info <- select(mag_all_info, -Row.names)
dim(mag_all_info)
```

38 24

In [21]:
```
#transpose to generate standard OTU table (samples are rows, genomes are columns)
tmeta_ab <- t(meta_ab_named)
tmeta_ab <- as.data.frame(tmeta_ab)

#merge with total reads info for normalization
metagenome_info <- read.table(
    "/SCIENCE/Nelson_lab/data_files_nelson/el_paso_metagenomics/metagenome_info.txt",
    header=TRUE, sep="\t", row.names=1)
total_reads <- subset(metagenome_info, select=c(Total_reads))
tmeta_ab <- merge(tmeta_ab, total_reads, by = 0) #merge total reads for normalizing
row.names(tmeta_ab) <- tmeta_ab$Row.names

#get prefered ordering of samples
tmeta_ab$Row.names <- factor(tmeta_ab$Row.names, levels=c(
'WW_2ndary_152', 'WW_2ndary_184', 'WW_2ndary_206',
'GAC_media1_234', 'GAC_media2_234', 'GAC_media3_234',
'GAC_filt1_206', 'GAC_filt2_206', 'GAC_filt3_206',
'SDS_1_205', 'SDS_2_205', 'SDS_3_205'))
tmeta_ab <- tmeta_ab[order(tmeta_ab$Row.names),]
tmeta_ab <- subset(tmeta_ab, select=-c(Row.names))

#normalize to total read depth
meta.alldatnorm <- 10000000*subset(tmeta_ab, select=-c(Total_reads))/tmeta_ab$Total_reads
meta.alldatnorm_mat <- as.matrix(meta.alldatnorm) #convert to matrix
```

In [22]:
```
#clustering samples and genomes for clustered heatmap

#cluster samples by Bray-Curtis dissimilarity (first turn NA to zero)
meta.alldatnorm_mat_for_clust <- ifelse(is.na(meta.alldatnorm_mat), 0, meta.alldatnorm_mat)
#sample_clust <- hclust(vegdist(meta.alldatnorm_mat_for_clust, method='bray'))#makes weird clusters

#cluster genomes by occurrence, using Spearman rank correlation converted to distance matrix
corrdist_dissimilarity <- as.dist(1 - cor(meta.alldatnorm_mat_for_clust, method="spearman"))
genome_clust <- hclust(corrdist_dissimilarity)
```

In [23]:
```
options(repr.plot.width = 4.5, repr.plot.height = 6)
meta.abundance <- ifelse(meta.alldatnorm_mat==0, NA, meta.alldatnorm_mat)
meta.logabundance <- log10(meta.abundance)
pheatmap(t(meta.logabundance),
         color=rev(magma(15)[1:14]),
         cluster_rows=genome_clust,
         cluster_cols=FALSE,
         na_col="white",
         treeheight_row=30)#,#)
#save as pdf (uncomment these rows)
#filename="/SCIENCE/Nelson_lab/data_files_nelson/el_paso_metagenomics/mags_mapping_heatmap.pdf", width=4.5, height=6)

#plot iRep heatmap (combine in illustrator)
options(repr.plot.width = 2.5, repr.plot.height = 6)
colors1 = colorRampPalette(rev(brewer.pal(n = 11, name = "RdBu")))
pheatmap(irep,
         #color=rev(magma(15)[1:14]),
         cluster_rows=genome_clust,
         color=rev(viridis(10)),
         #color=colors1(10),
         cluster_cols=FALSE,
         show_rownames=FALSE,
         na_col="gray",
         treeheight_row=0)
#filename="/SCIENCE/Nelson_lab/data_files_nelson/el_paso_metagenomics/mags_iRep_heatmap.pdf", width=2.5, height=6)
```

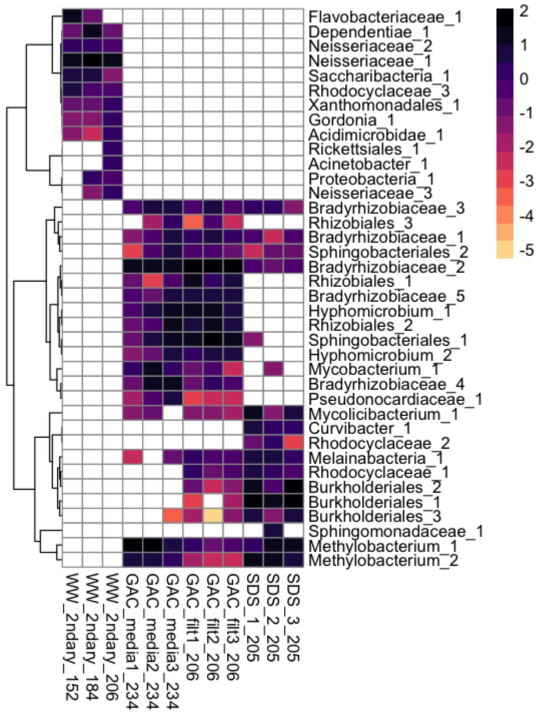

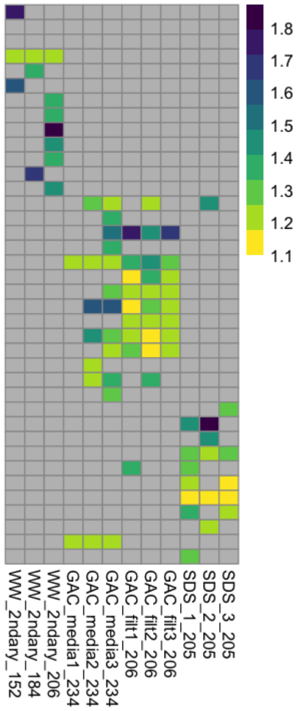

Rank abundance curves

In [24]:
```
#choose how many otus from top abundance to use (e.g. top 25 organisms in each sample)
num_rank <- 10
#melt together into 3 columns: genome, sample, abundance
meta.abundance2 <- as.data.frame(t(meta.alldatnorm))
meta.abundance2$bin <- row.names(meta.abundance2)
melted <- melt(meta.abundance2, id.vars = c("bin"), variable="Sample", value.name="Abundance")
melted$Abundance <- as.numeric(melted$Abundance)
head(melted)
#sort by sample (ascending), then by abundance (descending)
ordered <- melted[order(rev(melted$Sample), melted$Abundance, decreasing = TRUE), ]

#add a column that is numerical ordering of 1...144 organisms and repeats for each sample
bins_count_vector <- 1:length(meta.abundance2$bin)
samples_count <- length(colnames(meta.abundance2))-1
Ranks <- rep(bins_count_vector, samples_count)
```

bin	Sample	Abundance
Rhizobiales_1	WW_2ndary_152	0
Hyphomicrobium_1	WW_2ndary_152	0
Rhizobiales_2	WW_2ndary_152	0
Bradyrhizobiaceae_1	WW_2ndary_152	0
Bradyrhizobiaceae_2	WW_2ndary_152	0
Mycobacterium_1	WW_2ndary_152	0

In [25]:
```
ranked <- cbind(ordered, Ranks)
top_ranked <- subset(ranked, ranked$Ranks<=num_rank)
top_ranked <- subset(top_ranked, top_ranked$Abundance>0)
```

In [26]:
```
options(repr.plot.width = 15, repr.plot.height = 15)

ggplot(top_ranked, aes(x=Ranks, y=Abundance)) +
  geom_line() +
  geom_point(aes(color=bin), size=5) +
  ylab("Coverage per 10 million reads") +
  expand_limits(x = c(0,15), y = c(0,200)) +
  geom_text(aes(label=bin, vjust = -1, hjust = -.1), size = 3.5,  angle = 45) +
  theme(panel.background = element_blank(), axis.line = element_line(color = "black"), panel.grid.minor = element_blank(), panel.border = element_rect(color = "b
lack", fill = NA), strip.background = element_blank()) +
  theme(legend.position="none") +
  facet_wrap(~ Sample, ncol=3)

#ggsave(width=20, height=12, filename="/SCIENCE/Nelson_lab/data_files_nelson/el_paso_metagenomics/rank_abundance_curves.pdf", device="pdf", useDingbats=FALSE)
```

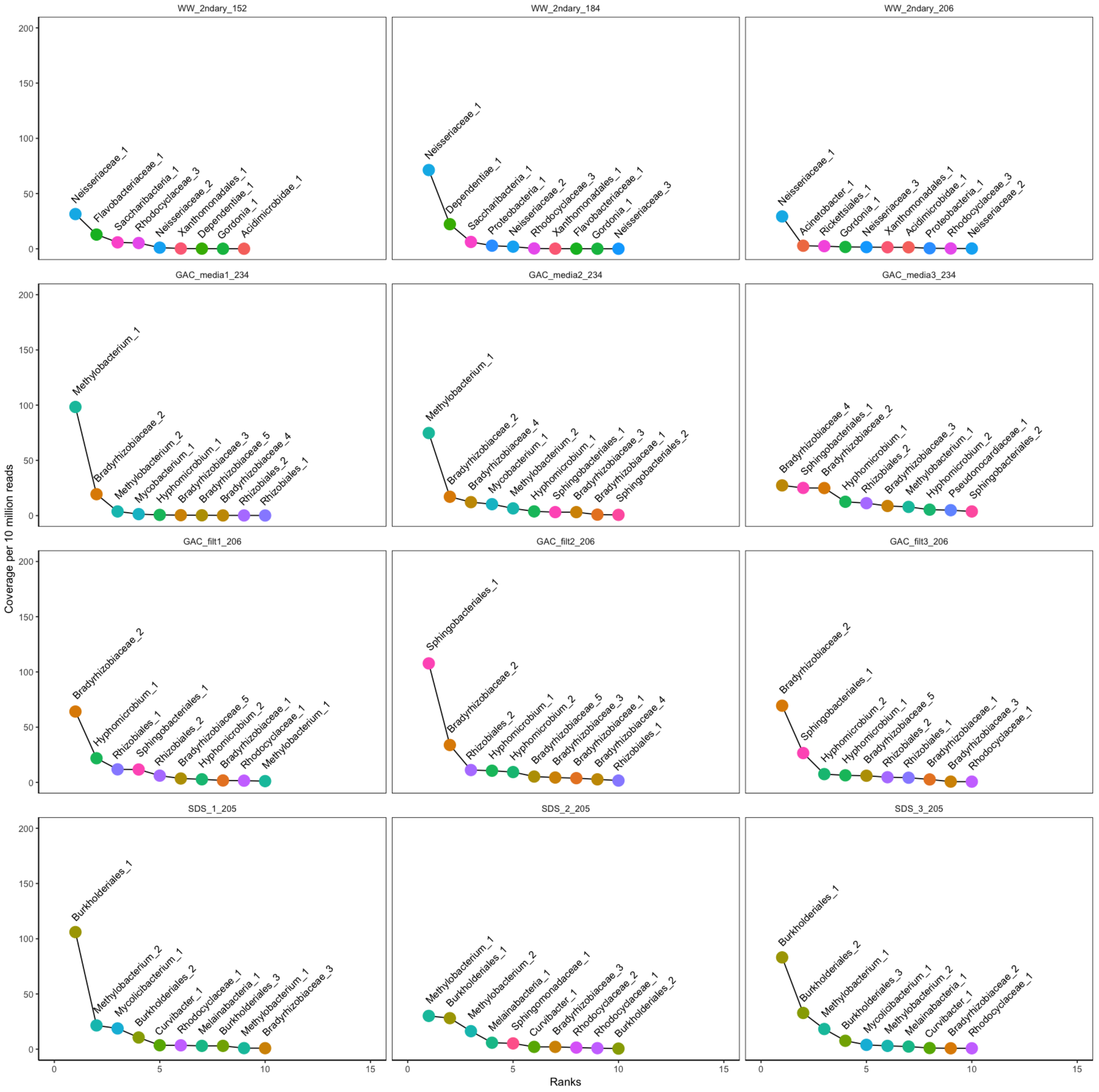

ARGs

Make heatmap of ARGs by MAG and barplot of summed coverages of ARGs by sample

In [27]:
```
#Import data
args <- read.table("/SCIENCE/Nelson_lab/data_files_nelson/el_paso_metagenomics/args/resfams.vs.all.binned.out", quote = "")[1:7]
colnames(args) <- c("bins", "geneID", "accession", "query_name", "query_accession","full_eval", "full_score")

#parse hmmsearch output to keep only the highest scoring hit for each gene (remove redundancy)
    #group by geneID and then filter for the top 1 highest score

dim(args)
args.filtered <- args %>% group_by(geneID) %>% top_n(n = 1, wt = full_score)
dim(args.filtered)
#head(args.filtered)
length(unique(args.filtered$query_name))

#write this filtered table to use it for clustering ARGs at 99% ID
#write.table(args.filtered, "/SCIENCE/Nelson_lab/data_files_nelson/el_paso_metagenomics/args/resfams.vs.all.binned.filtered.out", sep="\t", row.names=FALSE, quot
e=FALSE)
head(args.filtered)
```

1958 7

1609 7

34

bins	geneID	accession	query_name	query_accession	full_eval	full_score
GAC_media1_234_MAG_00002	sample_185_scaffold_650_16	-	ABC_efflux	RF0007	2.7e-95	321.3
GAC_media1_234_Bin_00004	sample_185_scaffold_911_3	-	ABC_efflux	RF0007	8.4e-90	303.2
GAC_media1_234_Bin_00004	sample_185_scaffold_121_1	-	ABC_efflux	RF0007	8.7e-90	303.1
GAC_media1_234_MAG_00002	sample_185_scaffold_193_25	-	ABC_efflux	RF0007	2.7e-88	298.2
GAC_media1_234_MAG_00003	sample_185_scaffold_311_6	-	ABC_efflux	RF0007	3.0e-88	298.1
no_bin	sample_185_scaffold_5488_1	-	ABC_efflux	RF0007	1.6e-86	292.4

In [28]:
```
#add in scaffold coverage info and total read counts by sample
    #mapped reads to own assembly of scaffolds > 1 kbp
    #parsed with calculate_coverage.py (Chris Brown, ctbBio on github)

scaf_cov <- read.table("/SCIENCE/Nelson_lab/data_files_nelson/el_paso_metagenomics/all_samples_min1000_coverage.txt",
                       col.names=c("scaffold", "length", "coverage"))

#merge with ARG counts
args_cov <- extract(args.filtered, col=geneID, into=c("scaffold"), regex="(sample_.*_scaffold_.*)_.*", remove=FALSE)
args_cov <- extract(args_cov, col=scaffold, into=c("sample"), regex="(sample_.*)_scaffold_.*", remove=FALSE)
args_cov <- merge(args_cov, scaf_cov, by="scaffold", all.x=TRUE, all.y=FALSE)

#merge with readcounts and sample names
metagenome_info <- read.table("/SCIENCE/Nelson_lab/data_files_nelson/el_paso_metagenomics/metagenome_info.txt",
    header=TRUE, sep="\t")
metagenome_reads <- subset(metagenome_info, select=c(Sample_code, Sample_number, Total_reads))
args_cov <- merge(args_cov, metagenome_reads, by.x="sample", by.y="Sample_number", all.x = TRUE)

#Normalize to coverage per 10 million reads
args_cov$normalized_cov <- (args_cov$coverage * 10000000 / args_cov$Total_reads)
```

Barplot of summed normalized coverage by sample

In [29]:
```
#cast data to summarize and melt into long format
args_to_cast <- select(args_cov, Sample_code, query_name, normalized_cov)
#args_normcov.x.sample <- dcast(data=args_to_cast, Sample_code ~ query_name, value.var="normalized_cov", fun.aggregate = sum)
args_normcov.x.sample <- dcast(data=args_to_cast, Sample_code ~ query_name, value.var="normalized_cov", fun.aggregate = length)

#remove regulators and efflux pumps
args_normcov.x.sample <- subset(args_normcov.x.sample, select= -c(RND_efflux, ABC_efflux, vanS, soxR, baeR))
#turn sample code into rownames for normalization, then turn back into a column
#row.names(args_normcov.x.sample) <- args_normcov.x.sample$Sample_code
#args_hellinger.x.sample <- subset(args_normcov.x.sample, select=-c(Sample_code))
#args_hellinger.x.sample <- decostand(args_hellinger.x.sample, method="hellinger") #rows are sites
#args_hellinger.x.sample$Sample_code <- row.names(args_hellinger.x.sample)

args_normcov.sample.long <- melt(args_normcov.x.sample,
                                 id.vars = "Sample_code",
                                 variable.name = "ARG",
                                 value.name = "Gene_count")

#add "Type" column for faceting in ggplot
sample2type <- data.frame(Sample_code=c("WW_2ndary_152", "WW_2ndary_184", "WW_2ndary_206",
            "GAC_media1_234", "GAC_media2_234", "GAC_media3_234",
            "GAC_filt1_206", "GAC_filt2_206", "GAC_filt3_206",
            "SDS_1_205", "SDS_2_205", "SDS_3_205"),
                          Type=c("WW_2ndary","WW_2ndary","WW_2ndary",
                                "GAC_media","GAC_media","GAC_media",
                                "GAC_filt","GAC_filt","GAC_filt",
                                "SDS","SDS","SDS"))
args_normcov.sample.long <- merge(args_normcov.sample.long, sample2type, by="Sample_code")
args_normcov.sample.long$Type <- factor(args_normcov.sample.long$Type, levels=c("WW_2ndary", "GAC_filt", "GAC_media", "SDS"))

options(repr.plot.width = 9, repr.plot.height = 6) #for plotting size in jupyter
ggplot(args_normcov.sample.long, aes(x=Sample_code, y=Gene_count, fill=ARG))+
geom_bar(stat="identity", color="black")+
theme(panel.background=element_blank(), panel.border=element_rect(color = "black", fill = NA),
        axis.text.x = element_text(angle = 45, hjust = 1, vjust = 1)) +
scale_fill_manual(values = colors) +
#ylab("Aggregated gene coverage per 10 million reads")+
ylab("Hellinger-transformed gene copies")+
xlab("Sample")+
facet_wrap(~Type, scales="free_x", ncol=4)
#ggsave("/SCIENCE/Nelson_lab/data_files_nelson/el_paso_metagenomics/args/arg_sum_normcov_by_sample.pdf", device="pdf", width=9, height=6)
```

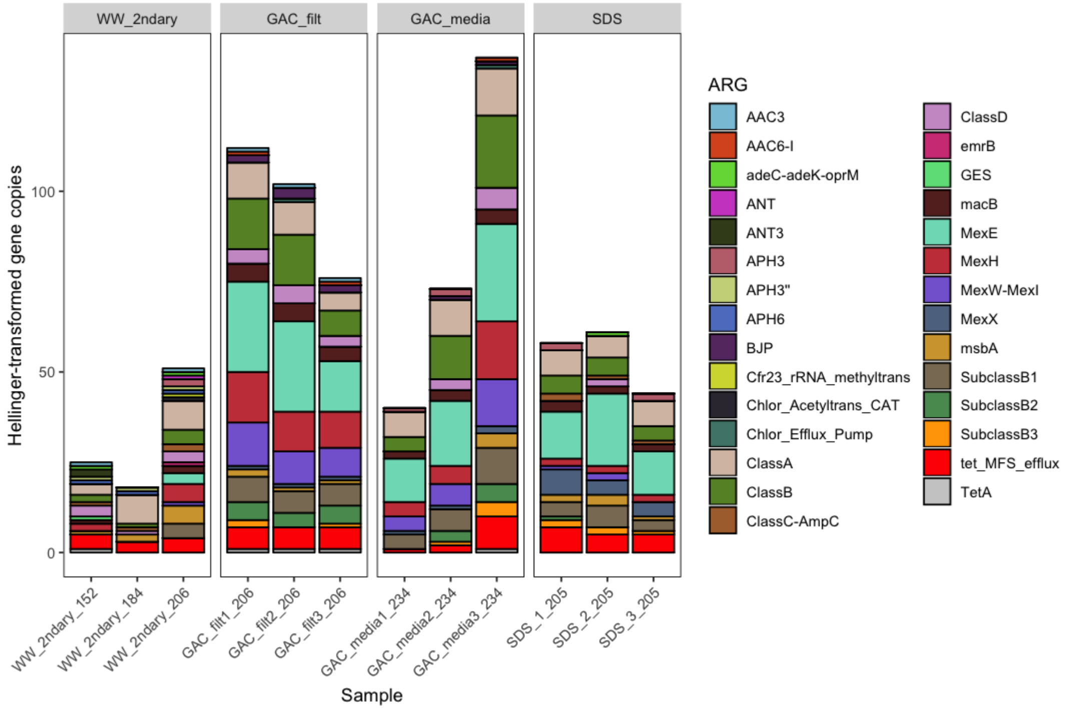

Heatmap of MAGs x ARGs

In [30]:
```
#subset args_cov to include only dereplicated MAGs based on this table
mag_info <- read.table("/SCIENCE/Nelson_lab/data_files_nelson/el_paso_metagenomics/anvio_work/genomes_info_091418.txt",
                       sep="\t", row.names = 1, header=TRUE)

#keep only dRep non-redundant genomes
mag_nonredundant <- mag_info[!is.na(mag_info$is_winner),]
mag_nonredundant <- select(mag_nonredundant, Genome_short_name)
args_cov_nonredundant <- merge(mag_nonredundant, args_cov, by.x=0, by.y="bins", all.x=TRUE, all.y=FALSE)
args_to_cast_mags <- select(args_cov_nonredundant, Genome_short_name, query_name, normalized_cov)

#pivot table to MAGs x ARGs
mag.x.arg <- dcast(data=args_to_cast_mags, Genome_short_name ~ query_name, value.var="normalized_cov", fun.aggregate = length)
row.names(mag.x.arg) <- mag.x.arg$Genome_short_name
mag.x.arg <- select(mag.x.arg, -Genome_short_name)

mag.x.arg <- as.matrix(mag.x.arg) #convert to matrix for plotting
mag.x.arg <- ifelse(mag.x.arg==0, NA, mag.x.arg) #convert zeros to NA for plotting black
mag.x.arg <- as.data.frame(mag.x.arg)

#remove ARGs that regulators
mag.x.arg <- subset(mag.x.arg, select= -c(vanS, soxR, baeR))
#mag.x.arg <- subset(mag.x.arg, select= -c(RND_efflux, ABC_efflux))

#sort by meta.ab dataframe rownames so that cluster_rows will match the clustering for MAG abundance and irep
mag.x.arg <- mag.x.arg[match(rownames(t(meta.logabundance)), rownames(mag.x.arg)), ]


#sort ARGs by function in heatmaps:
arg2function_mags <- read.table("/SCIENCE/Nelson_lab/data_files_nelson/el_paso_metagenomics/args/ResFam_arg_names2functions_mags.txt",
                            header=TRUE, row.names=1, sep="\t", quote="")
colnames(arg2function_mags) <- c("type")
#sort by dataframe rownames so that cluster_rows will group ARGs by classes
tmag.x.arg_heatmap <- as.data.frame(t(mag.x.arg)) #first transform so that ARGs are rownames in both dataframes

mag.x.arg_heatmap_sorted <- tmag.x.arg_heatmap[match(rownames(arg2function_mags), rownames(tmag.x.arg_heatmap)), ]
mag.x.arg_heatmap <- t(mag.x.arg_heatmap_sorted)

#corrdist_dissimilarity_args <- as.dist(1 - cor(mag.x.arg_heatmap, method="pearson"))
#arg_clust <- hclust(corrdist_dissimilarity_args)

options(repr.plot.width = 4.5, repr.plot.height = 6)
colors2 = colorRampPalette(c("white", "black"))
pheatmap(mag.x.arg_heatmap,
         cluster_cols=FALSE,
         #cluster_cols = arg_clust,
         cluster_rows=genome_clust, #keep genome clustering same as for abundance heatmap
         #cluster_rows=FALSE,
         na_col="white",
         color=colors2(15)[5:15],
         #show_rownames=FALSE,
         treeheight_row=0,
         treeheight_col=0)
#filename="/SCIENCE/Nelson_lab/data_files_nelson/el_paso_metagenomics/mags_args-some_heatmap.pdf", width=4.5, height=6.5)
```

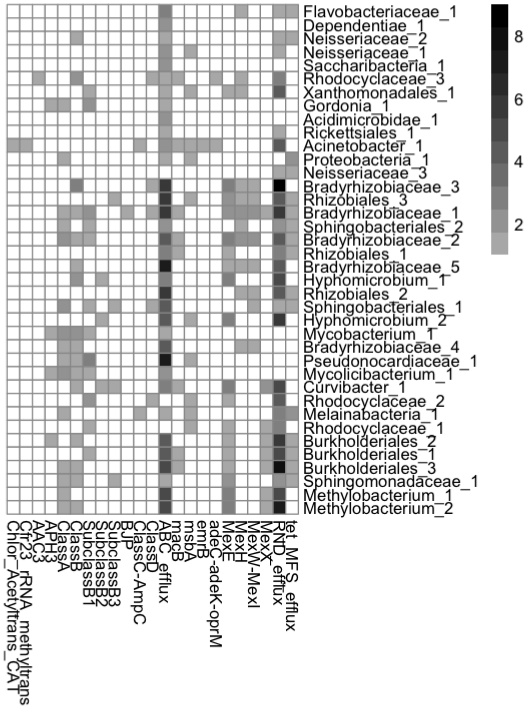

In [31]:
```
#making collapsed heatmap with category and count of ARG type
mag.x.arg_collapse <- as.data.frame(mag.x.arg_heatmap)
mag.x.arg_collapse <- select(mag.x.arg_collapse, -ABC_efflux, -RND_efflux) #remove these bc of non-specific hits
mag.x.arg_collapse <- as.data.frame(t(mag.x.arg_collapse))#transform
mag.x.arg_collapse <- merge(mag.x.arg_collapse, arg2function_mags, by=0, all.x=TRUE, all.y=FALSE)#get ARG type
mag.x.arg_collapse[is.na(mag.x.arg_collapse)] <- 0

#melt so it can be summarized with dplyr and recast
mag.x.arg_melt <- melt(mag.x.arg_collapse, id.vars=c("Row.names", "type"), value.name = "count", variable.name = "genome")
mag.x.arg_sum <- mag.x.arg_melt %>% group_by(type, genome) %>% summarise(arg_count=sum(count))

mag.x.arg_collapse <- dcast(mag.x.arg_sum, type ~ genome, value.var = "arg_count", fun.aggregate = sum)
arg_category_order <- data.frame(type=c(
                                        "Aminoglycoside Modifying Enzyme",
                                        "Chloramphenicol Resistance",
                                        "rRNA Methyltransferase",
                                        "MFS Transporter",
                                        "Beta-Lactamase",
                                        "RND Antibiotic Efflux",
                                        "ABC Transporter",
                                        "Tetracycline MFS Efflux"))

row.names(mag.x.arg_collapse) <- mag.x.arg_collapse$type
mag.x.arg_collapse <- select(mag.x.arg_collapse, -type)
mag.x.arg_collapse_sorted <- mag.x.arg_collapse[match(arg_category_order$type, row.names(mag.x.arg_collapse)), ]

mag.x.arg_collapse_sorted <- t(mag.x.arg_collapse_sorted)

options(repr.plot.width = 3.5, repr.plot.height = 7)
colors2 = colorRampPalette(c("white", "red"))
pheatmap(mag.x.arg_collapse_sorted,
         cluster_cols=FALSE,
         #cluster_cols = arg_clust,
         cluster_rows=genome_clust, #keep genome clustering same as for abundance heatmap
         #cluster_rows=FALSE,
         color=colors2(15),
         #show_rownames=FALSE,
         treeheight_row=0,
         treeheight_col=0)
#filename="/SCIENCE/Nelson_lab/data_files_nelson/el_paso_metagenomics/mags_args_final.pdf", width=3.5, height=7)
```

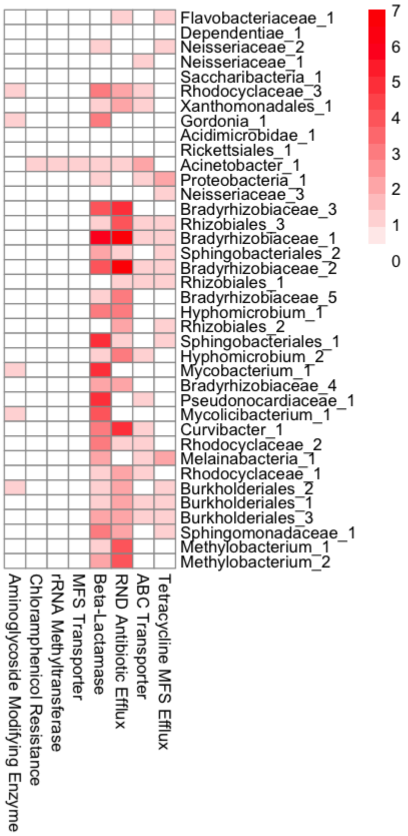

In [32]:
```
#checking what is unbinned? Can't do this by sample and display in this heatmap...
#if bins does not contain "MAG", then select the row and make new table of just those rows
non_MAGs_args <- dplyr::filter(args_cov, !grepl("MAG",bins))

non_MAGs_args.x.sample <- dcast(data=non_MAGs_args, Sample_code ~ query_name, value.var="normalized_cov", fun.aggregate = length)

#order by sample
non_MAGs_args.x.sample$Sample_code <- factor(non_MAGs_args.x.sample$Sample_code, levels=c(
    'WW_2ndary_152', 'WW_2ndary_184', 'WW_2ndary_206',
    'GAC_media1_234', 'GAC_media2_234', 'GAC_media3_234',
    'GAC_filt1_206', 'GAC_filt2_206', 'GAC_filt3_206',
    'SDS_1_205', 'SDS_2_205', 'SDS_3_205'))
non_MAGs_args.x.sample <- non_MAGs_args.x.sample[order(non_MAGs_args.x.sample$Sample_code),]

row.names(non_MAGs_args.x.sample) <- non_MAGs_args.x.sample$Sample_code
non_MAGs_args.x.sample <- subset(non_MAGs_args.x.sample, select=-c(Sample_code))

non_MAGs_args.x.sample <- as.matrix(non_MAGs_args.x.sample)
non_MAGs_args.x.sample <- ifelse(non_MAGs_args.x.sample==0, NA, non_MAGs_args.x.sample) #convert zeros to NA for plotting black
non_MAGs_args.x.sample <- as.data.frame(non_MAGs_args.x.sample)


options(repr.plot.width = 4.5, repr.plot.height = 3)
colors2 = colorRampPalette(c("white", "black"))
pheatmap(non_MAGs_args.x.sample,
         cluster_cols=FALSE,
         cluster_rows=FALSE,
         na_col="white",
         color=colors2(15)[5:15],
         #show_rownames=FALSE,
         treeheight_row=0,
         treeheight_col=0)
#filename="/SCIENCE/Nelson_lab/data_files_nelson/el_paso_metagenomics/non-mags_args_heatmap.pdf", width=4.5, height=3)
```

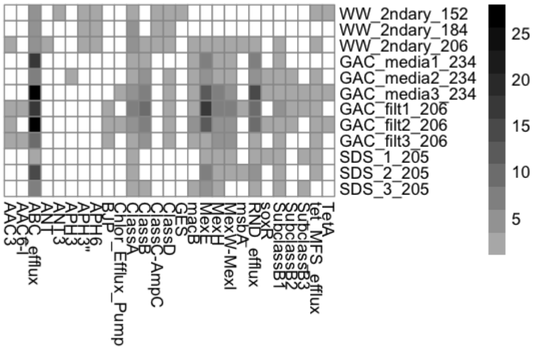

In [33]:
```
#make heatmap of all ARGs x Sample
#args_hellinger.x.sample
args_normcov.x.sample$Sample_code <- factor(args_normcov.x.sample$Sample_code, levels=c(
#args_hellinger.x.sample$Sample_code <- factor(args_hellinger.x.sample$Sample_code, levels=c(

    'WW_2ndary_152', 'WW_2ndary_184', 'WW_2ndary_206',
    'GAC_media1_234', 'GAC_media2_234', 'GAC_media3_234',
    'GAC_filt1_206', 'GAC_filt2_206', 'GAC_filt3_206',
    'SDS_1_205', 'SDS_2_205', 'SDS_3_205'))

args_normcov.x.sample_heatmap <- args_normcov.x.sample[order(args_normcov.x.sample$Sample_code),]
#args_normcov.x.sample_heatmap <- args_hellinger.x.sample[order(args_hellinger.x.sample$Sample_code),]

row.names(args_normcov.x.sample_heatmap) <- args_normcov.x.sample_heatmap$Sample_code

args_normcov.x.sample_heatmap <- subset(args_normcov.x.sample_heatmap, select=-c(Sample_code))

corrdist_dissimilarity <- as.dist(1 - cor(args_normcov.x.sample_heatmap, method="spearman"))
arg_clust <- hclust(corrdist_dissimilarity)

#convert zeros to NA (must be converted to matrix first)
args_normcov.x.sample_heatmap <- as.matrix(args_normcov.x.sample_heatmap)
args_normcov.x.sample_heatmap <- ifelse(args_normcov.x.sample_heatmap==0, NA, args_normcov.x.sample_heatmap)
args_normcov.x.sample_heatmap.log <- log10(args_normcov.x.sample_heatmap)

#sort ARGs by function in heatmaps:
arg2function <- read.table("/SCIENCE/Nelson_lab/data_files_nelson/el_paso_metagenomics/args/ResFam_arg_names2functions.txt",
                           header=TRUE, row.names=1, sep="\t", quote="")
targs_normcov.x.sample_heatmap <- t(args_normcov.x.sample_heatmap)

#sort by dataframe rownames so that cluster_rows will group ARGs by classes
targs_normcov.x.sample_heatmap <- targs_normcov.x.sample_heatmap[match(rownames(arg2function), rownames(targs_normcov.x.sample_heatmap)), ]

options(repr.plot.width = 4.5, repr.plot.height = 5)

pheatmap(targs_normcov.x.sample_heatmap,
         color=rev(magma(15)[1:14]),
         cluster_rows=FALSE,
         #cluster_rows=arg_clust,
         cluster_cols=FALSE,
         na_col="white")
#save as pdf (uncomment these rows)
#filename="/SCIENCE/Nelson_lab/data_files_nelson/el_paso_metagenomics/args/arg_sum_normcov_by_sample_heatmap_class.pdf", width=4.5, height=6)
```

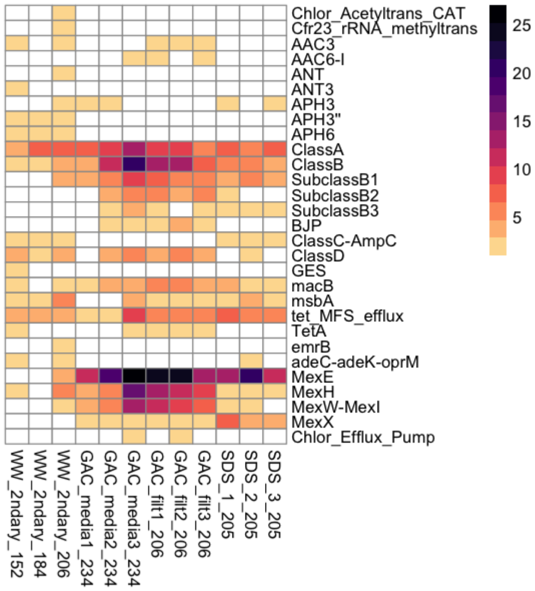

In [34]:
```
#sort ARGs by function in heatmaps:
arg2function <- read.table("/SCIENCE/Nelson_lab/data_files_nelson/el_paso_metagenomics/args/ResFam_arg_names2functions.txt",
                           header=TRUE, row.names=1, sep="\t", quote="")#, stringsAsFactors = FALSE, allowEscapes = TRUE)
targs_normcov.x.sample_heatmap <- t(args_normcov.x.sample_heatmap)

#sort by meta.ab dataframe rownames so that cluster_rows will match the clustering for MAG abundance and irep
targs_normcov.x.sample_heatmap <- targs_normcov.x.sample_heatmap[match(rownames(arg2function), rownames(targs_normcov.x.sample_heatmap)), ]
```

Normalize to coverage of ribosomal protein S3: For each sample: sum of args coverages / sum of rps3 coverages

(sum of args coverages) / (sum of rps3 coverages)

Gene count was also lower, not just explained by % reads mapping to assembly or assembly size. Poorer assembly?

In [35]:
```
#coverages of RPS3 genes: Do these show a similar pattern, indicative of higher coverage in less diverse samples?
rps3_ab <- read.table(
    "/SCIENCE/Nelson_lab/data_files_nelson/el_paso_metagenomics/rps3_analyses/mean_coverage_Q2Q3_contigs.txt",
    sep="\t", header=TRUE)
names(rps3_ab) <- c("split", "WW_2ndary_152", "WW_2ndary_184", "WW_2ndary_206",
                    "GAC_filt1_206", "GAC_filt2_206", "GAC_filt3_206",
                    "GAC_media1_234", "GAC_media2_234", "GAC_media3_234",
                    "SDS_1_205", "SDS_2_205", "SDS_3_205", "contig")
rps3_covsum <- as.data.frame(colSums(rps3_ab[2:13]))
names(rps3_covsum) <- c("rps3_summed_cov")
head(rps3_covsum)
```

	rps3_summed_cov
**WW_2ndary_152**	266.9387
**WW_2ndary_184**	428.4576
**WW_2ndary_206**	261.4262
**GAC_filt1_206**	625.2515
**GAC_filt2_206**	524.1912
**GAC_filt3_206**	598.0730

In [36]:
```
##repeat the wrangling from above but don't normalize coverage to read counts this time. Prepare to normalize by rps3.

#cast data to summarize and melt into long format
args_to_cast <- select(args_cov, Sample_code, query_name, coverage)
args_cov.x.sample <- dcast(data=args_to_cast, Sample_code ~ query_name, value.var="coverage", fun.aggregate = sum)

#merge with rps3 summed coverages
args_cov.x.sample <- merge(args_cov.x.sample, rps3_covsum, by.x="Sample_code", by.y=0)
#remove regulators and efflux pumps
args_cov.x.sample <- subset(args_cov.x.sample, select= -c(RND_efflux, ABC_efflux, vanS, soxR, baeR))

#normalize
args_cov.x.sample[2:length(colnames(args_cov.x.sample))] <- args_cov.x.sample[2:length(colnames(args_cov.x.sample))] /args_cov.x.sample$rps3_summed_cov
args_cov.x.sample <- subset(args_cov.x.sample, select= -c(rps3_summed_cov))

args_cov.sample.long <- melt(args_cov.x.sample,
                                  id.vars = "Sample_code",
                                  variable.name = "ARG",
                                  value.name = "Gene_count" )

#add "Type" column for faceting by sample type in ggplot
sample2type <- data.frame(Sample_code=c("WW_2ndary_152", "WW_2ndary_184", "WW_2ndary_206",
             "GAC_media1_234", "GAC_media2_234", "GAC_media3_234",
             "GAC_filt1_206", "GAC_filt2_206", "GAC_filt3_206",
             "SDS_1_205", "SDS_2_205", "SDS_3_205"),
                          Type=c("WW_2ndary","WW_2ndary","WW_2ndary",
                                 "GAC_media","GAC_media","GAC_media",
                                 "GAC_filt","GAC_filt","GAC_filt",
                                 "SDS","SDS","SDS"))

args_cov.sample.long <- merge(args_cov.sample.long, sample2type, by="Sample_code")

args_cov.sample.long$Type <- factor(args_cov.sample.long$Type, levels=c("WW_2ndary", "GAC_filt", "GAC_media", "SDS"))

options(repr.plot.width = 9, repr.plot.height = 6) #for plotting size in jupyter
ggplot(args_cov.sample.long, aes(x=Sample_code, y=Gene_count, fill=ARG))+
geom_bar(stat="identity", color="black")+
theme(panel.background=element_blank(), panel.border=element_rect(color = "black", fill = NA),
          axis.text.x = element_text(angle = 45, hjust = 1, vjust = 1)) +
scale_fill_manual(values = colors) +
ylab("Aggregated gene coverage per aggregated ribosomal protein S3 coverage")+
xlab("Sample")+
facet_wrap(~Type, scales="free_x", ncol=4)
#ggsave("/SCIENCE/Nelson_lab/data_files_nelson/el_paso_metagenomics/args/arg_sum_per_rps3_sumcov_by_sample.pdf", device="pdf", width=9, height=6)
```

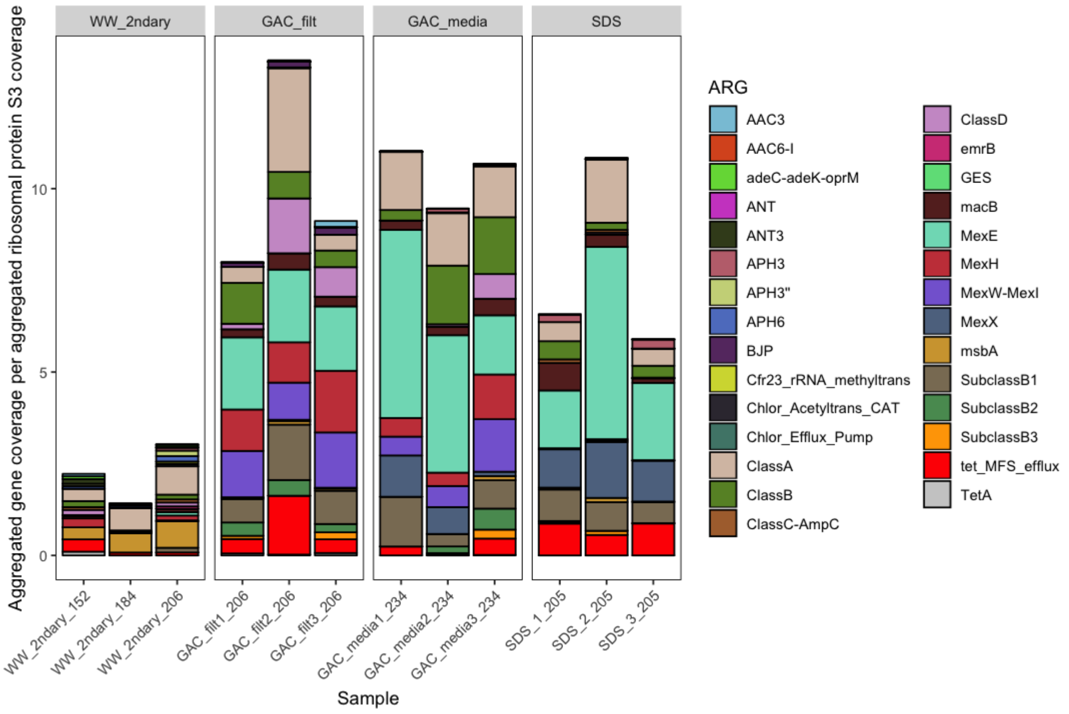

Carbon utilization

In [37]:
```
#manual lists of genes based on kegg and alignments
#used keywords and ECs combined to pull out
#1) methanol dehydrogenases (indicative of facultative and obligate methylotrophs)
#2) formaldehyde dehydrogenases (key in conversion of C1 for energy generation or growth)
#3) four key enzymes of serine cycle (anabolic pathway for growth on C1)
#4) formate dehydrogenase (all types, for energy generation on C1)
#5) RuBisCo (alternative means of fixing carbon while catabolizing C1 compounds)
# skipped RuMP pathway here (the third alternative way of fixing C1)

carbon_genes_kegg <- read.table("/SCIENCE/Nelson_lab/data_files_nelson/el_paso_metagenomics/carbon_utilization/kegg_results/methanol_formaldehyde_lists.binned.tx
t", sep="\t")
colnames(carbon_genes_kegg) <- c("bins", "id", "annotation")
mag.x.carbon.kegg <- dcast(data=carbon_genes_kegg, bins ~ annotation, value.var="id", fun.aggregate = length)

#subset carbon_genes to include only dereplicated MAGs based on this table
mag_info <- read.table("/SCIENCE/Nelson_lab/data_files_nelson/el_paso_metagenomics/anvio_work/genomes_info_091418.txt",
                       sep="\t", row.names = 1, header=TRUE)

#keep only dRep non-redundant MAGs for this analysis
mag_nonredundant <- mag_info[!is.na(mag_info$is_winner),]
mag_nonredundant <- select(mag_nonredundant, Genome_short_name)
mag.x.carbon.kegg <- merge(mag_nonredundant, mag.x.carbon.kegg, by.x=0, by.y="bins", all.x=TRUE, all.y=FALSE)
row.names(mag.x.carbon.kegg) <- mag.x.carbon.kegg$Genome_short_name
mag.x.carbon.kegg <- select(mag.x.carbon.kegg, -Row.names, -Genome_short_name)


#sort columns so the genes are grouped by pathway in a specific order
carbon_gene2pathway <- c(
"PQQ_dependent_methanol_dehydrogenase",
"Formaldehyde_activating_enzyme",
"Glutathione_dependent_formaldehyde_activating_protein",
"Glutathione_independent_formaldehyde_dehydrogenase",
"Glycerate_kinase",
"Malate_CoA_ligase",
"Malyl_CoA_lyase",
"Hydroxypyruvate_reductase",
"RuBisCo_large_subunit",
"Formate_dehydrogenase_alpha",
"Formate_dehydrogenase_unknown"
)
carbon_gene2pathway <- as.data.frame(carbon_gene2pathway)

tmag.x.carbon.kegg <- t(mag.x.carbon.kegg)

tmag.x.carbon.kegg <- tmag.x.carbon.kegg[match(carbon_gene2pathway$carbon_gene2pathway,
                                               rownames(tmag.x.carbon.kegg)), ]
mag.x.carbon.kegg <- t(tmag.x.carbon.kegg)
#sort rows so they can be clustered in the same order as other heatmaps
mag.x.carbon.kegg <- mag.x.carbon.kegg[match(rownames(t(meta.logabundance)), rownames(mag.x.carbon.kegg)), ]

mag.x.carbon.kegg <- as.matrix(mag.x.carbon.kegg)
mag.x.carbon.kegg <- ifelse(mag.x.carbon.kegg==0, NA, mag.x.carbon.kegg)

options(repr.plot.width = 4, repr.plot.height = 8)
colors2 = colorRampPalette(c("white", "black"))
pheatmap(mag.x.carbon.kegg,
         cluster_cols=FALSE,
         #cluster_cols = arg_clust,
         cluster_rows=genome_clust, #keep genome clustering same as for abundance heatmap
         #cluster_rows=FALSE,
         na_col="white",
         color=colors2(15)[5:15],
         #show_rownames=FALSE,
         treeheight_row=0,
         treeheight_col=0)
```

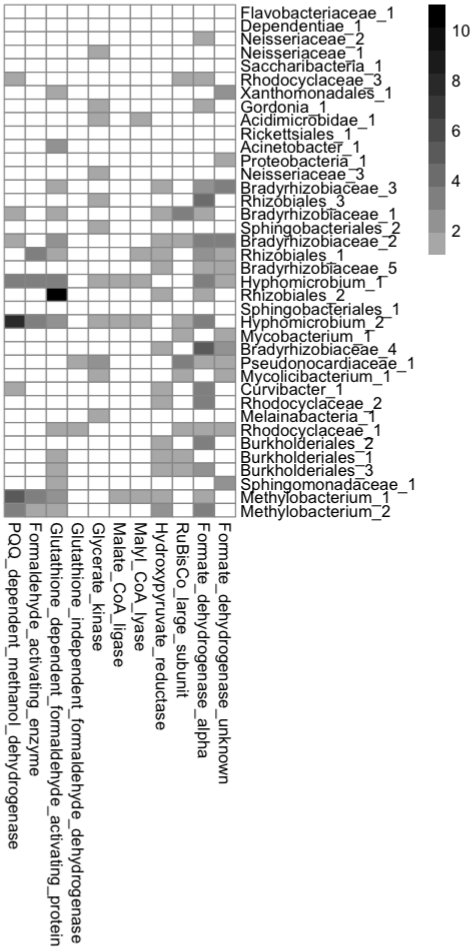
Flavobacteriaceae_1
Dependentiae_1
Neisseriaceae_2
Neisseriaceae_1
Saccharibacteria_1
Rhodocyclaceae_3
Xanthomonadales_1
Gordonia_1
Acidimicrobidae_1
Rickettsiales_1
Acinetobacter_1
Proteobacteria_1
Neisseriaceae_3
Bradyrhizobiaceae_3
Rhizobiales_3
Bradyrhizobiaceae_1
Sphingobacteriales_2
Bradyrhizobiaceae_2
Rhizobiales_1
Bradyrhizobiaceae_5
Hyphomicrobium_1
Rhizobiales_2
Sphingobacteriales_1
Hyphomicrobium_2
Mycobacterium_1
Bradyrhizobiaceae_4
Pseudonocardiaceae_1
Mycolicibacterium_1
Curvibacter_1
Rhodocyclaceae_2
Melainabacteria_1
Rhodocyclaceae_1
Burkholderiales_2
Burkholderiales_1
Burkholderiales_3
Sphingomonadaceae_1
Methylobacterium_1
Methylobacterium_2
10
8
6
4
2
PQQ_dependent_methanol_dehydrogenase
Formaldehyde_activating_enzyme
Glutathione_dependent_formaldehyde_activating_protein
Glutathione_independent_formaldehyde_dehydrogenase
Glycerate_kinase
Malate_CoA_ligase
Malyl_CoA_lyase
Hydroxypyruvate_reductase
RuBisCo_large_subunit
Formate_dehydrogenase_alpha
Formate_dehydrogenase_unknown

In [38]:

```
#collapsing to 5 columns: methanol dehydrogenase, formaldehyde dehydrogenase, serine cycle, formate dehydrogenase, rubisco

#convert to present/absense:
mag.x.carbon.kegg_PA <- ifelse(is.na(mag.x.carbon.kegg)==TRUE, 0, 1)
mag.x.carbon.kegg_PA <- as.data.frame(mag.x.carbon.kegg_PA)
mag.x.carbon.kegg_PA$Formaldehyde <- mag.x.carbon.kegg_PA$Glutathione_dependent_formaldehyde_activating_protein + mag.x.carbon.kegg_PA$Glutathione_independent_fo
rmaldehyde_dehydrogenase
mag.x.carbon.kegg_PA$Serine_cycle <- mag.x.carbon.kegg_PA$Glycerate_kinase + mag.x.carbon.kegg_PA$Malate_CoA_ligase + mag.x.carbon.kegg_PA$Malyl_CoA_lyase + mag.
x.carbon.kegg_PA$Hydroxypyruvate_reductase
#require 3 out of 4 enzymes from serine cycle
mag.x.carbon.kegg_PA$Serine_cycle[mag.x.carbon.kegg_PA$Serine_cycle < 3] <- 0

#combine alpha and unknown formate dehydrogenases
mag.x.carbon.kegg_PA$Formate_dehydrogenase <- mag.x.carbon.kegg_PA$Formate_dehydrogenase_alpha + mag.x.carbon.kegg_PA$Formate_dehydrogenase_unknown

#select new columns
mag.x.carbon.kegg_PA <- select(mag.x.carbon.kegg_PA, PQQ_dependent_methanol_dehydrogenase, Formaldehyde, Serine_cycle, Formate_dehydrogenase, RuBisCo_large_subun
it)

#convert 0 to NA again
mag.x.carbon.kegg_PA <- as.matrix(mag.x.carbon.kegg_PA)
mag.x.carbon.kegg_PA <- ifelse(mag.x.carbon.kegg_PA>0, 1, 0)

options(repr.plot.width = 3, repr.plot.height = 8.5)
colors2 = colorRampPalette(c("white", "blue"))
pheatmap(mag.x.carbon.kegg_PA,
         cluster_cols=FALSE,
         #cluster_cols = arg_clust,
         cluster_rows=genome_clust, #keep genome clustering same as for abundance heatmap
         #cluster_rows=FALSE,
         na_col="white",
         color=colors2(15),
         #show_rownames=FALSE,
         treeheight_row=0,
         treeheight_col=0)
#filename="/SCIENCE/Nelson_lab/data_files_nelson/el_paso_metagenomics/mag.x.carbon_heatmap.final.pdf", width=3, height=7.5)
```

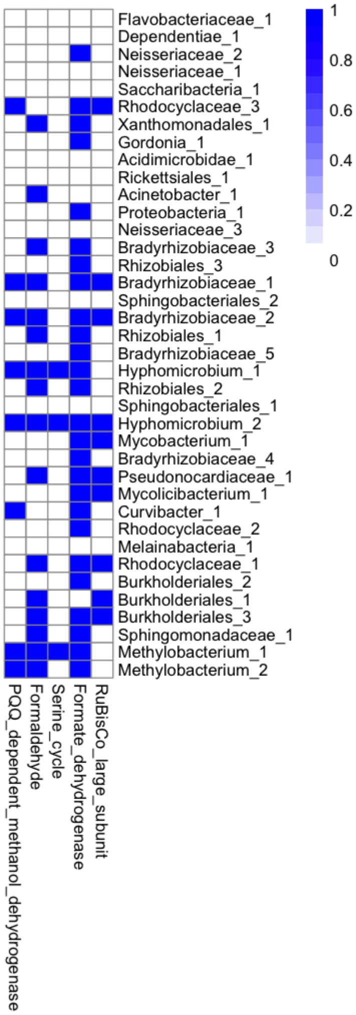

In [39]:
```
#all C1 based on usearch vs KEGG
#not all annotations had an EC number, so this method of catching proteins by EC was incomplete
#get gene names
carbon_genes_kegg <- read.table("/SCIENCE/Nelson_lab/data_files_nelson/el_paso_metagenomics/carbon_utilization/kegg_results/all_MAGs_c1_enzymes_by_EC.binned.txt"
, sep="\t")
colnames(carbon_genes_kegg) <- c("bins", "query", "target", "id",
                            "alignment_length", "mismatch", "gap",
                            "qstart", "qend", "tstart", "t_end",
                            "e_value", "bit_score", "annotation")

gene_names <- read.table("/SCIENCE/Nelson_lab/data_files_nelson/el_paso_metagenomics/carbon_utilization/ec-to-enzyme_name.txt", sep="\t", header=TRUE)
carbon_genes_kegg <- extract(carbon_genes_kegg, col=annotation, into=c("ec_number"), regex="EC:(.*)\\)")

carbon_genes_kegg <- merge(carbon_genes_kegg, gene_names, by="ec_number", all.x=TRUE, all.y=FALSE)

carbon_genes_kegg <- select(carbon_genes_kegg, bins, enzyme_name, id)
mag.x.carbon.kegg <- dcast(data=carbon_genes_kegg, bins ~ enzyme_name, value.var="id", fun.aggregate = length)

#subset carbon_genes to include only dereplicated MAGs based on this table
mag_info <- read.table("/SCIENCE/Nelson_lab/data_files_nelson/el_paso_metagenomics/anvio_work/genomes_info_091418.txt",
                       sep="\t", row.names = 1, header=TRUE)

#keep only dRep non-redundant MAGs for this analysis
mag_nonredundant <- mag_info[!is.na(mag_info$is_winner),]
mag_nonredundant <- select(mag_nonredundant, Genome_short_name)
mag.x.carbon.kegg <- merge(mag_nonredundant, mag.x.carbon.kegg, by.x=0, by.y="bins", all.x=TRUE, all.y=FALSE)
row.names(mag.x.carbon.kegg) <- mag.x.carbon.kegg$Genome_short_name
mag.x.carbon.kegg <- select(mag.x.carbon.kegg, -Row.names, -Genome_short_name)

#sort rows so they can be clustered in the same order as other heatmaps
mag.x.carbon.kegg <- mag.x.carbon.kegg[match(rownames(t(meta.logabundance)), rownames(mag.x.carbon.kegg)), ]
##
#sort columns so the genes are grouped by pathway in a specific order
tmag.x.carbon.kegg <- t(mag.x.carbon.kegg)
#tmag.x.carbon.kegg <- tmag.x.carbon.kegg[match(rownames(carbon_gene2pathway), rownames(tmag.x.carbon.kegg)), ]

tmag.x.carbon.kegg <- as.matrix(tmag.x.carbon.kegg)
tmag.x.carbon.kegg <- ifelse(tmag.x.carbon.kegg==0, NA, tmag.x.carbon.kegg)

options(repr.plot.width = 6, repr.plot.height = 9)
colors2 = colorRampPalette(c("white", "black"))
pheatmap(t(tmag.x.carbon.kegg),
         cluster_cols=FALSE,
         #cluster_cols = arg_clust,
         cluster_rows=genome_clust, #keep genome clustering same as for abundance heatmap
         #cluster_rows=FALSE,
         na_col="white",
         color=colors2(15)[5:15],
         #show_rownames=FALSE,
         treeheight_row=0,
         treeheight_col=0)
#filename="/SCIENCE/Nelson_lab/data_files_nelson/el_paso_metagenomics/mag.x.carbon_heatmap.pdf", width=5, height=8)
```

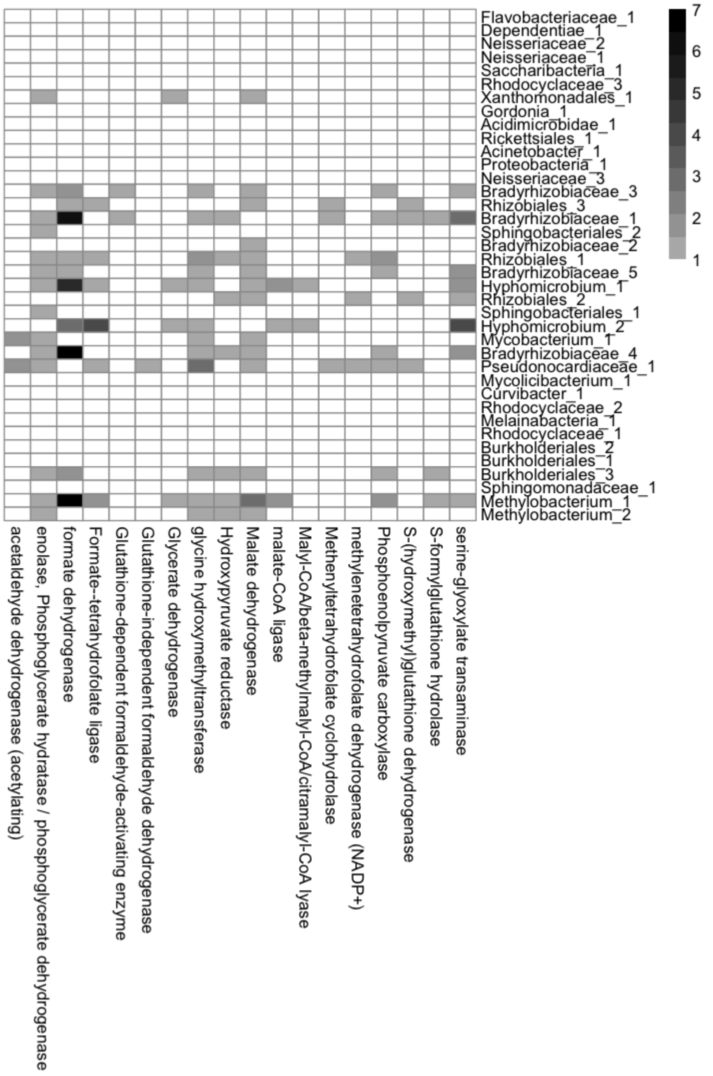
Flavobacteriaceae_1
Dependentiae_1
Neisseriaceae_2
Neisseriaceae_1
Saccharibacteria_1
Rhodocyclaceae_3
Xanthomonadales_1
Gordonia_1
Acidimicrobidae_1
Rickettsiales_1
Acinetobacter_1
Proteobacteria_1
Neisseriaceae_3
Bradyrhizobiaceae_3
Rhizobiales_3
Bradyrhizobiaceae_1
Sphingobacteriales_2
Bradyrhizobiaceae_2
Rhizobiales_1
Bradyrhizobiaceae_5
Hyphomicrobium_1
Rhizobiales_2
Sphingobacteriales_1
Hyphomicrobium_2
Mycobacterium_1
Bradyrhizobiaceae_4
Pseudonocardiaceae_1
Mycolicibacterium_1
Curvibacter_1
Rhodocyclaceae_2
Melainabacteria_1
Rhodocyclaceae_1
Burkholderiales_2
Burkholderiales_1
Burkholderiales_3
Sphingomonadaceae_1
Methylobacterium_1
Methylobacterium_2
7
6
5
4
3
2
1
acetaldehyde dehydrogenase (acetylating)
enolase, Phosphoglycerate hydratase / phosphoglycerate dehydrogenase
formate dehydrogenase
Formate--tetrahydrofolate ligase
Glutathione-dependent formaldehyde-activating enzyme
Glutathione-independent formaldehyde dehydrogenase
Glycerate dehydrogenase
glycine hydroxymethyltransferase
Hydroxypyruvate reductase
Malate dehydrogenase
malate-CoA ligase
Malyl-CoA/beta-methylmalyl-CoA/citramalyl-CoA lyase
Methenyltetrahydrofolate cyclohydrolase
methylenetetrahydrofolate dehydrogenase (NADP+)
Phosphoenolpyruvate carboxylase
S-(hydroxymethyl)glutathione dehydrogenase
S-formylglutathione hydrolase
serine-glyoxylate transaminase

In [40]:
```
#based on Prokka: (didn't use in final figure because prokka annotations were less comprehensive than KEGG)
carbon_genes <- read.table("/SCIENCE/Nelson_lab/data_files_nelson/el_paso_metagenomics/carbon_utilization/prokka_results/all_carbon_genes3.binned.txt", sep="\t")
colnames(carbon_genes) <- c("bins", "scaffold", "source", "type", "start", "stop", "matched", "strand", "eval", "annotation")
carbon_genes <- extract(carbon_genes, col=annotation, into=c("annotation_name"), regex="product=(.*)")

#change all "S-formyl glutathione hydrolase" genes to have the same name
carbon_genes[carbon_genes$annotation_name=="S-formylglutathione hydrolase FrmB",]$annotation_name <- "S-formylglutathione hydrolase"
carbon_genes[carbon_genes$annotation_name=="S-formylglutathione hydrolase YeiG",]$annotation_name <- "S-formylglutathione hydrolase"

#change Acetaldehyde dehydrogenase 4 genes to have same name (checked by alignment and they are the same)
carbon_genes[carbon_genes$annotation_name=="Acetaldehyde dehydrogenase 4",]$annotation_name <- "Acetaldehyde dehydrogenase"

#change all serine hydroxymethyltransferase genes to have same name (checked by alignment and they are the same)
carbon_genes[carbon_genes$annotation_name=="Serine hydroxymethyltransferase 1",]$annotation_name <- "Serine hydroxymethyltransferase"
carbon_genes[carbon_genes$annotation_name=="Serine hydroxymethyltransferase 2",]$annotation_name <- "Serine hydroxymethyltransferase"

#Serine-pyruvate aminotransferase is ortholog of serine-glyoxylate transaminase, in serine cycle. Prokka didn't distinguish them.
carbon_genes[carbon_genes$annotation_name=="Serine-pyruvate aminotransferase",]$annotation_name <- "Serine-pyruvate/glyoxylate aminotransferase"

#drop genes that got picked up by grep but aren't what we were looking for
drop <- c("Enolase 2","Enolase-phosphatase E1","2%2C3-diketo-5-methylthiopentyl-1-phosphate enolase", "Acetaldehyde dehydrogenase 2")
carbon_genes <- subset(carbon_genes, !(annotation_name %in% drop))

#subset carbon_genes to include only dereplicated MAGs based on this table
mag_info <- read.table("/SCIENCE/Nelson_lab/data_files_nelson/el_paso_metagenomics/anvio_work/genomes_info_091418.txt",
                       sep="\t", row.names = 1, header=TRUE)

#checking what is unbinned? Can't do this by sample and display in this heatmap...
#if bins does not contain "MAG", then select the row and make new table of just those rows
#non_MAGs_carbon_genes <- dplyr::filter(carbon_genes, !grepl("MAG",bins))

#unique(non_MAGs_carbon_genes$annotation_name) #what genes are represented in non_MAG data?

#keep only dRep non-redundant MAGs for this analysis
mag_nonredundant <- mag_info[!is.na(mag_info$is_winner),]
mag_nonredundant <- select(mag_nonredundant, Genome_short_name)
carbon_genes_nonredundant <- merge(mag_nonredundant, carbon_genes, by.x=0, by.y="bins", all.x=TRUE, all.y=FALSE)
carbon_genes_to_cast_mags <- select(carbon_genes_nonredundant, Genome_short_name, annotation_name, type)

mag.x.carbon <- dcast(data=carbon_genes_to_cast_mags, Genome_short_name ~ annotation_name, value.var="type", fun.aggregate = length)
row.names(mag.x.carbon) <- mag.x.carbon$Genome_short_name
#dcast creates a column called "NA" for genomes that contain none of the genes of interest
mag.x.carbon <- select(mag.x.carbon, -Genome_short_name)

#sort rows so they can be clustered in the same order as other heatmaps
mag.x.carbon <- mag.x.carbon[match(rownames(t(meta.logabundance)), rownames(mag.x.carbon)), ]

#sort columns so the genes are grouped by pathway in a specific order
carbon_gene2pathway <- read.table("/SCIENCE/Nelson_lab/data_files_nelson/el_paso_metagenomics/carbon_utilization/prokka_results/carbon_gene2pathway.txt",
                            header=TRUE, row.names=1, sep="\t", quote="")

tmag.x.carbon <- t(mag.x.carbon)
tmag.x.carbon <- tmag.x.carbon[match(rownames(carbon_gene2pathway), rownames(tmag.x.carbon)), ]


tmag.x.carbon <- as.matrix(tmag.x.carbon)
tmag.x.carbon <- ifelse(tmag.x.carbon==0, NA, tmag.x.carbon)

options(repr.plot.width = 6, repr.plot.height = 8)
colors2 = colorRampPalette(c("white", "black"))
pheatmap(t(tmag.x.carbon),
         cluster_cols=FALSE,
         #cluster_cols = arg_clust,
         cluster_rows=genome_clust, #keep genome clustering same as for abundance heatmap
         #cluster_rows=FALSE,
         na_col="white",
         color=colors2(15)[5:15],
         #show_rownames=FALSE,
         treeheight_row=0,
         treeheight_col=0)
#filename="/SCIENCE/Nelson_lab/data_files_nelson/el_paso_metagenomics/mag.x.carbon_heatmap.pdf", width=5, height=8)
```

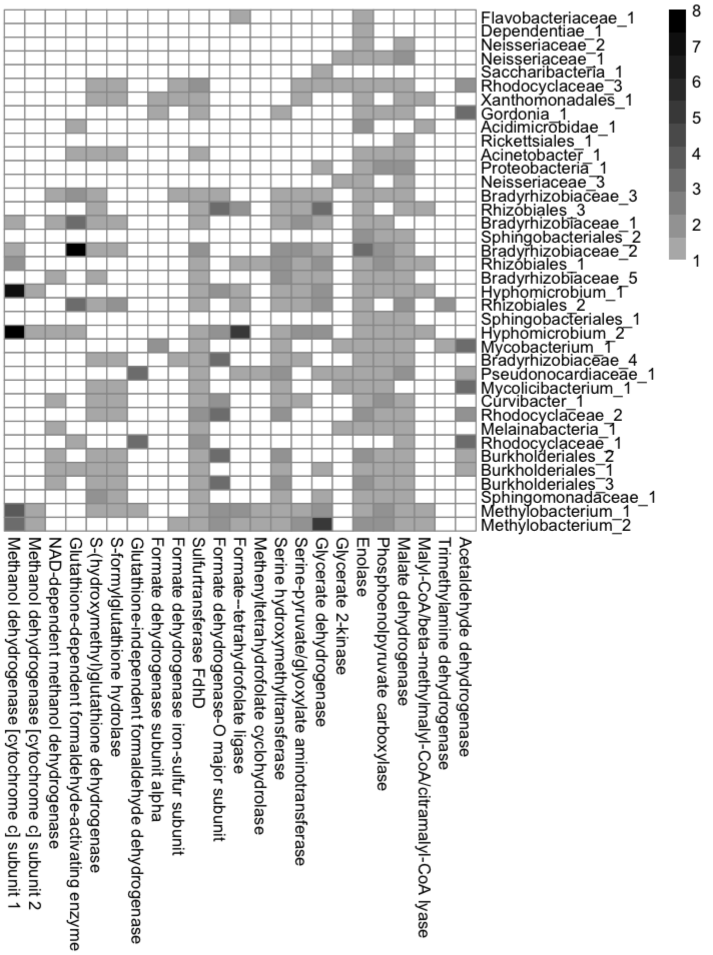

In [41]:
```
#save all MAG info for table S5
mag_all_info2 <- merge(mag_all_info, mag.x.arg_heatmap, by=0, suffixes = c("",".ARG"))
row.names(mag_all_info2) <- mag_all_info2$Row.names
mag_all_info2 <- select(mag_all_info2, -Row.names)
mag_all_info2 <- merge(mag_all_info2, mag.x.carbon.kegg, by=0)
row.names(mag_all_info2) <- mag_all_info2$Row.names
mag_all_info2 <- select(mag_all_info2, -Row.names)
#write.table(mag_all_info2, "/SCIENCE/Nelson_lab/data_files_nelson/el_paso_metagenomics/mag_all_info_supplemental.txt", sep="\t", quote=FALSE)
```
